# Selective Synthesis of Chiral Carbocycles by Iridium-Catalyzed Asymmetric Mono‑, Double‑, or Triple Hydrogenation of Cyclic Dienones

**DOI:** 10.1021/acs.orglett.5c04476

**Published:** 2025-12-22

**Authors:** Bram B. C. Peters, Jia Zheng, Haili Zhang, Pher G. Andersson

**Affiliations:** † Department of Chemistry, 7675 Stockholm University, Stockholm, 10691, Sweden; ‡ College of Chemistry and Chemical Engineering, Southwest Petroleum University, Chengdu, Sichuan 610500, China; § School of Ocean and Tropical Medicine, 12453 Guangdong Medical University, Zhanjiang, Guangdong 524023, China

## Abstract

A divergent N,P-iridium-catalyzed asymmetric hydrogenation of cyclic dienones into chiral cyclohexenones, cyclohexanones, or cyclohexanols is described. The π-bonds in cyclic dienones underwent hydrogenation in a sequential manner, favoring the (s)-*cis* conformed alkene followed by the olefin in the (s)-*trans* conformation and at last the ketone, to install up to three stereocenters in a single step. The simple choice of the proper catalyst allowed the formation of each respective product in high yield and stereopurity (up to 99% *ee*, up to 99/1 d.r.). This protocol provides an interesting opportunity to access multiple and stereopure carbocycles starting from the same precursor that otherwise require multistep syntheses.

## Graphical abstract

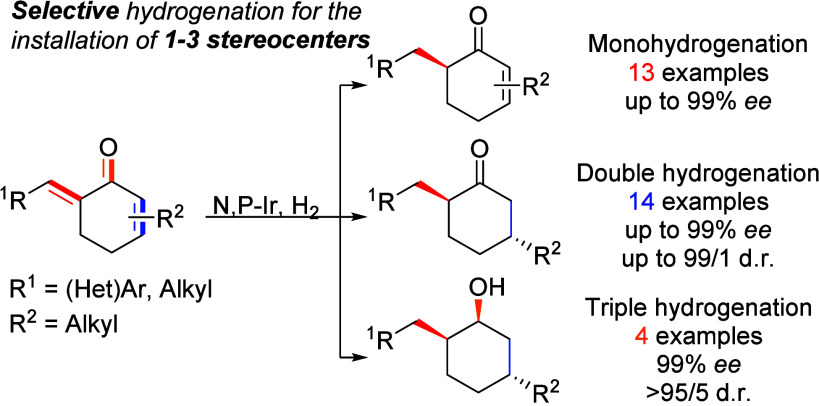

Chiral carbocycles are prominent scaffolds that are often found in natural products. Interestingly, the ring structures in these chiral motifs are constructed of a varying ratio between sp^2^ and sp^3^ hybridized carbon atoms.^1^ For example, hernandulcin, piperitone, and testosterone bear a higher fractional sp^3^ character in the carbocyclic ring compared to menthone and camphor and menthol, borneol, and fenchol (Scheme 1).

**Scheme 1:**
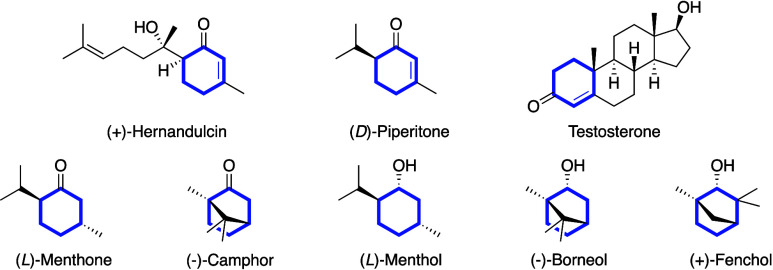
Naturally Occurring Chiral Carbocycles

Transition metal catalyzed asymmetric hydrogenation that utilizes molecular hydrogen constitutes one of the most direct strategies for the preparation of stereoenriched carbocycles. Typically low catalyst loading, high stereocontrol, and operational simplicity attract a vast amount of academic and industrial research focusing on this transformation.^2^ The majority of developed methodologies for hydrogenation focus on the conversion of a substrate into one corresponding chiral product. It would be advantageous if the reaction conditions could be easily altered to access multiple chiral building blocks out of a single substrate, each product with their own interesting features. The importance of selective hydrogenation starting from the same precursor for the chemical industry is highlighted by the production of cyclohexene and cyclohexane.^3^ In these syntheses, clean conversion into either product can be achieved, depending on the used heterogeneous catalyst and applied reaction conditions (Scheme 2a). In 2017, the Andersson group demonstrated the possibility to selectively hydrogenate cyclic 1,4-dienes into chiral equivalents by means of homogeneous N,P-iridium catalysis.^4^ High yield of the monohydrogenated or double hydrogenated product (94% and 99%, respectively) was realized by modification of the reaction time and hydrogen pressure, favoring the reduction of the more electron rich alkene (Scheme 2b). Glorius showed that substituted phenols can be hydrogenated into either cyclohexanones or diastereomerically enriched cyclohexanols depending on the nature of the heterogeneous catalysts and by proper choice of the reaction conditions (Scheme 2c).^5^

**Scheme 2:**
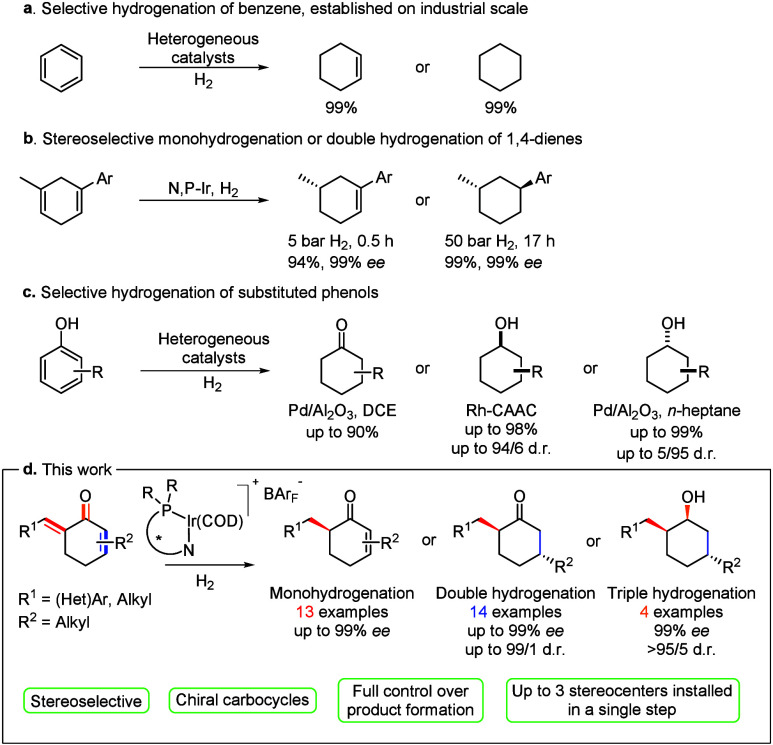
Synthesis of Different Carbocycles by Hydrogenation of the Same Precursor: (a) Benzene, (b) 1,4-Dienes, (c) Substituted Phenols, and (d) This Work, Cyclic Dienones

Recently we communicated a novel strategy to perform a regioselective monohydrogenation of cyclic dienones based on conformational restriction.^6^ It was found that conjugated enones in the (s)-*cis* conformation (*cis* conformer along the single bond in a conjugated π-system) are hydrogenated preferentially over analogues in the (s)-*trans* conformation. Later this method for a monohydrogenation of dienones was applied to the reduction of 5-alkylidene cyclopentenones by Yuan, Deng, Zhang, and co-workers.^7^ Although their work contains examples of double hydrogenation, the introduction of an ester group at the β-position was key for the stereochemical outcome. A divergent hydrogenation of a single dienone into both products (monohydrogenation and double hydrogenation) was not attainable with satisfactory stereocontrol of the pair.

In this report, we demonstrate that the two remaining π-bonds on the carbocycle can also be further reduced with excellent control over the stereochemical outcome. Using this methodology, the same dienone precursor can selectively be hydrogenated into three different carbocycles in which up to three stereogenic centers are installed in a single step (Scheme 2d). Excellent control over product formation and stereoselectivity was obtained. Therefore, this protocol expands the methods to prepare chiral carbocycles that otherwise involve multistep asymmetric syntheses. Even though rhodium-catalyzed hydrogenations of piperitone are described in the literature to afford mixed products, selective formation of either carbocycle constituted a challenge and double reduction was deemed essential to proceed via stepwise hydrogenations.^8^

In addition to the previously established regioselective monohydrogenation of dienones,^6^ some of the other and more reactive N,P-iridium catalysts were found to also catalyze the hydrogenation of the remaining *endo*-cyclic olefin under similar conditions (Table 1, see Supporting Information for an extended optimization of reaction conditions). For comparison, catalyst **A** is unreactive to the *endo*-cyclic alkene and thus exclusively yields **2a** (Entry 1). On the contrary, catalyst **B** reduced the *endo*-cyclic olefin in addition to the hydrogenation of the *exo*-cyclic olefin and produced solely the double-hydrogenated product *trans*-**3a** as a single stereoisomer (99% *ee*, 99/1 d.r., Entry 2). Interestingly, when the successful catalyst for the monohydrogenation (**A**) was used under more harsh conditions (1 mol % catalyst loading and 50 bar hydrogen pressure), still only a minor formation of **3a** was observed (5%, Entry 3). By serendipity, upon the use of catalyst **C**, a small amount of the completely saturated carbocycle, in which also the C=O double bond was reduced, was observed. With increased catalyst loading and hydrogen pressure, 88% of (1*S*,2*S*,5*R*)-**4a** was formed in excellent stereochemical purity (99% *ee*, >95/5 d.r., Entry 4). The diastereomeric ratio of **3a** dropped upon increasing formation of **4a** (from 96/4 at 8% conversion to 69/31 at 88% conversion toward **4a**, see Supporting Information). This revealed that the intermediate in the complete saturation of all π-bonds (*trans*-**3a**) was consumed with kinetic resolution since the mismatched stereoisomer (*cis*-**3a**) accounted for 4% in both mixtures and hence remained untouched. Racemization of any installed stereocenter was not observed. Thus, three different chiral carbocycles can be prepared in a divergent manner starting from the same cyclic dienone precursor simply by selecting a suitable N,P-iridium catalyst.

**Table 1:**
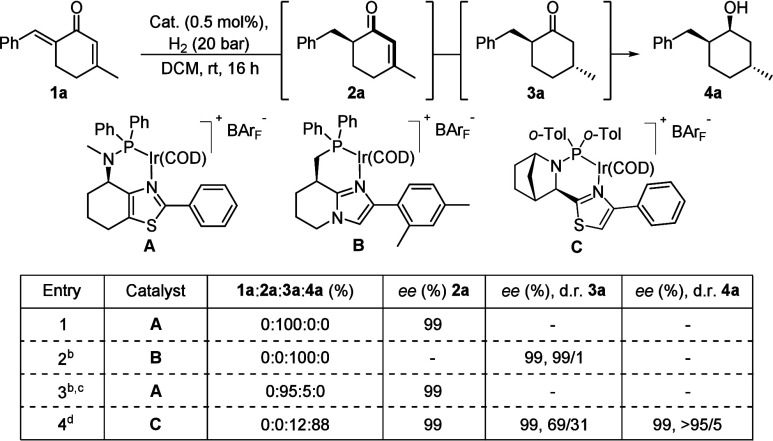
Brief Catalyst Evaluation in the Hydrogenation of Cyclic Dienones^a^

a Reaction conditions: 0.05 mmol of the substrate, 0.5 mol % catalyst, 0.5 mL of DCM, 20 bar H_2_, rt, 16 h. The product distribution was determined by ^1^H NMR spectroscopy, and the stereoselectivity was determined by GC or SFC analysis using a chiral stationary phase combined with ^1^H NMR spectroscopy for **4a**.

b 1.0 mol % catalyst.

c 50 bar H_2_.

d 4.0 mol % catalyst, 100 bar H_2_.

Given the interest in regioselective monohydrogenations of dienes,^9^ in which a synthetically useful alkene is retained in the chiral product, the substrate scope was broadened with respect to retention of the *endo*-cyclic olefin (Table 2). Substrates substituted in the *para*-position (both electron-donating and electron-withdrawing) yielded the desired products **2a**–**c** in excellent enantioselectivity (99% *ee*) and isolated yield (>96%). Substrates bearing a *meta*-tolyl, 2-naphthyl, or 3-furyl substituent were all compatible, providing **2d**–**f** in 99% *ee*. Then, the β-substituent of the *endo*-cyclic olefin was varied to ethyl (**2g**), which smoothly underwent the regioselective monohydrogenation. As anticipated, a tetrasubstituted olefin could remain untouched, and **1h** was converted into the monohydrogenated product without any over-reduction. Next, aliphatic substituted dienones were evaluated and **2i**,**j** were all found tolerant to give the corresponding monohydrogenated products in excellent enantioselectivity (99% *ee*) and isolated yield (>92%). The *i*-butyl (**12k**)- and *i*-propyl (**2l**)-substituted dienones were hydrogenated with a slight decrease in enantioselectivity to 94% *ee*. The *endo*-cyclic substituent could be elongated to ethyl to furnish **2m** in 97% *ee*.

**Table 2:**
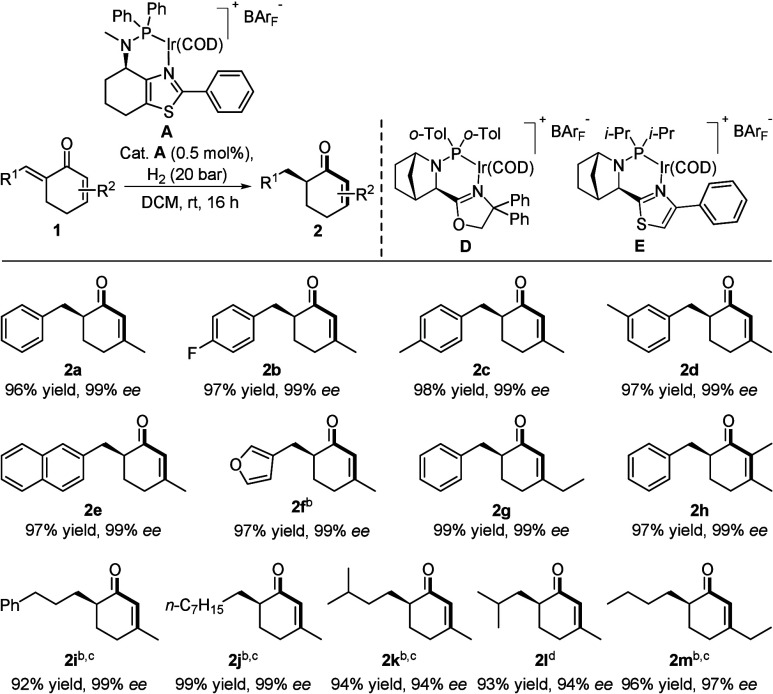
Substrate Scope for the Regioselective Monohydrogenation of Cyclic Dienones^a^

a Reaction conditions: 0.1 mmol of the substrate, 0.5 mol % catalyst, 0.5 mL of DCM, 20 bar H_2_, rt, 16 h. The enantiomeric excess was determined by SFC analysis using a chiral stationary phase. Yield refers to isolated yield.

b 1.0 mol % catalyst.

c Catalyst **D** was used, 50 bar H_2_.

d Catalyst **E** was used.

Kinetic profiling of the reaction under double-hydrogenation conditions showed that **1a** is reduced to intermediate **2a** within the first 5 min of reaction time (Scheme 3). Thereafter, a second hydrogenation gradually consumes **2a** to yield **3a**. Thus, even though catalyst **B** is able to reduce the *endo*-cyclic olefin in the locked (s)-*trans* conformation, hydrogenation of the *exo*-cyclic olefin in the (s)-*cis* conformation is still favored to a large extent.

**Scheme 3:**
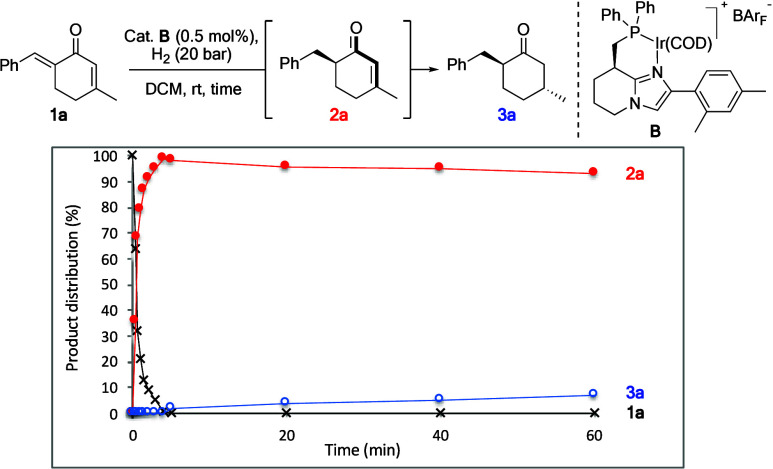
Kinetic Profiling of the Stereoselective Double Hydrogenation

The second stereogenic center introduced in this stereoselective double hydrogenation is installed in accordance with the classic nonchelated (Ir^III^/Ir^V^) hydrogenation pathway typically operative for these N,P-iridium complexes.^10^ However, hydrogenation from the same diastereotopic face is predicted regardless of whether the *endo*-cyclic alkene is hydrogenated via the classic nonchelated (Ir^III^/Ir^V^) pathway or a chelated (Ir^III^) mechanism as proposed for functionalized alkenes.^6,11–13^ The intermediate in the stereoselective double hydrogenation (*S*)-**2a** was isolated in an enantiomerically pure form and introduced to a second hydrogenation to evaluate the impact of the first installed stereogenic center on the hydrogenation of the remaining alkene (Scheme 4). Catalyst **B** (which exhibits the same sense of stereoinduction as catalyst **A**) produced *trans*-**3a** as a single stereoisomer (99/1 d.r.). In contrast, *cis*-**3a** was formed in poor quantity and diastereoselectivity (50% conv., 36/64 d.r.) when the enantiomeric form of the catalysts (*ent*-**B**) was used. Advantageously, the installation of both stereogenic centers starting from dienone **1a** advances in a stereochemical “matched” manner to furnish the thermodynamically more stable *trans*-**3a**.

**Scheme 4:**
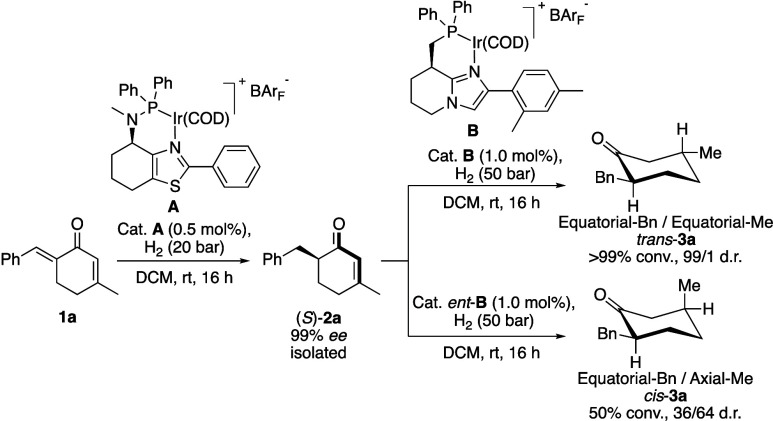
Stereoselectivity in the Double Hydrogenation

The substrate scope for the formation of *trans*-2,5-disubstituted cyclohexanones in the double hydrogenation was evaluated next (Table 3). Variation of the electronic properties of the phenyl ring on the *exo*-cyclic olefin (*para*-H, *para*-F, *para*-OMe, *para*-Me, *meta*-Me) did not deviate the stereochemical outcome, and all corresponding products were obtained in excellent stereoselectivity (**3a**–**d**,**n**, 99% *ee*, 99/1 d.r.). Naphthyl-substituted (hydrogenated on a 1 mmol scale) and heteroaromatic-containing dienones were well tolerated, and the cyclohexanone products were obtained in high stereopurity (**3e**,**f**,**o**). The size of the *endo*-cyclic substituent influenced the reactivity of the second hydrogenation, and **3g** was isolated in satisfactory yield, albeit with slightly adjusted conditions. The double hydrogenation was also extended to substrates having only aliphatic substituents, and **3i** could be obtained in 99% *ee* and 90/10 d.r. Replacement of the *endo*-cyclic methyl substituent by an ethyl group together with the variation of the *exo*-cyclic substituent to *n*-propyl, *i*-propyl, *i*-butyl, or cyclohexyl all yielded the desired *trans*-cyclohexanones in high isolated yield and stereoselectivity (**3m**–**r**, >92% yield, 98–99% *ee*, 93/7–97/3 d.r.).

**Table 3:**
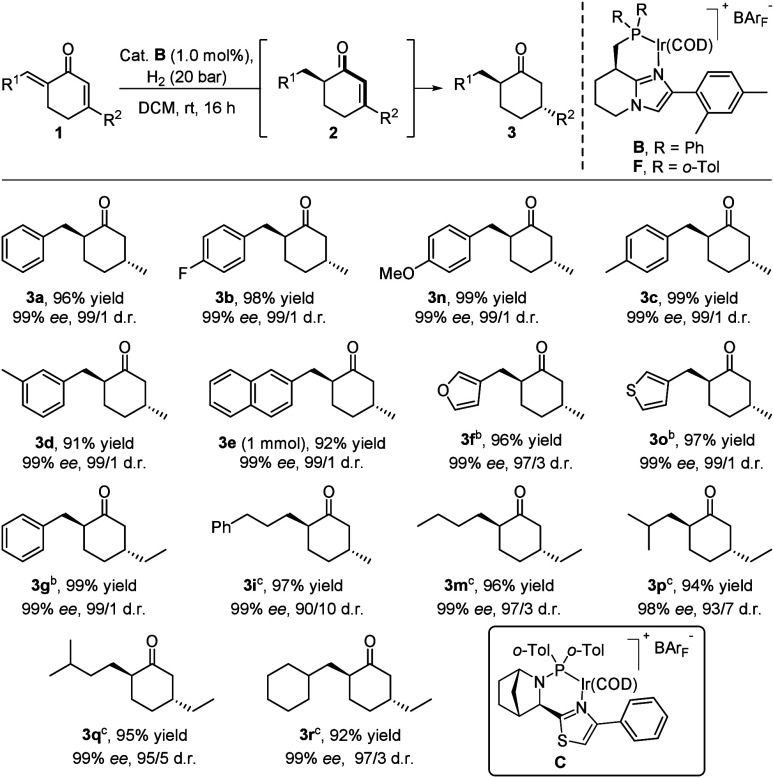
Substrate Scope for the Stereoselective Double Hydrogenation of Dienones^a^

a Reaction conditions: 0.1 mmol of the substrate, 1.0 mol % catalyst, 0.5 mL of DCM, 20 bar H_2_, rt, 16 h. The stereoselectivity was determined by SFC or GC analysis using a chiral stationary phase. Yield refers to isolated yield.

b Catalyst **F** was used.

c Catalyst **C** was used, 50 bar H_2_.

Next, the full saturation toward (1*S*,2*S*,5*R*)-cyclohexanol derivatives containing three stereogenic centers was also demonstrated on some substrates (*para*-F-, *para*-OMe-, and naphthyl-substituted, **4b**,**e**,**n**) and were hydrogenated with similar results compared to the model dienone **1a** into **4a** (Scheme 5a). The desired cyclohexanols bearing three chiral centers were isolated in excellent stereochemical purity (99% *ee*, >95/5 d.r.) and good yields (75–86%). The absolute configuration of the carbinol carbon was assigned based on ^1^H NMR spectroscopy using the Karplus relationship, which correlates the coupling constant of vicinal protons with their dihedral angle. A reduction of stereochemically pure *trans*-**3a** with NaBH_4_ produced a diastereomeric mixture of 1,2-*cis* **4a** (the formed diastereomer in the triple hydrogenation, ^*3*^ *J*_H,H_ = 2.9 Hz) and 1,2-*trans* **4a** (^*3*^ *J*_H,H_ = 4.4 Hz) in a 52/48 ratio. As a result, the final hydrogenation of the ketone preferentially took place from the least sterically hindered equatorial face of *trans*-**3a**, what led to the less thermodynamically stable 1,2-*cis* diastereomer (Scheme 5b). Large reducing agents and other metal-hydride catalysts commonly favor the reduction of cyclohexanones to take place from the equatorial face as a consequence of 1,3-diaxial interactions.^14^

**Scheme 5:**
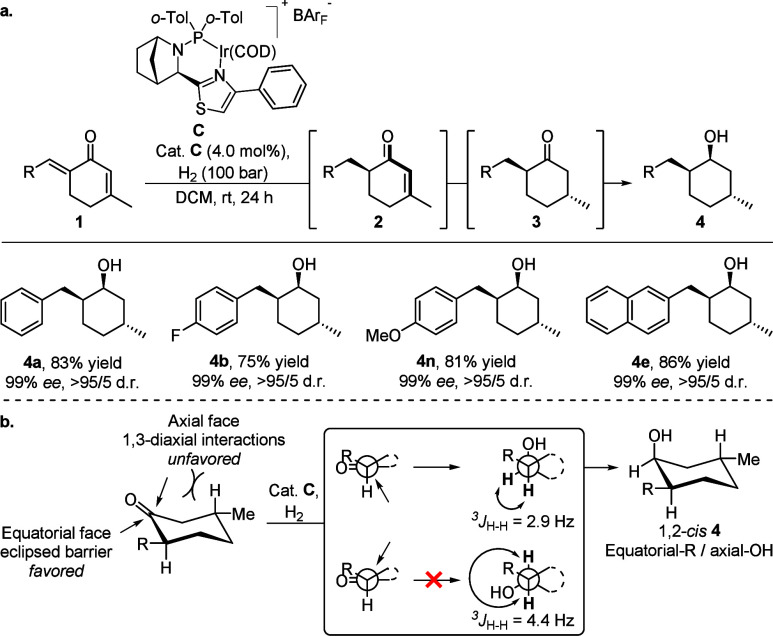
Full Saturation of Cyclic Dienones: (a) Substrate Scope for the Stereoselective Triple Hydrogenation^a^ and (b) Stereoselectivity in the Ketone Hydrogenation

a Reaction conditions: 0.1 mmol of the substrate, 4.0 mol % catalyst, 0.5 mL of DCM, 100 bar H_2_, rt, 24 h. The stereoselectivity was determined by ^1^H NMR spectroscopy combined with SFC analysis using a chiral stationary phase. Yield refers to isolated yield.

To summarize, it was demonstrated that the same dienone precursor can selectively be hydrogenated in a divergent manner into three different chiral carbocycles by a simple choice of the N,P-ligated iridium complex. The substrate scope for the regioselective monohydrogenation of this motif was expanded first to produce cyclohexenones in which a synthetically useful alkene is retained (up to 99% *ee*). In addition, the further stereoselective reduction of these dienones was demonstrated to selectively form either *trans*-2,5-disubstituted cyclohexanones (up to 99% *ee*, 99/1 d.r.) or cyclohexanols (up to 99% *ee*, 95/5 d.r.) bearing two to three stereogenic centers. This high-yielding and atom-economical protocol provides an interesting opportunity to selectively install one, two, or three stereogenic centers at carbocycles that otherwise would require multistep syntheses

## Supplementary Material



## Data Availability

The data underlying this study are available in the published article and its Supporting Information.

## Notes

Experimental procedures, optimization of the hydrogenation, characterization data of new compounds, separation of chiral products, and NMR spectra of new compounds (PDF)
