# Supplementary material for: Selective Synthesis of Chiral Carbocycles by Iridium-Catalyzed Asymmetric Mono‑, Double‑, or Triple Hydrogenation of Cyclic Dienones
Source: Org Lett. 2025 Dec 22;28(1):106–10. doi: 10.1021/acs.orglett.5c04476 (PMC12797332; doi:10.1021/acs.orglett.5c04476)
Supplement: Supplementary file 1 [file ol5c04476_si_001.pdf]

Supporting Information

Selective Synthesis of Chiral Carbocycles by Iridium-Catalyzed Asymmetric Mono-, Double- or Triple Hydrogenation of Cyclic Dienones

Bram B. C. Peters,*[1‡] Jia Zheng,*[13‡] Haili Zhang,[3] Pher G. Andersson*[12]

[1]Department of Chemistry, Stockholm University, Stockholm, 10691, Sweden.

[2]College of Chemistry and Chemical Engineering, Southwest Petroleum University, Chengdu, Sichuan, 610500, China.

[3]School of Ocean and Tropical Medicine, Guangdong Medical University, Zhanjiang, Guangdong, 524023, China.

‡These authors contributed equally to this work.

1. General information……………………………………………………………………..S2

2. Experimental detail and characterization data of new compounds…………………………S3

2.1 General procedure for the synthesis of cyclic dienones…………………………S3

2.2 General procedure for the asymmetric hydrogenation of cyclic dienones……….S4

2.3 Characterization of cyclic dienones………………………………………………S5

2.4 Characterization of hydrogenated products……………………………………..S10

3. Optimization of the asymmetric hydrogenation…………………………………………..S19

4. Assignment of the absolute configuration of the products……….………………………S20

5. References……………………………………………………………………………...S21

6. NMR spectra – dienones………………………………………………………………..S22

7. NMR spectra – hydrogenated products………………………………………………...S39

8. Separation of chiral products…………………………………………………………...S73

9. Chromatograms………………………………………………………………………...S76

1. General information

All reaction vessels were dried in a vacuum oven (160 °C) and cooled down to room temperature under a flow of nitrogen prior to use. Dichloromethane was dried over calcium hydride and freshly distilled under nitrogen. THF was distilled from sodium-benzophenone under nitrogen. The commercially available chemicals were used directly or purified by either distillation or column chromatography. Chromatographic separations were performed on Kiesel gel 60 H silica gel (particle size: 0.063-0.100 mm). Thin-layer chromatography (TLC) was performed on aluminum plates coated with Kieselgel 60 (0.20 mm, UV 254 nm) and visualized under ultraviolet light followed by staining with potassium permanganate or phosphomolybdic acid. ^{1}H NMR spectra were recorded at 400 MHz in $CDCl_3$ and referenced internally to the residual $CHCl_3$ signal (7.26 ppm). ^{13}C NMR spectra were recorded at 100 MHz in $CDCl_3$ and referenced to the central peak of $CHCl_3$ (77.16 ppm). ^{19}F NMR spectra were recorded at 377 MHz in $CDCl_3$. Chemical shifts were reported in ppm (δ scale), and coupling constants (*J*) were reported in Hertz (Hz). High resolution mass spectrometric (HRMS) data were obtained from Bruker microTOF-Q II instrument operated at ambient temperatures. Optical rotation was recorded on a thermostated polarimeter using sodium lamp (589 nm) and a 10 cm cell. Enantiomeric excesses were determined using either GC-MS (30 m column, helium gas carrier at 1 mL/min, constant pressure) or SFC-DIAD (250 mm, CO_2/MeOH) using chiral stationary phases. Racemic compounds were in all cases used for comparison.

2. Experimental details and characterization data of new compounds

2.1 General procedure for the synthesis of cyclic dienones

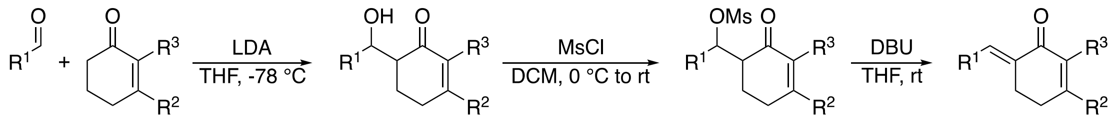

A freshly prepared solution of LDA in THF (1M, 10 mL, 10 mmol, 1.0 equiv.) was added to a stirring solution of the suitable cyclohexanone (10 mmol, 1.0 equiv.) in dry THF (50 mL) at -78 °C. The reaction mixture was stirred at -78 °C for 1 h, the suitable aldehyde (10 mmol, 1.0 equiv.) was added and the mixture was stirred at -78 °C until the substrate was completely consumed (monitored by TLC, usually 2 h). Then, the reaction mixture was quenched with saturated aqueous NH_4Cl (20 mL) and extracted with Et_2O (3 × 20 mL). The combined organic phases were washed with brine (20 mL), dried over Na_2SO_4 and evaporated *in vacuo* to dryness to give the crude. The crude product was used in the next step without purification.

MsCl (0.93 mL, 12 mmol, 1.2 equiv.) was added to a stirring solution of the obtained alcohol (10 mmol, 1.0 equiv.) in DCM (50 mL) at 0 °C and the reaction mixture was stirred at room temperature until the substrate was completely consumed (monitored by TLC, usually 3 h). Then, the reaction mixture was washed with water (20 mL), washed with brine (20 mL), dried over Na_2SO_4 and evaporated *in vacuo* to dryness to give the crude. The crude product was used in the next step without purification.

DBU (1.49 mL, 10 mmol, 1.0 equiv.) was added to the obtained mesylate (10 mmol, 1.0 equiv.), dissolved in THF (50 mL) and the mixture was stirred at room temperature until the substrate was completely consumed (monitored by TLC, usually 3 h). Then, water (30 mL) was added and the reaction mixture was extracted with Et_2O (3 × 20 mL). The combined organic phases were washed with HCl (1M, 20 mL), washed with brine (20 mL), dried over Na_2SO_4 and evaporated *in vacuo* to dryness to give the crude. The crude product was purified by flash chromatography (pentane/Et_2O, 90:10). In addition, solid products were recrystallized.

2.2 General procedure for the asymmetric hydrogenation of cyclic dienones

An oven-dried vial was charged with the dienone (0.1 mmol, 1.0 equiv.) and the in-house developed Ir-N,P-catalyst **A-F** (0.5-4.0 mol%). The catalysts were synthesized according to literature procedures; for **A**[1], **B**[2], **C**[3], **D**[4], **E**[5], **F**[6]. DCM (0.5 mL) and a magnetic stirring bar were added and the vial was placed in a high-pressure hydrogenation apparatus. The reactor was purged three times with Ar, purged three times with H_2 and then pressurized with H_2 (20-100 bar). The reaction was stirred at room temperature for 16 h before the H_2 pressure was released and the solvent removed under reduced pressure. The residue was purified by flash chromatography (pentane/Et_2O, 50/50) on silica gel to give the product. The stereoselectivity was determined by GC analysis or SFC analysis using a chiral stationary phase. The corresponding racemic product was used for comparison and it was prepared on a 0.05 mmol scale using Pd/C (or a racemic Ir-complex) as the catalyst, following the same hydrogenation procedure. The absolute configuration was determined by comparing the sign of optical rotation with reported values where applicable. In the other cases, similar hydrogenation pathways are assumed and the assignment if tentative. In the case of triple hydrogenation, the third chiral center was assigned on the basis of ^{1}H NMR coupling constants.

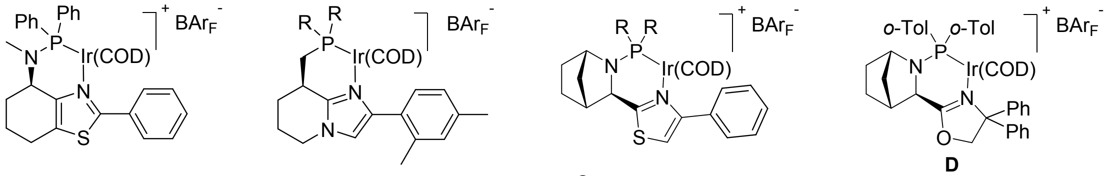

A

B, R = Ph
F, R = *o*-Tol

C, R = *o*-Tol
E, R = *i*-Pr

D

2.3 Characterization of cyclic dienones

Dienones **1n** and **1o** were prepared according to reported procedures and the spectroscopic data was in agreement with the values reported therein.[7]

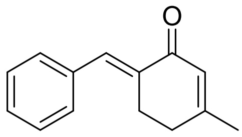

(*E*)-6-benzylidene-3-methylcyclohex-2-en-1-one (1a) White solid (1.03 g, 5.2 mmol, 52% yield over three steps), prepared according to the general procedure for the synthesis of cyclic dienones on a 10 mmol scale. The product was purified by flash chromatography (pentane/Et_2O, 90:10) on silica gel. **^{1}H NMR** (400 MHz, $CDCl_3$) δ 7.59 (s, 1H), 7.46 – 7.29 (m, 5H), 6.10 (d, *J* = 1.2 Hz, 1H), 3.00 (td, *J* = 6.4, 1.9 Hz, 2H), 2.35 (t, *J* = 6.4 Hz, 2H), 2.00 (s, 3H). **^{13}C NMR** (100 MHz, $CDCl_3$) δ 188.9, 161.5, 136.1, 134.6, 134.4, 129.9, 128.6, 128.4, 127.6, 30.8, 26.5, 24.7. **HRMS (ESI)** m/z: [M + Na]+ Calcd for $C_{14}H_{14}ONa$ 221.0937; Found 221.0938.

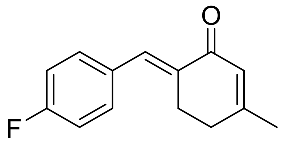

(*E*)-6-(4-fluorobenzylidene)-3-methylcyclohex-2-en-1-one (1b) Yellow solid (0.60 g, 2.8 mmol, 56% yield over three steps), prepared according to the general procedure for the synthesis of cyclic dienones on a 5 mmol scale. The product was purified by flash chromatography (pentane/Et_2O, 90:10) on silica gel. **^{1}H NMR** (400 MHz, $CDCl_3$) δ 7.54 (s, 1H), 7.34 (dd, *J* = 8.5, 5.5 Hz, 2H), 7.08 (dd, *J* = 12.0, 5.2 Hz, 2H), 6.09 (d, *J* = 1.0 Hz, 1H), 2.96 (td, *J* = 6.4, 1.8 Hz, 2H), 2.35 (t, *J* = 6.4 Hz, 2H), 2.00 (s, 3H). **^{13}C NMR** (100 MHz, $CDCl_3$) δ 188.7, 162.6 (d, $^1J_{C-F}$ = 247.4 Hz), 161.5, 134.2 (d, $^5J_{C-F}$ = 1.2 Hz), 133.4, 132.2 (d, $^4J_{C-F}$ = 3.4 Hz), 131.7 (d, $^3J_{C-F}$ = 8.2 Hz), 127.6, 115.6 (d, $^2J_{C-F}$ = 21.6 Hz, 1H) 30.7, 26.4, 24.7. **^{19}F NMR** (377 MHz, $CDCl_3$) δ -112.5. **HRMS (ESI)** m/z: [M + Na]+ Calcd for $C_{14}H_{13}FONa$ 239.0844; Found 239.0843.

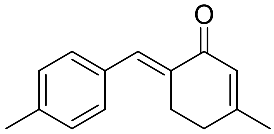

(*E*)-3-methyl-6-(4-methylbenzylidene)cyclohex-2-en-1-one (1c) White solid (0.50 g, 2.4 mmol, 47% yield over three steps), prepared according to the general procedure for the synthesis of cyclic dienones on a 5 mmol scale. The product was purified by flash chromatography (pentane/Et_2O, 90:10) on silica gel. **^{1}H NMR** (400 MHz, $CDCl_3$) δ 7.57 (s, 1H), 7.32 – 7.24 (m, 2H), 7.20 (d, *J* = 8.0 Hz, 2H), 6.09 (dd, *J* = 2.6, 1.3 Hz, 1H), 3.00 (td, *J* = 6.5, 1.9 Hz, 2H), 2.40 – 2.29 (m, 5H), 2.00 (d, *J* = 1.1 Hz, 3H). **^{13}C NMR** (100 MHz, $CDCl_3$) δ 189.0, 161.3, 138.6, 134.7, 133.7, 133.3, 123.0, 129.3, 127.7, 30.8, 26.5, 24.7, 21.6. **HRMS (ESI)** m/z: [M + Na]+ Calcd for $C_{15}H_{16}ONa$ 235.1093; Found 235.1097.

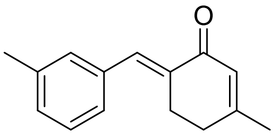

(*E*)-3-methyl-6-(3-methylbenzylidene)cyclohex-2-en-1-one (1d) White solid (0.64 g, 3.0 mmol, 60% yield over three steps), prepared according to the general procedure for the synthesis of cyclic dienones on a 5 mmol scale. The product was purified by flash chromatography (pentane/Et_2O, 90:10) on silica gel. **^{1}H NMR** (400 MHz, $CDCl_3$) δ 7.57 (s, 1H), 7.33 – 7.23 (m, 1H), 7.15 (dd, *J* = 13.6, 7.0 Hz, 3H), 6.09 (dd, *J* = 2.6, 1.3 Hz, 1H), 3.00 (td, *J* = 6.4, 1.9 Hz, 2H), 2.40 – 2.28 (m, 5H), 2.00 (d, *J* = 1.0 Hz, 3H). **^{13}C NMR** (100 MHz, $CDCl_3$) δ 188.8, 161.3, 138.0, 135.9, 134.5, 134.0, 130.4, 129.0, 128.2, 127.5, 126.7, 30.6, 26.3, 24.5, 21.5. **HRMS (ESI)** m/z: [M + Na]+ Calcd for $C_{15}H_{16}ONa$ 235.1093; Found 235.1087.

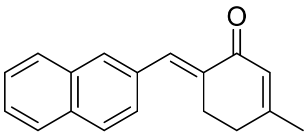

(*E*)-3-methyl-6-(naphthalen-2-ylmethylene)cyclohex-2-en-1-one (1e) White solid (1.02 g, 4.1 mmol, 41% yield over three steps), prepared according to the general procedure for the synthesis of cyclic dienones on a 10 mmol scale. The product was purified by flash chromatography (pentane/Et_2O, 90:10) on silica gel. **^{1}H NMR** (400 MHz, $CDCl_3$) δ 7.90 – 7.79 (m, 4H), 7.75 (s, 1H), 7.55 – 7.45 (m, 3H), 6.13 (d, *J* = 1.3 Hz, 1H), 3.10 (td, *J* = 6.4, 1.9 Hz, 2H), 2.37 (t, *J* = 6.4 Hz, 2H), 2.02 (s, 3H). **^{13}C NMR** (100 MHz, $CDCl_3$) δ 188.9, 161.5, 134.7, 133.7, 133.3, 133.1, 129.4, 128.4, 128.2, 127.9, 127.7, 127.5, 126.8, 126.6, 30.8, 26.6, 24.7. **HRMS (ESI)** m/z: [M + Na]+ Calcd for $C_{18}H_{16}ONa$ 271.1093; Found 271.1090.

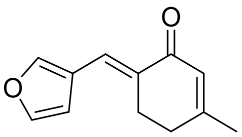

(*E*)-6-(furan-3-ylmethylene)-3-methylcyclohex-2-en-1-one (1f) White solid (0.47 g, 2.5 mmol, 50% yield over three steps), prepared according to the general procedure for the synthesis of cyclic dienones on a 5 mmol scale. The product was purified by flash chromatography (pentane/Et_2O, 90:10) on silica gel. **^{1}H NMR** (400 MHz, $CDCl_3$) δ 7.64 (s, 1H), 7.47 – 7.43 (m, 1H), 7.41 (s, 1H), 6.58 (d, *J* = 1.6 Hz, 1H), 6.07 (dd, *J* = 2.7, 1.3 Hz, 1H), 2.95 (td, *J* = 6.6, 1.9 Hz, 2H), 2.40 (t, *J* = 6.5 Hz, 2H), 2.00 (d, *J* = 1.2 Hz, 3H). **^{13}C NMR** (100 MHz, $CDCl_3$) δ 188.1, 161.2, 144.4, 143.7, 132.6, 127.6, 124.9, 122.0, 111.1, 30.2, 26.4, 24.7. **HRMS (ESI)** m/z: [M + Na]+ Calcd for $C_{12}H_{12}O_2Na$ 211.0730; Found 211.0730.

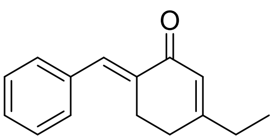

(*E*)-6-benzylidene-3-ethylcyclohex-2-en-1-one (1g) Yellow solid (0.63 g, 3.0 mmol, 59% yield over three steps), prepared according to the general procedure for the synthesis of cyclic dienones on a 5 mmol scale. The product was purified by flash chromatography (pentane/Et_2O, 90:10) on silica gel. **^{1}H NMR** (400 MHz, $CDCl_3$) δ 7.60 (s, 1H), 7.42 – 7.29 (m, 5H), 6.14 – 6.07 (m, 1H), 3.00 (td, *J* = 6.4, 1.9 Hz, 2H), 2.35 (t, *J* = 6.4 Hz, 2H), 2.28 (q, *J* = 7.4 Hz, 2H), 1.13 (t, *J* = 7.4 Hz, 3H). **^{13}C NMR** (100 MHz, $CDCl_3$) δ 189.2, 166.6, 136.1, 134.8, 134.5, 129.9, 128.6, 128.4, 125.5, 31.1, 29.6, 26.6, 11.4. **HRMS (ESI)** m/z: [M + Na]+ Calcd for $C_{15}H_{16}ONa$ 235.1093; Found 235.1091.

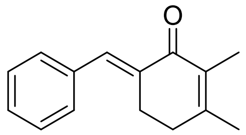

(*E*)-6-benzylidene-2,3-dimethylcyclohex-2-en-1-one (1h) White solid (0.72 g, 3.4 mmol, 68% yield over three steps), prepared according to the general procedure for the synthesis of cyclic dienones on a 5 mmol scale. The product was purified by flash chromatography (pentane/Et_2O, 90:10) on silica gel. **^{1}H NMR** (400 MHz, $CDCl_3$) δ 7.59 (d, *J* = 1.6 Hz, 1H), 7.43 – 7.27 (m, 5H), 2.94 (td, *J* = 6.4, 1.9 Hz, 2H), 2.37 (t, *J* = 6.1 Hz, 2H), 1.98 (d, *J* = 0.7 Hz, 3H), 1.92 – 1.87 (m, 3H). **^{13}C NMR** (100 MHz, $CDCl_3$) δ 188.9, 154.2, 136.4, 135.2, 134.1, 132.2, 129.8, 128.5, 128.2, 32.0, 26.4, 21.9, 11.9. **HRMS (ESI)** m/z: [M + Na]+ Calcd for $C_{15}H_{16}ONa$ 235.1093; Found 235.1092.

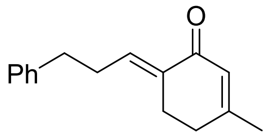

(*E*)-3-methyl-6-(3-phenylpropylidene)cyclohex-2-en-1-one (1i) White solid (0.55 g, 2.5 mmol, 49% yield over three steps), prepared according to the general procedure for the synthesis of cyclic dienones on a 5 mmol scale. The product was purified by flash chromatography (pentane/Et_2O, 90:10) on silica gel. **^{1}H NMR** (400 MHz, $CDCl_3$) δ 7.30 – 7.25 (m, 2H), 7.22 – 7.14 (m, 3H), 6.68 (tt, *J* = 7.5, 1.6 Hz, 1H), 5.97 (dd, *J* = 2.7, 1.3 Hz, 1H), 2.76 (t, *J* = 7.6 Hz, 2H), 2.56 – 2.45 (m, 4H), 2.17 (t, *J* = 6.4 Hz, 2H), 1.95 (d, *J* = 1.0 Hz, 3H). **^{13}C NMR** (100 MHz, $CDCl_3$) δ 188.7, 161.8, 141.5, 135.9, 134.4, 128.7, 138.6, 127.6, 126.2, 35.3, 30.9, 30.0, 25.0, 24.7. **HRMS (ESI)** m/z: [M + Na]+ Calcd for $C_{16}H_{18}ONa$ 249.1250; Found 249.1269.

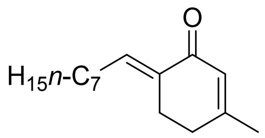

(*E*)-3-methyl-6-octylidenecyclohex-2-en-1-one (1j) Colorless oil (0.73 g, 3.3 mmol, 66% yield over three steps), prepared according to the general procedure for the synthesis of cyclic dienones on a 5 mmol scale. The product was purified by flash chromatography (pentane/Et_2O, 90:10) on silica gel. **^{1}H NMR** (400 MHz, $CDCl_3$) δ 6.64 (dd, *J* = 8.2, 7.0 Hz, 1H), 6.04 – 5.92 (m, 1H), 2.65 (t, *J* = 6.4 Hz, 2H), 2.32 (t, *J* = 6.4 Hz, 2H), 2.15 (q, *J* = 7.4 Hz, 2H), 1.97 (d, *J* = 0.7 Hz, 3H), 1.43 (dd, *J* = 13.8, 6.7 Hz, 2H), 1.28 (s, 8H), 0.87 (t, *J* = 6.3 Hz, 3H). **^{13}C NMR** (100 MHz, $CDCl_3$) δ 188.8, 161.6, 137.7, 133.5, 127.7, 32.0, 31.0, 29.5, 29.3, 29.1, 28.0, 25.0, 24.7, 22.9, 14.3. **HRMS (ESI)** m/z: [M + Na]+ Calcd for $C_{15}H_{24}ONa$ 243.1719; Found 243.1719.

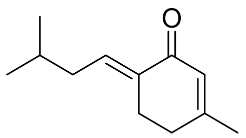

(*E*)-3-methyl-6-(3-methylbutylidene)cyclohex-2-en-1-one (1k) Colorless oil (0.33 g, 1.9 mmol, 37% yield over three steps), prepared according to the general procedure for the synthesis of cyclic dienones on a 5 mmol scale. The product was purified by flash chromatography (pentane/Et_2O, 90:10) on silica gel. **^{1}H NMR** (400 MHz, $CDCl_3$) δ 6.76 – 6.56 (m, 1H), 6.07 – 5.94 (m, 1H), 2.64 (t, *J* = 6.1 Hz, 2H), 2.32 (t, *J* = 6.2 Hz, 2H), 2.05 (td, *J* = 7.8, 2.2 Hz, 2H), 1.96 (t, *J* = 6.7 Hz, 3H), 1.75 (ttd, *J* = 13.3, 6.7, 2.3 Hz, 1H), 0.92 (dd, *J* = 6.6, 2.2 Hz, 6H). **^{13}C NMR** (100 MHz, $CDCl_3$) δ 188.7, 161.6, 136.4, 134.2, 127.6, 37.1, 31.0, 28.6, 25.2, 24.7, 22.7. **HRMS (ESI)** m/z: [M + Na]+ Calcd for $C_{12}H_{18}ONa$ 201.1250; Found 201.1252.

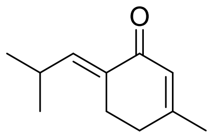
(E)-3-methyl-6-(2-methylpropylidene)cyclohex-2-en-1-one (1l) Colorless oil (0.48 g, 2.9 mmol, 58% yield over three steps), prepared according to the general procedure for the synthesis of cyclic dienones on a 5 mmol scale. The product was purified by flash chromatography (pentane/Et_2O, 90:10) on silica gel. **^{1}H NMR** (400 MHz, $CDCl_3$) δ 6.46 (dd, J = 9.8, 1.3 Hz, 1H), 6.07 – 5.94 (m, 1H), 2.75 – 2.54 (m, 3H), 2.33 (t, J = 6.4 Hz, 2H), 1.97 (d, J = 0.6 Hz, 3H), 1.02 (dd, J = 6.7, 0.8 Hz, 6H). **^{13}C NMR** (100 MHz, $CDCl_3$) δ 189.1, 161.3, 144.1, 131.6, 127.7, 31.1, 27.2, 25.1, 24.7, 22.6. **HRMS (ESI)** m/z: [M + Na]+ Calcd for $C_{11}H_{16}ONa$ 187.1093; Found 187.1091.

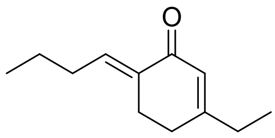
(E)-6-butylidene-3-ethylcyclohex-2-en-1-one (1m) Colorless oil (0.56 g, 3.2 mmol, 63% yield over three steps), prepared according to the general procedure for the synthesis of cyclic dienones on a 5 mmol scale. The product was purified by flash chromatography (pentane/Et_2O, 90:10) on silica gel. **^{1}H NMR** (400 MHz, $CDCl_3$) δ 6.65 (ddd, J = 7.6, 4.7, 1.7 Hz, 1H), 6.03 – 5.95 (m, 1H), 2.70 – 2.61 (m, 2H), 2.33 (t, J = 6.4 Hz, 2H), 2.25 (q, J = 7.3 Hz, 2H), 2.14 (q, J = 7.5 Hz, 2H), 1.56 – 1.41 (m, 2H), 1.10 (t, J = 7.4 Hz, 3H), 0.93 (t, J = 7.4 Hz, 3H). **^{13}C NMR** (100 MHz, $CDCl_3$) δ 189.1, 166.7, 137.4, 134.2, 125.5, 31.1, 30.0, 29.8, 25.2, 22.3, 14.1, 11.5. **HRMS (ESI)** m/z: [M + Na]+ Calcd for $C_{12}H_{18}ONa$ 201.1250; Found 201.1253.

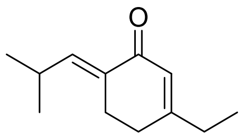
(E)-3-ethyl-6-(2-methylpropylidene)cyclohex-2-en-1-one (1p) Colorless oil (0.62 g, 3.5 mmol, 70% yield over three steps), prepared according to the general procedure for the synthesis of cyclic dienones on a 5 mmol scale. The product was purified by flash chromatography (pentane/Et_2O, 90:10) on silica gel. **^{1}H NMR** (400 MHz, $CDCl_3$) δ 6.47 (d, J = 9.8 Hz, 1H), 6.02 – 5.97 (m, 1H), 2.70 – 2.54 (m, 3H), 2.33 (t, J = 6.4 Hz, 2H), 2.25 (q, J = 7.4 Hz, 2H), 1.10 (t, J = 7.4 Hz, 3H), 1.02 (d, J = 6.7 Hz, 6H). **^{13}C NMR** (100 MHz, $CDCl_3$) δ 189.4, 166.7, 144.0, 132.0, 125.6, 31.1, 29.9, 27.3, 25.2, 22.6, 11.4. **HRMS (ESI)** m/z: [M + Na]+ Calcd for $C_{12}H_{18}ONa$ 201.1250; Found 201.1254.

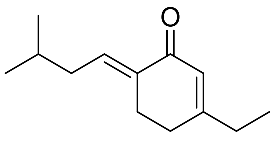
(E)-3-ethyl-6-(3-methylbutylidene)cyclohex-2-en-1-one (1q) Colorless oil (0.46 g, 2.4 mmol, 48% yield over three steps), prepared according to the general procedure for the synthesis of cyclic dienones on a 5 mmol scale. The product was purified by flash chromatography (pentane/Et_2O, 90:10) on silica gel. **^{1}H NMR** (400 MHz, $CDCl_3$) δ 6.73 – 6.63 (m, 1H), 6.02 – 5.97 (m, 1H), 2.69 – 2.62 (m, 2H), 2.32 (t, J = 6.4 Hz, 2H), 2.25 (q, J = 7.4 Hz, 2H), 2.05 (dd, J = 7.7, 6.9 Hz, 2H), 1.75 (dp, J = 13.4, 6.7 Hz, 1H), 1.10 (t, J = 7.4 Hz, 3H), 0.92 (d, J = 6.7 Hz, 6H). **^{13}C NMR** (100 MHz, $CDCl_3$) δ 189.0, 166.7, 136.4, 134.6, 125.5, 37.1, 31.1, 29.8, 28.6, 25.3, 22.7, 11.5. **HRMS (ESI)** m/z: [M + Na]+ Calcd for $C_{13}H_{20}ONa$ 215.1406; Found 215.1404.

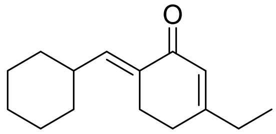 **(*E*)-6-(cyclohexylmethylene)-3-ethylcyclohex-2-en-1-one (1r)** White solid (0.26 g, 1.2 mmol, 60% yield over three steps), prepared according to the general procedure for the synthesis of cyclic dienones on a 2 mmol scale. The product was purified by flash chromatography (pentane/Et_2O, 90:10) on silica gel. **^{1}H NMR** (400 MHz, $CDCl_3$) δ 6.49 (dt, *J* = 9.6, 1.5 Hz, 1H), 6.05 – 5.93 (m, 1H), 2.66 (td, *J* = 6.4, 1.7 Hz, 2H), 2.29 (ddd, *J* = 21.7, 13.7, 6.9 Hz, 5H), 1.81 – 1.71 (m, 2H), 1.63 (m, 3H), 1.35 – 1.13 (m, 5H), 1.10 (t, *J* = 7.4 Hz, 3H). **^{13}C NMR** (100 MHz, $CDCl_3$) δ 189.5, 166.7, 142.6, 132.4, 125.7, 37.1, 32.6, 31.1, 30.0, 26.1, 25.9, 25.4, 11.5. **HRMS (ESI)** m/z: [M + Na]+ Calcd for $C_{15}H_{22}ONa$ 241.1563; Found 241.1561.

2.4 Characterization of hydrogenated products

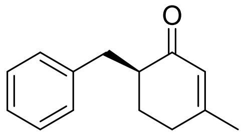

(*S*)-6-benzyl-3-methylcyclohex-2-en-1-one (2a) Colorless oil (19.2 mg, 96% yield, 99% *ee*), prepared according to the general procedure for the asymmetric hydrogenation on a 0.1 mmol scale using catalyst **A**. The product was purified by flash chromatography (pentane/Et_2O, 50:50) on silica gel. **^{1}H NMR** (400 MHz, $CDCl_3$) δ 7.31 – 7.26 (m, 2H), 7.20 (ddd, *J* = 8.3, 6.1, 3.0 Hz, 3H), 5.90 (dd, *J* = 2.8, 1.4 Hz, 1H), 3.42 – 3.31 (m, 1H), 2.55 – 2.40 (m, 2H), 2.30 – 2.23 (m, 2H), 1.99 – 1.90 (m, 4H), 1.68 – 1.57 (m, 1H). **^{13}C NMR** (100 MHz, $CDCl_3$) δ 200.7, 161.9, 140.4, 129.4, 128.6, 126.5, 126.3, 47.5, 35.6, 30.6, 27.1, 24.4. **HRMS (ESI)** m/z: [M + Na]+ Calcd for $C_{14}H_{16}ONa$ 223.1093; Found 223.1111. **$[\alpha]_D^{26}$** = +37.0 (c = 0.1, $CHCl_3$).

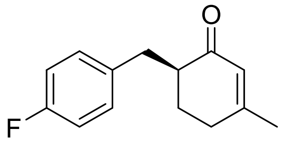

(*S*)-6-(4-fluorobenzyl)-3-methylcyclohex-2-en-1-one (2b) Colorless oil (21.1 mg, 97% yield, 99% *ee*), prepared according to the general procedure for the asymmetric hydrogenation on a 0.1 mmol scale using catalyst **A**. The product was purified by flash chromatography (pentane/Et_2O, 50:50) on silica gel. **^{1}H NMR** (400 MHz, $CDCl_3$) δ 7.16 – 7.09 (m, 2H), 7.01 – 6.91 (m, 2H), 5.89 (dd, *J* = 2.5, 1.2 Hz, 1H), 3.28 (dd, *J* = 13.8, 4.0 Hz, 1H), 2.51 (dd, *J* = 13.8, 9.5 Hz, 1H), 2.46 – 2.36 (m, 1H), 2.26 (dd, *J* = 6.4, 5.5 Hz, 2H), 1.98 – 1.87 (m, 4H), 1.60 (ddt, *J* = 13.3, 11.4, 7.6 Hz, 1H). **^{13}C NMR** (100 MHz, $CDCl_3$) δ 200.5, 162.0, 161.6 (d, $^1J_{C-F}$ = 242.2 Hz), 135.9 (d, $^4J_{C-F}$ = 3.3 Hz), 130.8 (d, $^3J_{C-F}$ = 7.7 Hz), 126.5, 115.3 (d, $^2J_{C-F}$ = 21.1 Hz), 47.5, 34.8, 30.7, 27.2, 24.4. **^{19}F NMR** (377 MHz, $CDCl_3$) δ -117.4. **HRMS (ESI)** m/z: [M + Na]+ Calcd for $C_{14}H_{15}FONa$ 241.0999; Found 241.1009. **$[\alpha]_D^{26}$** = +41.0 (c = 0.1, $CHCl_3$).

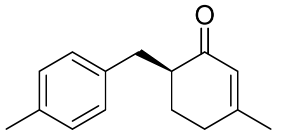

(*S*)-3-methyl-6-(4-methylbenzyl)cyclohex-2-en-1-one (2c) Colorless oil (21.0 mg, 98% yield, 99% *ee*), prepared according to the general procedure for the asymmetric hydrogenation on a 0.1 mmol scale using catalyst **A**. The product was purified by flash chromatography (pentane/Et_2O, 50:50) on silica gel. **^{1}H NMR** (400 MHz, $CDCl_3$) δ 7.15 – 6.99 (m, 4H), 5.89 (dd, *J* = 2.8, 1.4 Hz, 1H), 3.39 – 3.23 (m, 1H), 2.51 – 2.36 (m, 2H), 2.33 (d, *J* = 7.4 Hz, 3H), 2.24 (dd, *J* = 9.3, 4.5 Hz, 2H), 2.02 – 1.88 (m, 4H), 1.66 – 1.54 (m, 1H). **^{13}C NMR** (100 MHz, $CDCl_3$) δ 200.9, 161.9, 137.2, 135.7, 129.3, 129.2, 126.5, 47.6, 35.1, 30.6, 27.1, 24.4, 21.2. **HRMS (ESI)** m/z: [M + Na]+ Calcd for $C_{15}H_{18}ONa$ 237.1250; Found 237.1252. **$[\alpha]_D^{26}$** = +26.0 (c = 0.1, $CHCl_3$).

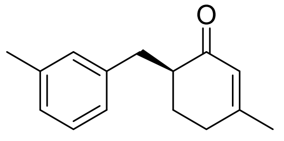

(*S*)-3-methyl-6-(3-methylbenzyl)cyclohex-2-en-1-one (2d) Colorless oil (21.2 mg, 98% purity, 97% yield, 99% *ee*), prepared according to the general procedure for the asymmetric hydrogenation on a 0.1 mmol scale using catalyst **A**. The product was purified by flash

chromatography (pentane/Et_2O, 50:50) on silica gel. **^{1}H NMR** (400 MHz, $CDCl_3$) δ 7.17 (t, *J* = 7.4 Hz, 1H), 7.00 (dd, *J* = 14.3, 9.3 Hz, 3H), 5.90 (dd, *J* = 2.8, 1.4 Hz, 1H), 3.40 – 3.26 (m, 1H), 2.51 – 2.38 (m, 2H), 2.33 (s, 3H), 2.29 – 2.22 (m, 2H), 2.01 – 1.90 (m, 4H), 1.69 – 1.56 (m, 1H). **^{13}C NMR** (100 MHz, $CDCl_3$) δ 200.8, 161.9, 140.3, 138.1, 130.2, 128.4, 127.0, 126.5, 126.4, 47.5, 35.5, 30.6, 27.1, 24.4, 21.6. **HRMS (ESI)** m/z: [M + Na]+ Calcd for $C_{15}H_{18}ONa$ 237.1250; Found 237.1259. **$[\alpha]_D^{26}$** = +35.0 (c = 0.1, $CHCl_3$).

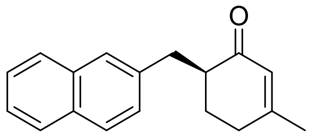

(*S*)-3-methyl-6-(naphthalen-2-ylmethyl)cyclohex-2-en-1-one (2e) Colorless oil (24.3 mg, 97% yield, 99% *ee*), prepared according to the general procedure for the asymmetric hydrogenation on a 0.1 mmol scale using catalyst **A**. The product was purified by flash chromatography (pentane/Et_2O, 50:50) on silica gel. **^{1}H NMR** (400 MHz, $CDCl_3$) δ 7.85 – 7.73 (m, 3H), 7.63 (s, 1H), 7.53 – 7.38 (m, 2H), 7.33 (dd, *J* = 8.4, 1.7 Hz, 1H), 5.93 (dd, *J* = 2.7, 1.3 Hz, 1H), 3.53 (dd, *J* = 13.7, 3.8 Hz, 1H), 2.67 (dd, *J* = 13.7, 9.9 Hz, 1H), 2.61 – 2.49 (m, 1H), 2.24 (dd, *J* = 9.1, 4.0 Hz, 2H), 2.05 – 1.88 (m, 4H), 1.65 (dddd, *J* = 13.4, 11.3, 8.5, 6.5 Hz, 1H). **^{13}C NMR** (100 MHz, $CDCl_3$) δ 200.6, 162.0, 137.9, 133.7, 132.3, 128.2, 127.8, 127.8, 127.8, 127.6, 126.5, 126.2, 125.5, 47.5, 35.7, 30.7, 27.2, 24.4 **HRMS (ESI)** m/z: [M + Na]+ Calcd for $C_{18}H_{18}ONa$ 273.1250; Found 273.1248. **$[\alpha]_D^{26}$** = +42.0 (c = 0.1, $CHCl_3$).

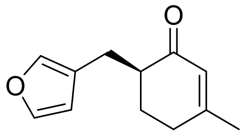

(*S*)-6-(furan-3-ylmethyl)-3-methylcyclohex-2-en-1-one (2f) Colorless oil (18.4mg, 97% yield, 99% *ee*), prepared according to the general procedure for the asymmetric hydrogenation on a 0.1 mmol scale using catalyst **A**. The product was purified by flash chromatography (pentane/Et_2O, 50:50) on silica gel. **^{1}H NMR** (400 MHz, $CDCl_3$) δ 7.34 (t, *J* = 1.7 Hz, 1H), 7.23 (dd, *J* = 1.5, 0.7 Hz, 1H), 6.28 – 6.22 (m, 1H), 5.88 (dt, *J* = 2.3, 1.2 Hz, 1H), 2.98 (dd, *J* = 14.6, 4.1 Hz, 1H), 2.51 (dd, *J* = 14.6, 8.7 Hz, 1H), 2.37 (ddt, *J* = 11.8, 8.8, 4.4 Hz, 1H), 2.32 – 2.24 (m, 2H), 2.01 (ddd, *J* = 13.5, 8.9, 4.7 Hz, 1H), 1.93 (s, 3H), 1.65 (dddd, *J* = 13.3, 11.7, 9.7, 5.7 Hz, 1H). **^{13}C NMR** (100 MHz, $CDCl_3$) δ 200.6, 162.0, 143.0, 140.2, 126.6, 122.6, 111.7, 46.3, 30.7, 27.4, 24.6, 24.4. **HRMS (ESI)** m/z: [M + Na]+ Calcd for $C_{12}H_{14}O_2Na$ 213.0086; Found 213.0085. **$[\alpha]_D^{26}$** = +50.0 (c = 0.1, $CHCl_3$).

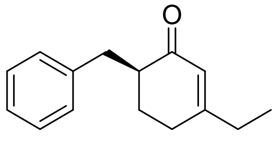

(*S*)-6-benzyl-3-ethylcyclohex-2-en-1-one (2g) Colorless oil (21.2 mg, 99% yield, 99% *ee*), prepared according to the general procedure for the asymmetric hydrogenation on a 0.1 mmol scale using catalyst **A**. The product was purified by flash chromatography (pentane/Et_2O, 50:50) on silica gel. **^{1}H NMR** (400 MHz, $CDCl_3$) δ 7.31 – 7.25 (m, 2H), 7.19 (ddd, *J* = 6.8, 6.0, 2.2 Hz, 3H), 5.93 – 5.88 (m, 1H), 3.42 – 3.32 (m, 1H), 2.56 – 2.42 (m, 2H), 2.31 – 2.17 (m, 4H), 1.95 (dq, *J* = 13.3, 4.5 Hz, 1H), 1.67 – 1.55 (m, 1H), 1.09 (t, *J* = 7.4 Hz, 3H). **^{13}C NMR** (100 MHz, $CDCl_3$) δ 201.0, 167.1, 140.4, 129.4, 128.5, 126.2, 124.4, 47.9, 35.6, 30.8, 29.3, 27.3, 11.5. **HRMS (ESI)** m/z: [M + Na]+ Calcd for $C_{15}H_{18}ONa$ 237.1250; Found 237.1249. **$[\alpha]_D^{26}$** = +32.0 (c = 0.1, $CHCl_3$).

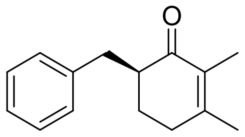

(*S*)-6-benzyl-2,3-dimethylcyclohex-2-en-1-one (2h) Colorless oil (20.8 mg, 97% yield, 99% *ee*), prepared according to the general procedure for the asymmetric hydrogenation on a 0.1 mmol scale using catalyst **A**. The product was purified by flash chromatography (pentane/Et_2O, 50:50) on silica gel. **^{1}H NMR** (400 MHz, $CDCl_3$) δ 7.28 (t, J = 7.3 Hz, 2H), 7.23 – 7.15 (m, 3H), 3.44 – 3.28 (m, 1H), 2.53 – 2.44 (m, 2H), 2.33 – 2.25 (m, 2H), 1.90 (t, J = 3.2 Hz, 3H), 1.87 (dd, J = 8.8, 4.4 Hz, 1H), 1.79 (d, J = 0.8 Hz, 3H), 1.64 – 1.52 (m, 1H). **^{13}C NMR** (100 MHz, $CDCl_3$) δ 200.3, 154.2, 140.6, 130.8, 129.4, 128.5, 126.1, 47.7, 36.1, 32.0, 26.8, 21.6, 11.4. **HRMS (ESI)** m/z: [M + Na]+ Calcd for $C_{15}H_{18}ONa$ 237.1250; Found 237.1250. **$[\alpha]_D^{26}$** = +49.0 (c = 0.1, $CHCl_3$).

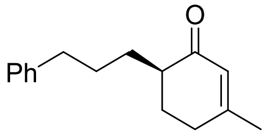

(*R*)-3-methyl-6-(3-phenylpropyl)cyclohex-2-en-1-one (2i) Colorless oil (19.6 mg, 97% purity, 92% yield, 99% *ee*), prepared according to the general procedure for the asymmetric hydrogenation on a 0.1 mmol scale using catalyst **D**. The product was purified by flash chromatography (pentane/Et_2O, 50:50) on silica gel. **^{1}H NMR** (400 MHz, $CDCl_3$) δ 7.30 – 7.24 (m, 2H), 7.21 – 7.14 (m, 3H), 5.83 (dd, J = 2.8, 1.4 Hz, 1H), 2.70 – 2.57 (m, 2H), 2.28 (t, J = 5.9 Hz, 2H), 2.20 (ddt, J = 10.2, 7.8, 5.0 Hz, 1H), 2.07 (dq, J = 13.3, 5.0 Hz, 1H), 1.92 (t, J = 2.6 Hz, 3H), 1.88 (ddd, J = 13.4, 8.4, 4.0 Hz, 1H), 1.78 – 1.61 (m, 3H), 1.46 – 1.37 (m, 1H). **^{13}C NMR** (100 MHz, $CDCl_3$) δ 201.8, 161.4, 142.6, 128.6, 128.5, 126.5, 125.9, 45.5, 36.3, 30.4, 29.2, 29.2, 27.8, 24.3. **HRMS (ESI)** m/z: [M + Na]+ Calcd for $C_{16}H_{20}ONa$ 229.0658; Found 229.0673. **$[\alpha]_D^{26}$** = +34.0 (c = 0.1, $CHCl_3$).

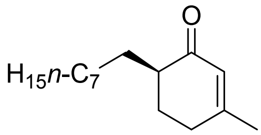

(*R*)-3-methyl-6-octylcyclohex-2-en-1-one (2j) Colorless oil (22.0 mg, 99% yield, 99% *ee*), prepared according to the general procedure for the asymmetric hydrogenation on a 0.1 mmol scale using catalyst **D**. The product was purified by flash chromatography (pentane/Et_2O, 50:50) on silica gel. **^{1}H NMR** (400 MHz, $CDCl_3$) δ 5.83 (dd, J = 2.7, 1.4 Hz, 1H), 2.29 (t, J = 5.8 Hz, 2H), 2.24 – 2.13 (m, 1H), 2.13 – 2.04 (m, 1H), 1.93 (d, J = 0.5 Hz, 3H), 1.87 – 1.65 (m, 2H), 1.40 – 1.22 (m, 13H), 0.87 (t, J = 6.9 Hz, 3H). **^{13}C NMR** (100 MHz, $CDCl_3$) δ 202.1, 161.3, 126.5, 45.6, 32.1, 30.3, 30.0, 29.7, 29.5, 29.4, 27.7, 27.3, 24.4, 22.9, 14.3. **HRMS (ESI)** m/z: [M + Na]+ Calcd for $C_{15}H_{26}ONa$ 245.1876; Found 245.1887. **$[\alpha]_D^{26}$** = +32.0 (c = 0.1, $CHCl_3$).

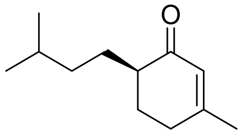

(*R*)-6-isopentyl-3-methylcyclohex-2-en-1-one (2k) Colorless oil (16.9 mg, 94% yield, 94% *ee*), prepared according to the general procedure for the asymmetric hydrogenation on a 0.1 mmol scale using catalyst **D**. The product was purified by flash chromatography (pentane/Et_2O, 50:50) on silica gel. **^{1}H NMR** (400 MHz, $CDCl_3$) δ 5.83 (dd, J = 2.8, 1.4 Hz, 1H), 2.29 (t, J = 5.8 Hz, 2H), 2.11 (dddt, J = 18.1, 15.1, 13.2, 4.9 Hz, 2H), 1.97 – 1.90 (m, 3H), 1.89 – 1.66 (m, 2H), 1.54 (td, J = 13.3, 6.6 Hz, 1H), 1.36 – 1.12 (m, 3H), 0.88 (dd, J = 6.6, 2.7 Hz, 6H). **^{13}C NMR** (100 MHz, $CDCl_3$) δ 202.1,

161.4, 126.5, 45.9, 36.5, 30.3, 28.4, 27.7, 27.2, 24.4, 23.0, 22.7. **HRMS (ESI)** m/z: [M + Na]+ Calcd for $C_{12}H_{20}ONa$ 203.1406; Found 203.1407. **[α]$_D$^{26}** = +33.0 (c = 0.1, $CHCl_3$).

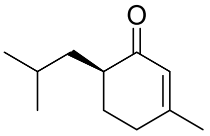

(*S*)-6-isobutyl-3-methylcyclohex-2-en-1-one (2l) Colorless oil (15.4 mg, 93% yield, 94% *ee*), prepared according to the general procedure for the asymmetric hydrogenation on a 0.1 mmol scale using catalyst **E**. The product was purified by flash chromatography (pentane/Et_2O, 50:50) on silica gel. **^{1}H NMR** (400 MHz, $CDCl_3$) δ 5.82 (dd, *J* = 2.8, 1.4 Hz, 1H), 2.35 – 2.18 (m, 3H), 2.06 (ddd, *J* = 13.4, 10.2, 5.1 Hz, 1H), 1.92 (d, *J* = 0.9 Hz, 3H), 1.76 – 1.61 (m, 3H), 1.22 – 1.13 (m, 1H), 0.99 – 0.90 (m, 3H), 0.89 – 0.83 (m, 3H). **^{13}C NMR** (100 MHz, $CDCl_3$) δ 202.4, 161.2, 126.5, 43.4, 38.4, 30.1, 27.8, 25.5, 24.3, 23.6, 21.9. **HRMS (ESI)** m/z: [M + Na]+ Calcd for $C_{11}H_{18}ONa$ 189.1250; Found 189.1249. **[α]$_D$^{26}** = +15.0 (c = 0.1, $CHCl_3$).

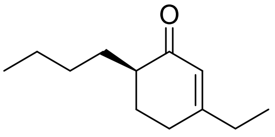

(*R*)-6-butyl-3-ethylcyclohex-2-en-1-one (2m) Colorless oil (17.3 mg, 96% yield, 97% *ee*), prepared according to the general procedure for the asymmetric hydrogenation on a 0.1 mmol scale using catalyst **D**. The product was purified by flash chromatography (pentane/Et_2O, 50:50) on silica gel. **^{1}H NMR** (400 MHz, $CDCl_3$) δ 5.82 (dd, *J* = 2.4, 1.2 Hz, 1H), 2.35 – 2.25 (m, 2H), 2.24 – 2.13 (m, 3H), 2.11 – 2.03 (m, 1H), 1.87 – 1.76 (m, 1H), 1.76 – 1.65 (m, 1H), 1.39 – 1.22 (m, 5H), 1.08 (t, *J* = 7.4 Hz, 3H), 0.89 (t, *J* = 6.9 Hz, 3H). **^{13}C NMR** (100 MHz, $CDCl_3$) δ 202.4, 166.5, 124.4, 46.0, 30.8, 29.5, 29.1, 29.0, 28.0, 23.0, 14.2, 11.5. **HRMS (ESI)** m/z: [M + Na]+ Calcd for $C_{12}H_{20}ONa$ 203.1406; Found 203.1409.

[α]$_D$^{26} = +35.0 (c = 0.1, $CHCl_3$).

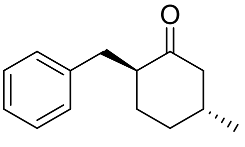

(2*S*,5*R*)-2-benzyl-5-methylcyclohexan-1-one (3a) Colorless oil (19.4 mg, 96% yield, 99% *ee*, 99/1 d.r.), prepared according to the general procedure for the asymmetric hydrogenation on a 0.1 mmol scale using catalyst **B**. The product was purified by flash chromatography (pentane/Et_2O, 50:50) on silica gel Spectroscopic data was in agreement with reported values.[8] **^{1}H NMR** (400 MHz, $CDCl_3$) δ 7.32 – 7.24 (m, 2H), 7.18 (ddd, *J* = 14.0, 6.8, 4.1 Hz, 3H), 3.25 (dd, *J* = 13.8, 4.5 Hz, 1H), 2.57 – 2.34 (m, 3H), 2.11 – 1.96 (m, 2H), 1.92 – 1.76 (m, 2H), 1.39 – 1.24 (m, 2H), 1.02 (d, *J* = 6.4 Hz, 3H). **^{13}C NMR** (100 MHz, $CDCl_3$) δ 212.1, 140.7, 129.3, 128.5, 126.1, 51.8, 50.7, 35.9, 35.4, 34.2, 32.6, 22.6. **HRMS (ESI)** m/z: [M + Na]+ Calcd for $C_{14}H_{18}ONa$ 225.1250; Found 225.1249. **[α]$_D$^{26}** = -27.0 (c = 0.1, $CHCl_3$).

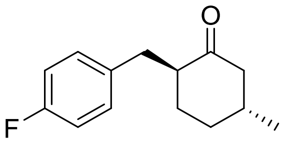
(2*S*,5*R*)-2-(4-fluorobenzyl)-5-methylcyclohexan-1-one (3b) Colorless oil (21.6 mg, 98% yield, 99% *ee*, 99/1 d.r.), prepared according to the general procedure for the asymmetric hydrogenation on a 0.1 mmol scale using catalyst **B**. The product was purified by flash chromatography (pentane/Et_2O, 50:50) on silica gel. **^{1}H NMR** (400 MHz, $CDCl_3$) δ 7.17 – 7.05 (m, 2H), 6.95 (ddd, *J* = 10.9, 5.8, 2.6 Hz, 2H), 3.18 (dd, *J* = 13.6, 4.6 Hz, 1H), 2.55 – 2.31 (m, 3H), 2.08 – 1.91 (m, 2H), 1.91 – 1.76 (m, 2H), 1.38 – 1.24 (m, 2H), 1.01 (d, *J* = 6.3 Hz, 3H). **^{13}C NMR** (100 MHz, $CDCl_3$) δ 211.9, 161.5 (d, $^1J_{C\text{-}F}$ = 243.6 Hz) 136.3 (d, $^4J_{C\text{-}F}$ = 3.2 Hz), 130.7 (d, $^3J_{C\text{-}F}$ = 7.8 Hz), 115.2 (d, $^2J_{C\text{-}F}$ = 21.1 Hz), 51.8, 50.7, 36.0, 34.6, 34.2, 32.7, 22.6. **^{19}F NMR** (377 MHz, $CDCl_3$) δ -117.6. **HRMS (ESI)** m/z: [M + Na]+ Calcd for $C_{14}H_{17}FONa$ 243.1156; Found 243.1152. **$[\alpha]_D^{26}$** = -23.0 (c = 0.1, $CHCl_3$).

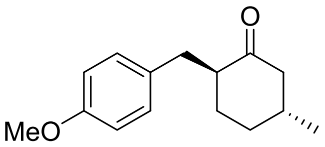
(2*S*,5*R*)-2-(4-methoxybenzyl)-5-methylcyclohexan-1-one (3n) Colorless oil (23.0 mg, 99% yield, 99% *ee*, 99/1 d.r.), prepared according to the general procedure for the asymmetric hydrogenation on a 0.1 mmol scale using catalyst **B**. The product was purified by flash chromatography (pentane/Et_2O, 50:50) on silica gel. **^{1}H NMR** (400 MHz, $CDCl_3$) δ 7.07 (d, *J* = 8.6 Hz, 2H), 6.81 (d, *J* = 8.7 Hz, 2H), 3.78 (s, 3H), 3.17 (dd, *J* = 13.9, 4.6 Hz, 1H), 2.39 (dddd, *J* = 29.3, 22.5, 9.6, 6.4 Hz, 3H), 2.09 – 1.97 (m, 2H), 1.91 – 1.77 (m, 2H), 1.36 – 1.24 (m, 2H), 1.01 (d, *J* = 6.3 Hz, 3H). **^{13}C NMR** (100 MHz, $CDCl_3$) δ 212.3, 158.1, 132.7, 130.2, 113.9, 55.4, 52.0, 50.7, 36.0, 34.5, 34.2, 32.6, 22.6. **HRMS (ESI)** m/z: [M + Na]+ Calcd for $C_{15}H_{20}O_2Na$ 255.1356; Found 255.1363.**$[\alpha]_D^{26}$** = -26.0 (c = 0.1, $CHCl_3$).

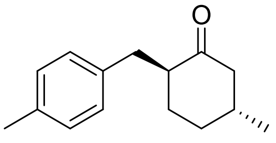
(2*S*,5*R*)-5-methyl-2-(4-methylbenzyl)cyclohexan-1-one (3c) Colorless oil (21.4 mg, 99% yield, 99% *ee*, 99/1 d.r.), prepared according to the general procedure for the asymmetric hydrogenation on a 0.1 mmol scale using catalyst **B**. The product was purified by flash chromatography (pentane/Et_2O, 50:50) on silica gel. **^{1}H NMR** (400 MHz, $CDCl_3$) δ 7.07 (q, *J* = 8.0 Hz, 4H), 3.20 (dd, *J* = 13.9, 4.6 Hz, 1H), 2.53 – 2.33 (m, 3H), 2.32 (s, 3H), 2.09 – 1.98 (m, 2H), 1.91 – 1.77 (m, 2H), 1.38 – 1.23 (m, 2H), 1.01 (d, *J* = 6.3 Hz, 3H). **^{13}C NMR** (100 MHz, $CDCl_3$) δ 212.2, 137.6, 135.6, 129.2, 129.2, 51.9, 50.7, 36.0, 35.0, 34.2, 32.6, 22.6, 21.2. **HRMS (ESI)** m/z: [M + Na]+ Calcd for $C_{15}H_{20}ONa$ 239.1406; Found 239.1417. **$[\alpha]_D^{26}$** = -23.0 (c = 0.1, $CHCl_3$).

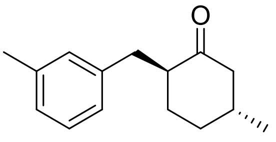
(2*S*,5*R*)-5-methyl-2-(3-methylbenzyl)cyclohexan-1-one (3d) Colorless oil (19.7 mg, 91% yield, 99% *ee*, 99/1 d.r.), prepared according to the general procedure for the asymmetric hydrogenation on a 0.1 mmol scale using catalyst **B**. The product was purified by flash chromatography (pentane/Et_2O, 50:50) on silica gel. **^{1}H NMR** (400 MHz, CDCl3) δ 7.16 (t, J = 7.5 Hz, 1H), 7.08 – 6.89

(m, 3H), 3.22 (dd, J = 13.9, 4.6 Hz, 1H), 2.57 – 2.27 (m, 6H), 2.13 – 1.96 (m, 2H), 1.94 – 1.76 (m, 2H), 1.39 – 1.23 (m, 2H), 1.02 (d, J = 6.3 Hz, 3H). **^{13}C NMR** (100 MHz, $CDCl_3$) δ 212.2, 140.7, 138.0, 130.1, 128.4, 126.9, 126.3, 51.8, 50.7, 36.0, 35.3, 34.2, 32.7, 22.6, 21.6. **HRMS (ESI)** m/z: [M + Na]+ Calcd for $C_{15}H_{20}ONa$ 239.1406; Found 239.1405. **$[\alpha]_D^{26}$** = -21.0 (c = 0.1, $CHCl_3$).

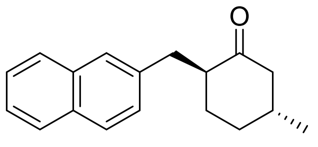

(2*S*,5*R*)-5-methyl-2-(naphthalen-2-ylmethyl)cyclohexan-1-one (3e) Colorless oil (239.4 mg, 97% purity, 92% yield, 99% *ee*, 99/1 d.r.), prepared according to the general procedure for the asymmetric hydrogenation on a 1 mmol scale using catalyst **B**. The product was purified by flash chromatography (pentane/Et_2O, 50:50) on silica gel. **^{1}H NMR** (400 MHz, $CDCl_3$) δ 7.84 – 7.73 (m, 3H), 7.60 (s, 1H), 7.43 (pd, *J* = 6.9, 1.5 Hz, 2H), 7.30 (dd, *J* = 8.4, 1.7 Hz, 1H), 3.48 – 3.34 (m, 1H), 2.64 – 2.52 (m, 2H), 2.43 (ddd, *J* = 13.0, 3.9, 2.2 Hz, 1H), 2.11 – 2.00 (m, 2H), 1.93 – 1.76 (m, 2H), 1.43 – 1.25 (m, 2H), 1.01 (t, *J* = 5.7 Hz, 3H). **^{13}C NMR** (100 MHz, $CDCl_3$) δ 212.1, 138.3, 133.7, 132.2, 128.1, 127.9, 127.8, 127.6, 126.2, 125.4, 51.8, 50.7, 36.0, 35.6, 34.2, 32.7, 22.6. **HRMS (ESI)** m/z: [M + Na]+ Calcd for $C_{18}H_{20}ONa$ 275.1406; Found 275.1412. **$[\alpha]_D^{26}$** = -14.0 (c = 0.1, $CHCl_3$).

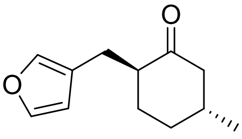

(2*S*,5*R*)-2-(furan-3-ylmethyl)-5-methylcyclohexan-1-one (3f) Colorless oil (18.4 mg, 96% yield, 99% *ee*, 97/3 d.r.), prepared according to the general procedure for the asymmetric hydrogenation on a 0.1 mmol scale using catalyst **F**. The product was purified by flash chromatography (pentane/Et_2O, 50:50) on silica gel. **^{1}H NMR** (400 MHz, $CDCl_3$) δ 7.32 (t, *J* = 1.7 Hz, 1H), 7.20 (dd, *J* = 1.5, 0.7 Hz, 1H), 6.23 (d, *J* = 0.9 Hz, 1H), 2.95 – 2.87 (m, 1H), 2.45 – 2.35 (m, 2H), 2.29 (dd, *J* = 14.5, 7.7 Hz, 1H), 2.14 – 1.98 (m, 2H), 1.90 – 1.78 (m, 2H), 1.41 – 1.24 (m, 2H), 1.01 (d, *J* = 6.2 Hz, 3H). **^{13}C NMR** (100 MHz, $CDCl_3$) δ 212.0, 142.8, 140.0, 123.0, 111.7, 50.7, 50.7, 35.9, 34.2, 32.7, 24.4, 22.6. **HRMS (ESI)** m/z: [M + Na]+ Calcd for $C_{12}H_{16}O_2Na$ 215.1043; Found 215.1060. **$[\alpha]_D^{26}$** = -10.0 (c = 0.1, $CHCl_3$).

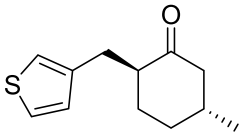

(2*S*,5*R*)-5-methyl-2-(thiophen-3-ylmethyl)cyclohexan-1-one (3o) Colorless oil (20.2 mg, 97% yield, 99% *ee*, 99/1 d.r.), prepared according to the general procedure for the asymmetric hydrogenation on a 0.1 mmol scale using catalyst **F**. The product was purified by flash chromatography (pentane/Et_2O, 50:50) on silica gel. **^{1}H NMR** (400 MHz, $CDCl_3$) δ 7.23 (dd, *J* = 4.9, 3.0 Hz, 1H), 6.96 – 6.92 (m, 1H), 6.90 (dd, *J* = 4.9, 1.2 Hz, 1H), 3.17 (q, *J* = 8.1 Hz, 1H), 2.55 – 2.44 (m, 2H), 2.44 – 2.37 (m, 1H), 2.10 – 2.00 (m, 2H), 1.91 – 1.78 (m, 2H), 1.39 – 1.26 (m, 2H), 1.02 (d, *J* = 6.3 Hz, 3H). **^{13}C NMR** (100 MHz, $CDCl_3$) δ 212.0, 140.8, 128.9, 125.4, 121.5, 51.2, 50.7, 35.9, 34.2, 32.8, 29.8, 22.6. **HRMS (ESI)** m/z: [M + Na]+ Calcd for $C_{12}H_{16}OSNa$ 231.0814; Found 231.0811. **$[\alpha]_D^{26}$** = -20.0 (c = 0.1, $CHCl_3$).

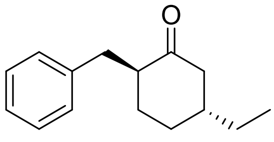

(2*S*,5*R*)-2-benzyl-5-ethylcyclohexan-1-one (3g) Colorless oil (21.4 mg, 99% yield, 99% *ee*, 99/1 d.r.), prepared according to the general procedure for the asymmetric hydrogenation on a 0.1 mmol scale using catalyst **F**. The product was purified by flash chromatography (pentane/Et_2O, 50:50) on silica gel. Spectroscopic data was in agreement with reported values.[9] **^{1}H NMR** (400 MHz, $CDCl_3$) δ 7.27 (ddd, *J* = 7.5, 2.3, 1.2 Hz, 2H), 7.18 (ddd, *J* = 13.2, 6.8, 4.1 Hz, 3H), 3.25 (dd, *J* = 13.9, 4.6 Hz, 1H), 2.58 – 2.44 (m, 2H), 2.38 (dd, *J* = 13.9, 8.7 Hz, 1H), 2.08 – 1.98 (m, 2H), 1.87 (ddd, *J* = 11.2, 5.5, 2.1 Hz, 1H), 1.71 – 1.60 (m, 1H), 1.43 – 1.23 (m, 4H), 0.89 (t, *J* = 7.5 Hz, 3H). **^{13}C NMR** (100 MHz, $CDCl_3$) δ 212.3, 140.7, 129.3, 128.5, 126.1, 52.2, 48.5, 42.5, 35.5, 32.6, 31.8, 30.0, 11.4. **HRMS (ESI)** m/z: [M + Na]+ Calcd for $C_{15}H_{20}ONa$ 239.1406; Found 239.1411. **$[\alpha]_D^{26}$** = -21.0 (c = 0.1, $CHCl_3$).

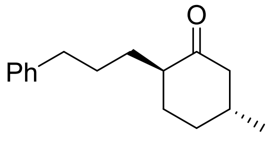

(2*R*,5*R*)-5-methyl-2-(3-phenylpropyl)cyclohexan-1-one (3i) Colorless oil (22.3 mg, 97% yield, 99% *ee*, 90/10 d.r.), prepared according to the general procedure for the asymmetric hydrogenation on a 0.1 mmol scale using catalyst **C**. The product was purified by flash chromatography (pentane/Et_2O, 50:50) on silica gel. **^{1}H NMR** (400 MHz, $CDCl_3$) δ 7.31 – 7.24 (m, 2H), 7.20 – 7.14 (m, 3H), 2.69 – 2.53 (m, 2H), 2.36 (ddd, *J* = 12.8, 3.7, 2.2 Hz, 1H), 2.19 (tt, *J* = 7.0, 3.4 Hz, 1H), 2.13 – 2.07 (m, 1H), 2.05 – 1.92 (m, 1H), 1.93 – 1.79 (m, 3H), 1.68 – 1.57 (m, 2H), 1.43 – 1.17 (m, 3H), 1.01 (d, *J* = 6.3 Hz, 3H). **^{13}C NMR** (100 MHz, $CDCl_3$) δ 212.8, 142.7, 128.6, 128.4, 125.8, 50.8, 50.0, 36.4, 35.9, 34.3, 33.1, 29.3, 29.0, 22.6. **HRMS (ESI)** m/z: [M + Na]+ Calcd for ; $C_{16}H_{22}ONa$ 253.1563; Found 253.1569. **$[\alpha]_D^{26}$** = +3.0 (c = 0.1, $CHCl_3$).

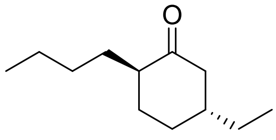

(2*R*,5*R*)-2-butyl-5-ethylcyclohexan-1-one (3m) Colorless oil (17.5 mg, 96% yield, 99% *ee*, 97/3 d.r.), prepared according to the general procedure for the asymmetric hydrogenation on a 0.1 mmol scale using catalyst **C**. The product was purified by flash chromatography (pentane/Et_2O, 50:50) on silica gel. **^{1}H NMR** (400 MHz, $CDCl_3$) δ 2.42 (ddd, *J* = 12.8, 4.0, 2.1 Hz, 1H), 2.16 (ddt, *J* = 12.5, 5.7, 4.4 Hz, 2H), 1.98 (td, *J* = 12.8, 1.1 Hz, 1H), 1.94 – 1.88 (m, 1H), 1.83 – 1.74 (m, 1H), 1.69 – 1.58 (m, 1H), 1.43 – 1.23 (m, 9H), 0.91 – 0.87 (m, 6H). **^{13}C NMR** (100 MHz, $CDCl_3$) δ 213.3, 50.5, 48.6, 42.5, 33.1, 31.9, 30.0, 29.7, 28.9, 23.1, 14.3, 11.4. **HRMS (ESI)** m/z: [M + Na]+ Calcd for $C_{12}H_{22}ONa$ 205.1563; Found 205.1556. **$[\alpha]_D^{26}$** = +4.0 (c = 0.1, $CHCl_3$).

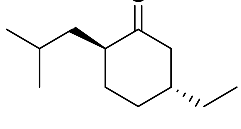

(2*S*,5*R*)-5-ethyl-2-isobutylcyclohexan-1-one (3p) Colorless oil (17.1 mg, 94% yield, 98% *ee*, 93/7 d.r.), prepared according to the general procedure for the asymmetric hydrogenation on a 0.1 mmol scale using catalyst **C**. The product was purified by flash chromatography (pentane/Et_2O, 50:50) on silica gel. **^{1}H NMR** (400 MHz, $CDCl_3$) δ 2.42 (ddd, *J* = 12.8, 3.9, 2.1 Hz, 1H), 2.27 (ddd, *J* =

7.0, 6.4, 1.2 Hz, 1H), 2.11 (ddt, J = 12.6, 6.3, 3.0 Hz, 1H), 2.00 (td, J = 12.8, 1.2 Hz, 1H), 1.95 – 1.87 (m, 1H), 1.75 – 1.59 (m, 3H), 1.43 – 1.23 (m, 4H), 1.01 (ddd, J = 13.2, 7.2, 5.8 Hz, 1H), 0.92 – 0.83 (m, 9H). **^{13}C NMR** (100 MHz, $CDCl_3$) δ 213.4, 48.7, 48.3, 42.5, 38.2, 33.4, 31.9, 30.0, 25.4, 23.4, 22.4, 11.4. **HRMS (ESI)** m/z: [M + Na]+ Calcd for $C_{12}H_{22}ONa$ 205.1563; Found 205.1556. **[α]$_D$^{26}** = -5.0 (c = 0.1, $CHCl_3$).

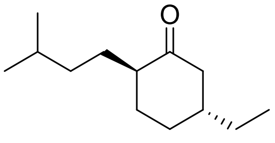

(2*R*,5*R*)-5-ethyl-2-isopentylcyclohexan-1-one (3q) Colorless oil (18.6 mg, 95% yield, 99% *ee*, 95/5 d.r.), prepared according to the general procedure for the asymmetric hydrogenation on a 0.1 mmol scale using catalyst **C**. The product was purified by flash chromatography (pentane/Et_2O, 50:50) on silica gel. **^{1}H NMR** (400 MHz, $CDCl_3$) δ 2.41 (ddd, J = 12.8, 3.9, 2.1 Hz, 1H), 2.20 – 2.10 (m, 2H), 1.98 (td, J = 12.8, 1.0 Hz, 1H), 1.93 – 1.86 (m, 1H), 1.82 – 1.75 (m, 1H), 1.69 – 1.60 (m, 1H), 1.51 (dt, J = 13.1, 6.6 Hz, 1H), 1.43 – 1.24 (m, 4H), 1.19 – 1.08 (m, 3H), 0.93 – 0.85 (m, 9H). **^{13}C NMR** (100 MHz, $CDCl_3$) δ 213.2, 50.8, 48.6, 42.5, 36.7, 33.1, 31.9, 30.0, 28.5, 27.0, 22.9, 22.7, 11.4. **HRMS (ESI)** m/z: [M + Na]+ Calcd for $C_{13}H_{24}ONa$ 219.1719; Found 219.1723. **[α]$_D$^{26}** = +3.0 (c = 0.1, $CHCl_3$).

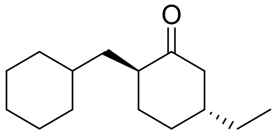

(2*S*,5*R*)-2-(cyclohexylmethyl)-5-ethylcyclohexan-1-one (3r) Colorless oil (20.4 mg, 92% yield, 99% *ee*, 97/3 d.r.), prepared according to the general procedure for the asymmetric hydrogenation on a 0.1 mmol scale using catalyst **C**. The product was purified by flash chromatography (pentane/Et_2O, 50:50) on silica gel. **^{1}H NMR** (400 MHz, $CDCl_3$) δ 2.42 (ddd, J = 12.8, 3.9, 2.1 Hz, 1H), 2.38 – 2.28 (m, 1H), 2.15 – 2.05 (m, 1H), 1.99 (td, J = 12.7, 1.2 Hz, 1H), 1.93 – 1.87 (m, 1H), 1.76 – 1.58 (m, 7H), 1.43 – 1.10 (m, 8H), 1.04 – 0.94 (m, 1H), 0.89 (td, J = 7.4, 3.2 Hz, 4H), 0.82 (dd, J = 17.4, 5.8 Hz, 1H). **^{13}C NMR** (100 MHz, $CDCl_3$) δ 213.6, 48.7, 47.5, 42.6, 36.7, 35.0, 34.2, 33.5, 33.2, 31.9, 30.0, 26.9, 26.6, 26.5, 11.4. **HRMS (ESI)** m/z: [M + Na]+ Calcd for $C_{15}H_{26}ONa$ 245.1876; Found 245.1875. **[α]$_D$^{26}** = -7.0 (c = 0.1, $CHCl_3$).

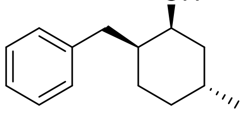

(1*S*,2*S*,5*R*)-2-benzyl-5-methylcyclohexan-1-ol (4a) Colorless oil (16.9 mg, 83% yield, 99% *ee*, >95/5 d.r.), prepared according to the general procedure for the asymmetric hydrogenation on a 0.1 mmol scale using catalyst **C**. The product was purified by flash chromatography (pentane/Et_2O, 100:0 to 50:50) on silica gel. **^{1}H NMR** (400 MHz, $CDCl_3$) δ 7.32 – 7.26 (m, 2H), 7.22 – 7.16 (m, 3H), 3.80 (d, J = 2.4 Hz, 1H), 2.70 (dd, J = 13.4, 7.8 Hz, 1H), 2.56 (dd, J = 13.4, 7.2 Hz, 1H), 1.84 – 1.67 (m, 3H), 1.63 – 1.55 (m, 1H), 1.52 – 1.40 (m, 2H), 1.30 (d, J = 1.4 Hz, 1H), 1.09 (ddd, J = 13.3, 8.2, 2.1 Hz, 1H), 0.94 – 0.82 (m, 4H). **^{13}C NMR** (100 MHz, $CDCl_3$) δ 141.2, 129.3, 128.4, 126.0, 68.8, 43.6, 42.6, 39.5, 35.0, 26.5, 26.2, 22.5. **HRMS (ESI)** m/z: [M + Na]+ Calcd for $C_{14}H_{20}ONa$ requires 227.1406; Found 227.1406. **[α]$_D$^{26}** = +26.0 (c = 0.1, $CHCl_3$).

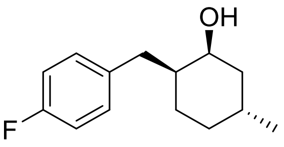

(1*S*,2*S*,5*R*)-2-(4-fluorobenzyl)-5-methylcyclohexan-1-ol (4b) Colorless oil (16.7 mg, 75% yield, 99% *ee*, >95/5 d.r.), prepared according to the general procedure for the asymmetric hydrogenation on a 0.1 mmol scale using catalyst **C**. The product was purified by flash chromatography (pentane/Et_2O, 100:0 to 50:50) on silica gel. **^{1}H NMR** (400 MHz, $CDCl_3$) δ 7.20 – 7.08 (m, 2H), 7.05 – 6.90 (m, 2H), 3.77 (dd, J = 5.2, 2.5 Hz, 1H), 2.67 (dd, J = 13.5, 7.8 Hz, 1H), 2.52 (dd, J = 13.5, 7.1 Hz, 1H), 1.83 – 1.65 (m, 3H), 1.59 – 1.37 (m, 3H), 1.30 (s, 1H), 1.09 (ddd, J = 13.4, 8.1, 2.0 Hz, 1H), 0.92 – 0.82 (m, 4H). **^{13}C NMR** (100 MHz, $CDCl_3$) δ 161.5 (d, $^1J_{C-F}$ = 243.2 Hz), 136.8 (d, $^4J_{C-F}$ = 3.2 Hz), 130.6 (d, $^3J_{C-F}$ = 7.7 Hz), 115.2 (d, $^2J_{C-F}$ = 21.0 Hz), 68.6, 43.6, 42.6, 38.6, 34.9, 26.5, 26.2, 22.5. **^{19}F NMR** (377 MHz, $CDCl_3$) δ 117.9. **HRMS (ESI)** m/z: [M + Na]+ Calcd for $C_{14}H_{19}FONa$ 245.1312; Found 245.1316. **$[\alpha]_D^{26}$** = +23.0 (c = 0.1, $CHCl_3$).

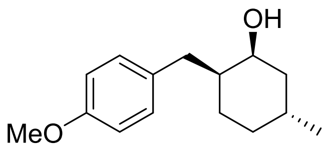

(1*S*,2*S*,5*R*)-2-(4-methoxybenzyl)-5-methylcyclohexan-1-ol (4n) Colorless oil (19.0 mg, 81% yield, 99% *ee*, >95/5 d.r.), prepared according to the general procedure for the asymmetric hydrogenation on a 0.1 mmol scale using catalyst **C**. The product was purified by flash chromatography (pentane/Et_2O, 100:0 to 50:50) on silica gel. **^{1}H NMR** (400 MHz, $CDCl_3$) δ 7.16 – 7.06 (m, 2H), 6.89 – 6.77 (m, 2H), 3.79 (s, 4H), 2.63 (dd, J = 13.6, 7.8 Hz, 1H), 2.50 (dd, J = 13.6, 7.1 Hz, 1H), 1.85 – 1.63 (m, 3H), 1.61 – 1.25 (m, 4H), 1.09 (ddd, J = 13.8, 11.8, 2.1 Hz, 1H), 0.94 – 0.83 (m, 4H). **^{13}C NMR** (100 MHz, $CDCl_3$) δ 158.0, 133.2, 130.2, 113.9, 68.7, 55.5, 43.7, 42.6, 38.6, 35.0, 26.5, 26.3, 22.5. **HRMS (ESI)** m/z: [M + Na]+ Calcd for $C_{15}H_{22}O_2Na$ 257.1512; Found 257.1517. **$[\alpha]_D^{26}$** = +20.0 (c = 0.1, $CHCl_3$).

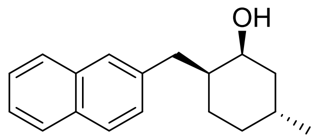

(1S,2S,5R)-5-methyl-2-(naphthalen-2-ylmethyl)cyclohexan-1-ol (4e) Colorless oil (21.8 mg, 86% yield, 99% *ee*, >95/5 d.r.), prepared according to the general procedure for the asymmetric hydrogenation on a 0.1 mmol scale using catalyst **C**. The product was purified by flash chromatography (pentane/Et_2O, 100:0 to 50:50) on silica gel. **^{1}H NMR** (400 MHz, $CDCl_3$) δ. 7.85 – 7.73 (m, 3H), 7.63 (s, 1H), 7.44 (pd, J = 6.9, 1.4 Hz, 2H), 7.35 (dd, J = 8.4, 1.7 Hz, 1H), 3.87 – 3.77 (m, 1H), 2.87 (dd, J = 13.4, 8.0 Hz, 1H), 2.73 (dd, J = 13.4, 7.2 Hz, 1H), 1.85 – 1.65 (m, 4H), 1.59 – 1.45 (m, 2H), 1.10 (ddd, J = 15.4, 13.0, 2.3 Hz, 1H), 0.96 – 0.81 (m, 4H). **^{13}C NMR** (100 MHz, $CDCl_3$) δ 138.8, 133.8, 132.2, 128.1, 128.0, 127.8, 127.6, 127.4, 126.1, 125.3, 68.8, 43.6, 42.6, 39.7, 35.0, 26.6, 26.2, 22.5. **HRMS (ESI)** m/z: [M + Na]+ Calcd for $C_{18}H_{22}ONa$ 277.1563; Found 277.1573. **$[\alpha]_D^{26}$** = +25.0 (c = 0.1, $CHCl_3$).

3. Optimization of the asymmetric hydrogenation

Table S1. Optimization for aromatic substrate **1a**

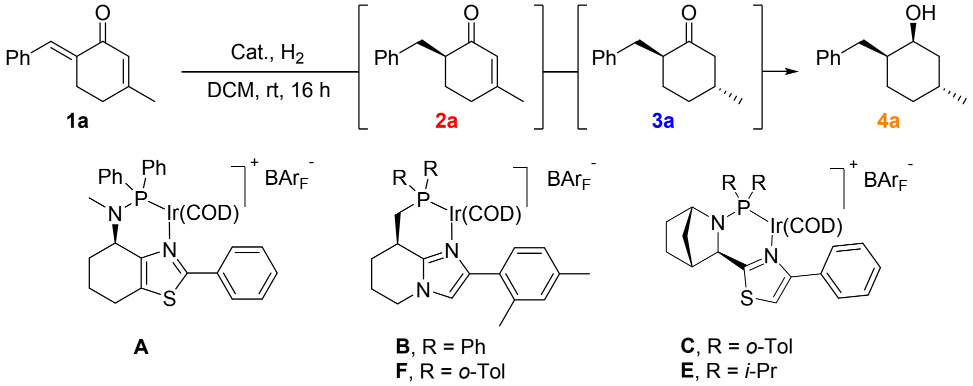

Entry	Catalyst (mol%)	H_2 (bar)	**1a**:**2a**:**3a**:**4a**	*ee* (%) **2a**	*ee* (%), d.r. **3a**	*ee* (%), d.r. **4a**
1	**A** (1.0)	50	0:95:5:0	99	n.d.	-
2	**B** (1.0)	50	0:0:100:0	-	99, 99/1	-
3	**F** (1.0)	50	0:0:100:0	-	99, 99/1	-
4	**C** (1.0)	50	0:0:92:8	-	99, 96/4	n.d., >95/5
5	**E** (1.0)	50	0:0:100:0	-	99, 94/6	-
6	**A** (0.5)	20	0:100:0:0	99	-	-
7	**B** (0.5)	20	0:70:30:0	99	99, 99/1	-
8	**B** (1.0)	20	0:0:100:0	-	99, 99/1	-
9	**C** (4.0)	100	0:0:12:88	-	99, 69/31	99, >95/5

Reaction conditions: 0.05 mmol substrate, 0.5 mL DCM, rt, 16 h. Product distribution was determined by ^{1}H NMR spectroscopy. Stereoselectivity was determined by GC or SFC analysis using a chiral stationary phase combined with ^{1}H NMR spectroscopy for **4a**.

Table S2. Optimization for aliphatic substrate **1s/m**

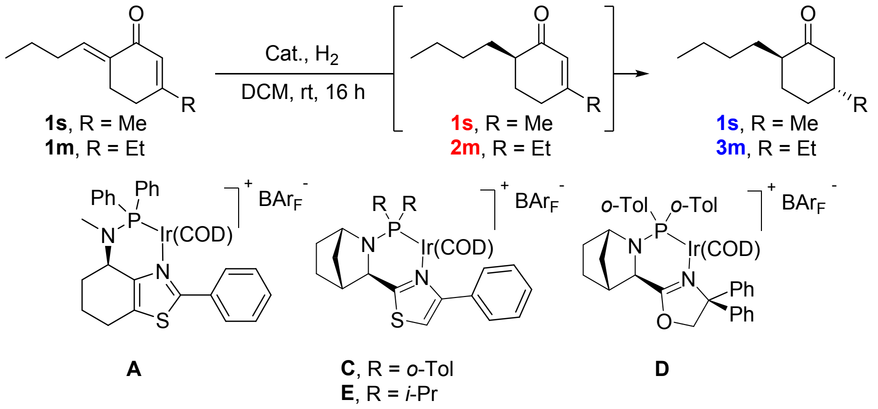

Entry	Catalyst (mol%)	R	H_2 (bar)	**1**:**2**:**3**	*ee* (%) **2**	*ee* (%), d.r. **3**
1	**A** (1.0)	Me	50	0:98:2	87	n.d.
2	**C** (1.0)	Me	50	0:0:100	-	n.d.[a]
3	**E** (1.0)	Me	50	0:85:15	95	n.d.[a]
4	**D** (1.0)	Me	50	0:100:0	97	-
5	**D** (1.0)	Me	20	19:81:0	97	-
6	**C** (1.0)	Et	50	0:0:100	-	99, 97/3

Reaction conditions: 0.05 mmol substrate, 0.5 mL DCM, rt, 16 h. Product distribution was determined by ^{1}H NMR spectroscopy. Stereoselectivity was determined by GC or SFC analysis using a chiral stationary phase. [a]Stereoselectivity was not determined as a result of incomplete separation of the stereoisomers of product **1s**.

4. Assignment of the absolute configuration of the products

The cyclic dienones in this work undergo the asymmetric hydrogenation in a sequential manner. At first, the *exo*-cyclic alkene is hydrogenated, followed by the *endo*-cyclic olefin and the ketone as last. The *relative* configuration was assigned by comparing the ^{1}H-NMR chemical shifts for products **3a** and **3g** with literature values, and was found in agreement with *trans*-**3a**[8] and *trans*-**3g**.[9] The *absolute* configuration of **3g** was assigned by comparing the sign of optical rotation with literature values. As opposite sign of rotation was measured for **3g** in this work compared to the reference, the absolute configuration was assigned to be (2*S*,5*R*)-**3g**. As product **3g** was formed *via* **2g**, the absolute configuration of **2g** was assigned by analogy. The installation of these two stereocenters is in accordance with the predicted outcome based on the proposed mechanism operative in the hydrogenation of conjugated unsaturated carbonyl compounds with N,P-iridium catalysts.[7,10-13] The absolute configuration of the remaining monohydrogenated products (**2**) and double hydrogenated products (**3**) was assigned by analogy. The assignment of absolute configuration of the carbinol carbon in products **4** was based on the Karplus relationship, see manuscript Scheme 5b.

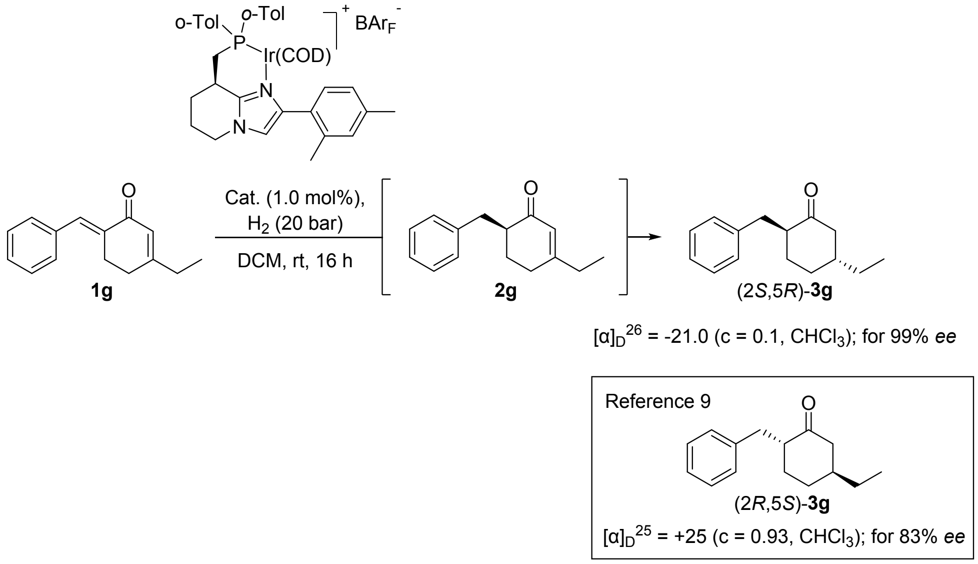

5. References

1) Engman, M.; Diesen, J. S.; Paptchikhine, A.; Andersson, P. G. *J. Am. Chem. Soc.* **2007**, *129*, 4536-4537.
2) Peters, B. K.; Liu, J.; Margarita, C.; Rabten, W.; Kerdphon, S.; Orebom, A.; Morsch, T.; Andersson, P. G. *J. Am. Chem. Soc.* **2016**, *138*, 11930-11935.
3) Li, J.-Q.; Paptchikhine, A.; Govender, T.; Andersson, P. G. *Tetrahedron Asymm* **2010**, 1328-1333.
4) Ponra, S.; Yang, J.; Kerdphon, S.; Andersson, P. G. *Angew. Chem. Int. Ed.* **2019**, *58*, 9282-9287.
5) Yang, J.; Massaro, L.; Krajangsri, S.; Singh, T.; Su, H.; Silvi, E.; Ponra, S.; Eriksson, L.; Ahlquist, M. S. G.; Andersson, P. G. *J. Am. Chem. Soc.* **2021**, *143*, 21594-21603.
6) Peters, B. B. C.; Zheng, J.; Krajangsri, S.; Andersson, P. G. *J. Am. Chem. Soc.* **2022**, *144*, 8734-8740.
7) Zheng, J.; Peters, B. B. C.; Jiang, W.; Suarez, L. A.; Ahlquist, M. S. G.: Singh, T.; Andersson, P. G. *Chem. Eur. J.* **2024**, *30*, e202303406.
8) Kalutharage, N.; Yi, C. S. *Angew. Chem. Int. Ed.* **2013**, *52*, 13651-13655.
9) Selim, K.; Soeta, T.; Yamada, K.-I.; Tomioka, K. *Chem. Asian J.* **2008**, *3*, 342-350.
10) Li, M.-L.; Yang, S.; Su, X.-C.; Wu, H.-L.; Yang, L.-L.; Zhu, S.-F.; Zhou, Q.-L. *J. Am. Chem. Soc.* **2017**, *139*, 541-547.
11) Engel, J.; Mersmann, S.; Norrby, P.-O.; Bolm, C. *ChemCatChem* **2016**, *8*, 3099-3106.
12) Zhou, J.; Ogle, J. W.; Fan, Y.; Banphavichit(Bee), V.; Zhu, Y.; Burgess K. *Chem. Eur. J.* **2007**, *13*, 7162-7170
13) Faiges, J.; Biosca, M.; Pericàs, M. A.; Besora, M.; Pàmies, O.; Diéguez, M. *Angew. Chem. Int. Ed.* **2024**, *63*, e202315872.

5. NMR spectra – dienones

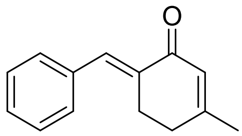

^{1}**H NMR** (400 MHz, $CDCl_3$) of compound **1a**

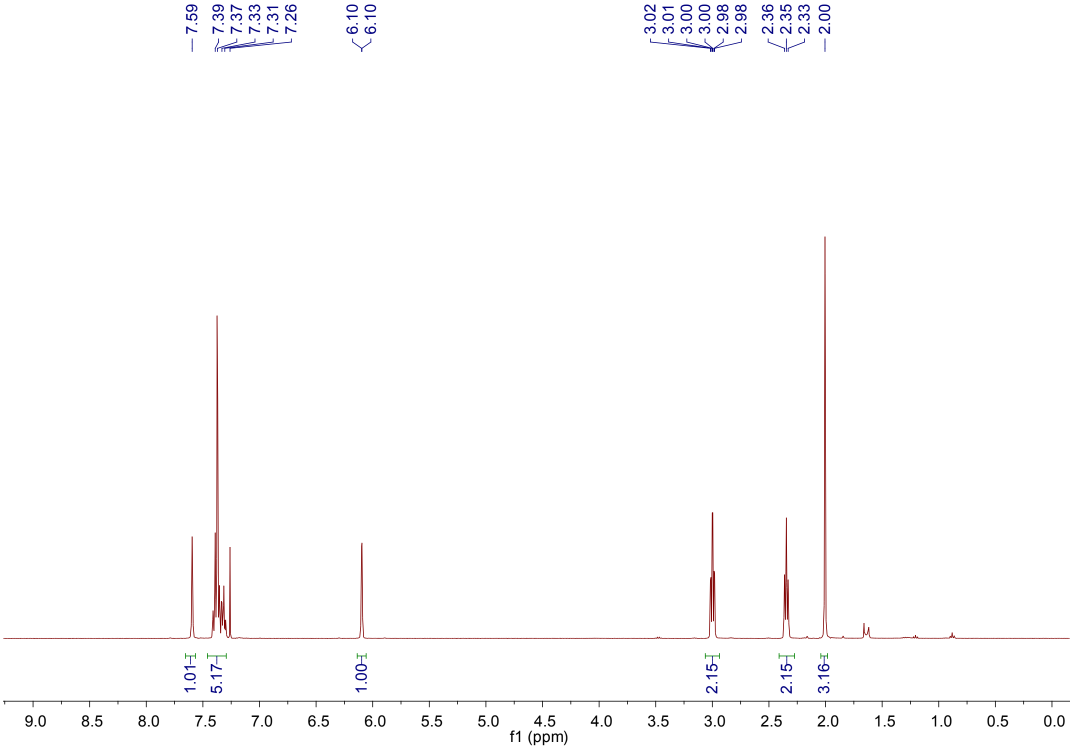

^{13}**C NMR** (100 MHz, $CDCl_3$) of compound **1a**

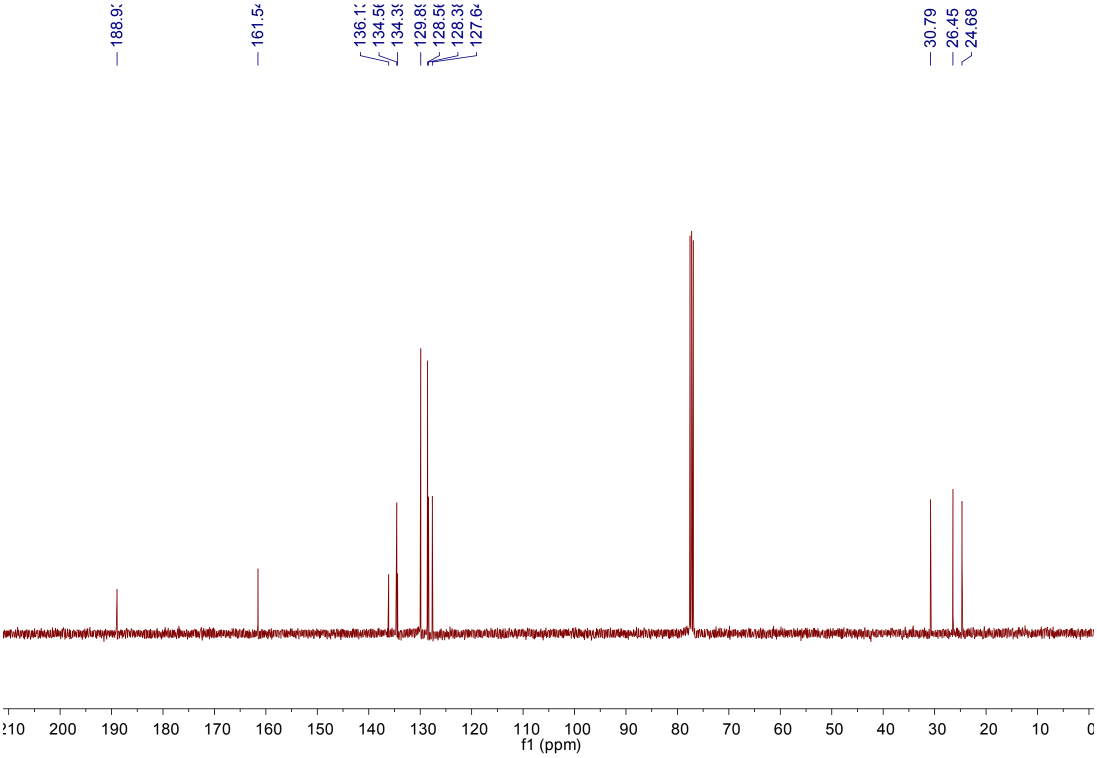

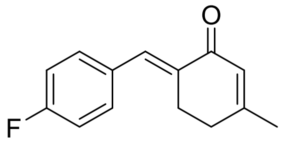

^{1}**H NMR** (400 MHz, $CDCl_3$) of compound **1b**

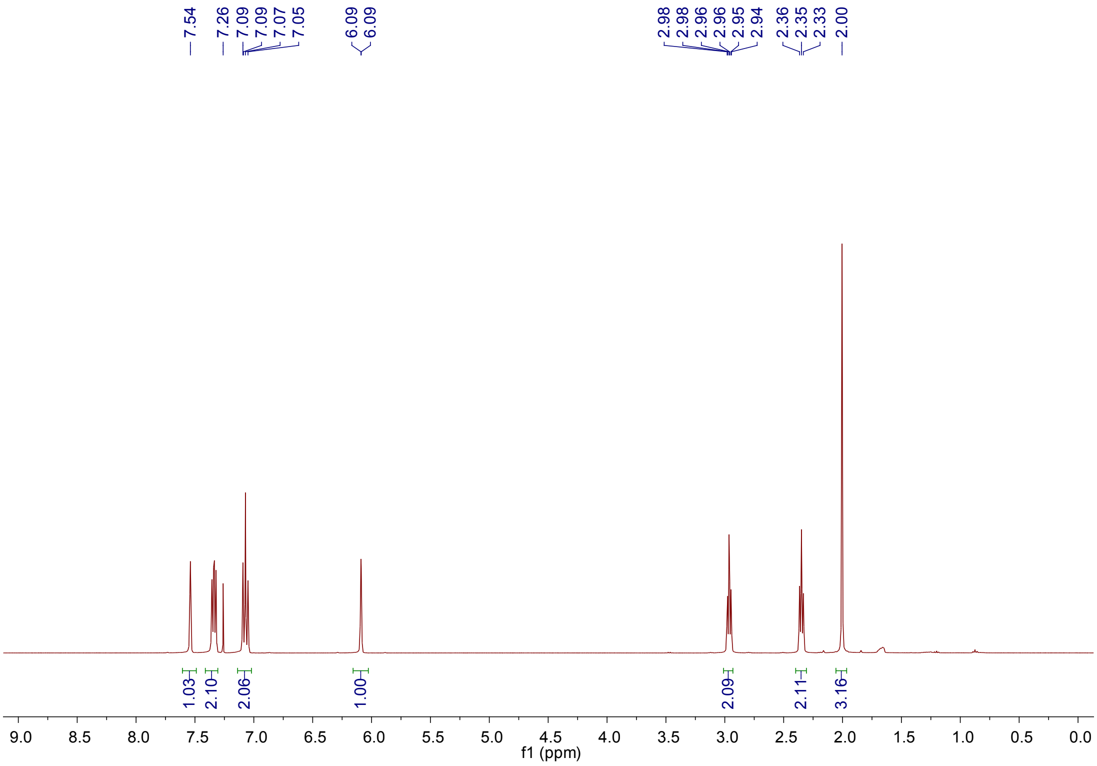

^{13}**C NMR** (100 MHz, $CDCl_3$) of compound **1b**

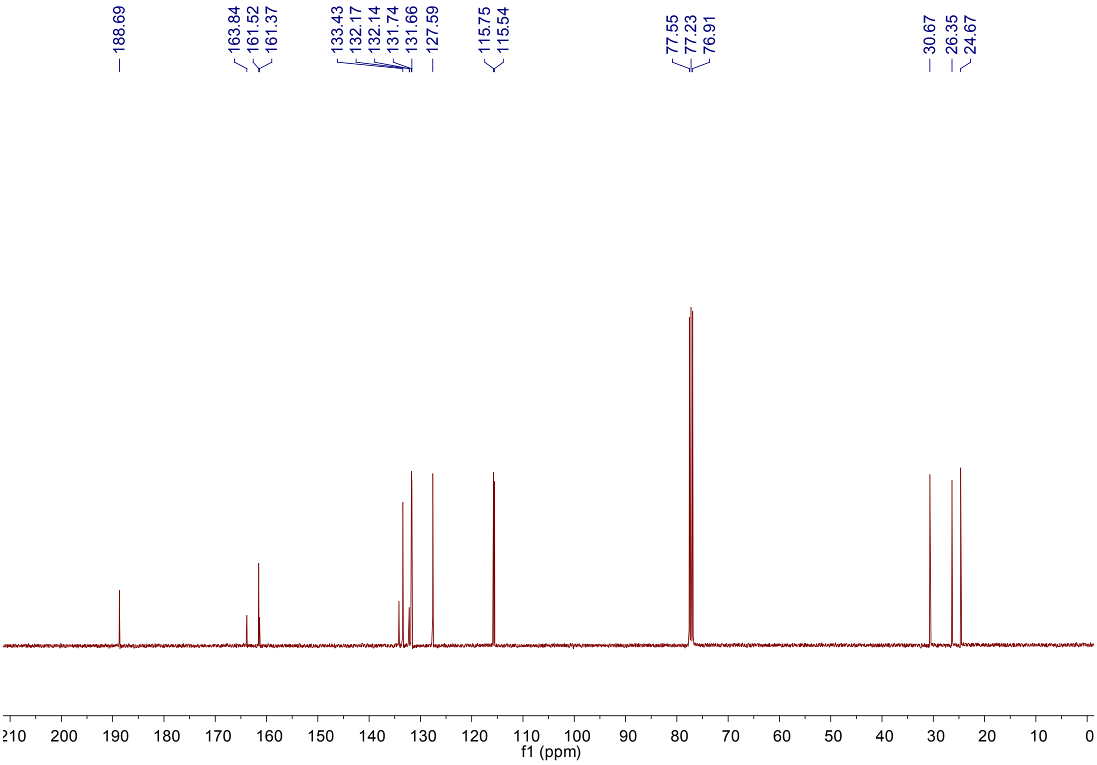

^{19}F NMR (377 MHz, $CDCl_3$) of compound **1b**

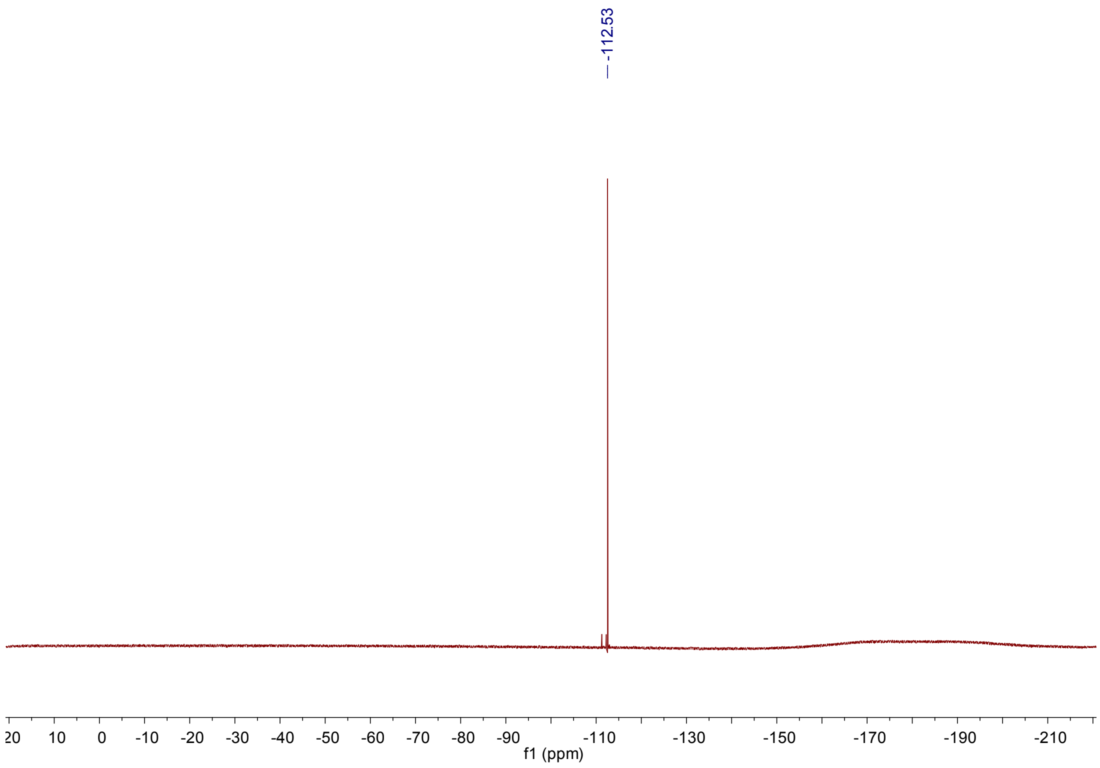

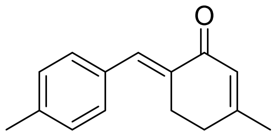

^{1}H NMR (400 MHz, $CDCl_3$) of compound **1c**

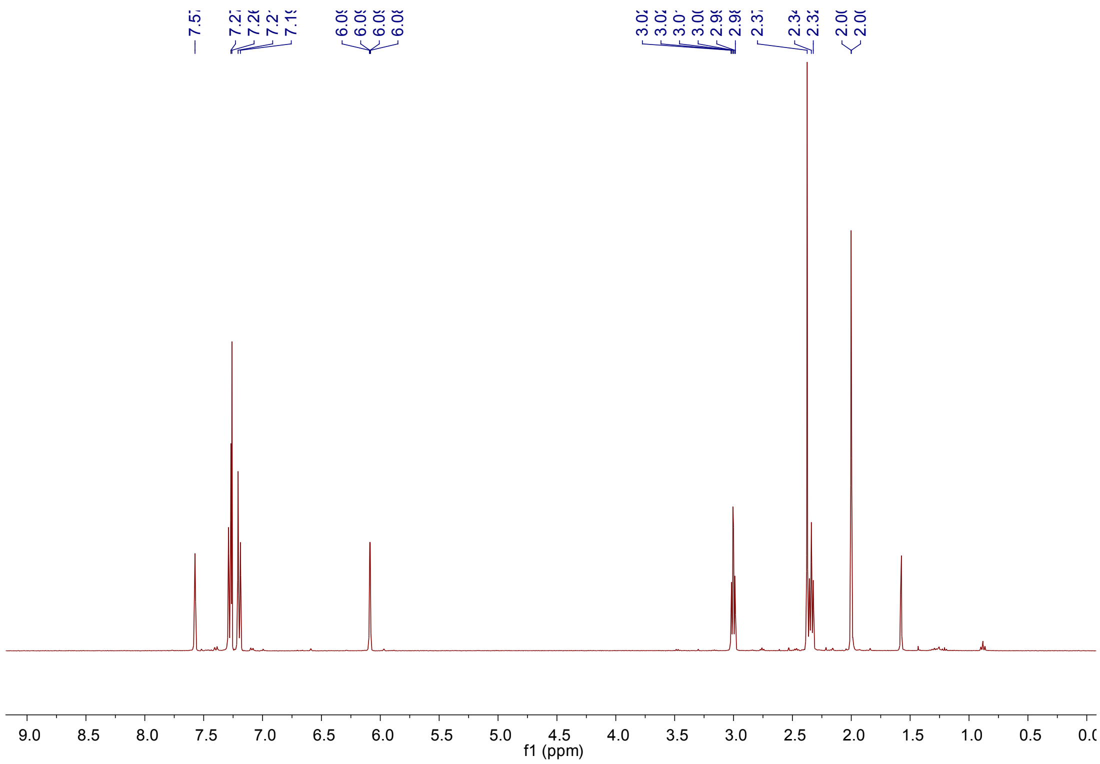

^{13}C NMR (100 MHz, $CDCl_3$) of compound **1c**

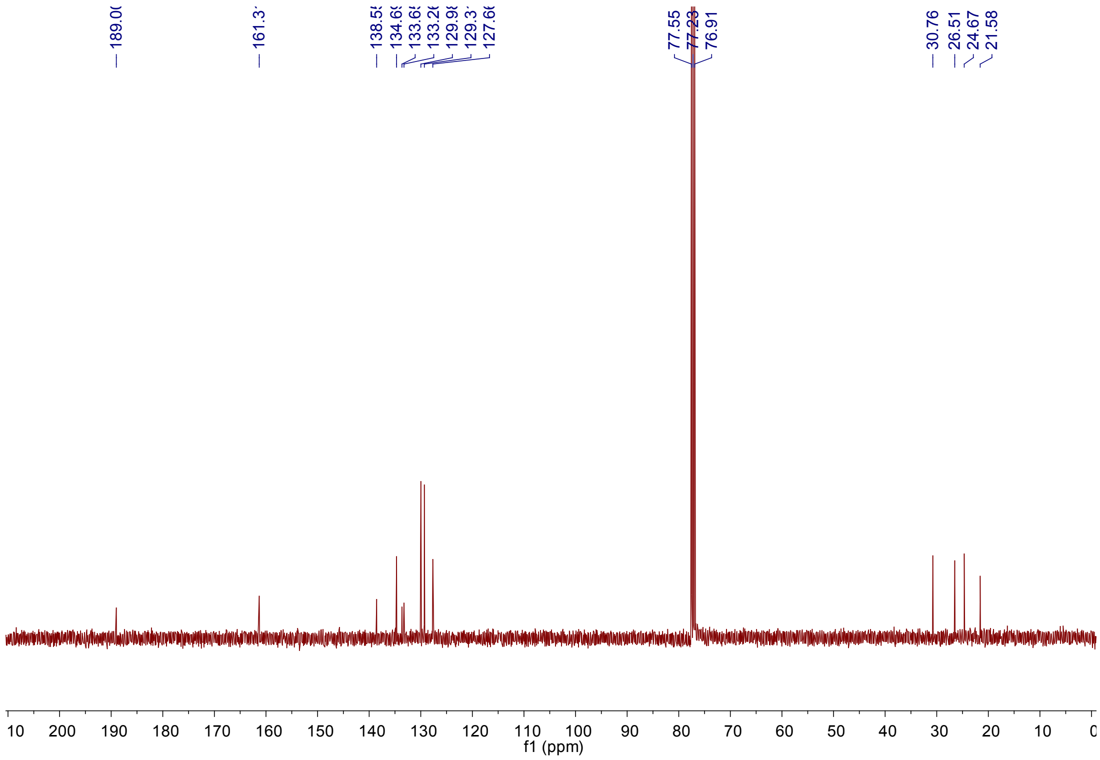

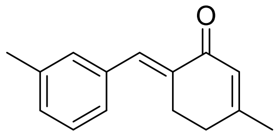

^{1}H NMR (400 MHz, $CDCl_3$) of compound **1d**

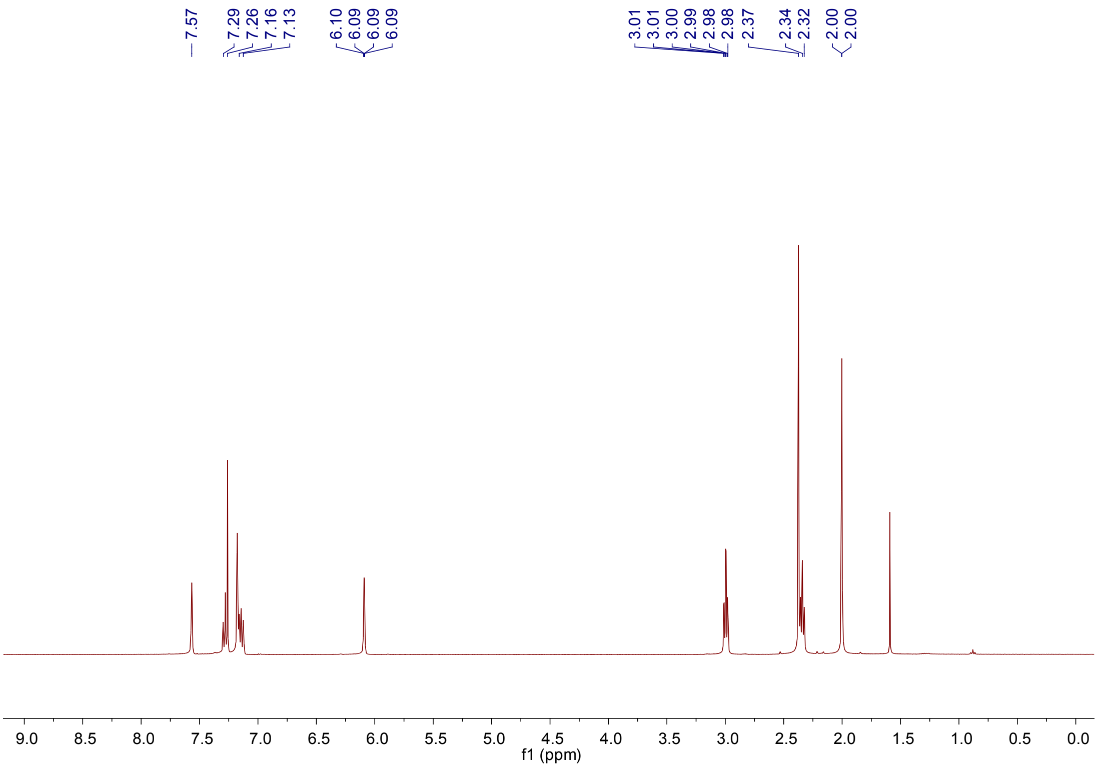

^{13}C NMR (100 MHz, $CDCl_3$) of compound **1d**

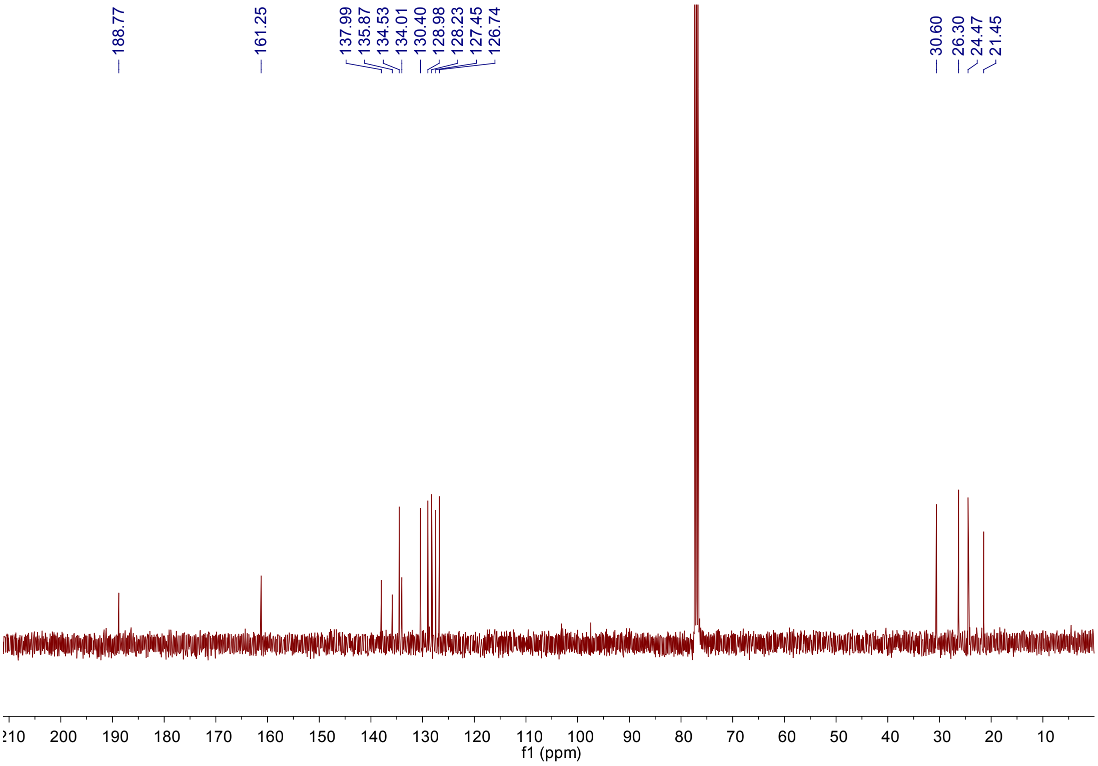

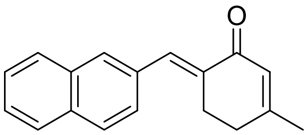

^{1}H NMR (400 MHz, $CDCl_3$) of compound **1e**

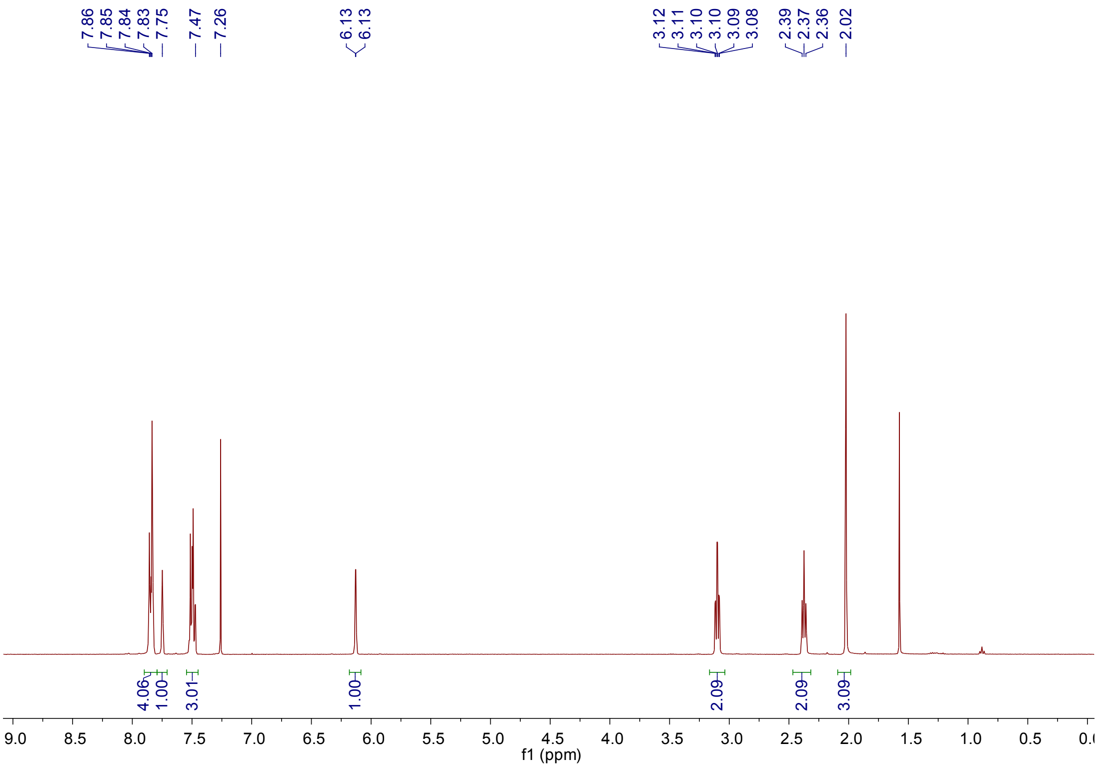

^{13}C NMR (100 MHz, $CDCl_3$) of compound **1e**

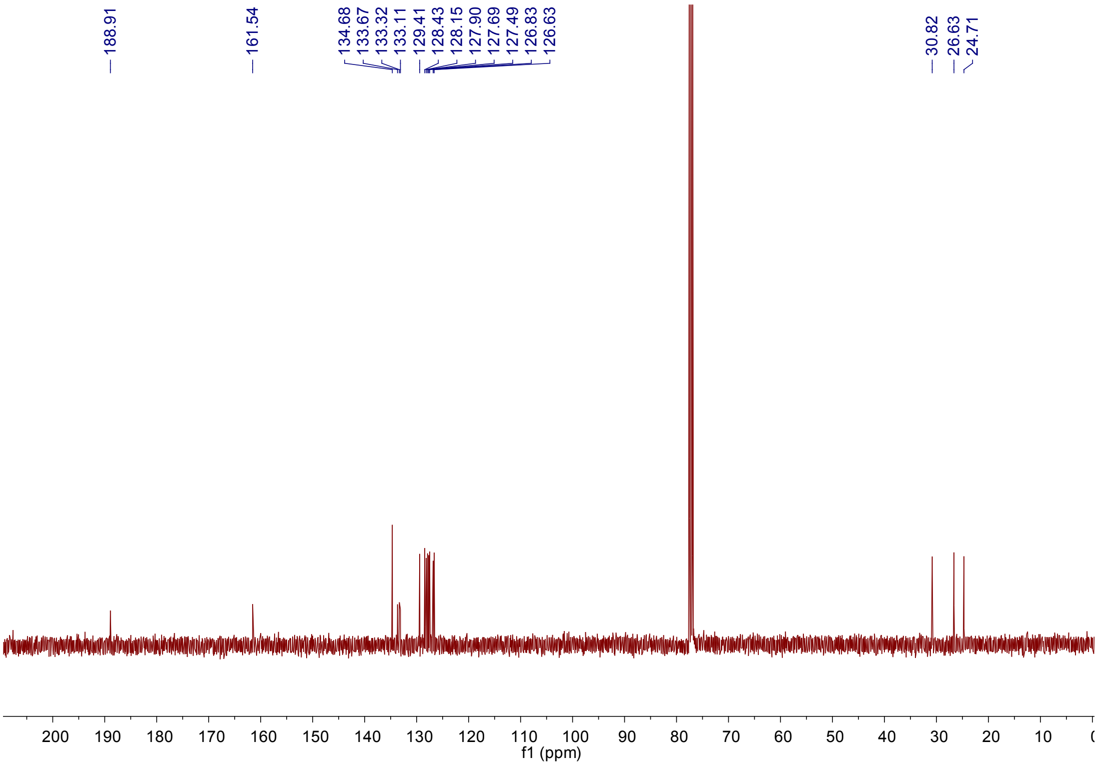

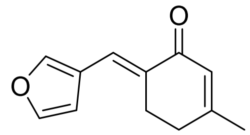

¹H NMR (400 MHz, $CDCl_3$) of compound **1f**

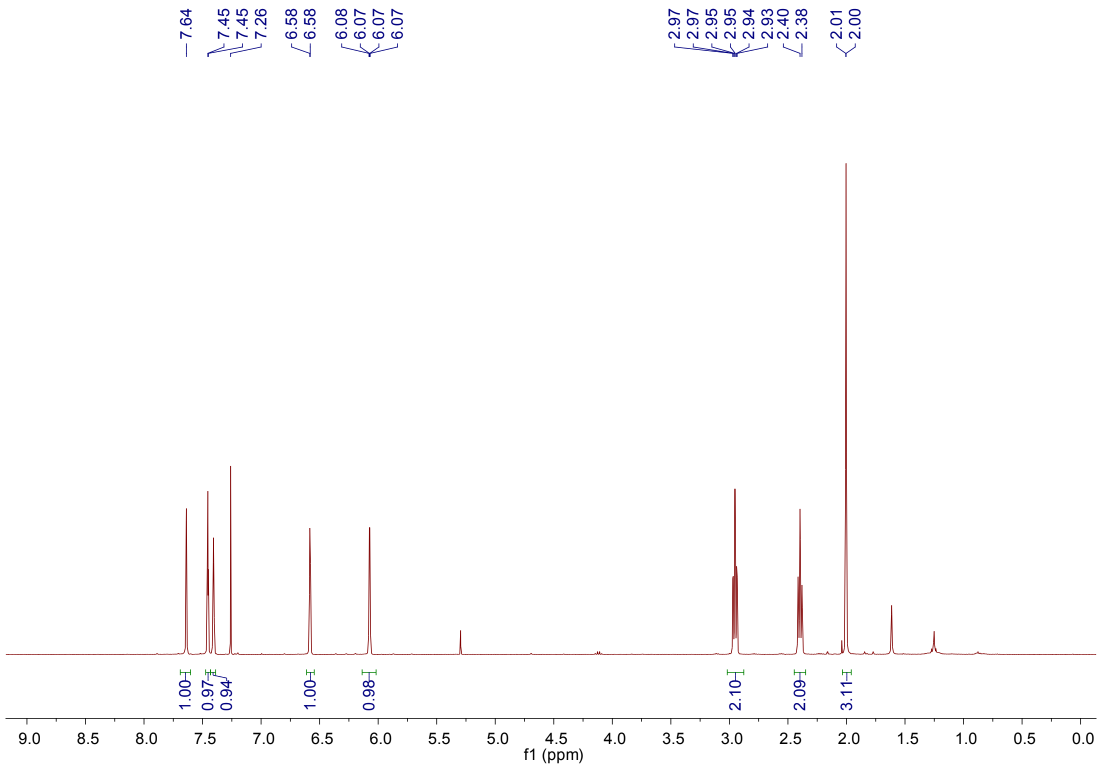

¹³C NMR (100 MHz, $CDCl_3$) of compound **1f**

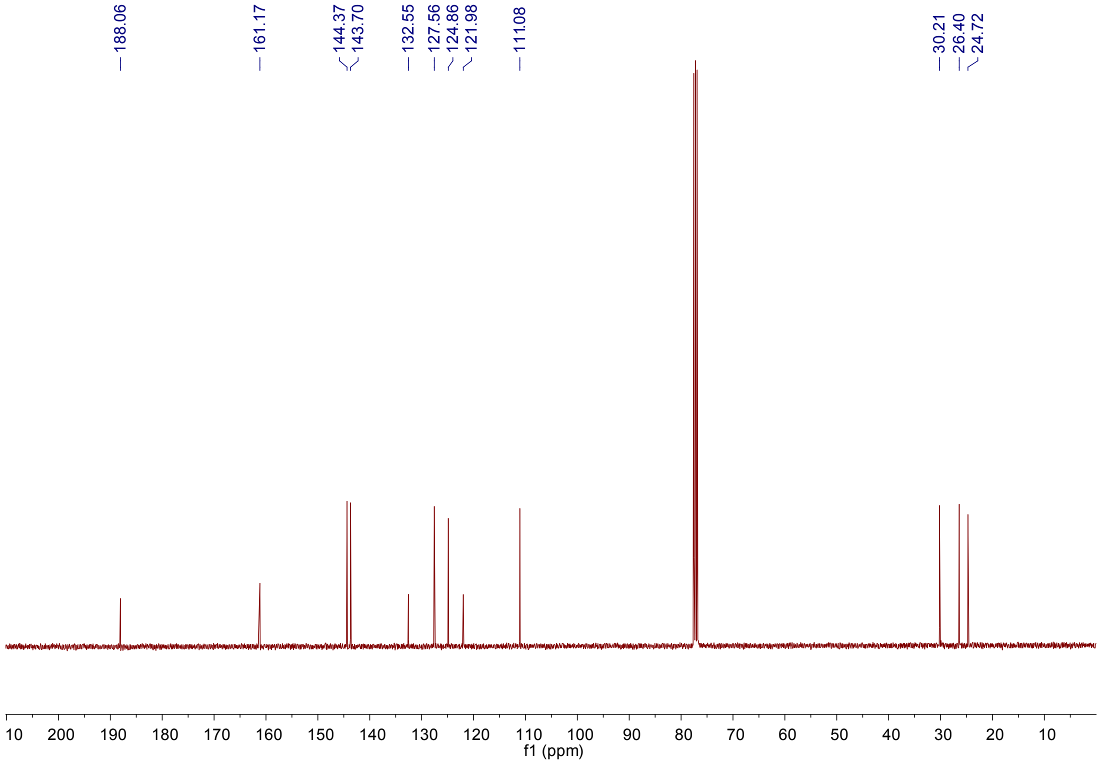

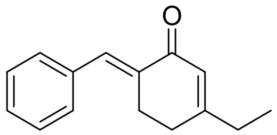

^{1}H NMR (400 MHz, $CDCl_3$) of compound **1g**

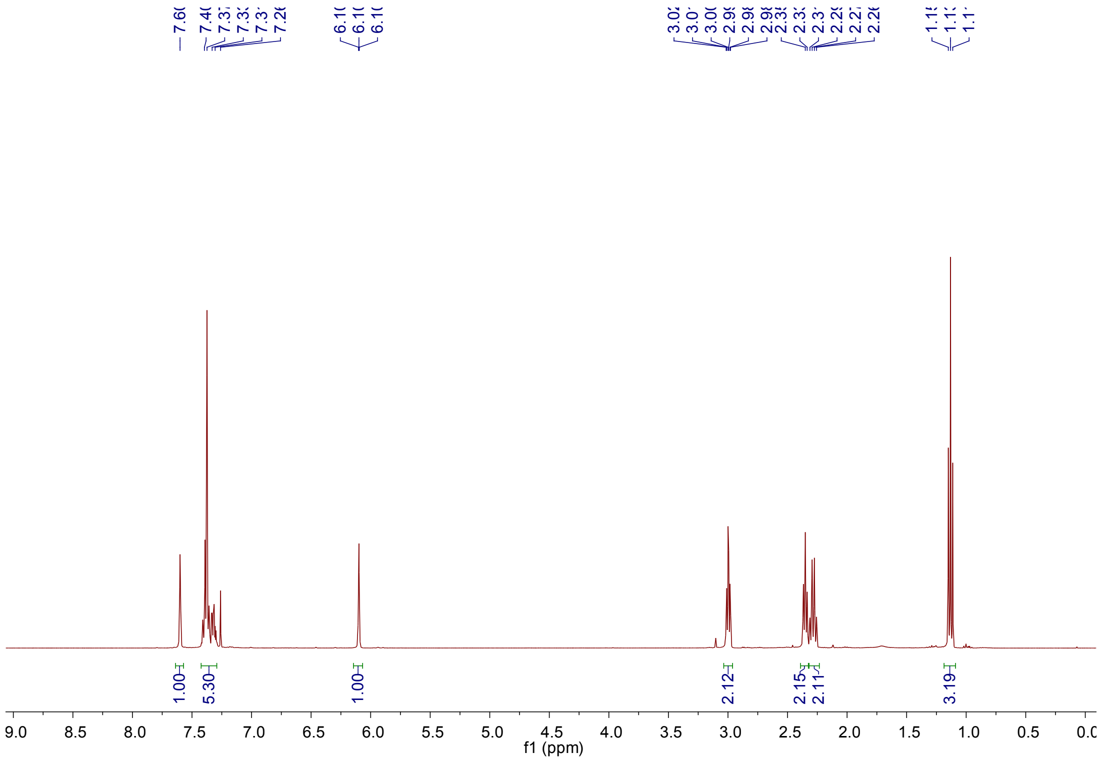

^{13}C NMR (100 MHz, $CDCl_3$) of compound **1g**

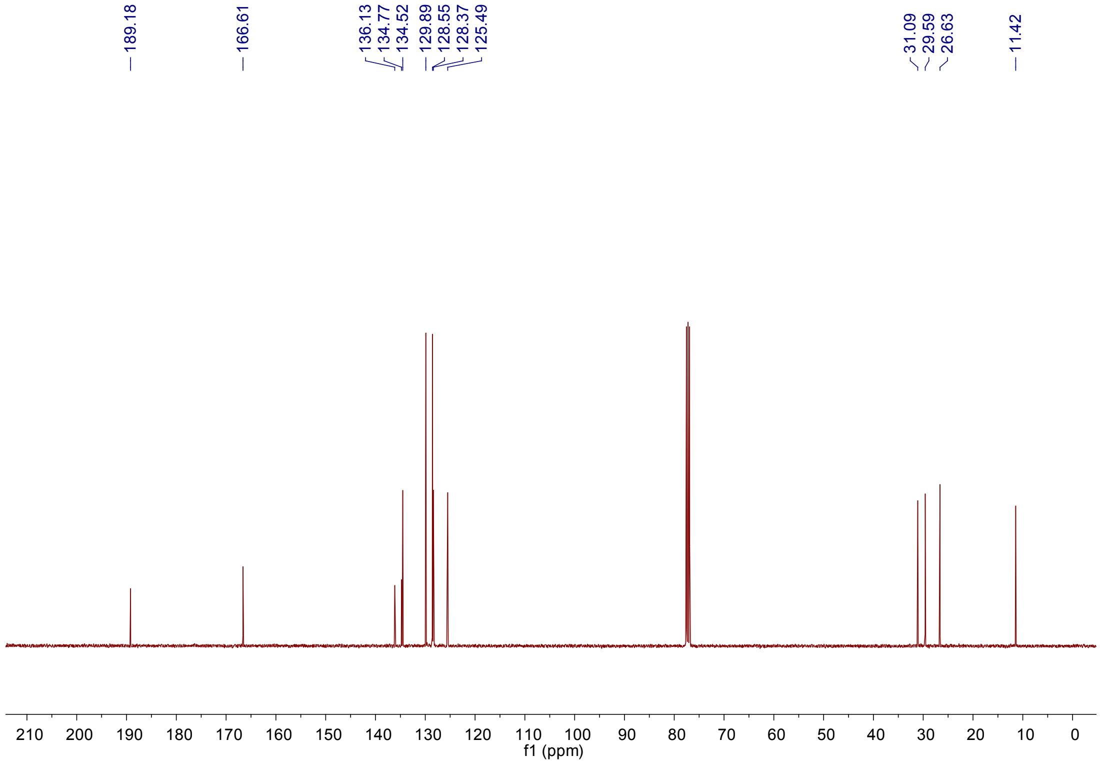

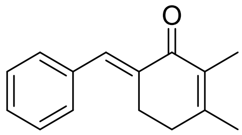

^{1}H NMR (400 MHz, $CDCl_3$) of compound **1h**

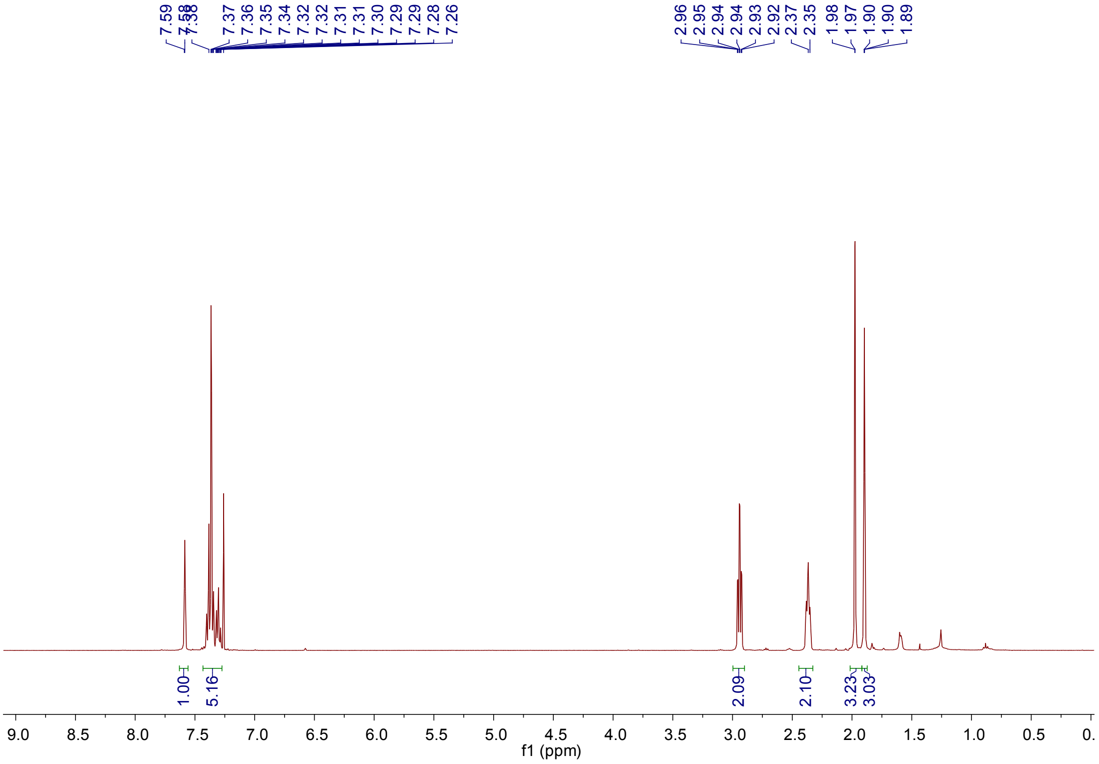

^{13}C NMR (100 MHz, $CDCl_3$) of compound **1h**

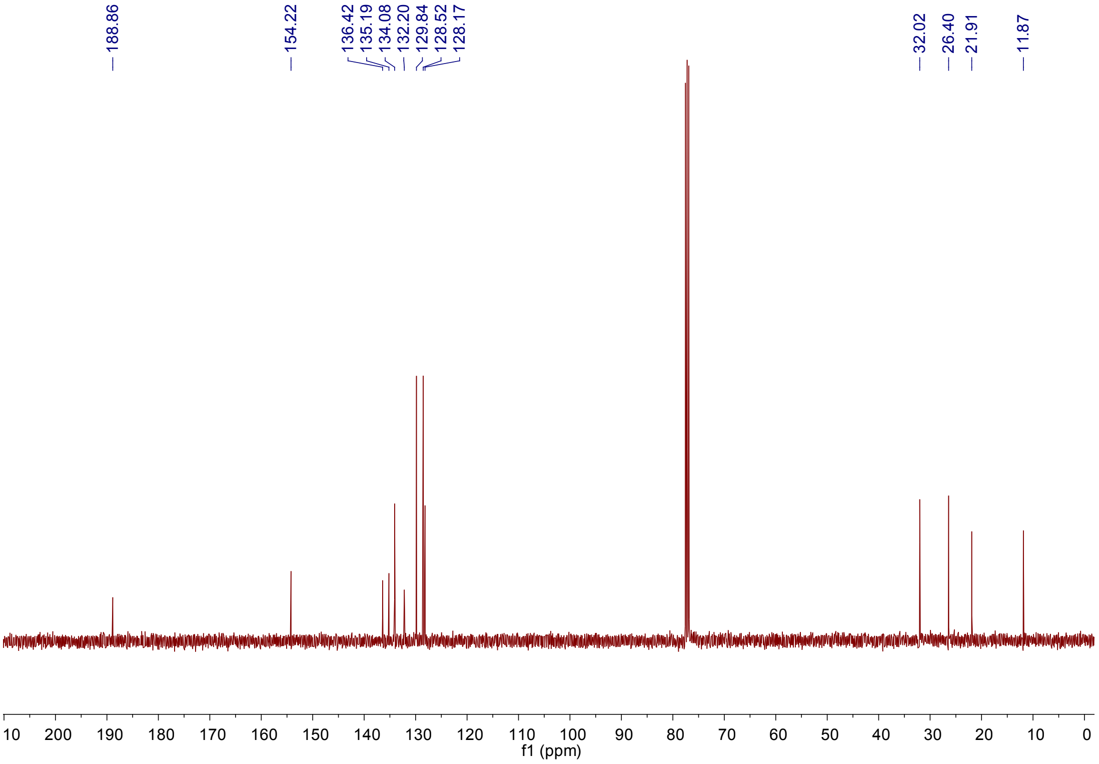

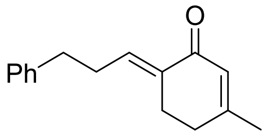

^{1}**H NMR** (400 MHz, $CDCl_3$) of compound **1i**

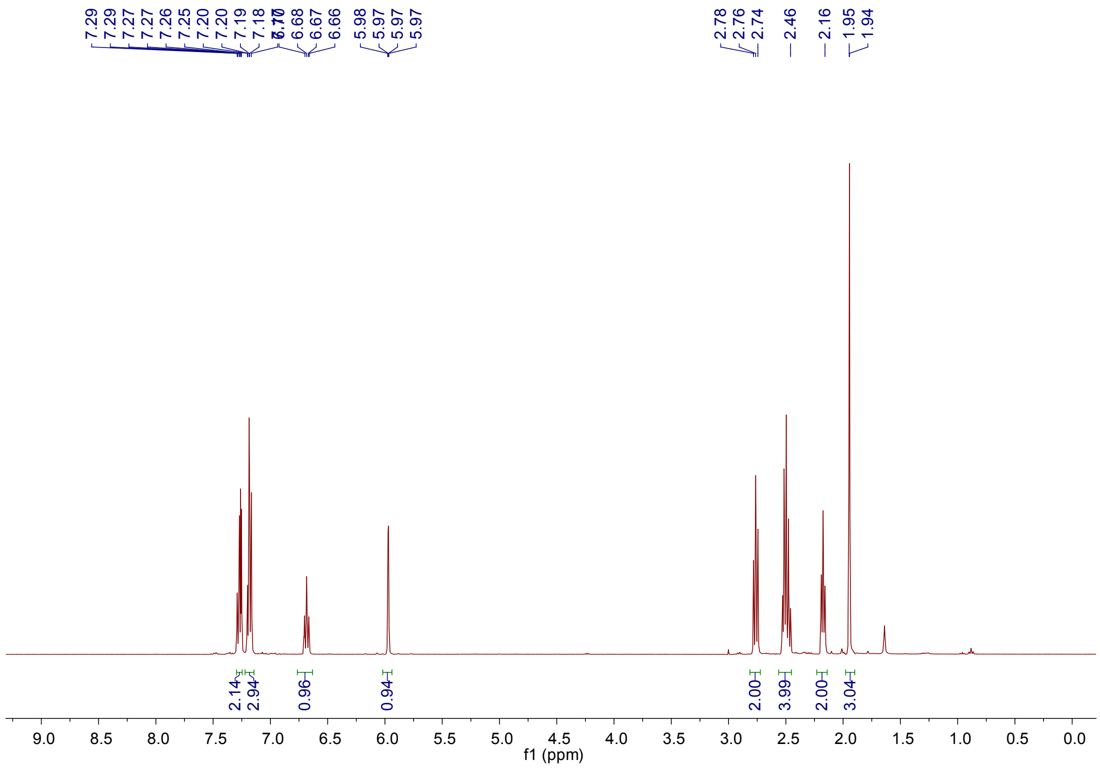

^{13}**C NMR** (100 MHz, $CDCl_3$) of compound **1i**

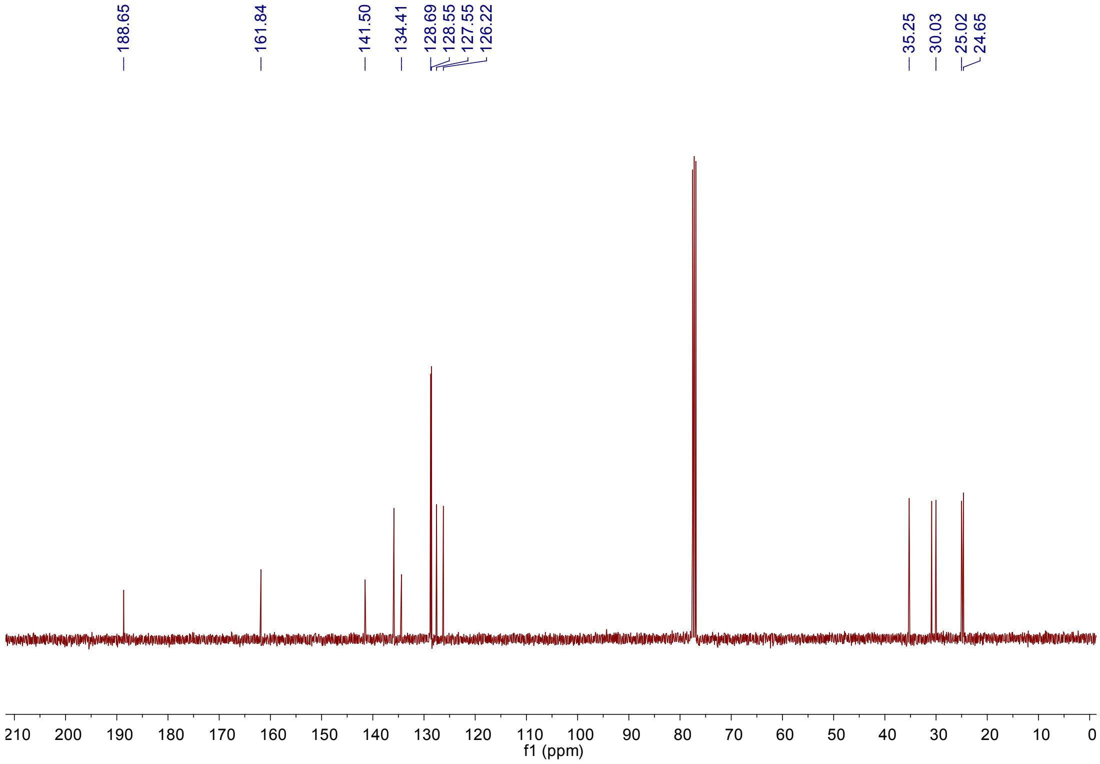

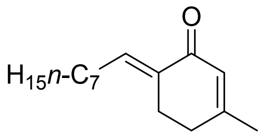

¹H NMR (400 MHz, $CDCl_3$) of compound **1j**

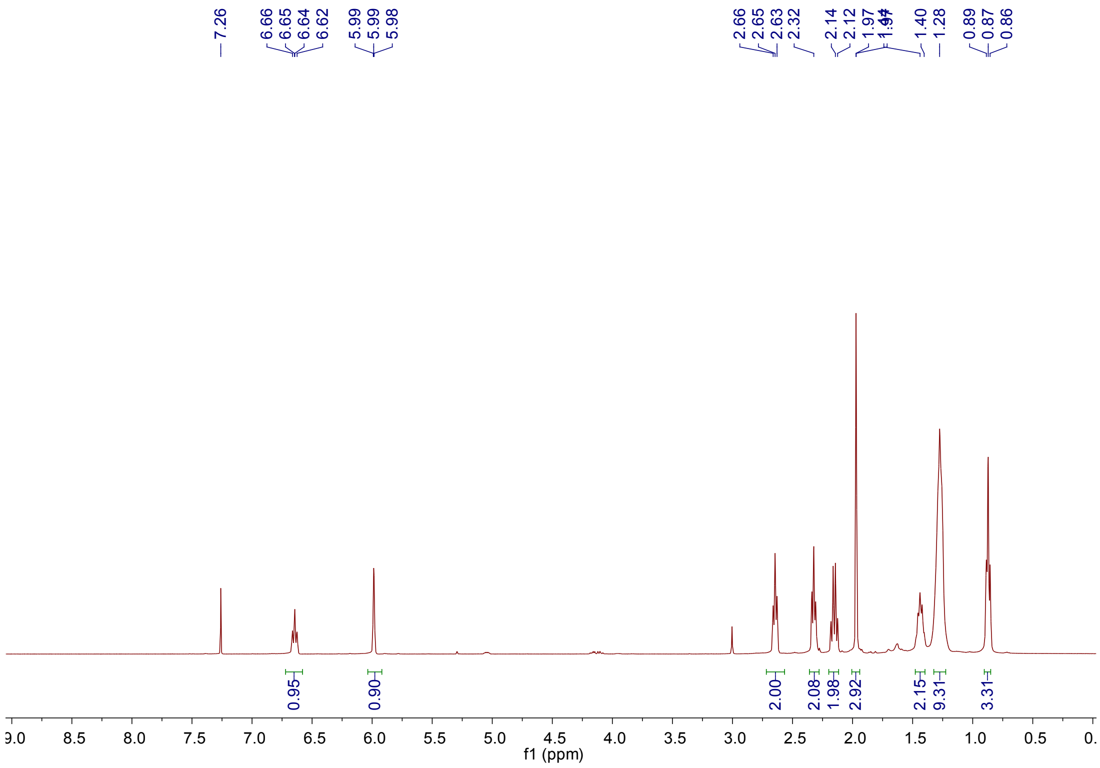

¹³C NMR (100 MHz, $CDCl_3$) of compound **1j**

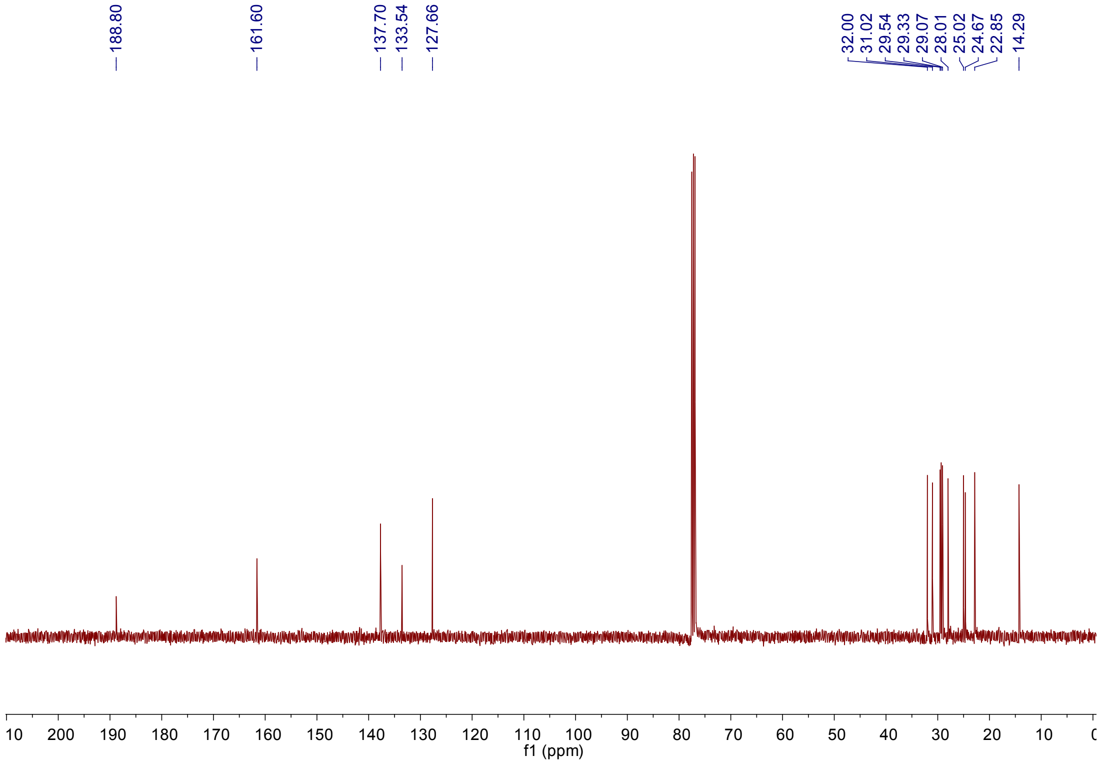

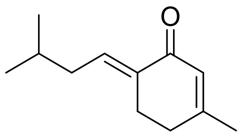

^{1}H NMR (400 MHz, $CDCl_3$) of compound **1k**

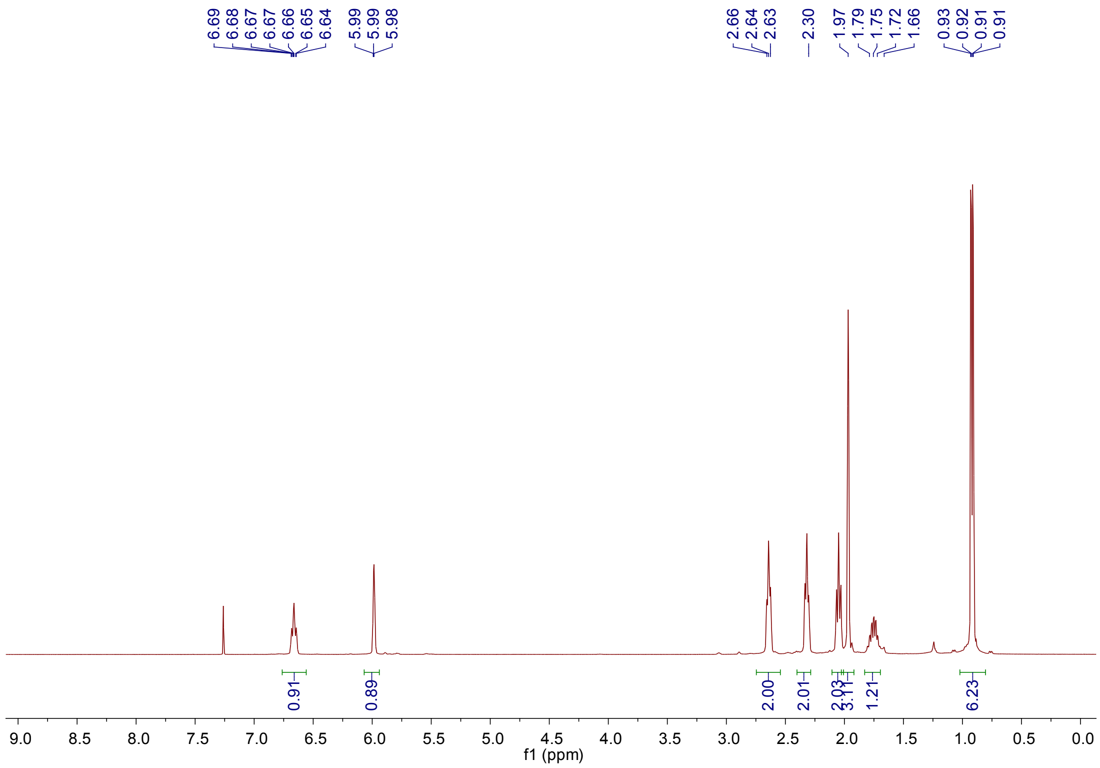

^{13}C NMR (100 MHz, $CDCl_3$) of compound **1k**

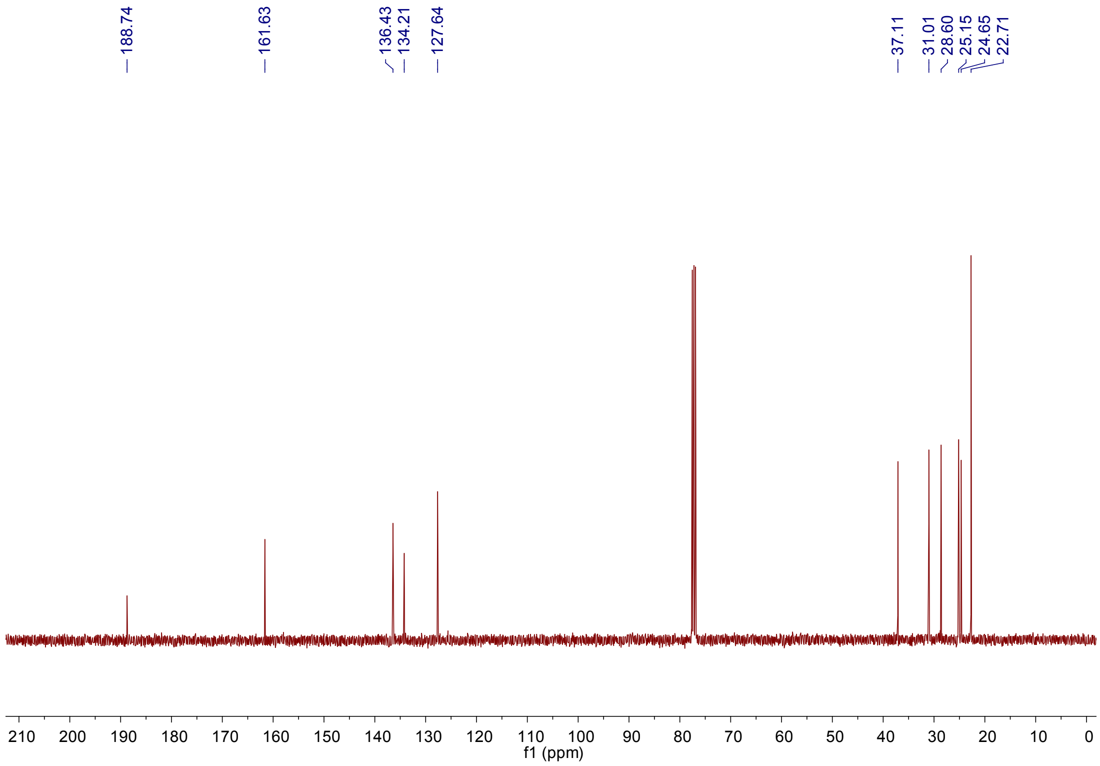

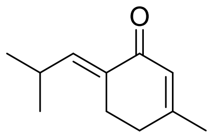

^{1}**H NMR** (400 MHz, $CDCl_3$) of compound **1l**

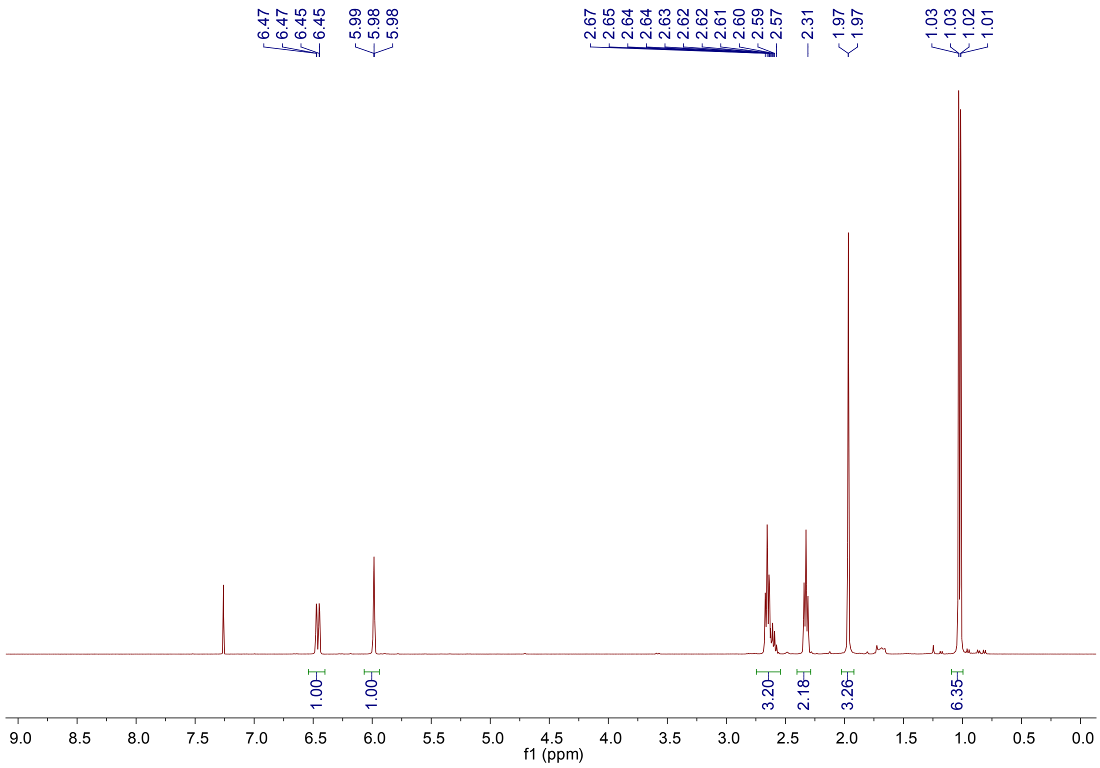

^{13}**C NMR** (100 MHz, $CDCl_3$) of compound **1l**

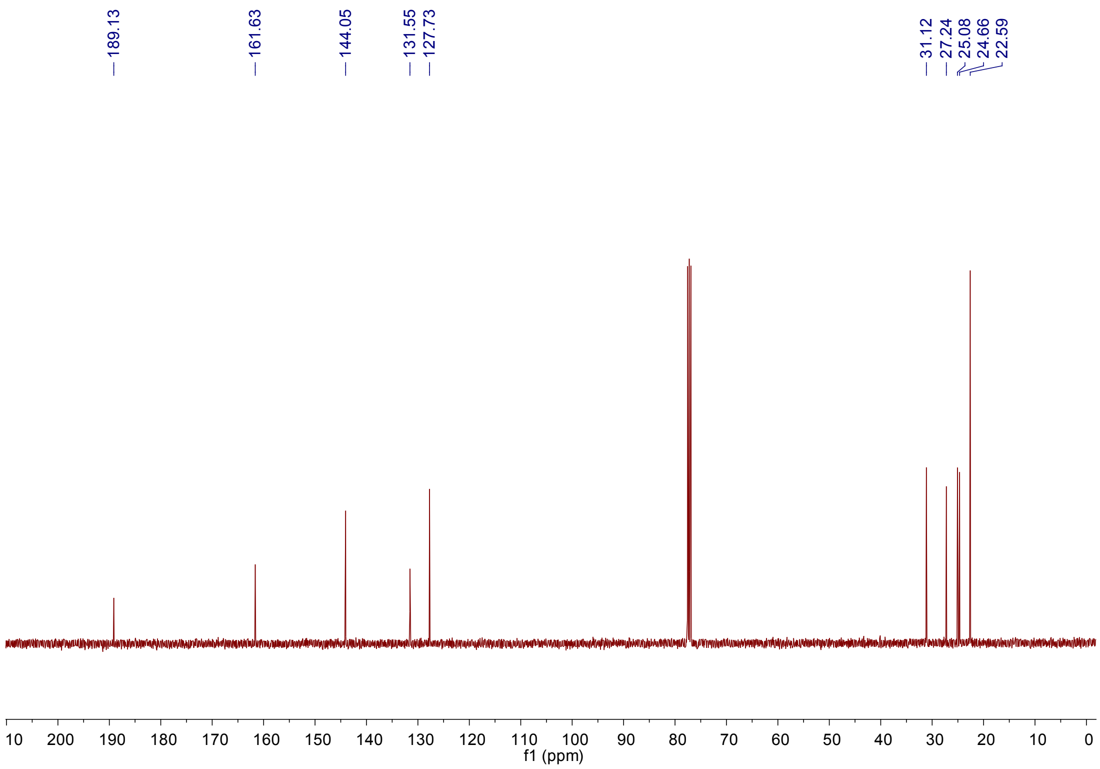

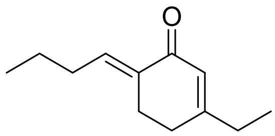

¹H NMR (400 MHz, $CDCl_3$) of compound **1m**

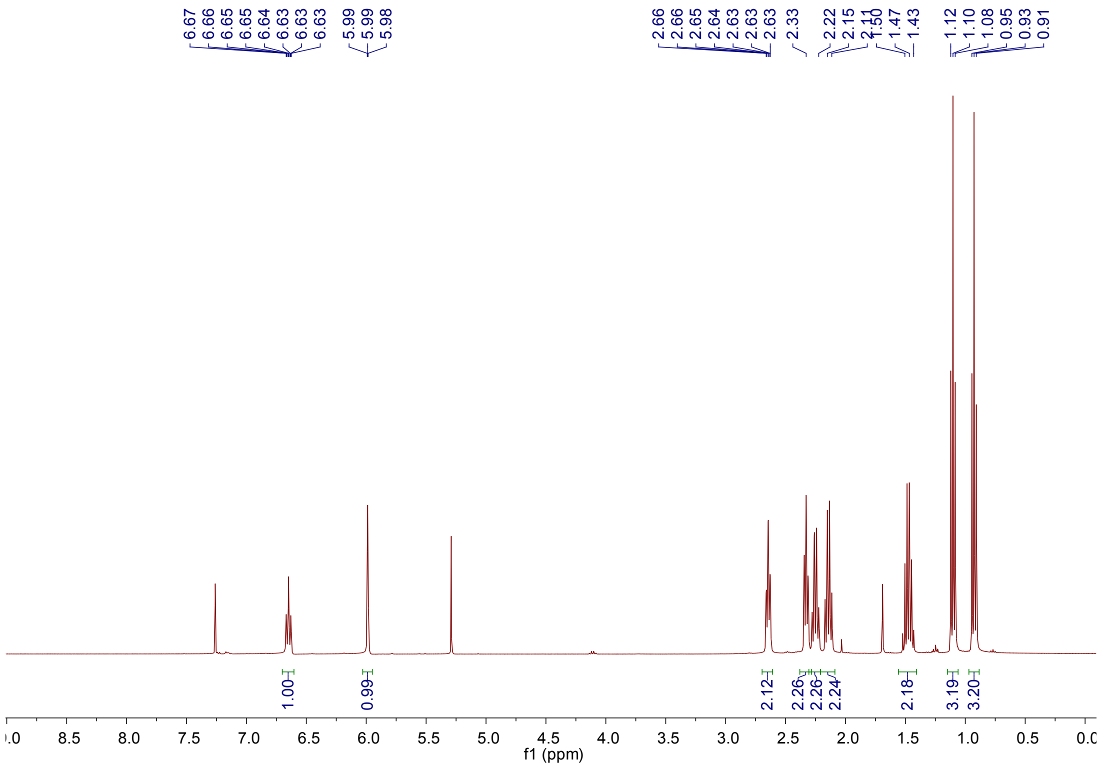

¹³C NMR (100 MHz, $CDCl_3$) of compound **1m**

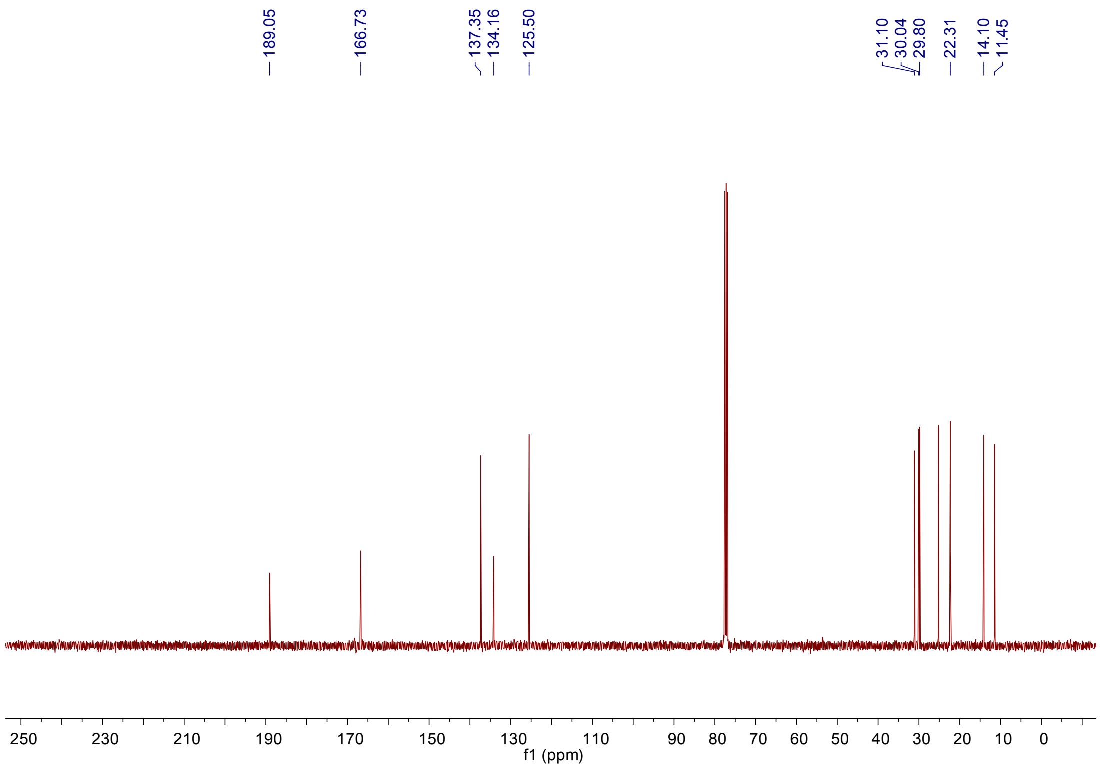

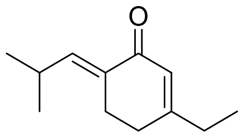

^{1}H NMR (400 MHz, $CDCl_3$) of compound **1p**

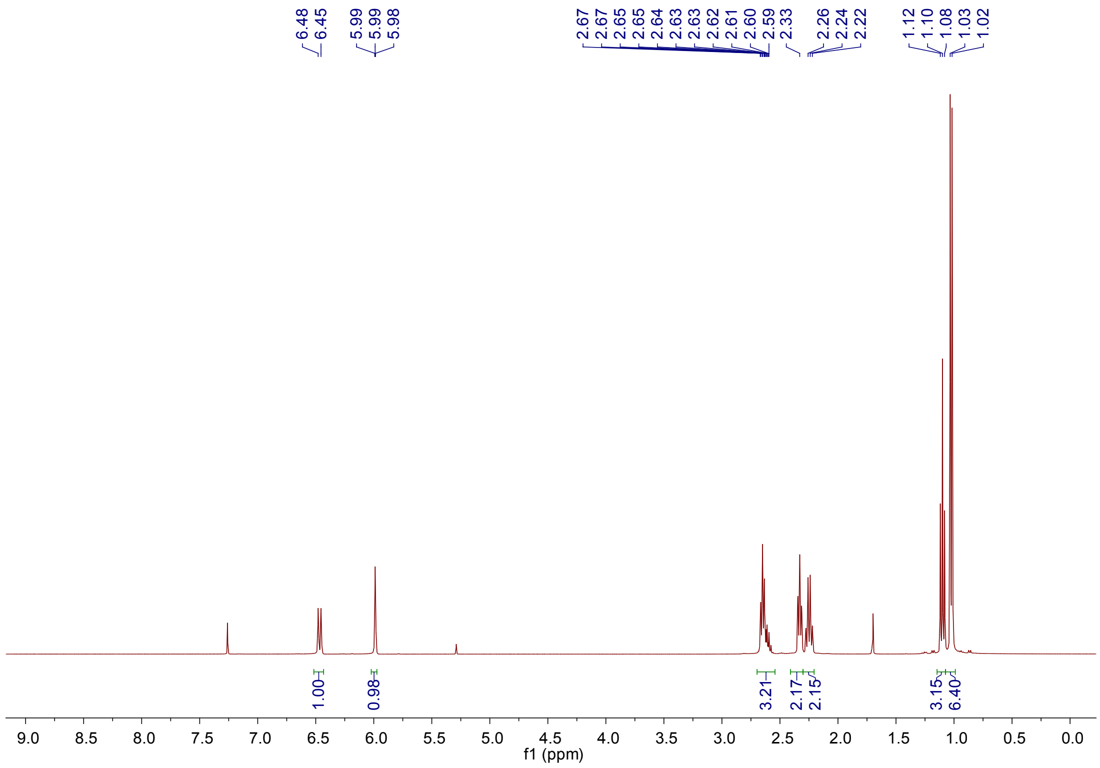

^{13}C NMR (100 MHz, $CDCl_3$) of compound **1p**

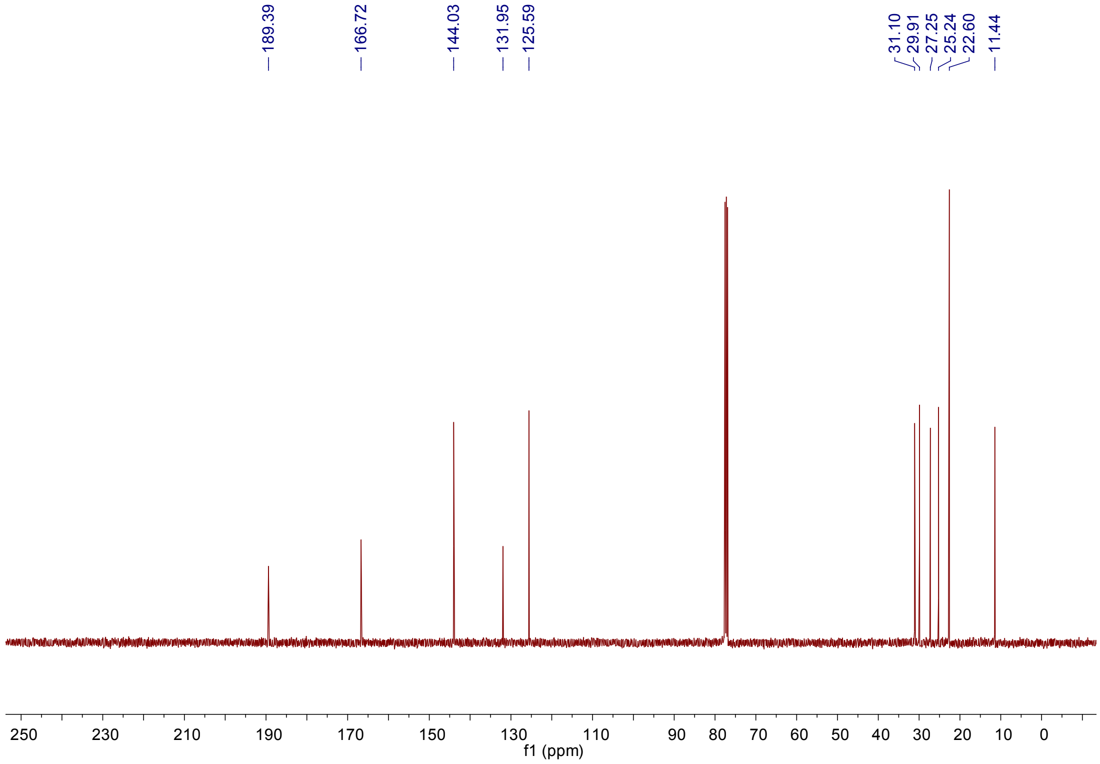

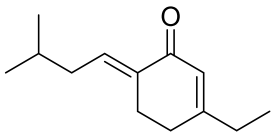

^{1}H NMR (400 MHz, $CDCl_3$) of compound **1q**

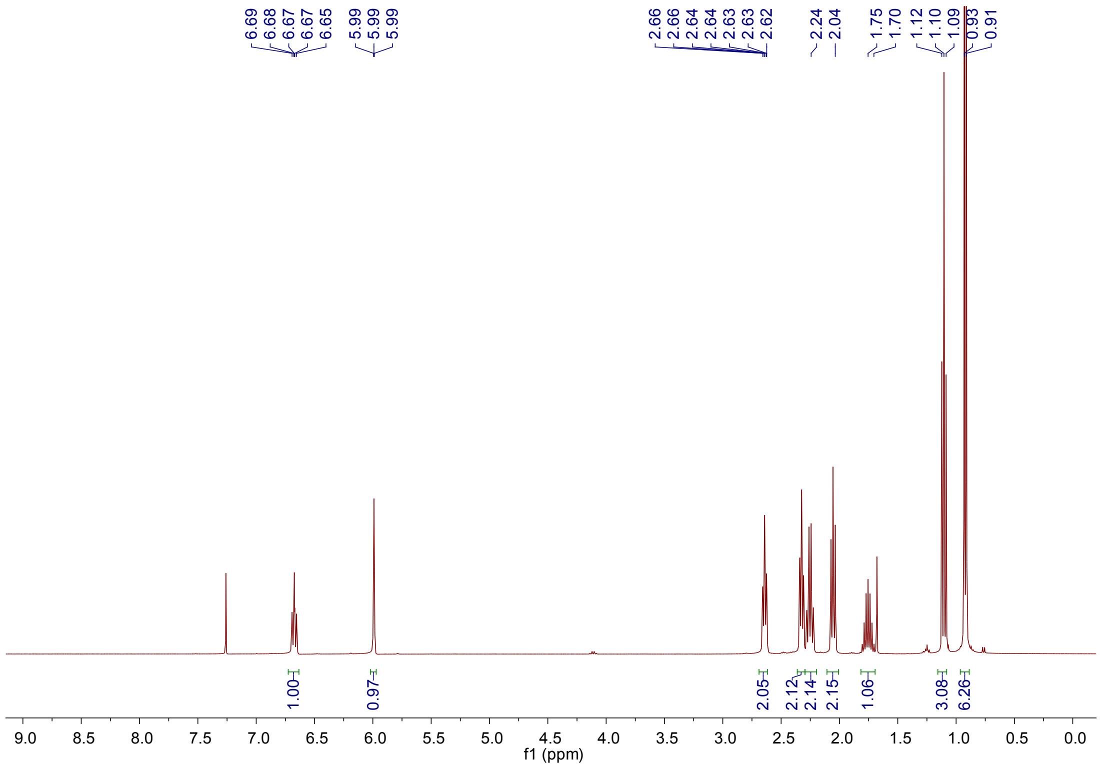

^{13}C NMR (100 MHz, $CDCl_3$) of compound **1q**

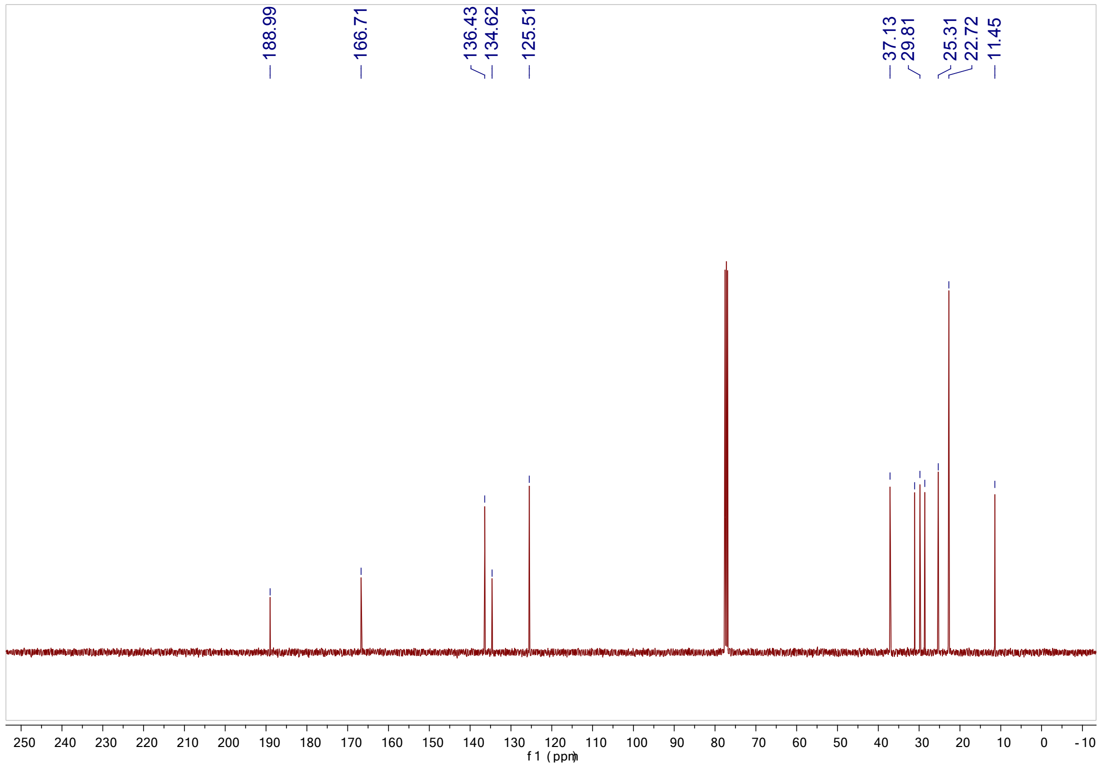

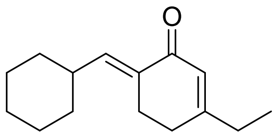

^{1}H NMR (400 MHz, $CDCl_3$) of compound **1r**

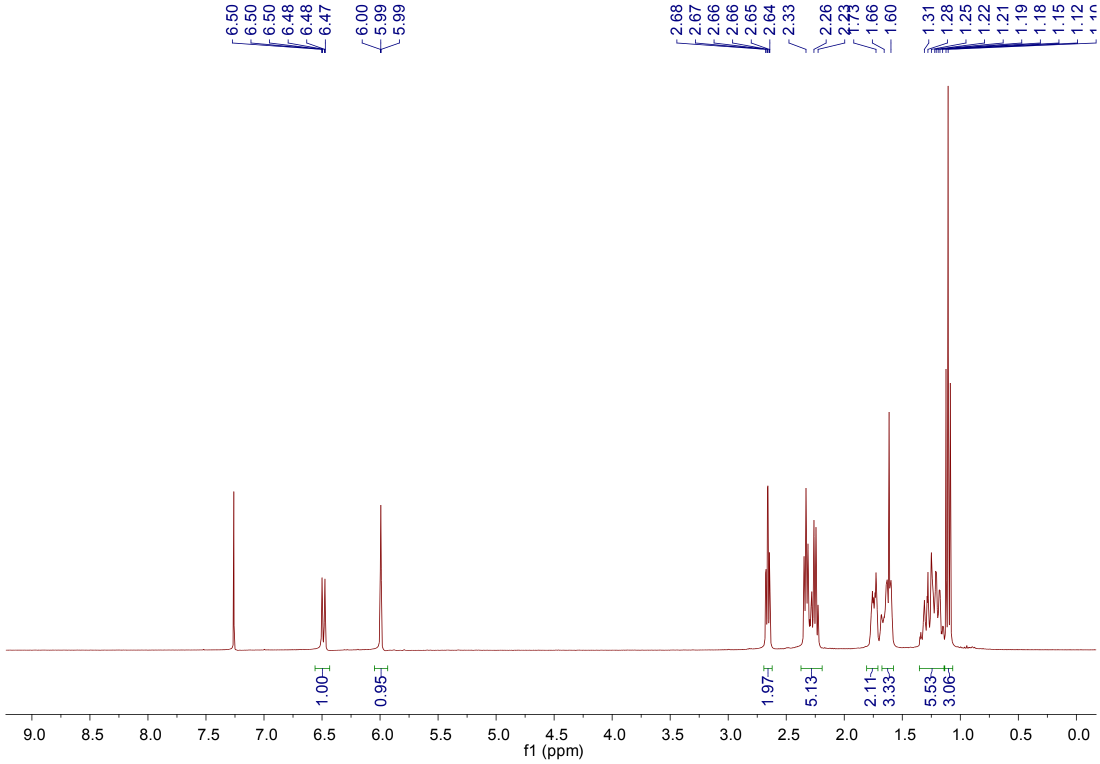

^{13}C NMR (100 MHz, $CDCl_3$) of compound **1r**

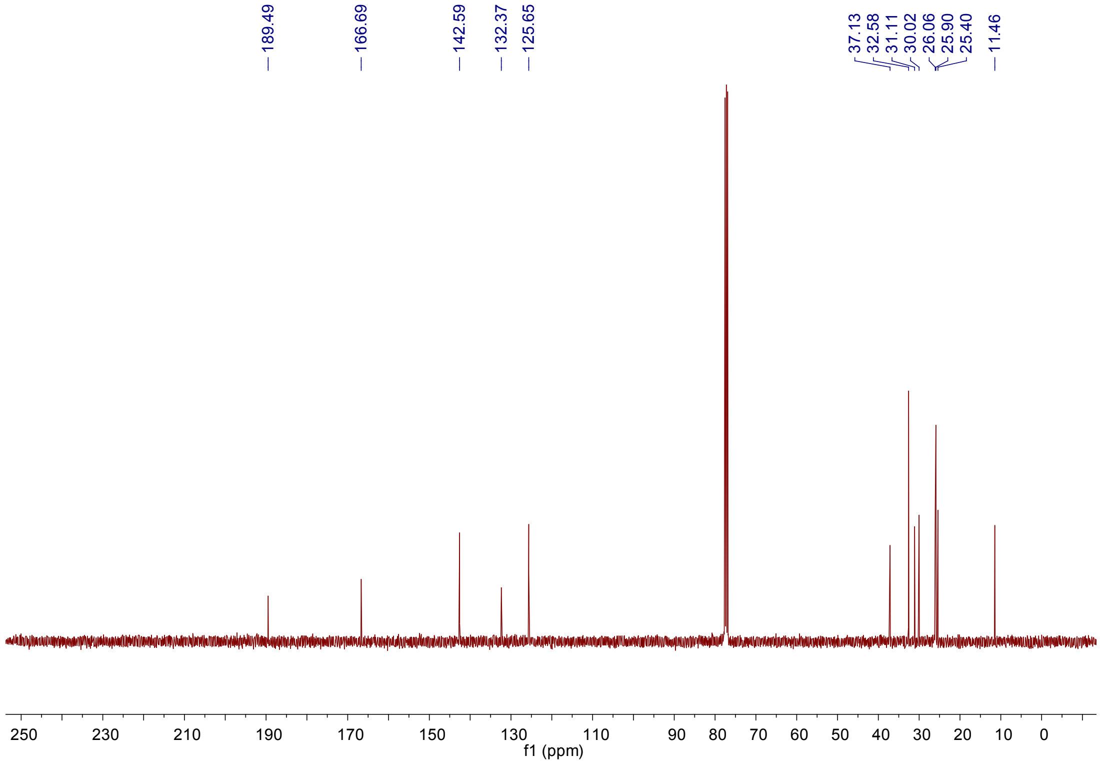

6. NMR spectra – hydrogenated products

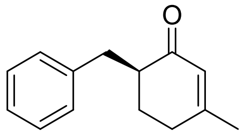

¹H NMR (400 MHz, $CDCl_3$) of compound **2a**

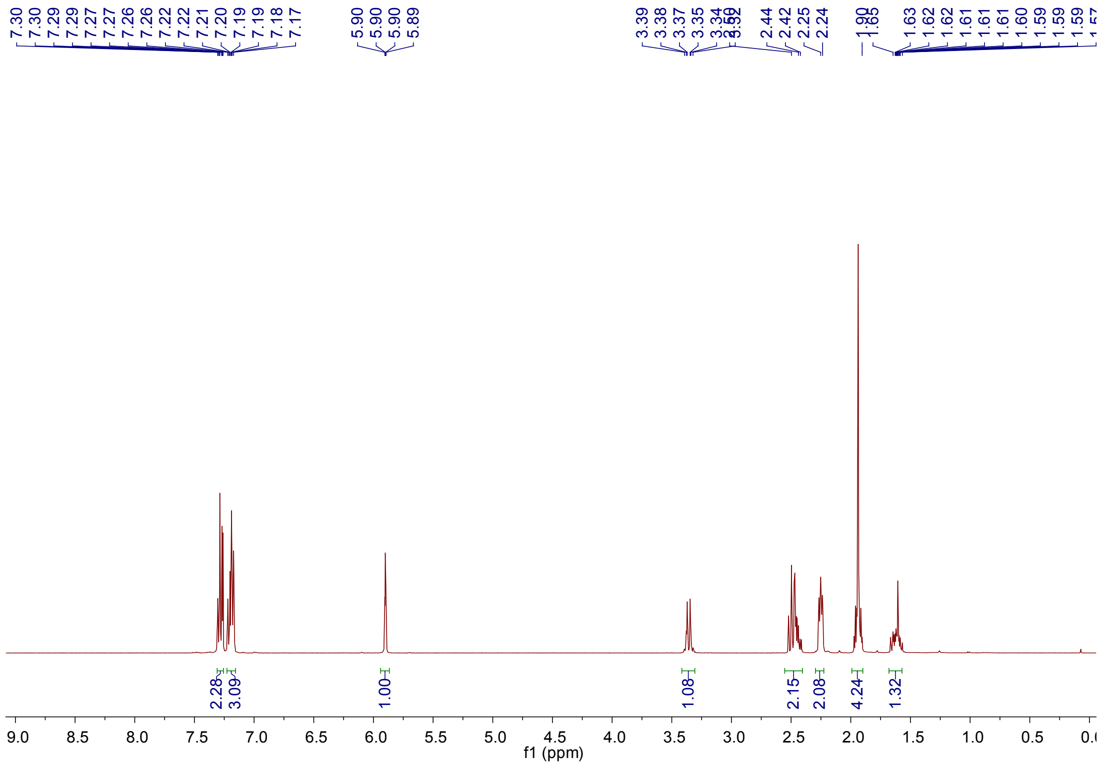

¹³C NMR (100 MHz, $CDCl_3$) of compound **2a**

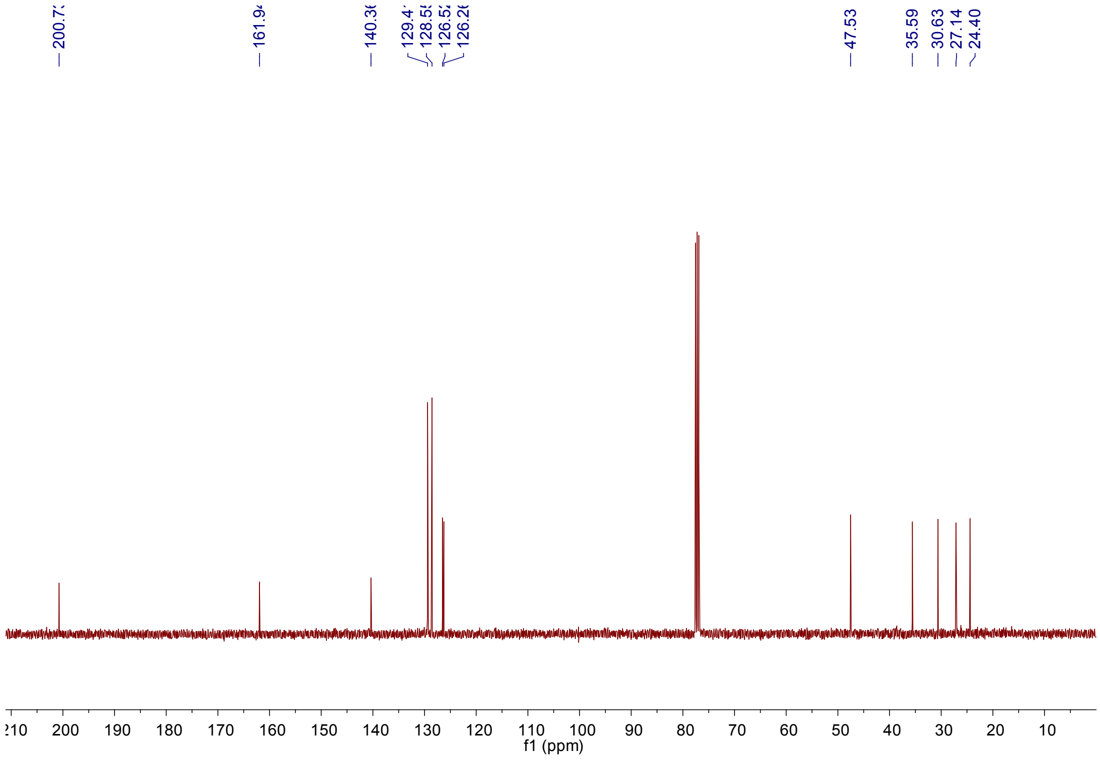

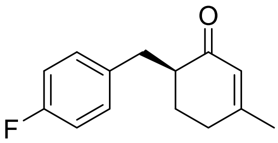

^{1}H NMR (400 MHz, $CDCl_3$) of compound **2b**

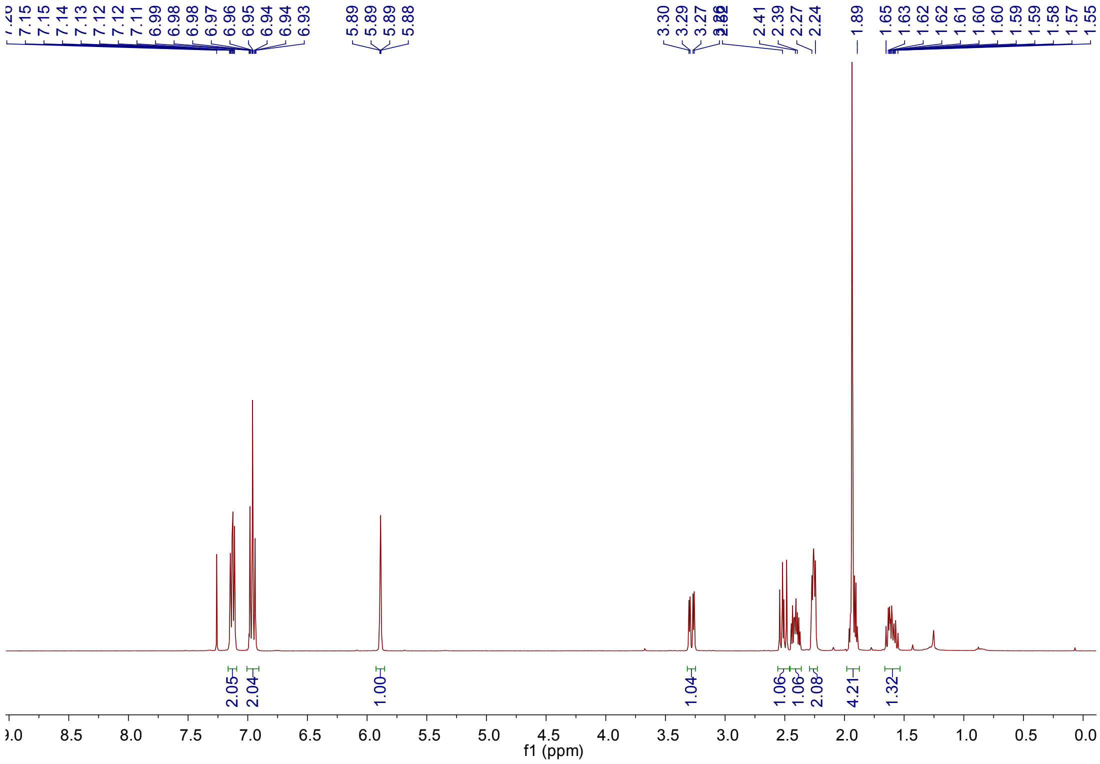

^{13}C NMR (100 MHz, $CDCl_3$) of compound **2b**

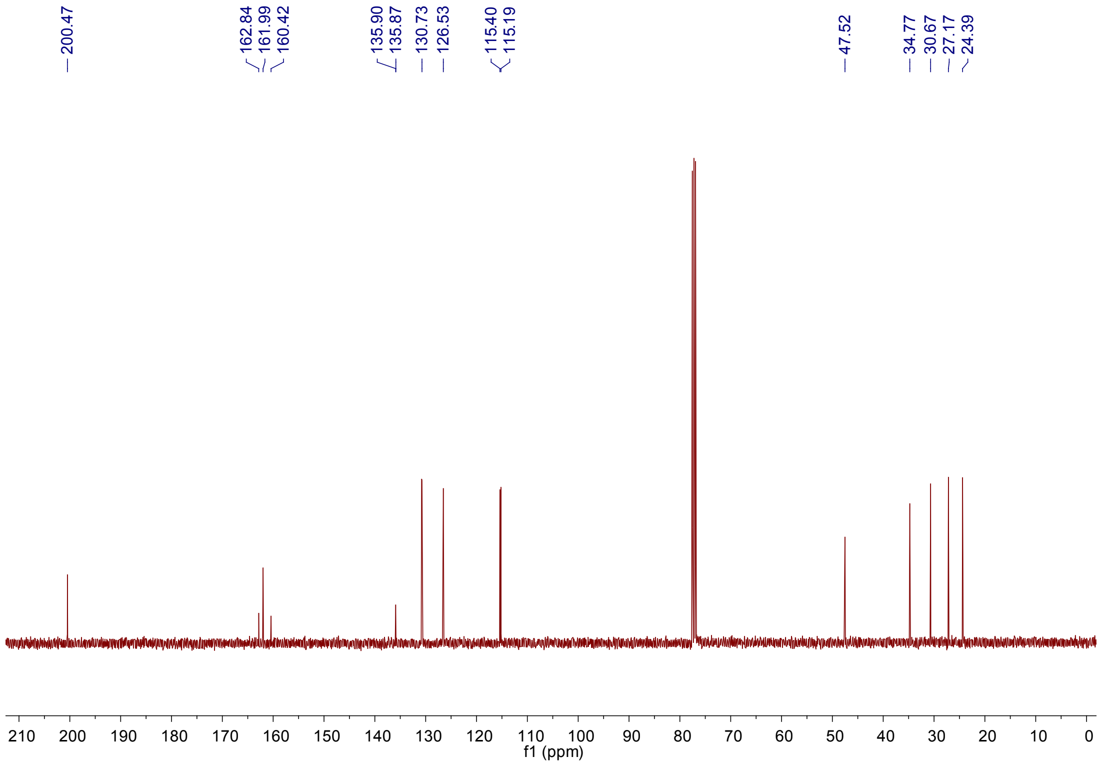

^{19}F NMR (377 MHz, $CDCl_3$) of compound **2b**

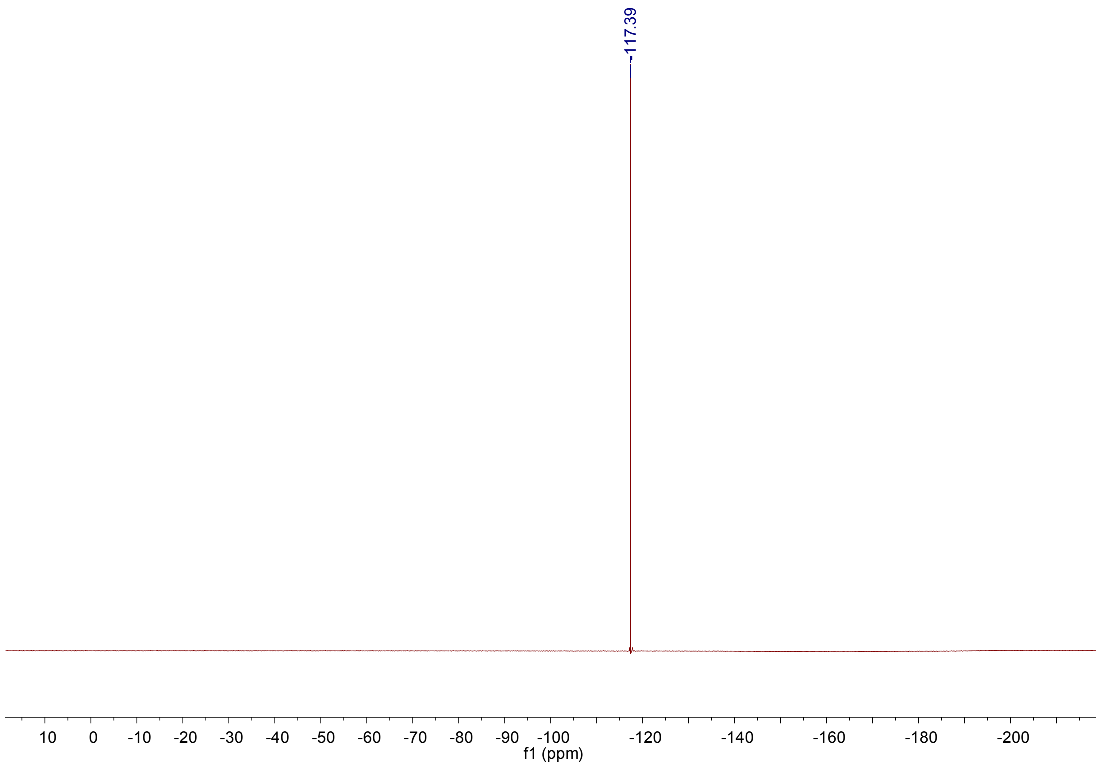

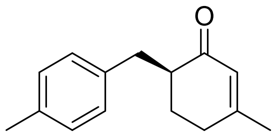

^{1}H NMR (400 MHz, $CDCl_3$) of compound **2c**

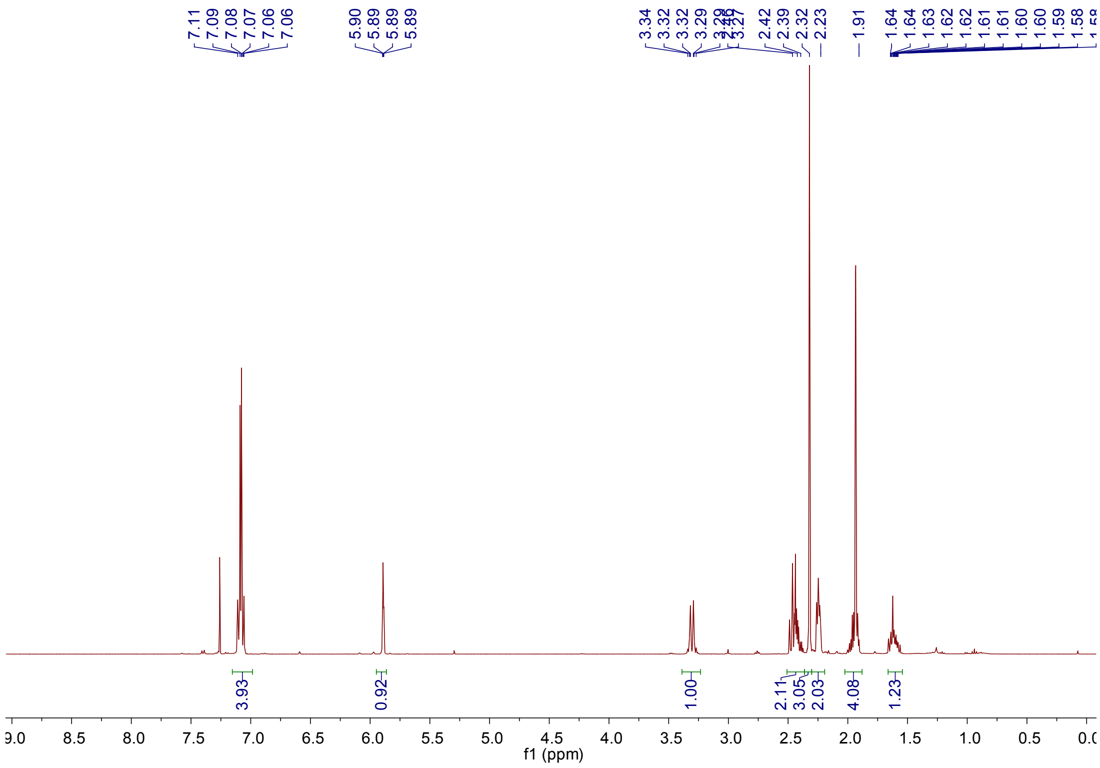

^{13}C NMR (100 MHz, $CDCl_3$) of compound **2c**

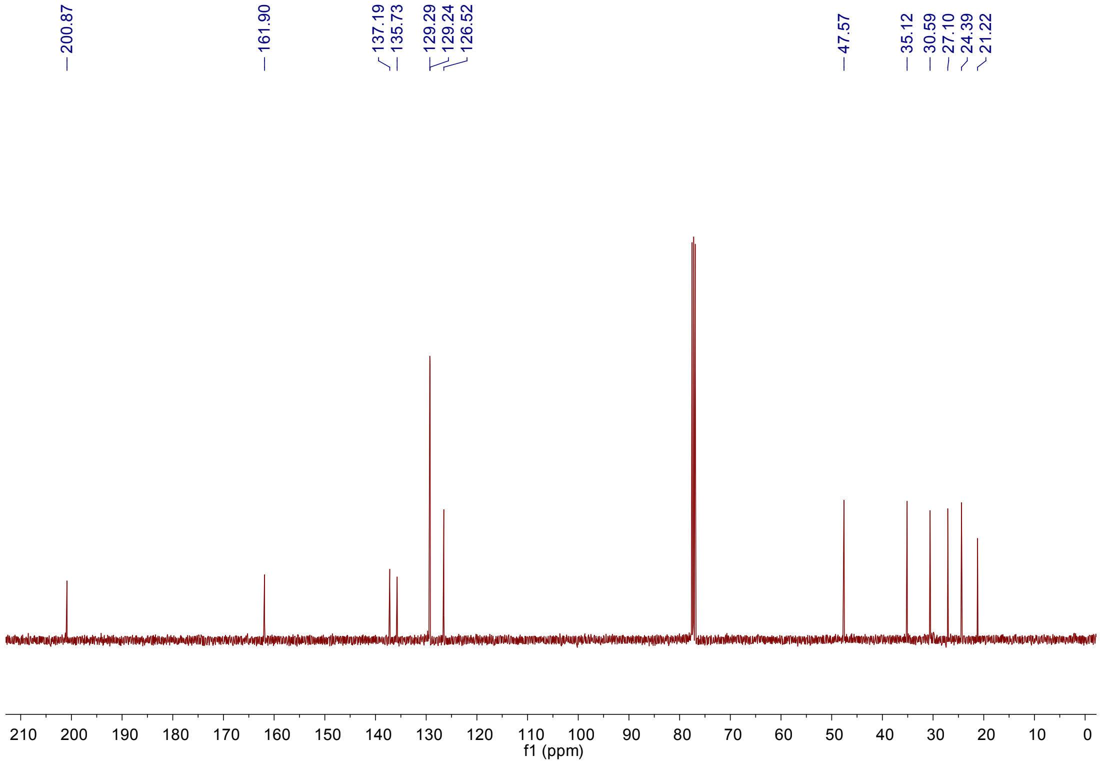

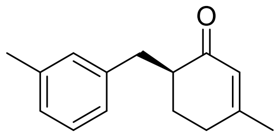

^{1}H NMR (400 MHz, $CDCl_3$) of compound **2d**

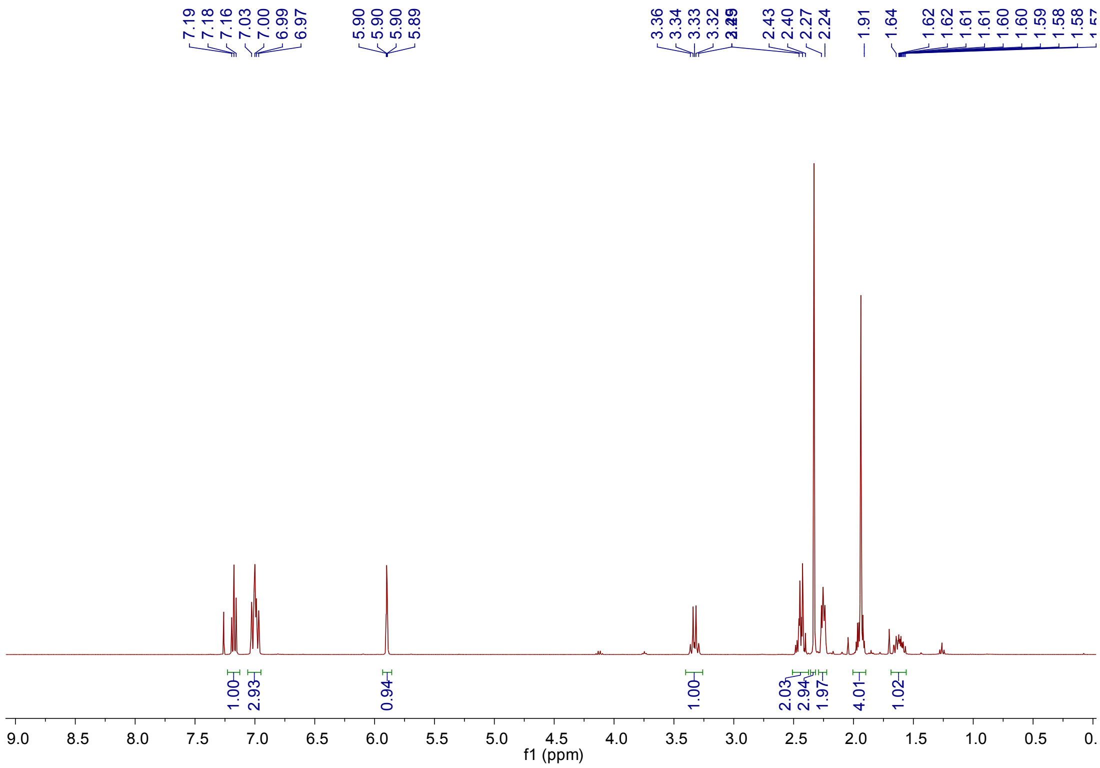

^{13}C NMR (100 MHz, $CDCl_3$) of compound **2d**

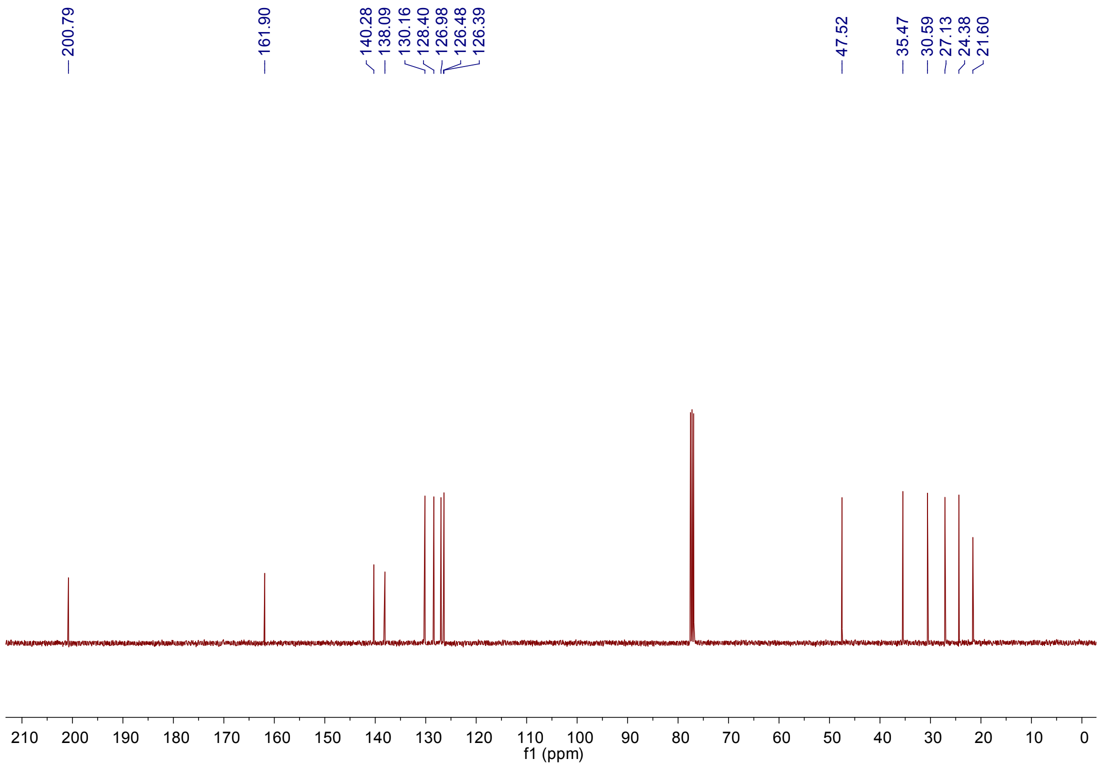

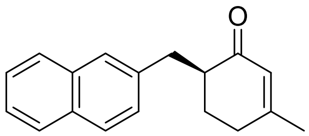

^{1}H NMR (400 MHz, $CDCl_3$) of compound **2e**

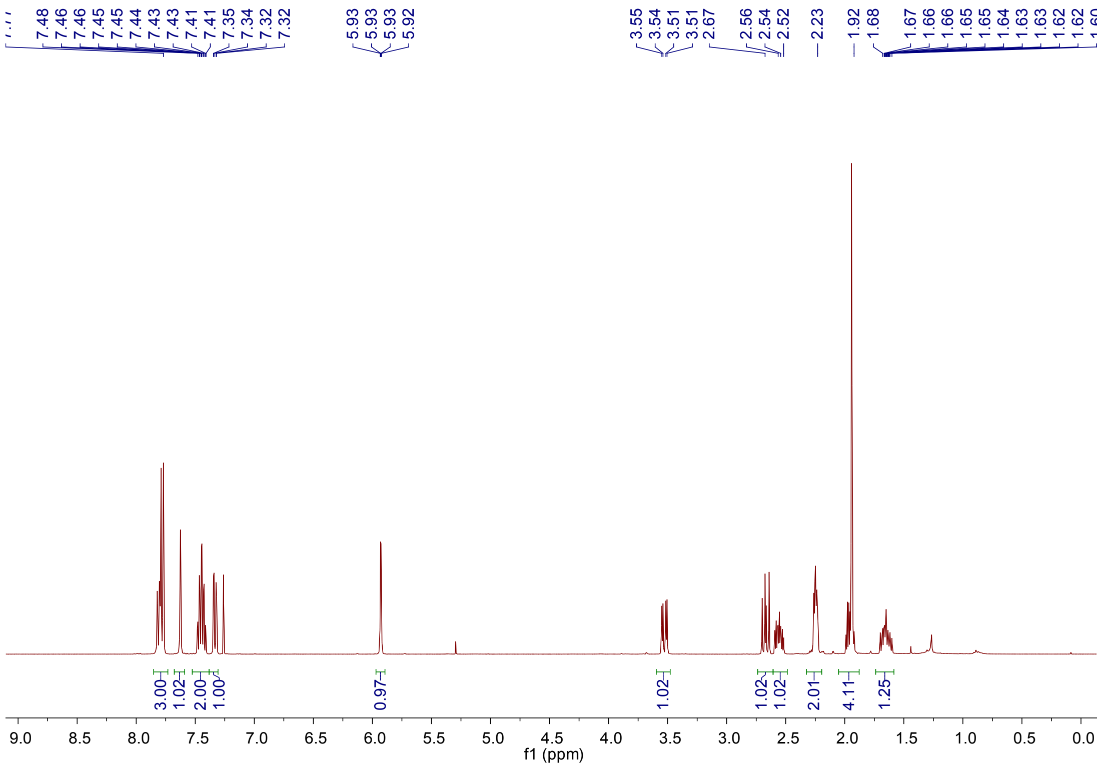

^{13}C NMR (100 MHz, $CDCl_3$) of compound **2e**

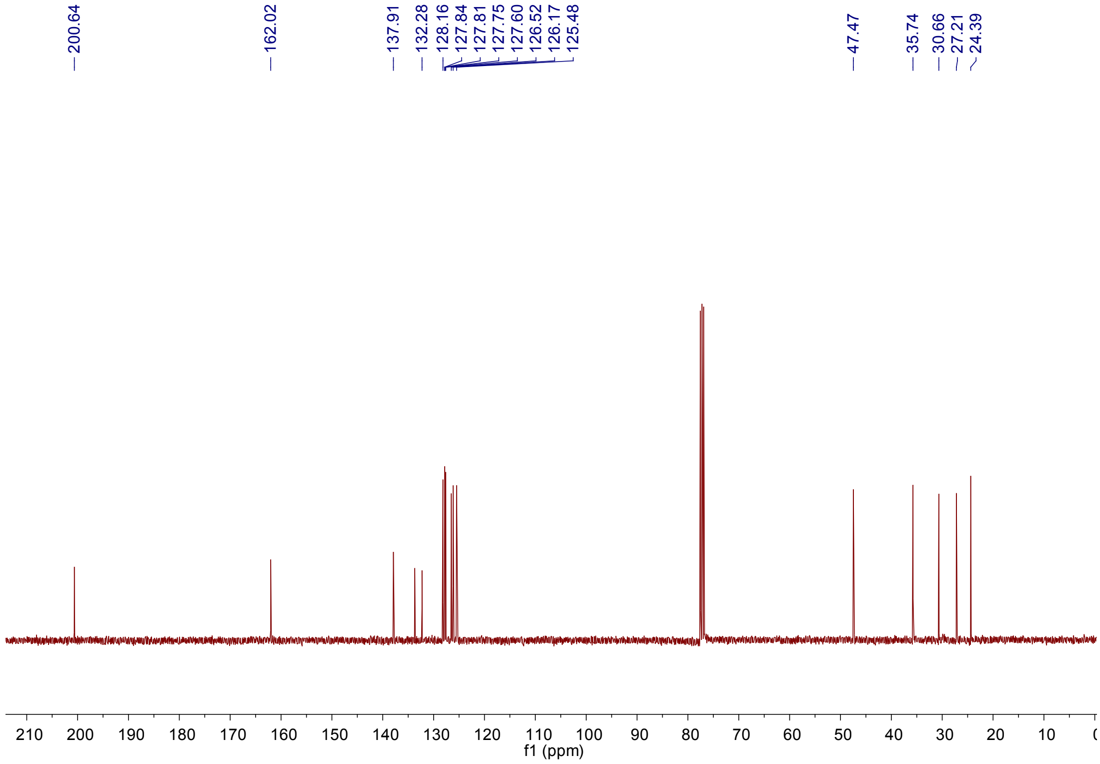

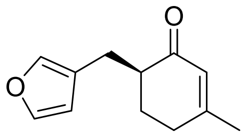

[1]**H NMR** (400 MHz, $CDCl_3$) of compound **2f**

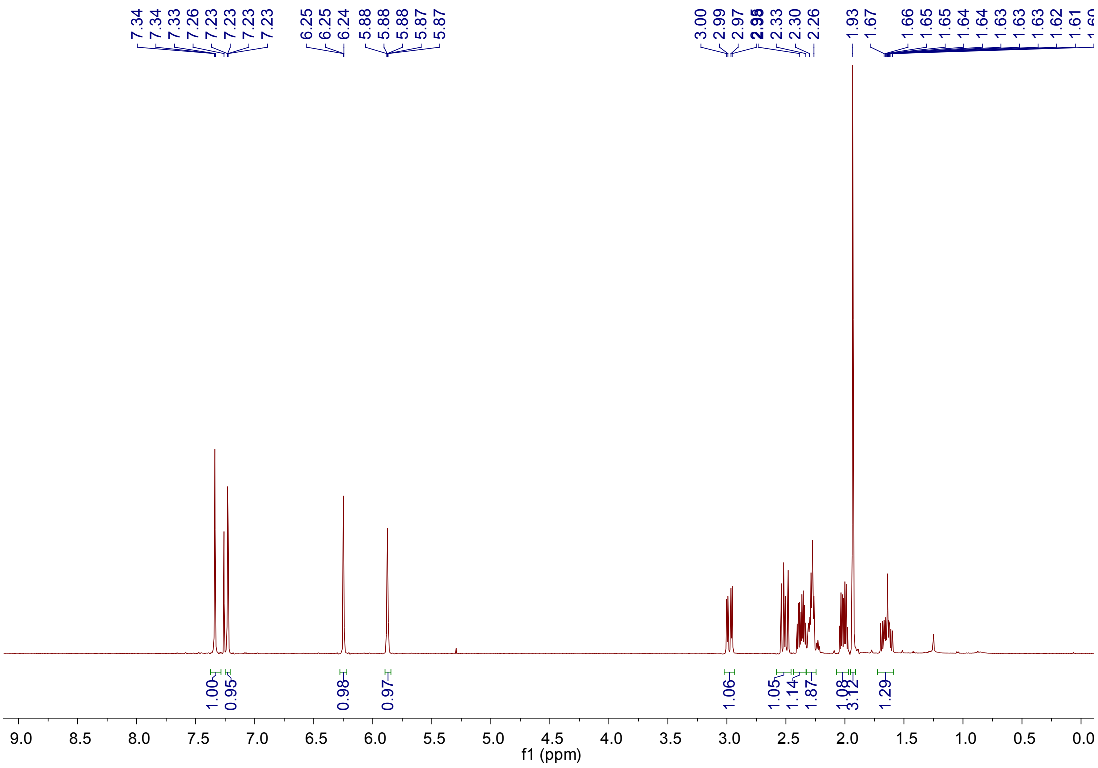

[13]**C NMR** (100 MHz, $CDCl_3$) of compound **2f**

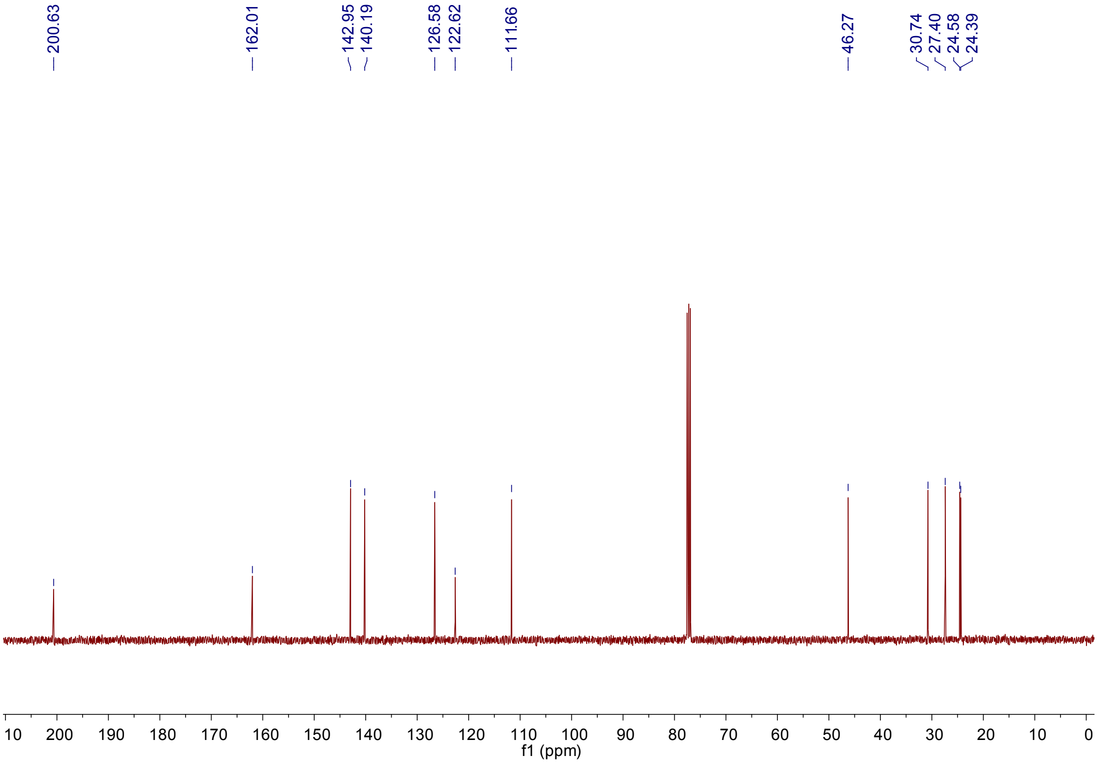

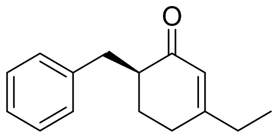

¹H NMR (400 MHz, $CDCl_3$) of compound **2g**

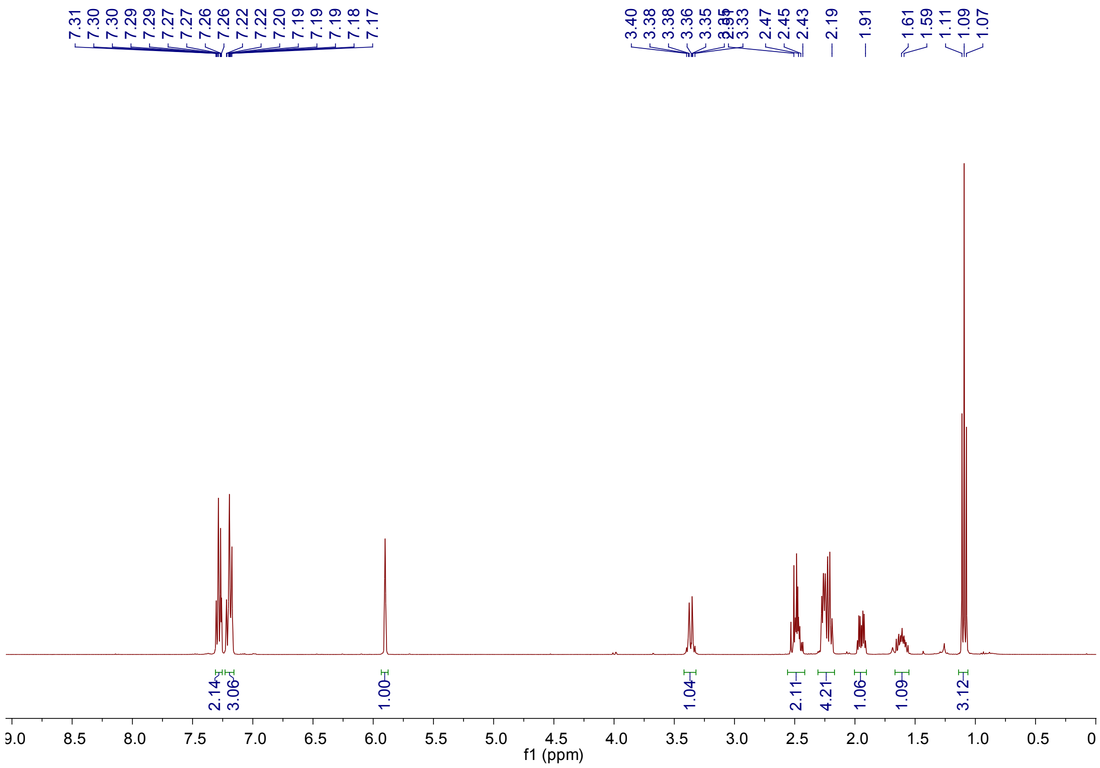

¹³C NMR (100 MHz, $CDCl_3$) of compound **2g**

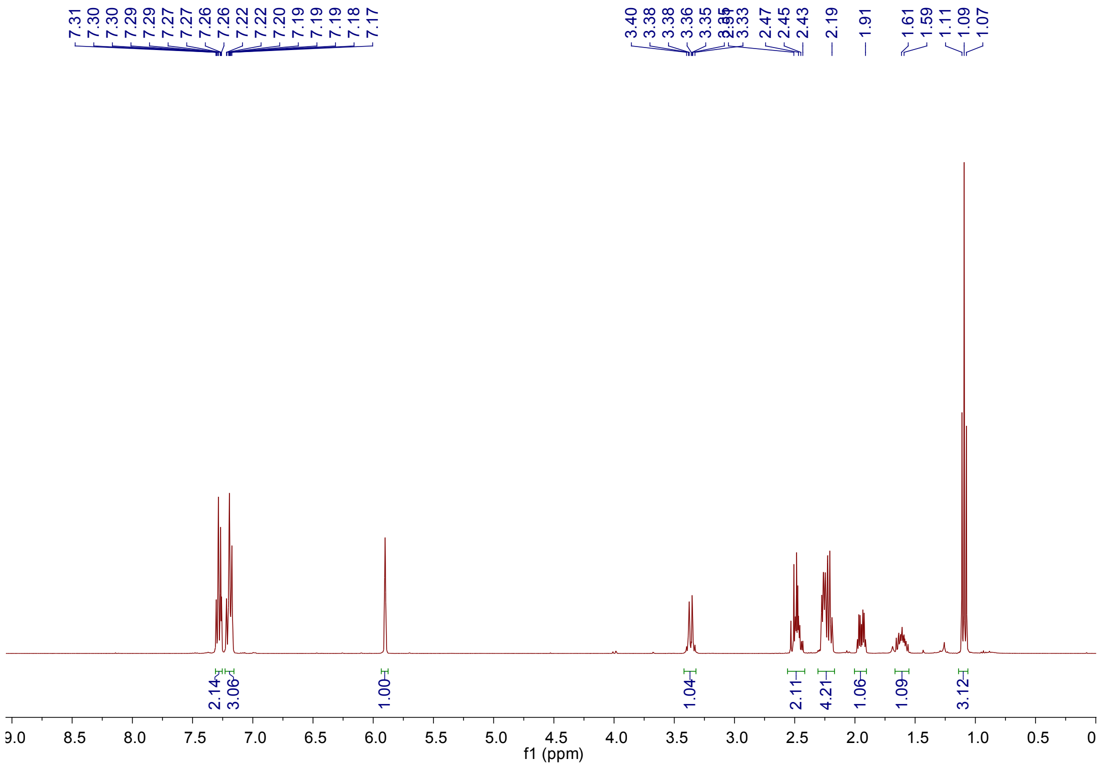

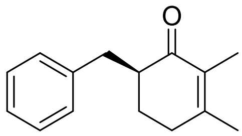

^{1}H NMR (400 MHz, $CDCl_3$) of compound **2h**

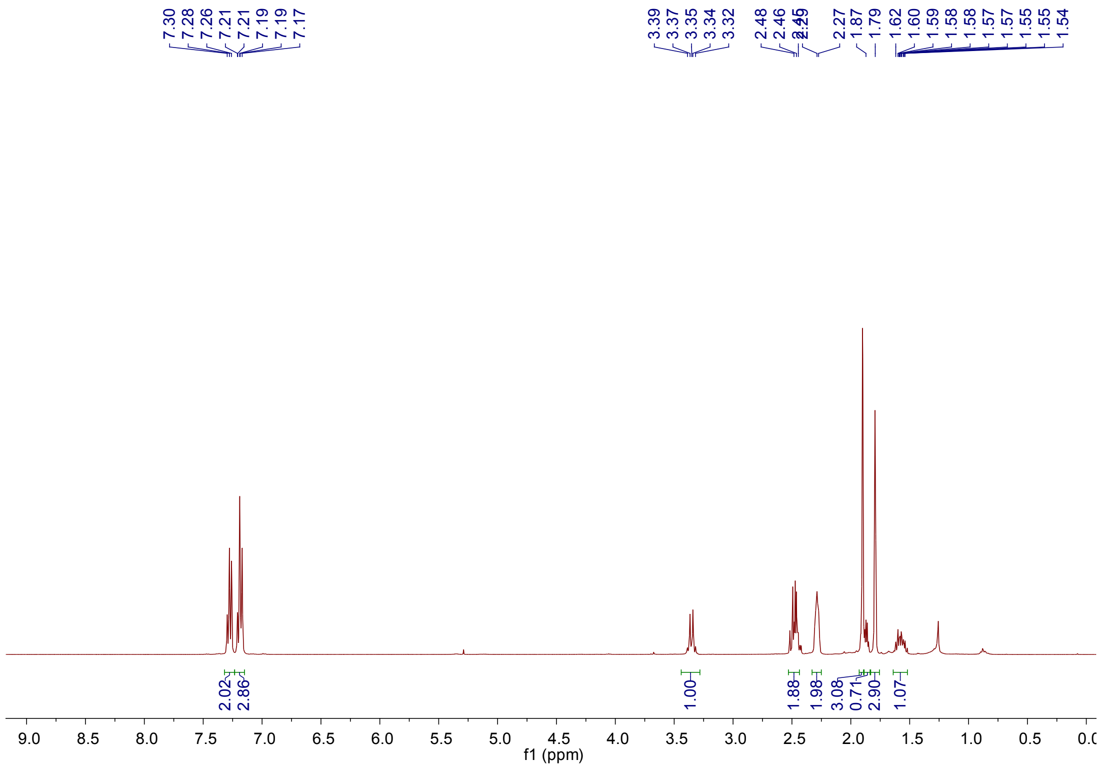

^{13}C NMR (100 MHz, $CDCl_3$) of compound **2h**

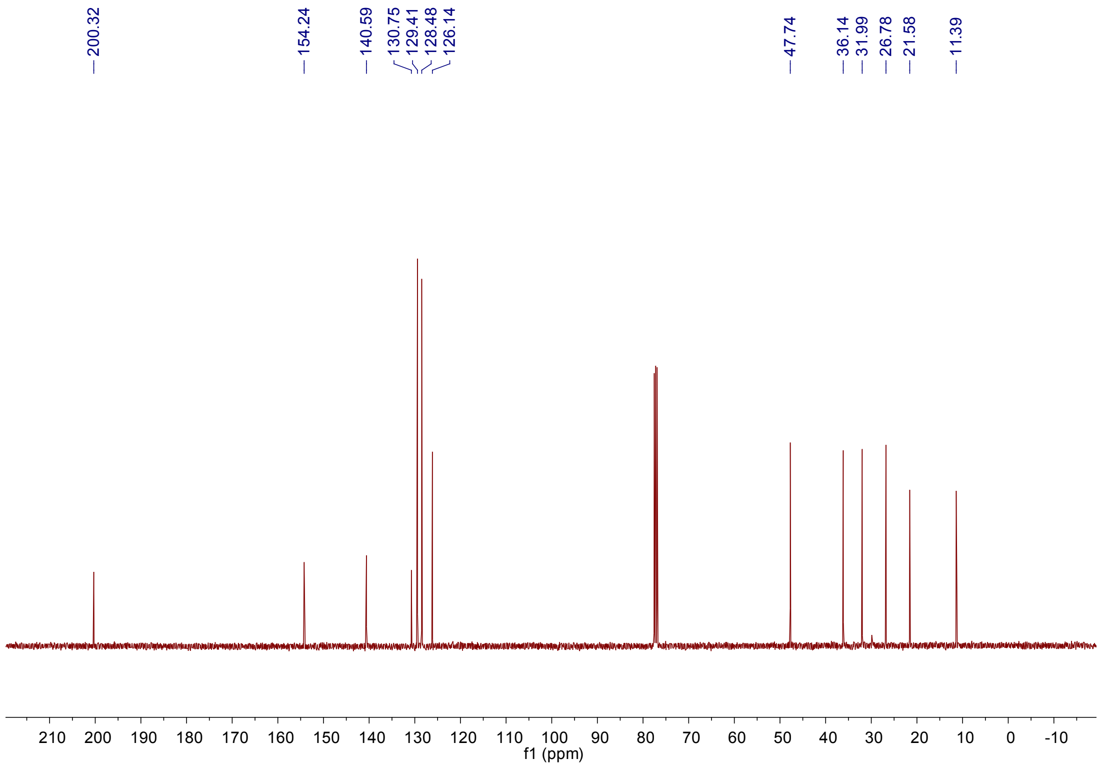

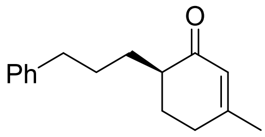

^{1}H NMR (400 MHz, $CDCl_3$) of compound **2i**

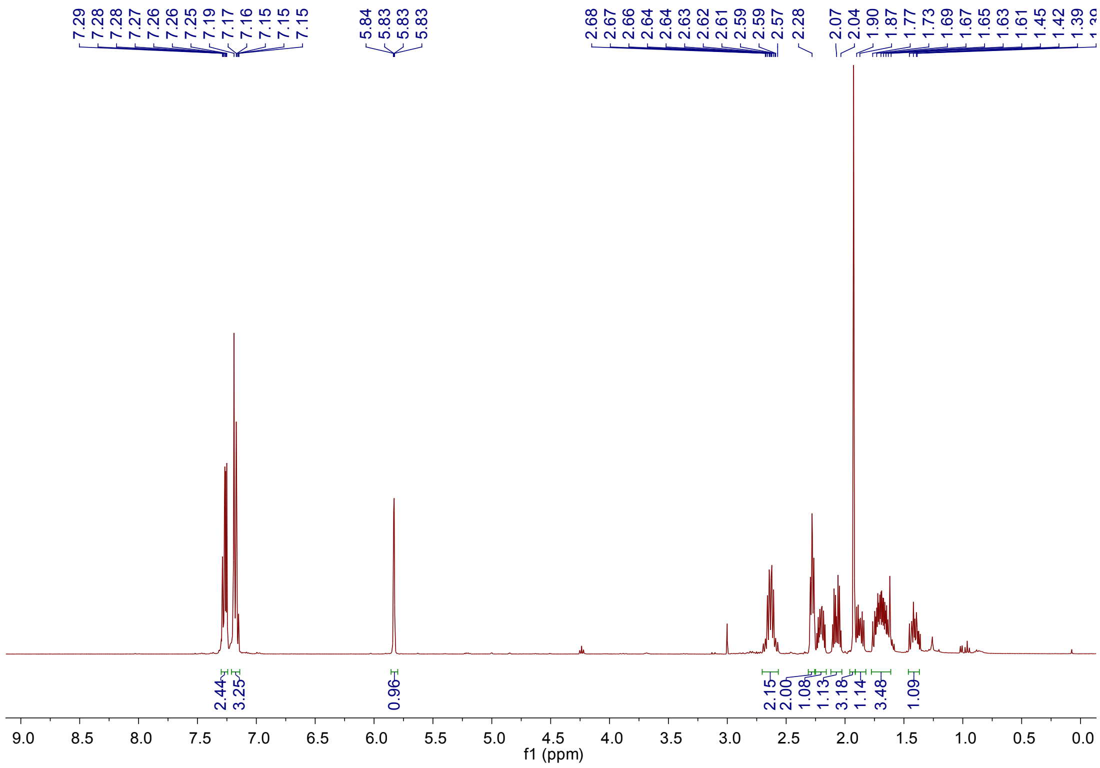

^{13}C NMR (100 MHz, $CDCl_3$) of compound **2i**

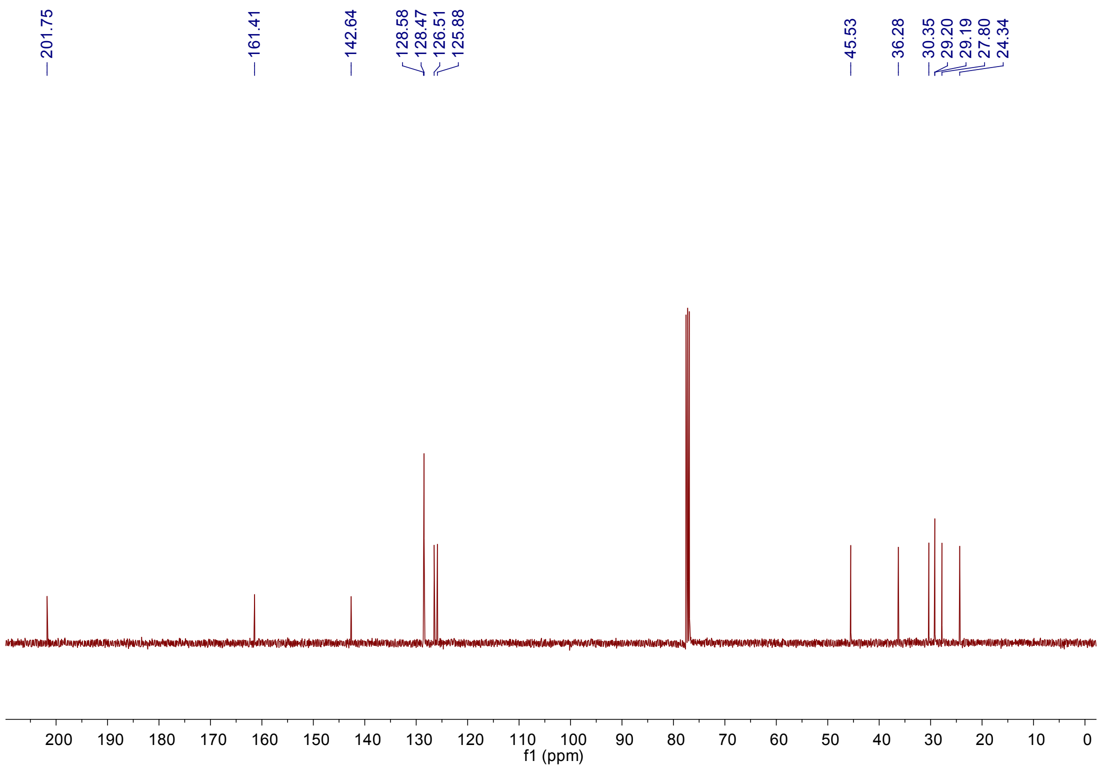

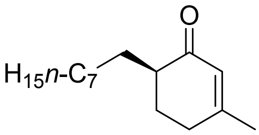

^{1}H NMR (400 MHz, $CDCl_3$) of compound **2j**

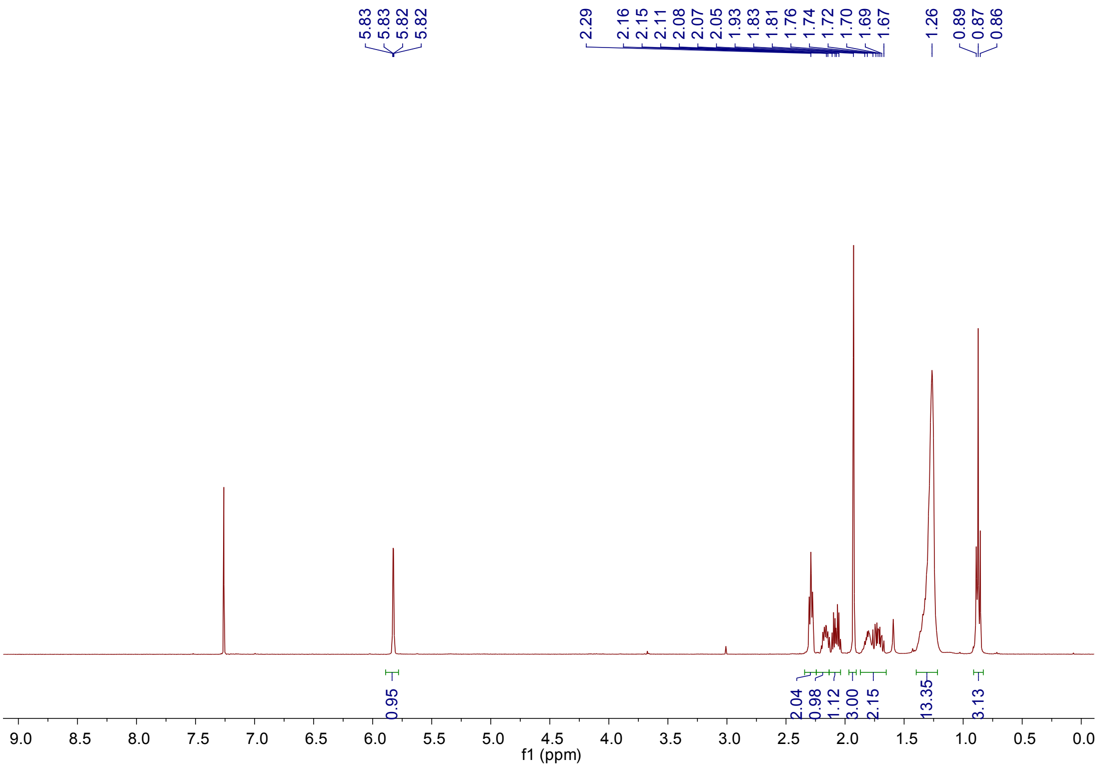

^{13}C NMR (100 MHz, $CDCl_3$) of compound **2j**

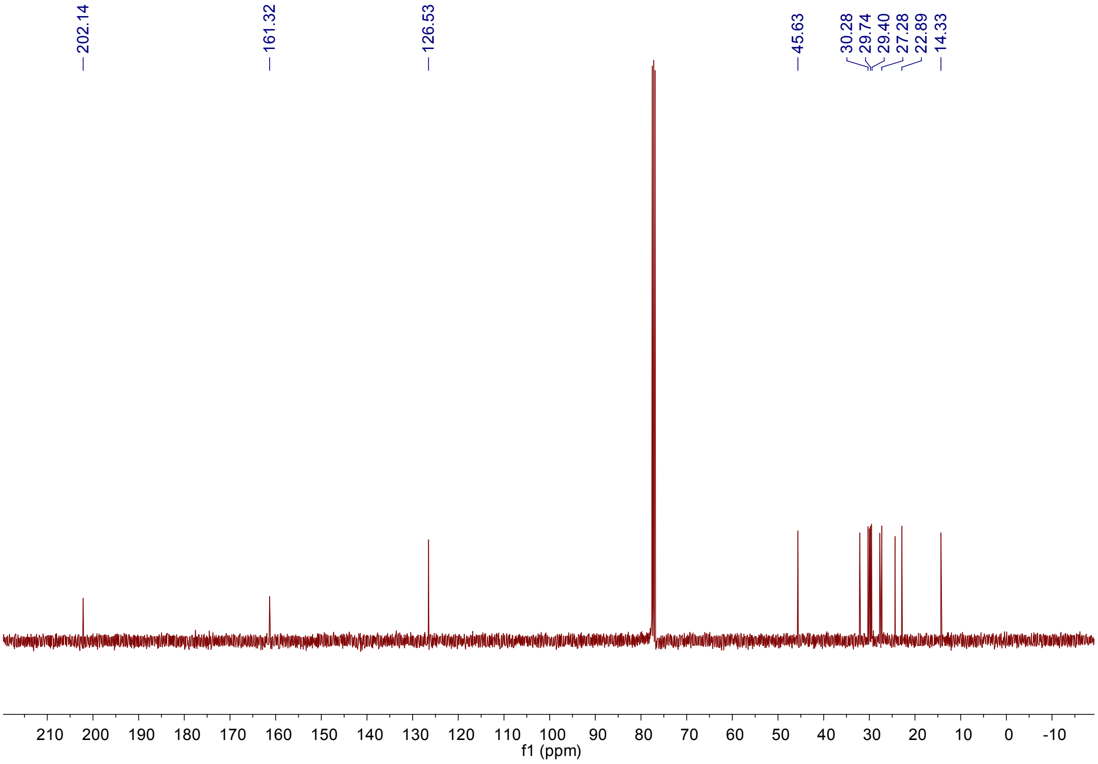

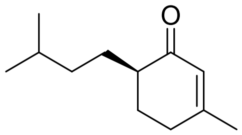

^{1}H NMR (400 MHz, $CDCl_3$) of compound **2k**

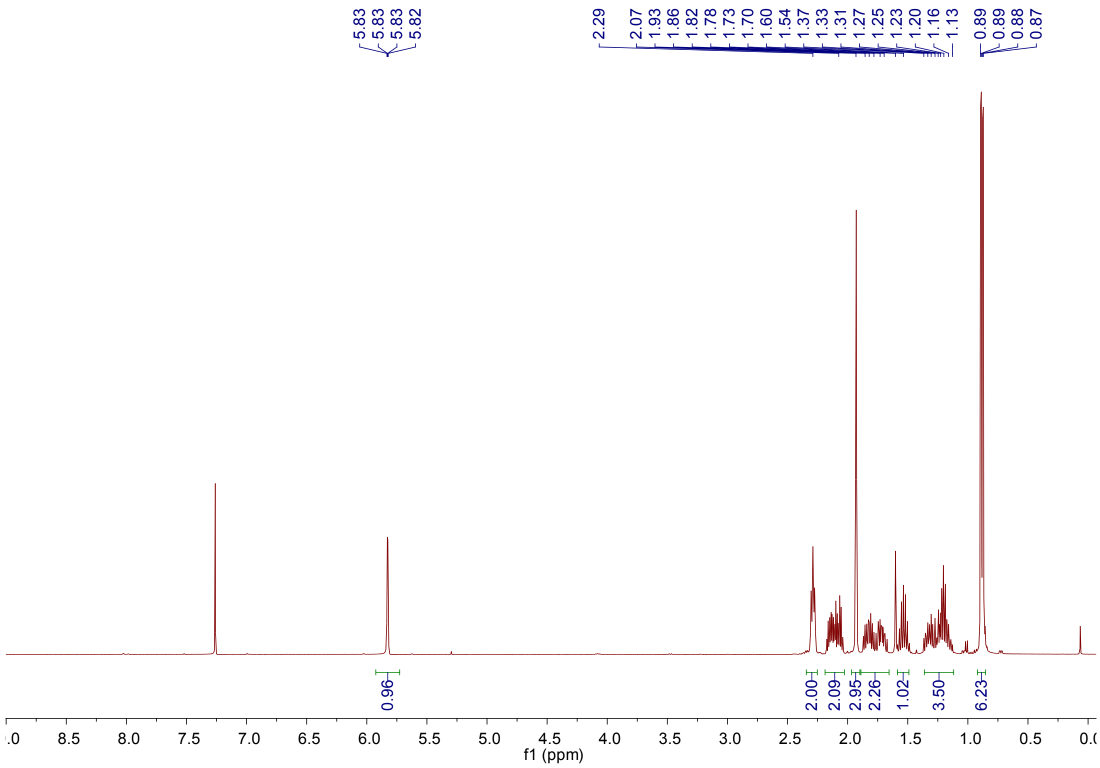

^{13}C NMR (100 MHz, $CDCl_3$) of compound **2k**

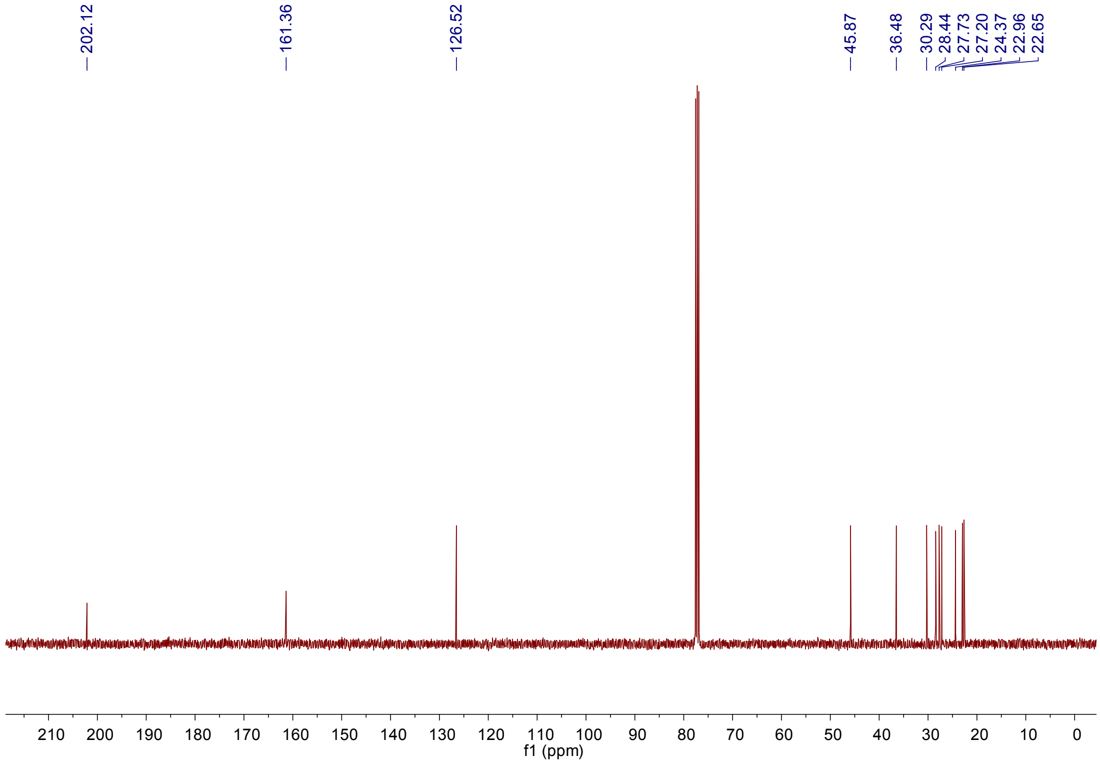

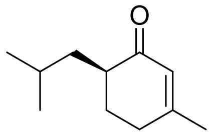

¹H NMR (400 MHz, $CDCl_3$) of compound **2l**

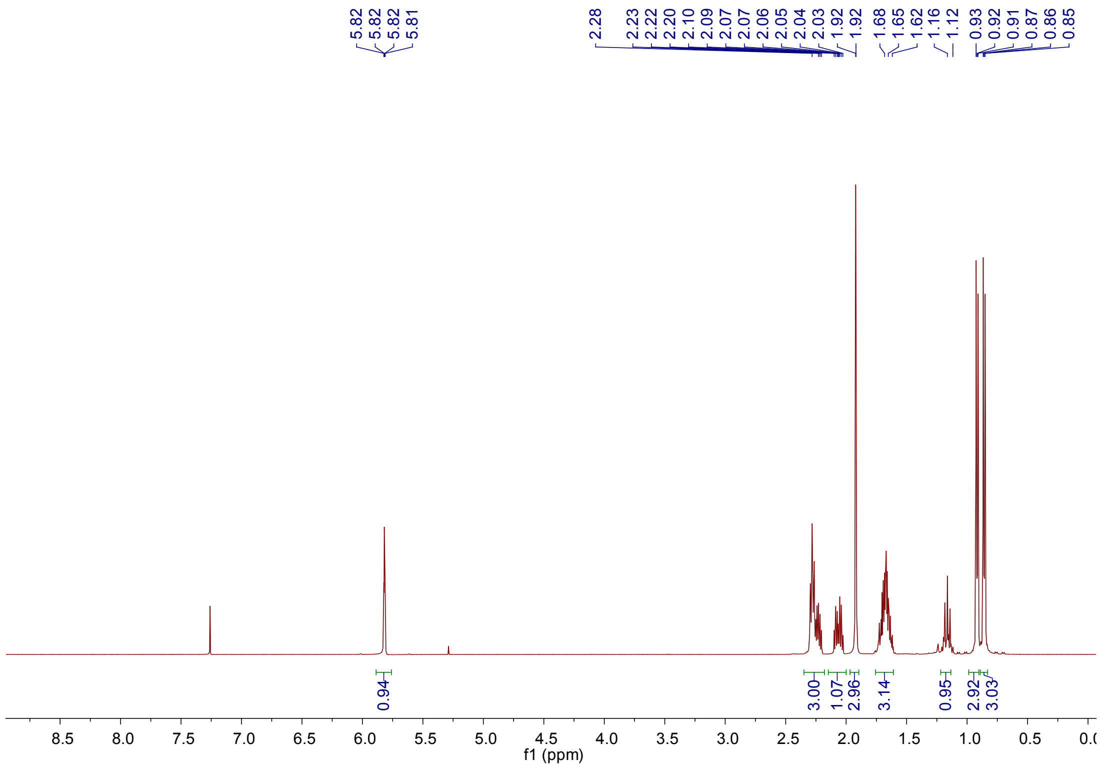

¹³C NMR (100 MHz, $CDCl_3$) of compound **2l**

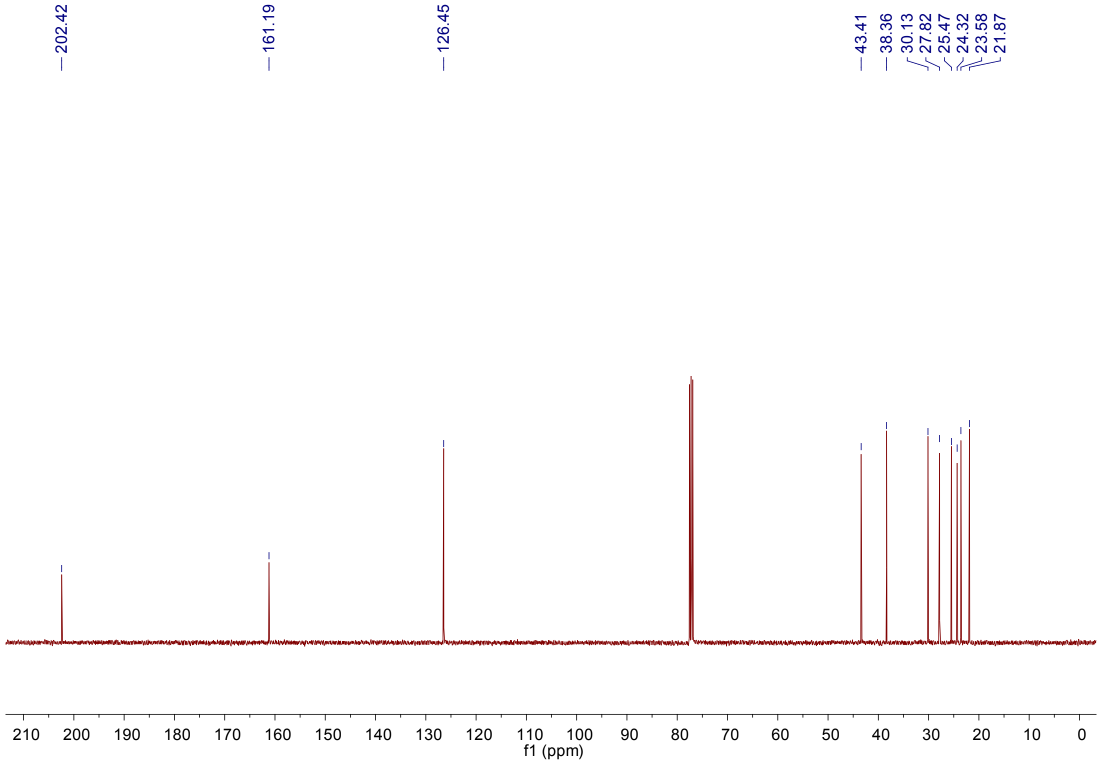

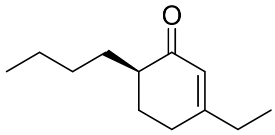

¹H NMR (400 MHz, $CDCl_3$) of compound **2m**

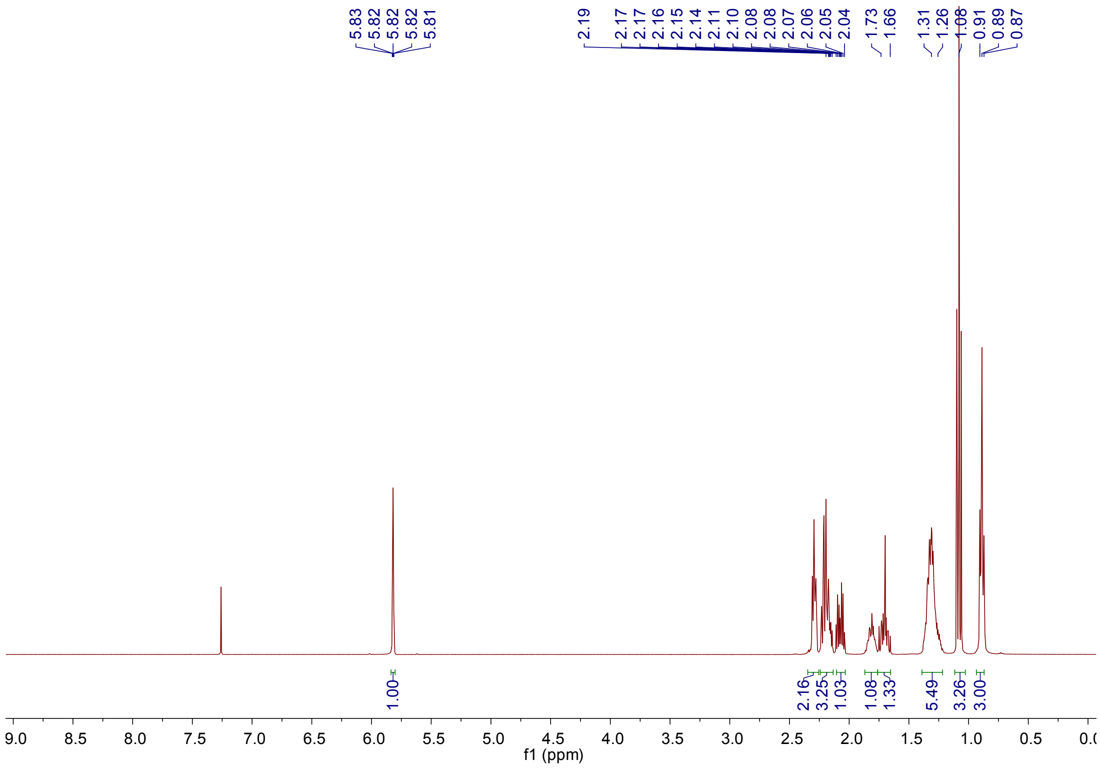

¹³C NMR (100 MHz, $CDCl_3$) of compound **2m**

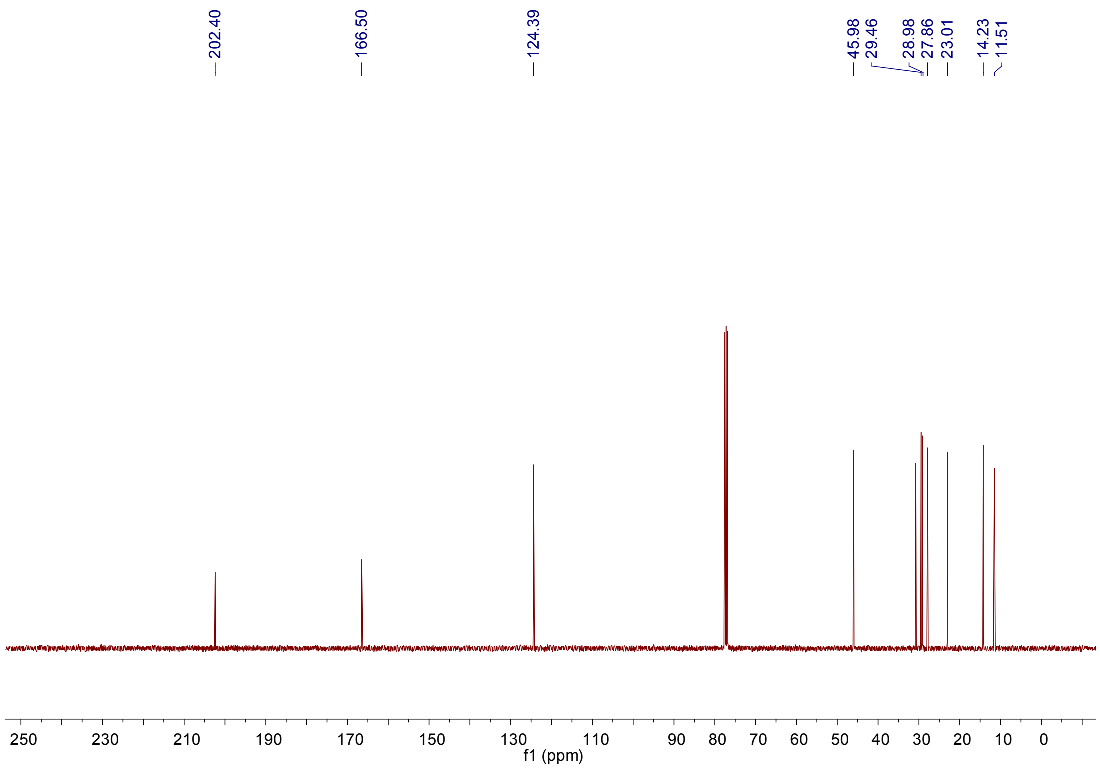

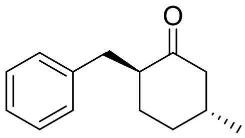

¹H NMR (400 MHz, $CDCl_3$) of compound **3a**

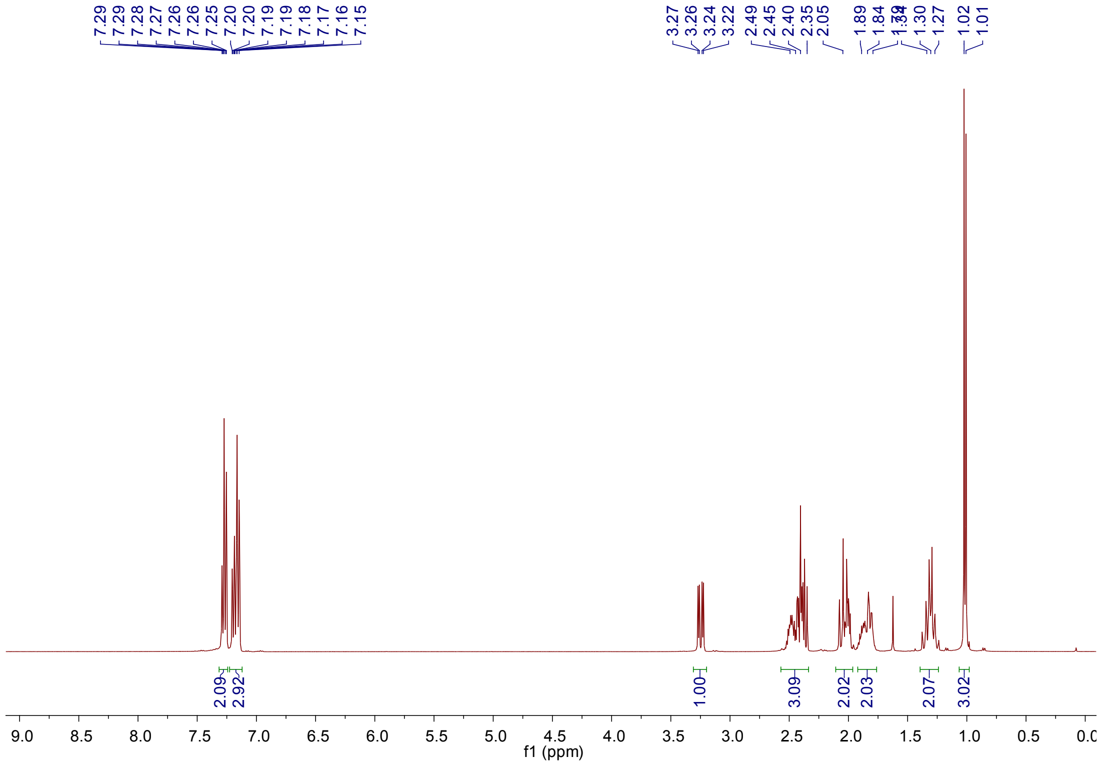

¹³C NMR (100 MHz, $CDCl_3$) of compound **3a**

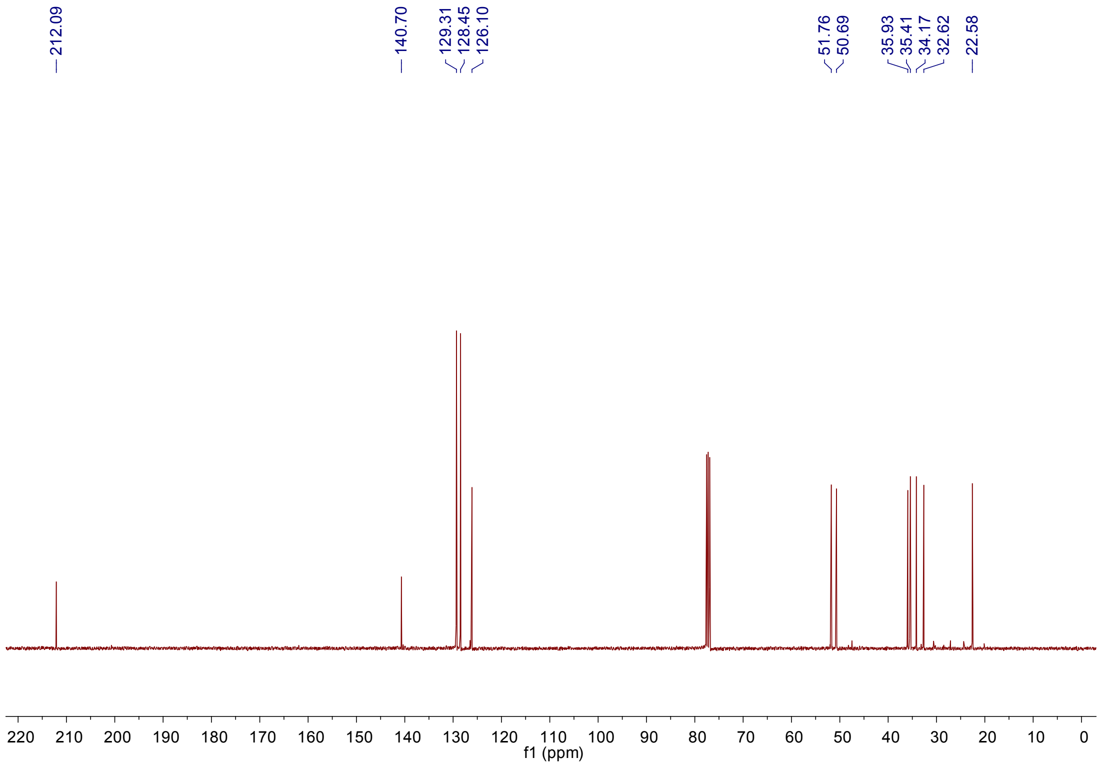

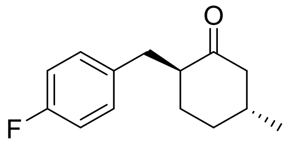

^{1}H NMR (400 MHz, $CDCl_3$) of compound **3b**

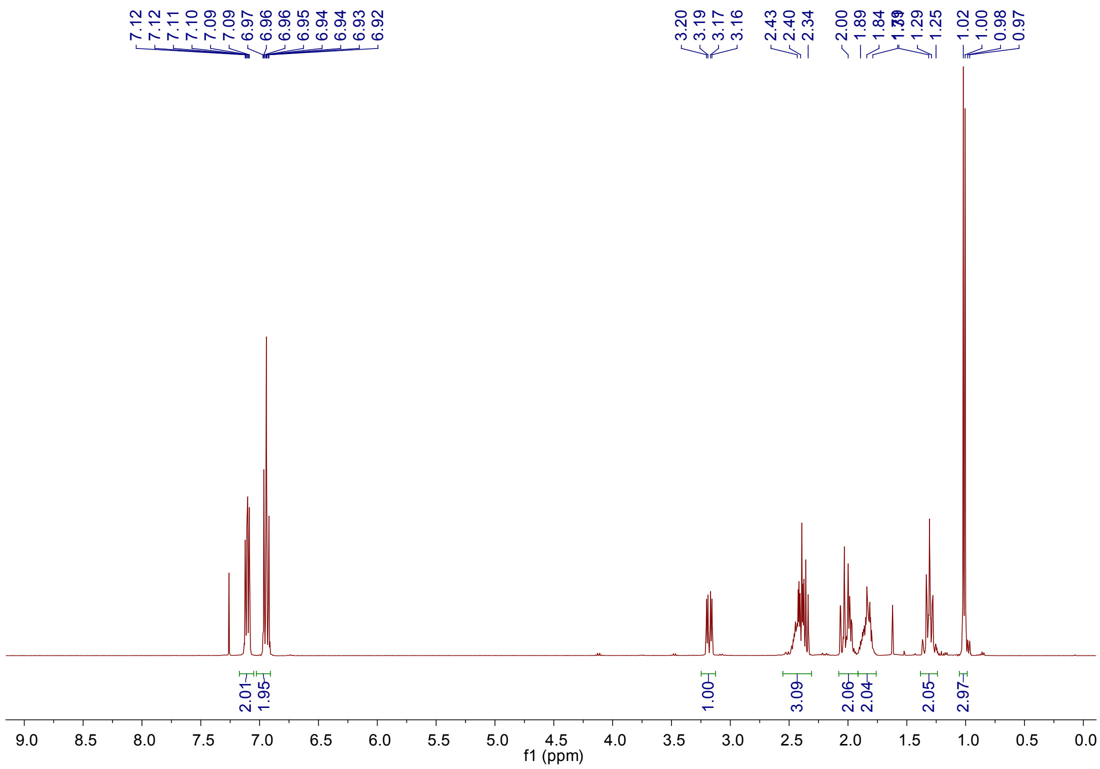

^{13}C NMR (100 MHz, $CDCl_3$) of compound **3b**

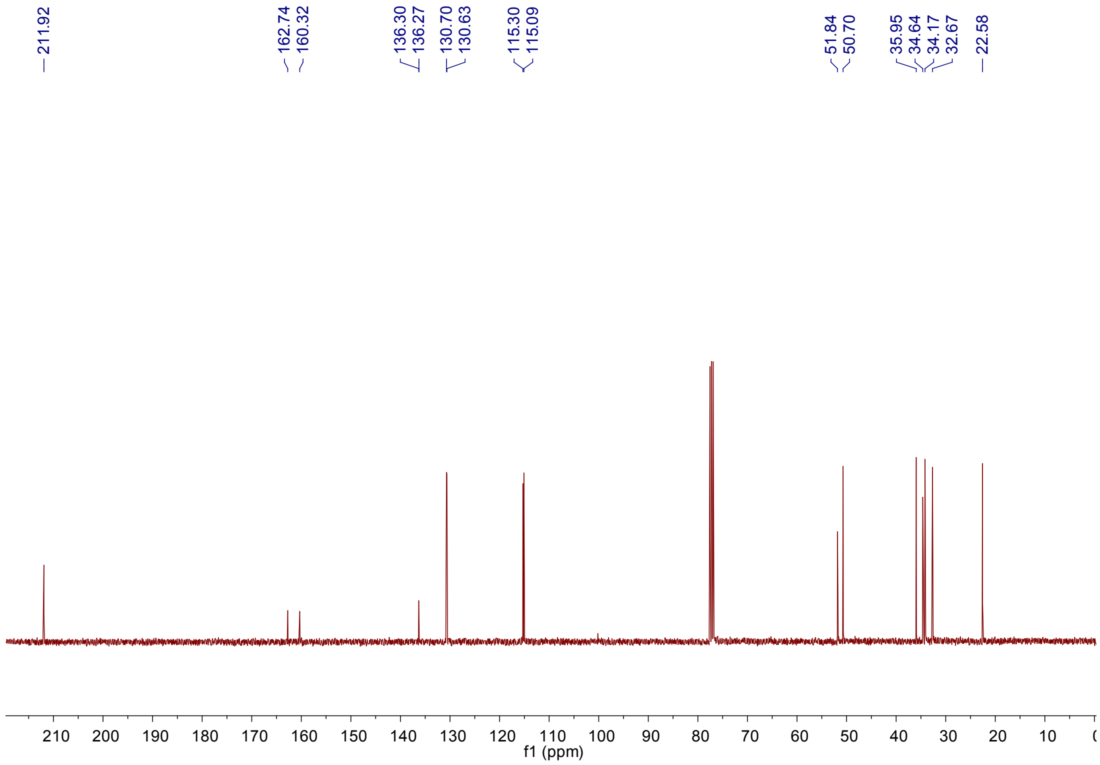

^{19}F NMR (377 MHz, $CDCl_3$) of compound **3b**

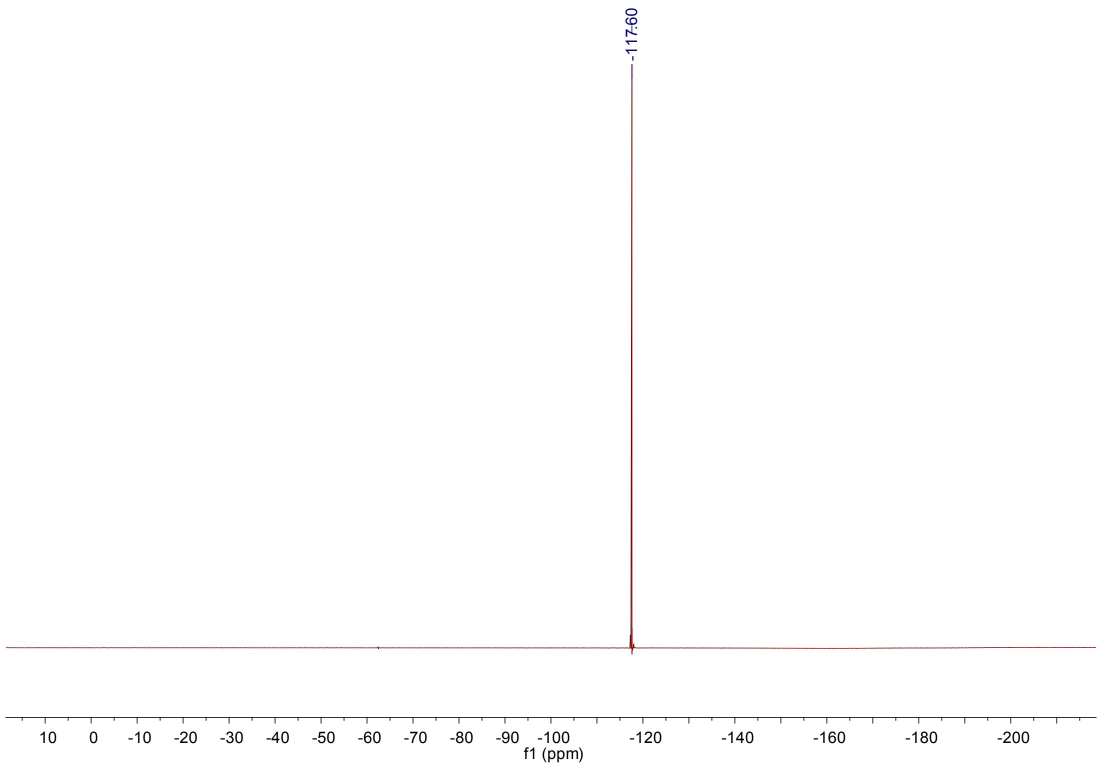

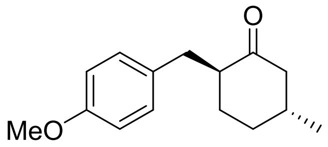

^{1}H NMR (400 MHz, $CDCl_3$) of compound **3n**

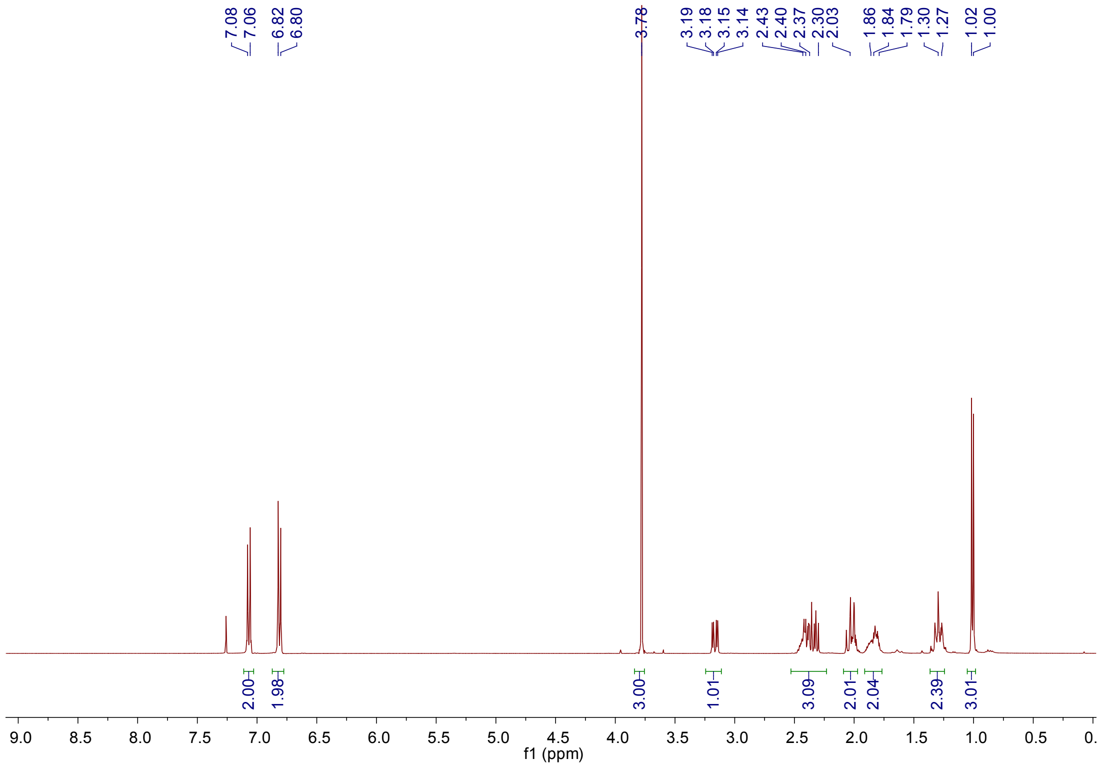

^{13}C NMR (100 MHz, $CDCl_3$) of compound **3n**

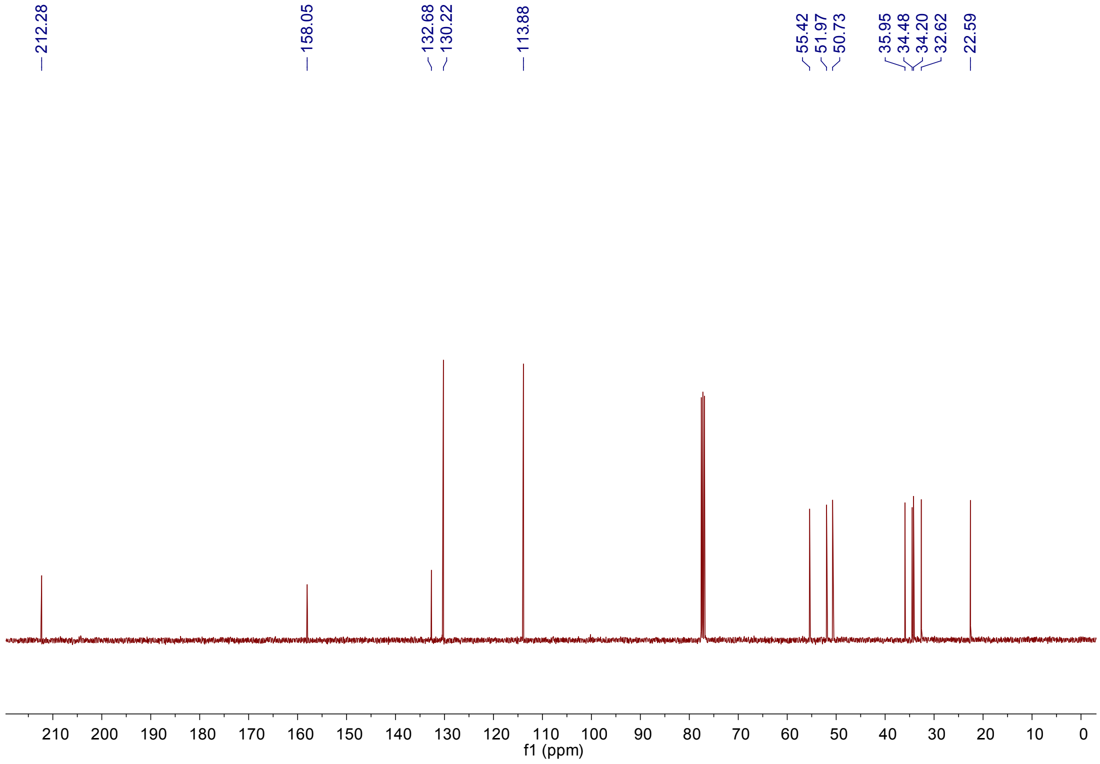

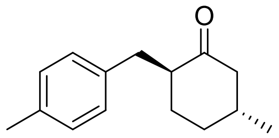

¹H NMR (400 MHz, $CDCl_3$) of compound **3c**

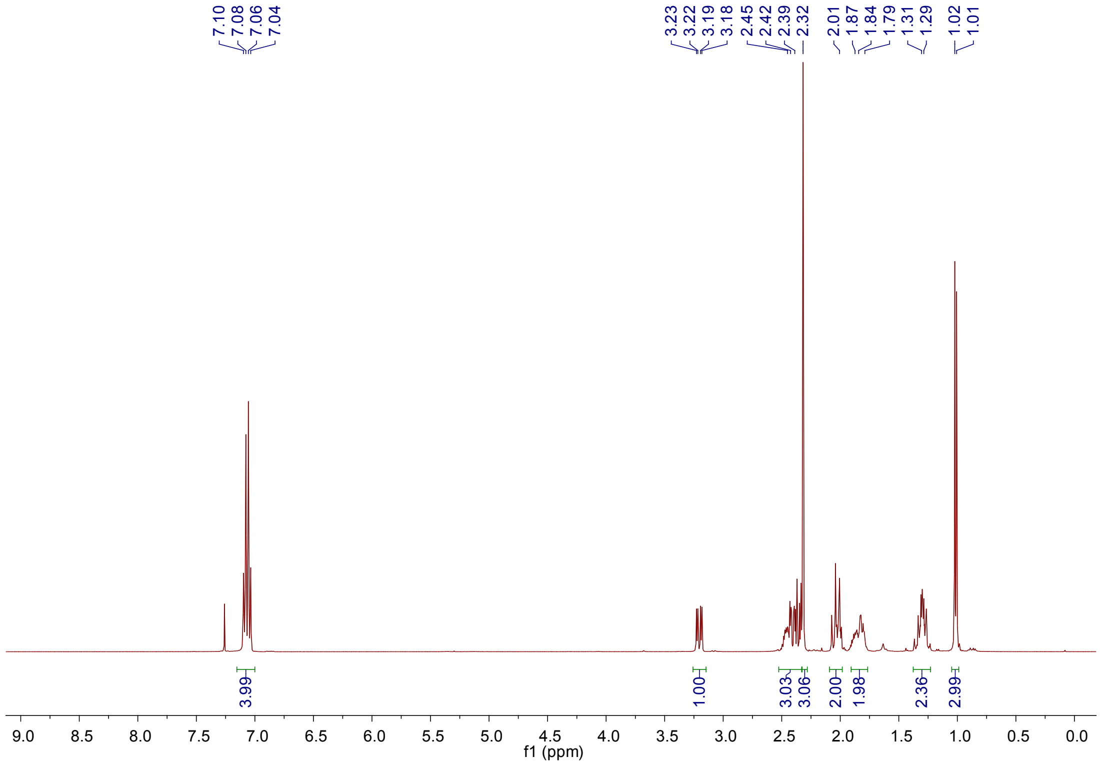

¹³C NMR (100 MHz, $CDCl_3$) of compound **3c**

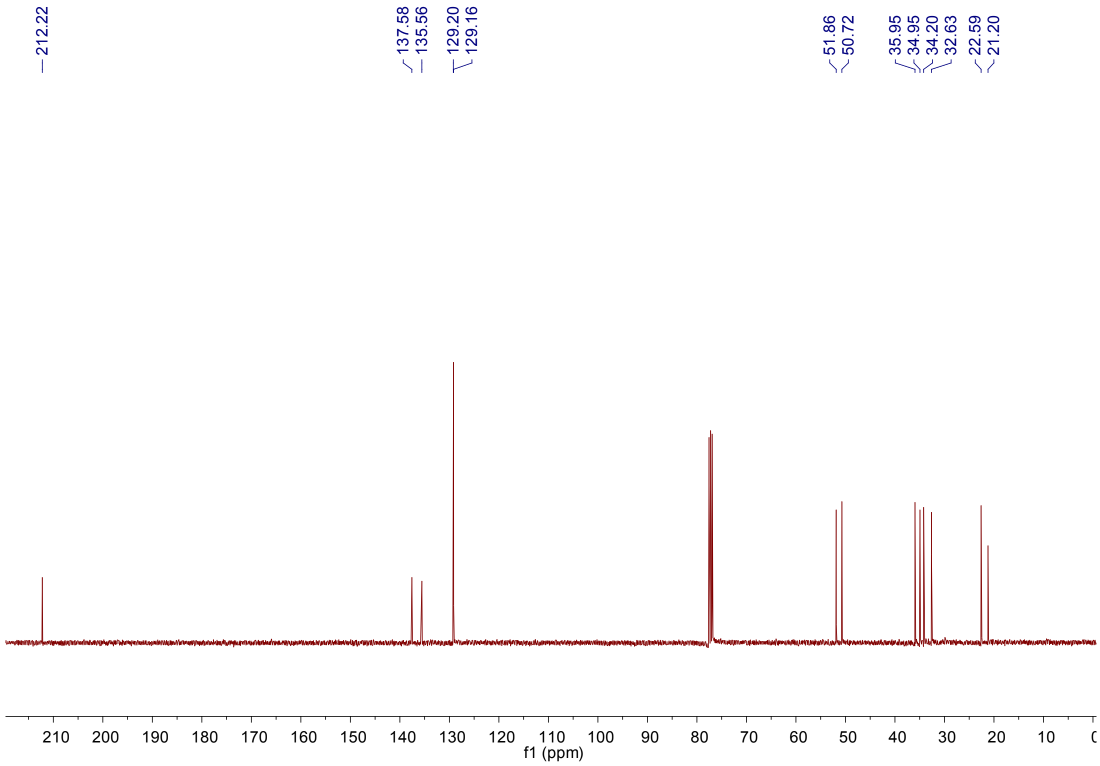

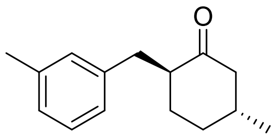

^{1}H NMR (400 MHz, $CDCl_3$) of compound **3d**

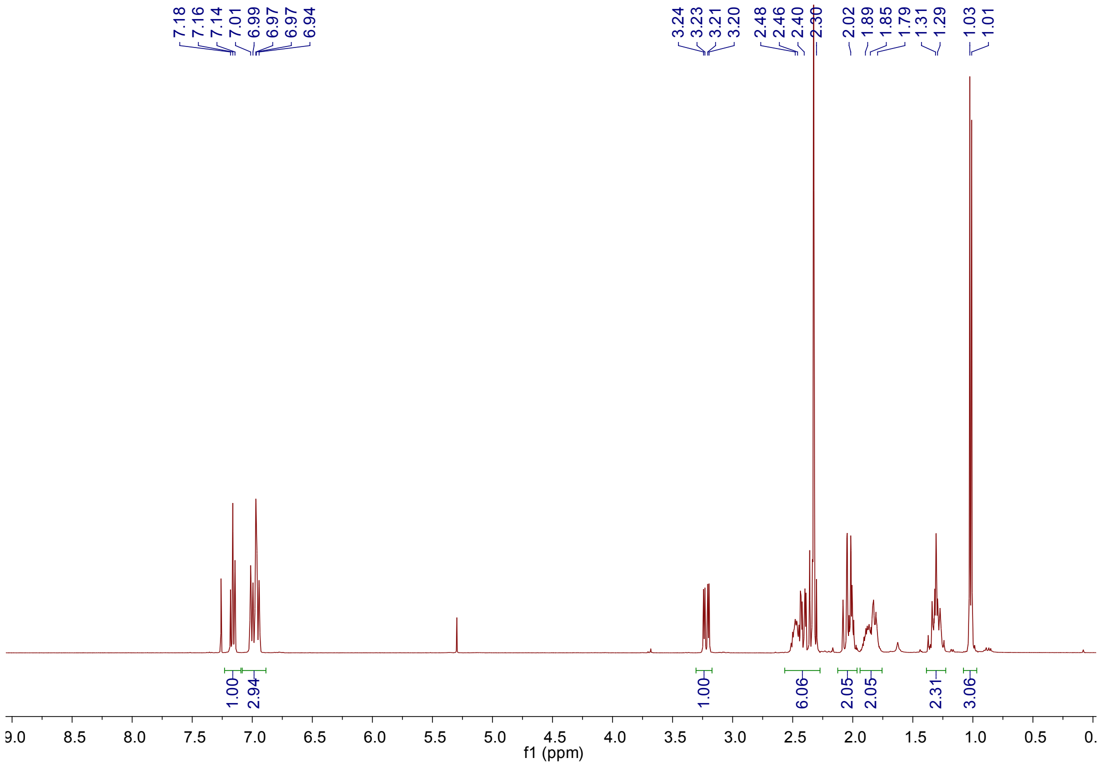

^{13}C NMR (100 MHz, $CDCl_3$) of compound **3d**

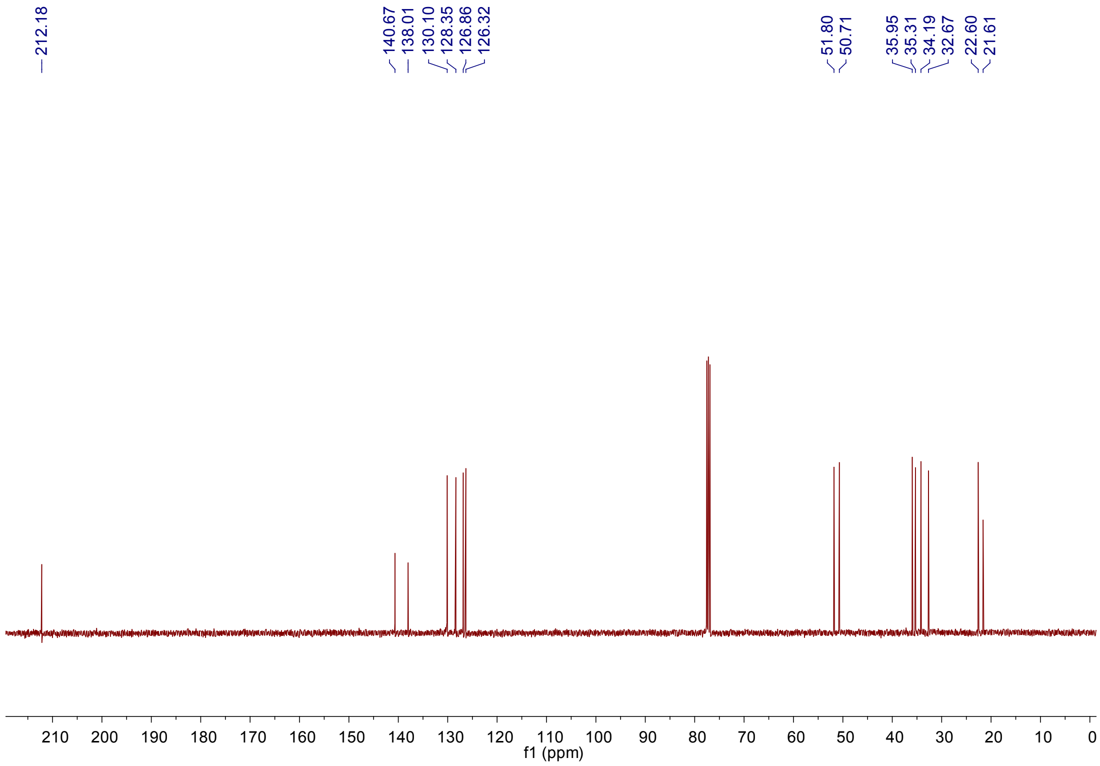

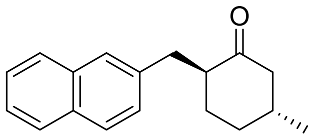

^{1}H NMR (400 MHz, $CDCl_3$) of compound **3e**

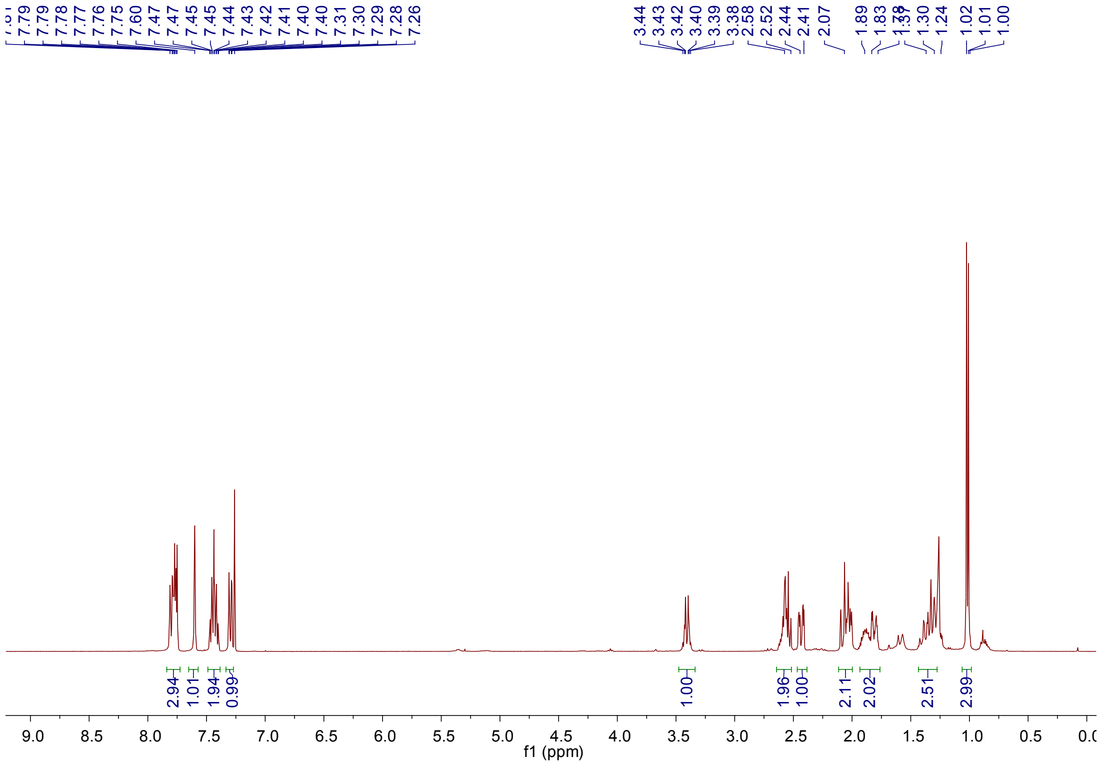

^{13}C NMR (100 MHz, $CDCl_3$) of compound **3e**

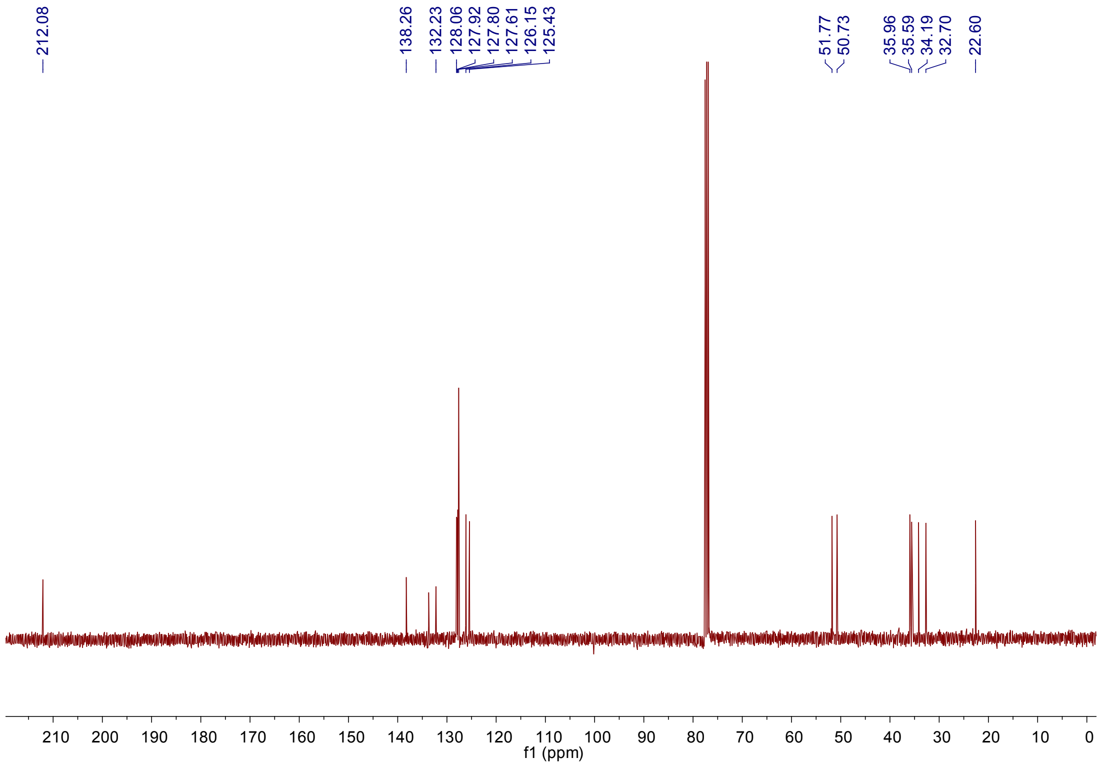

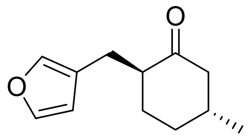

^{1}H NMR (400 MHz, $CDCl_3$) of compound **3f**

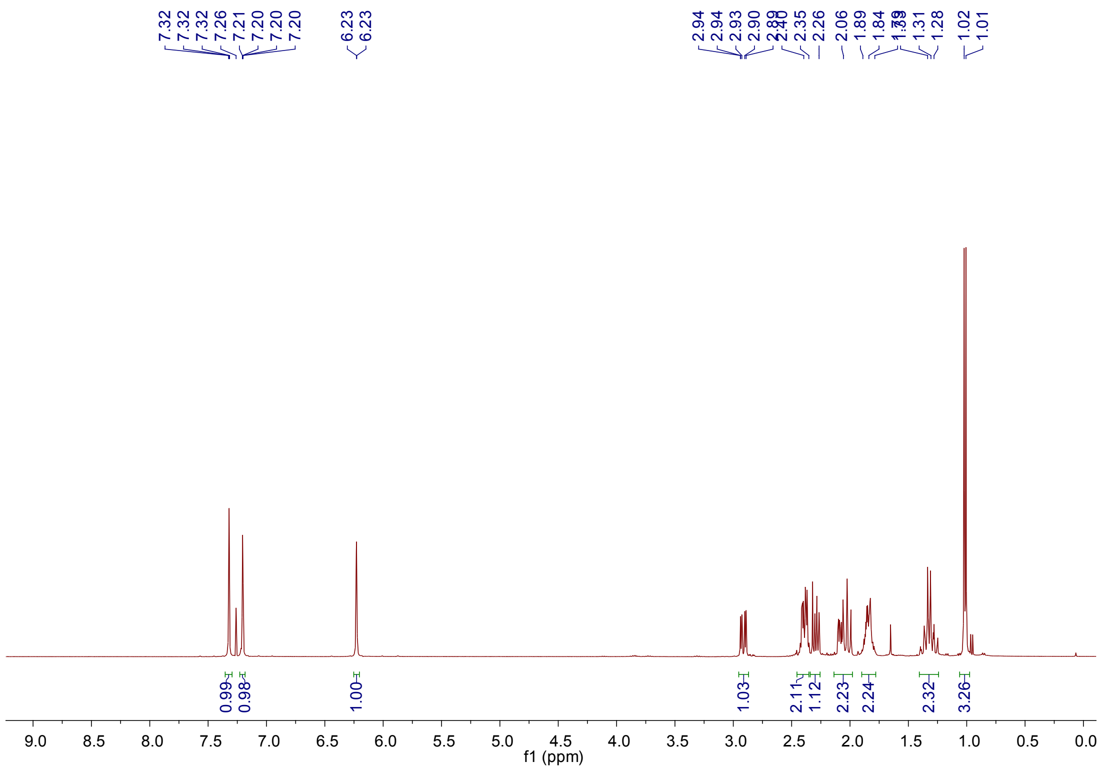

^{13}C NMR (100 MHz, $CDCl_3$) of compound **3f**

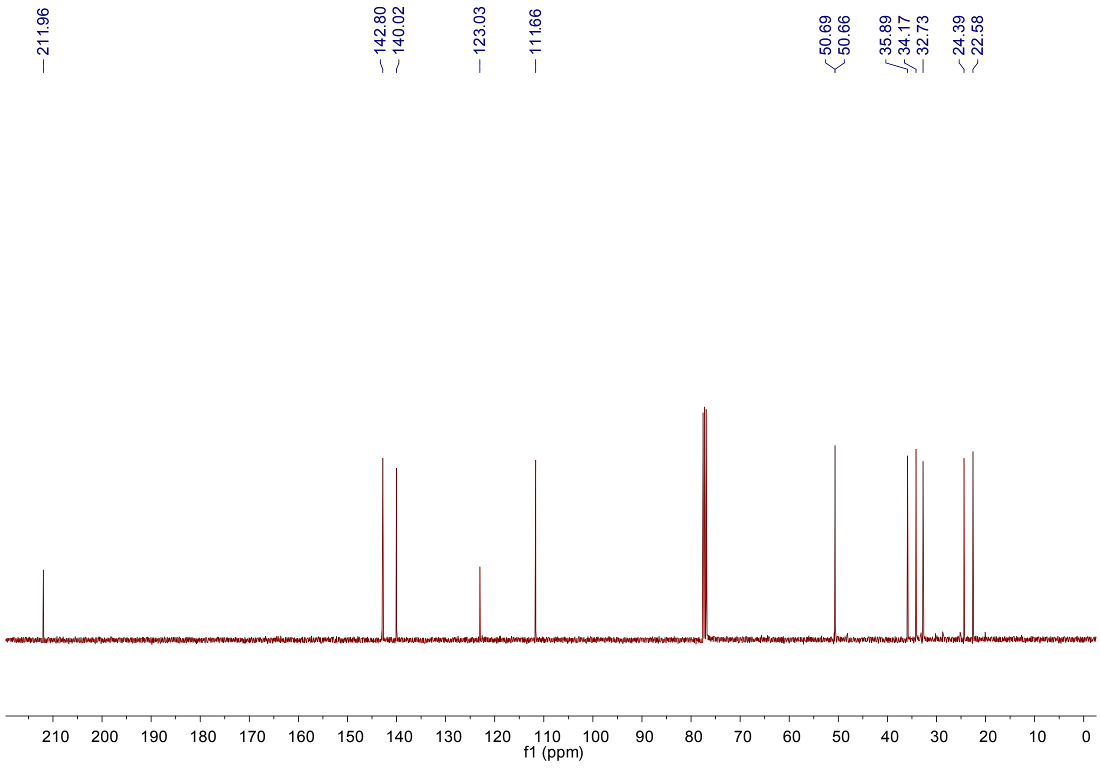

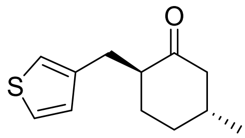

^{1}H NMR (400 MHz, $CDCl_3$) of compound **3o**

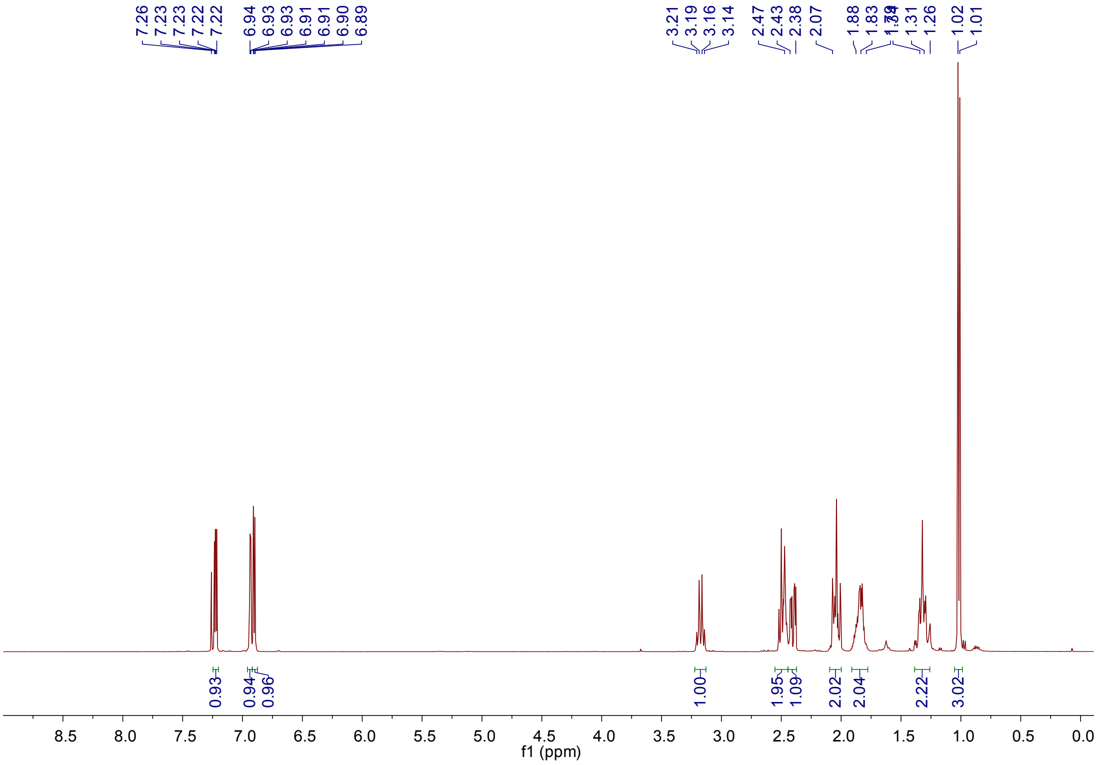

^{13}C NMR (100 MHz, $CDCl_3$) of compound **3o**

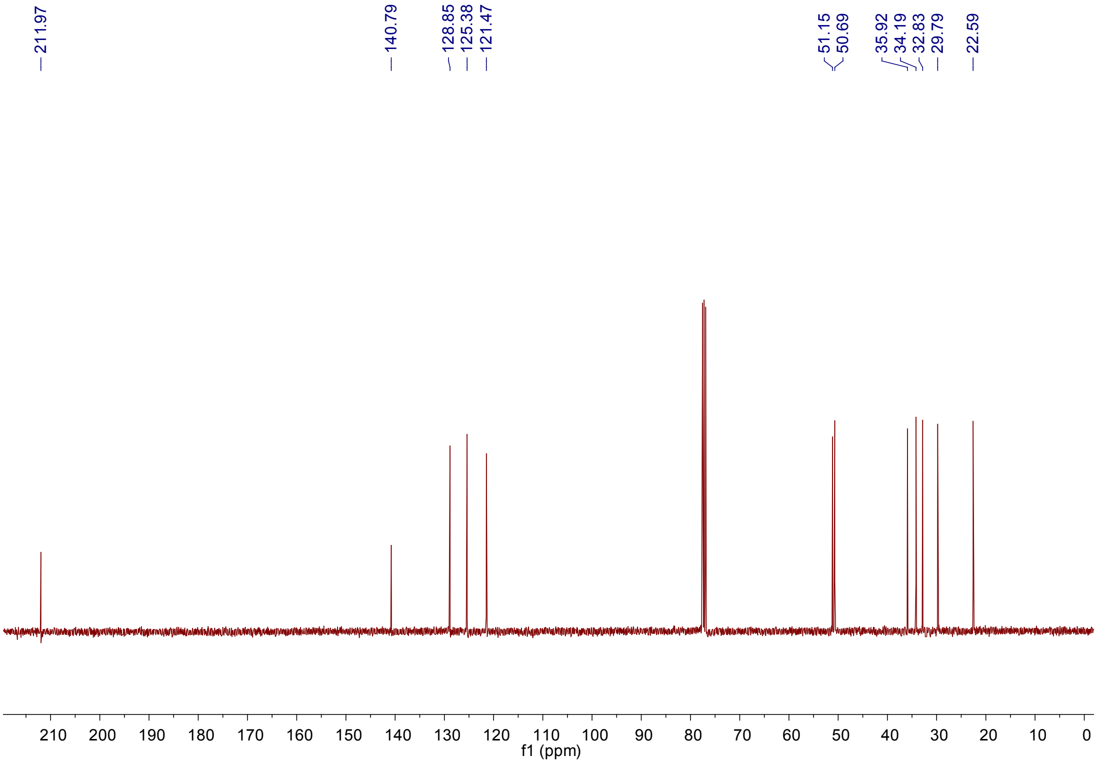

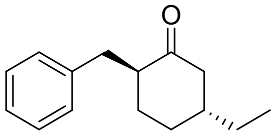

^{1}H NMR (400 MHz, $CDCl_3$) of compound **3g**

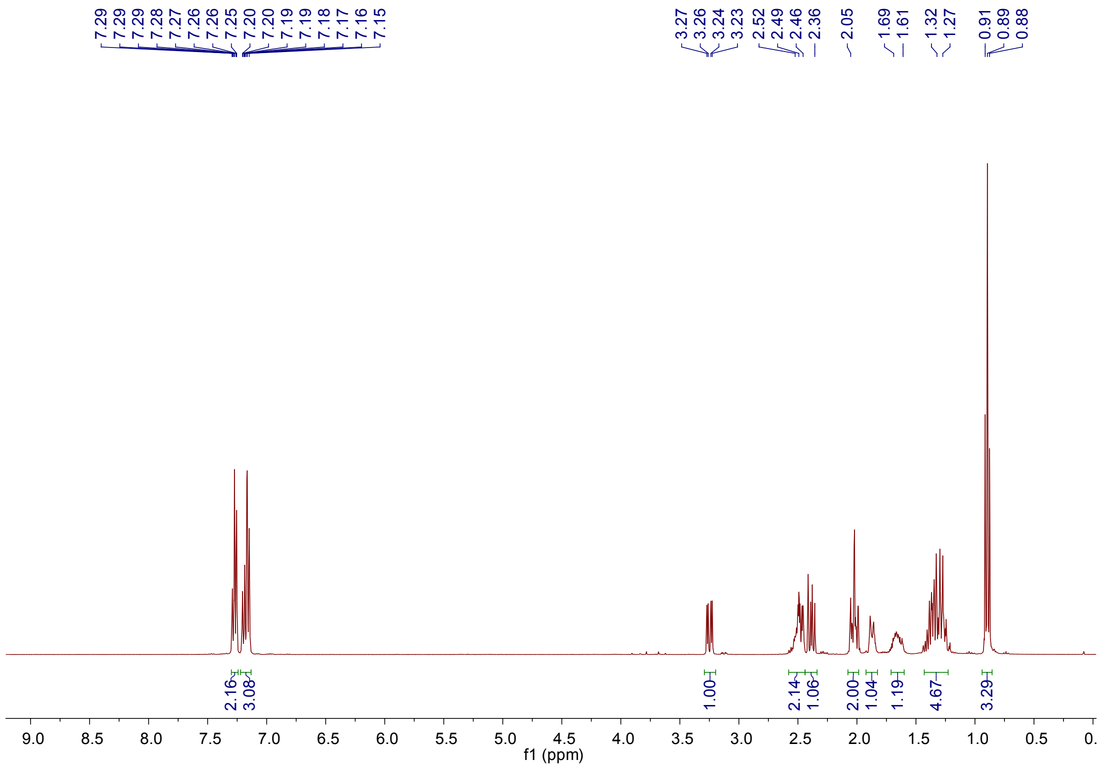

^{13}C NMR (100 MHz, $CDCl_3$) of compound **3g**

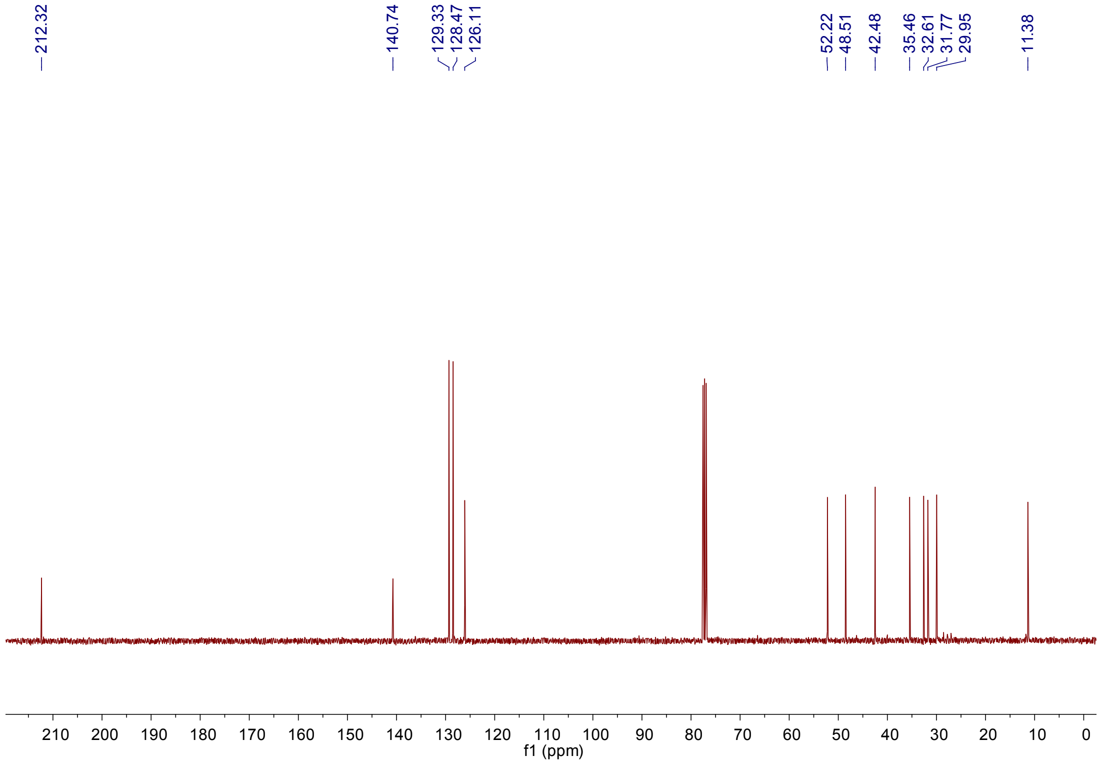

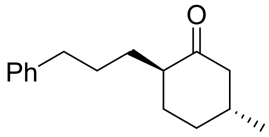

^{1}H NMR (400 MHz, $CDCl_3$) of compound **3i**

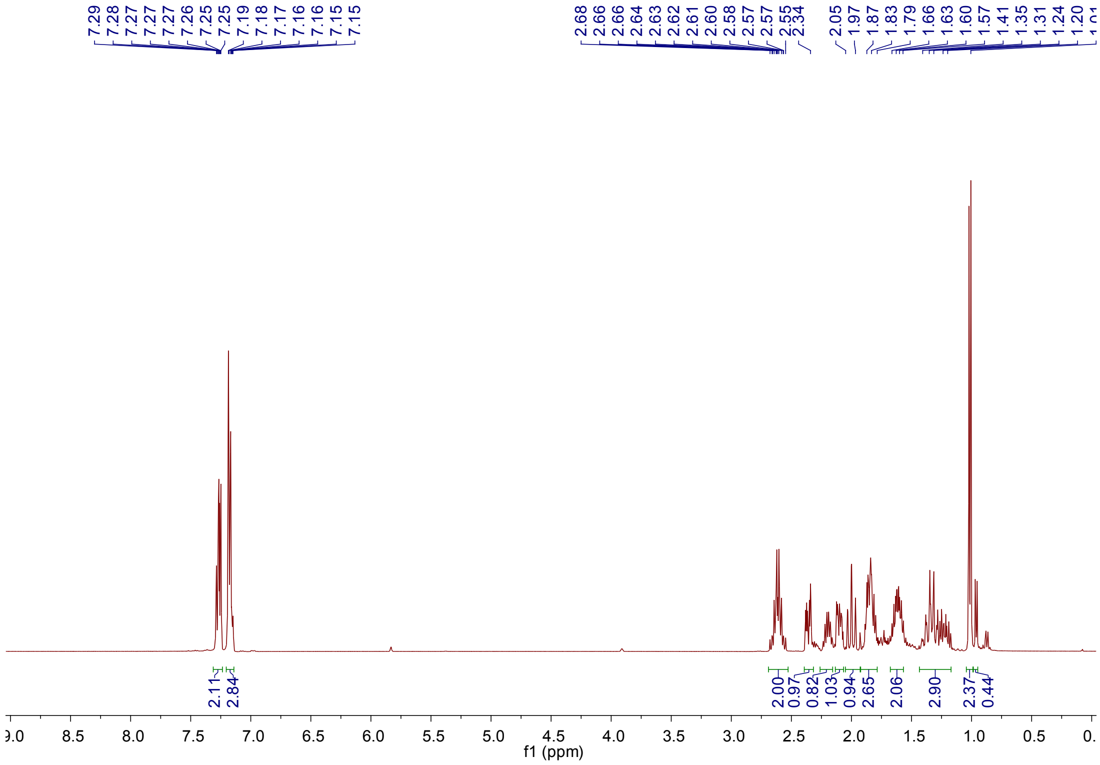

^{13}C NMR (100 MHz, $CDCl_3$) of compound **3i**

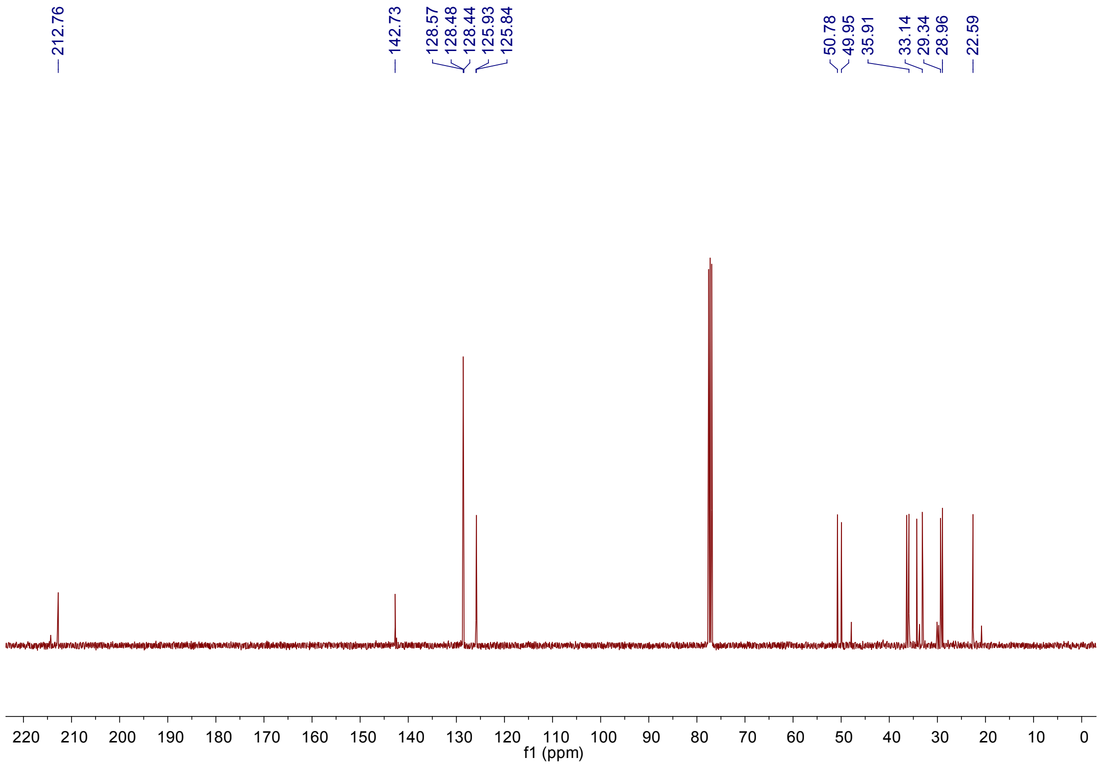

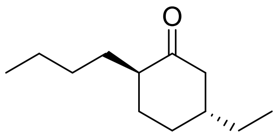

¹H NMR (400 MHz, $CDCl_3$) of compound **3m**

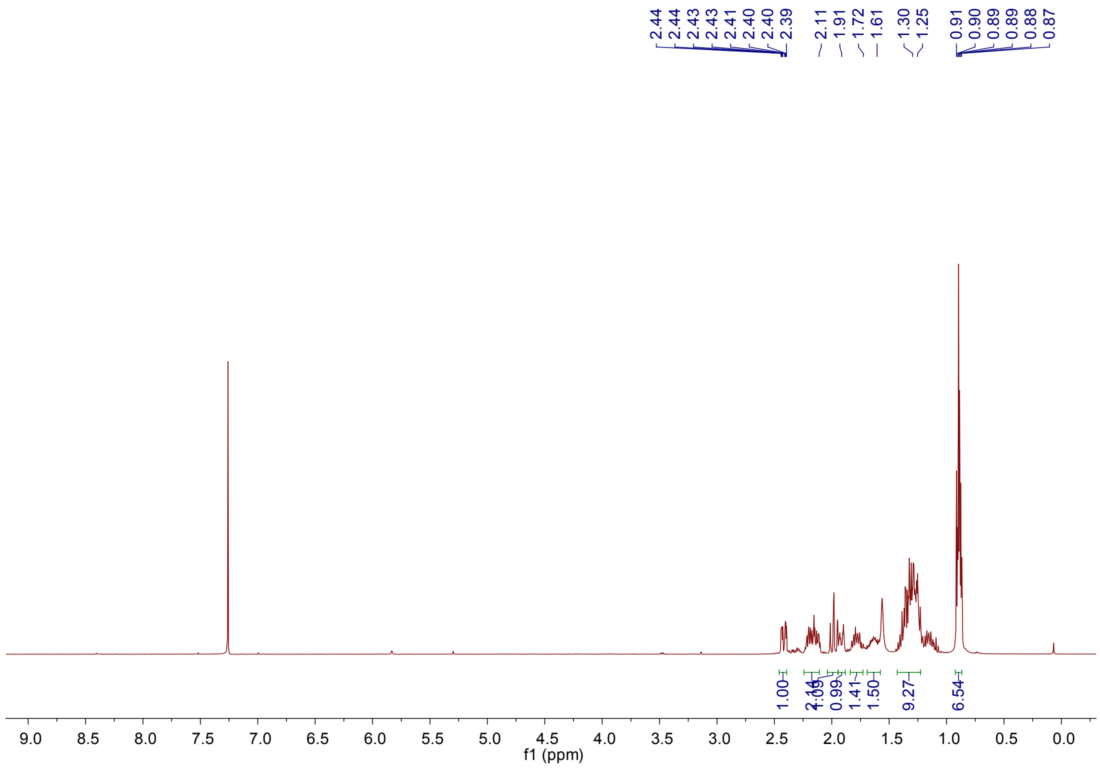

¹³C NMR (100 MHz, $CDCl_3$) of compound **3m**

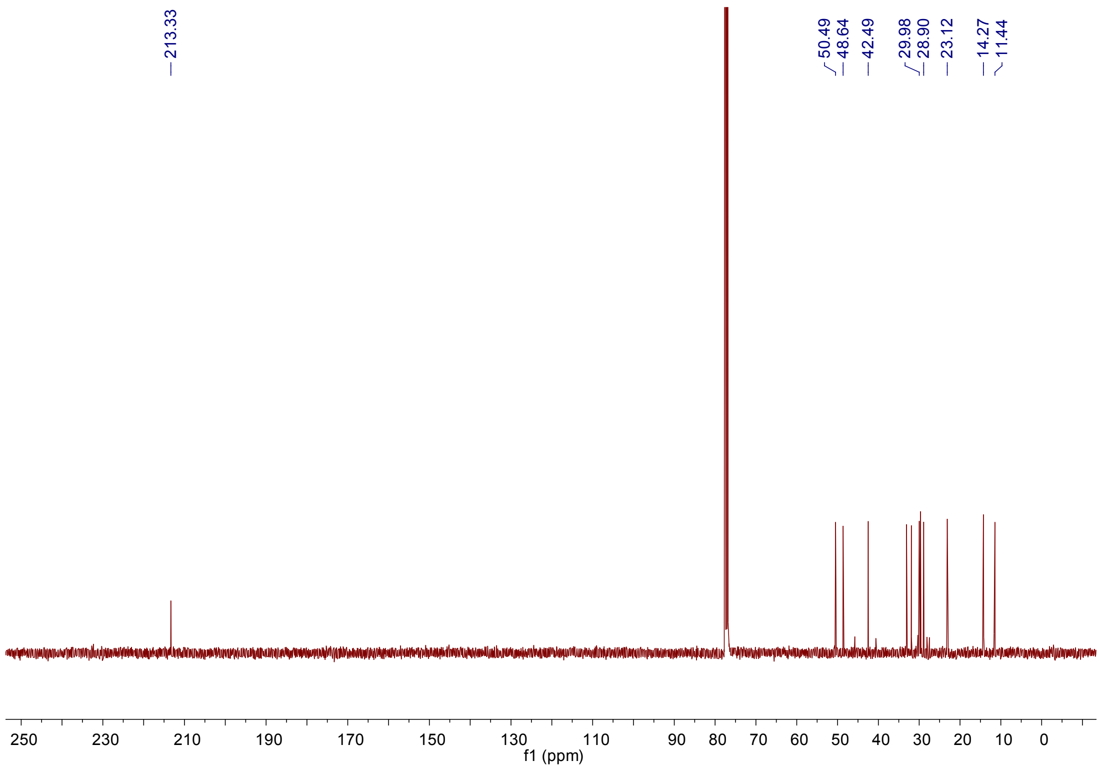

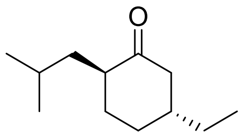

^{1}H NMR (400 MHz, $CDCl_3$) of compound **3p**

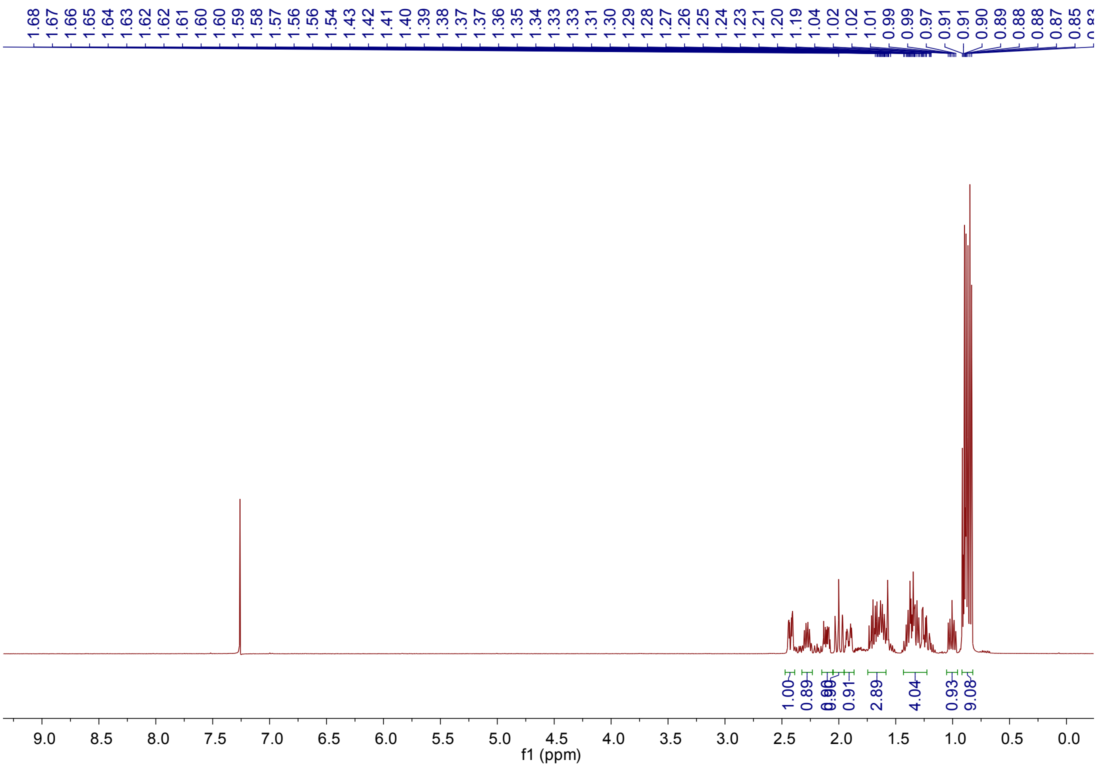

^{13}C NMR (100 MHz, $CDCl_3$) of compound **3p**

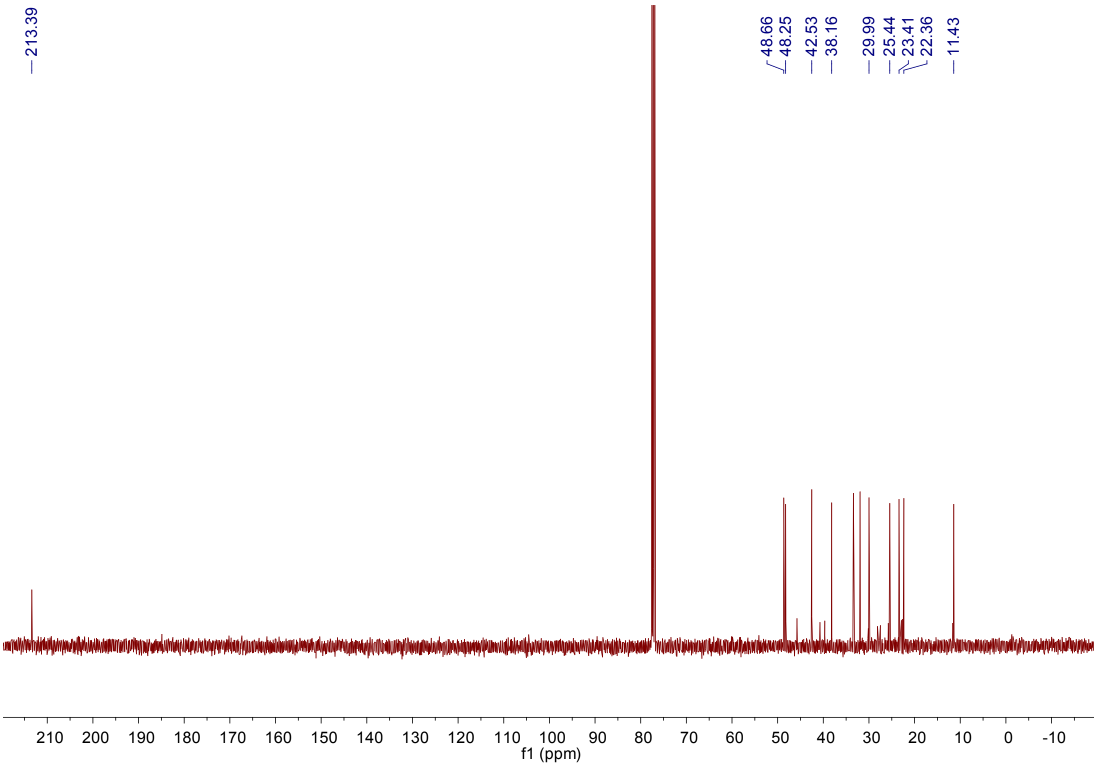

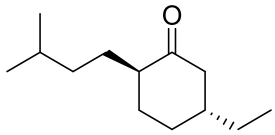

^{1}H NMR (400 MHz, $CDCl_3$) of compound **3q**

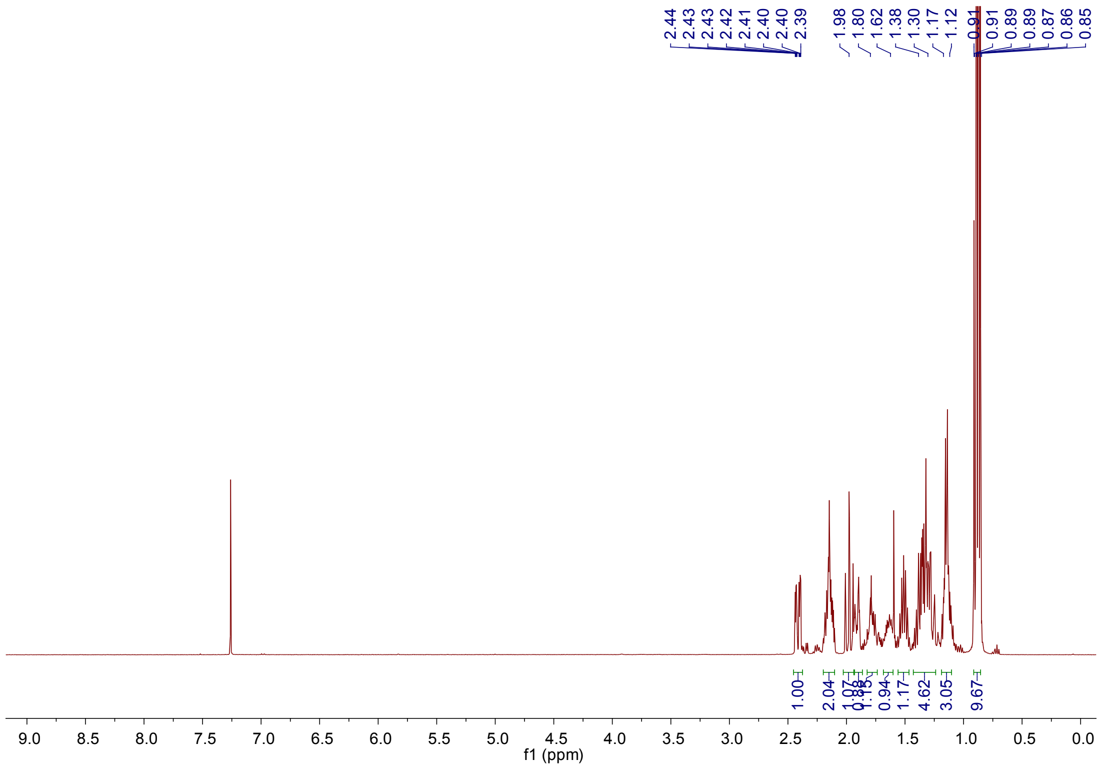

^{13}C NMR (100 MHz, $CDCl_3$) of compound **3q**

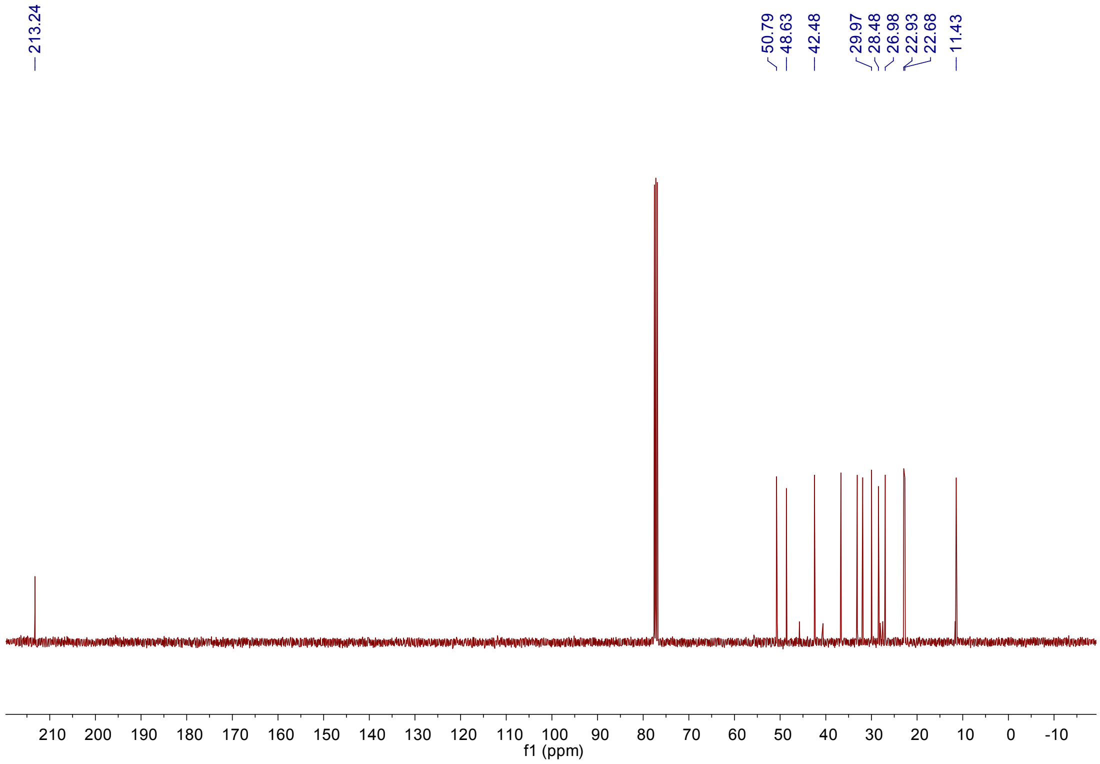

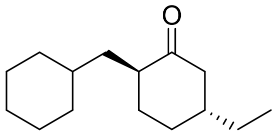

^{1}H NMR (400 MHz, $CDCl_3$) of compound **3r**

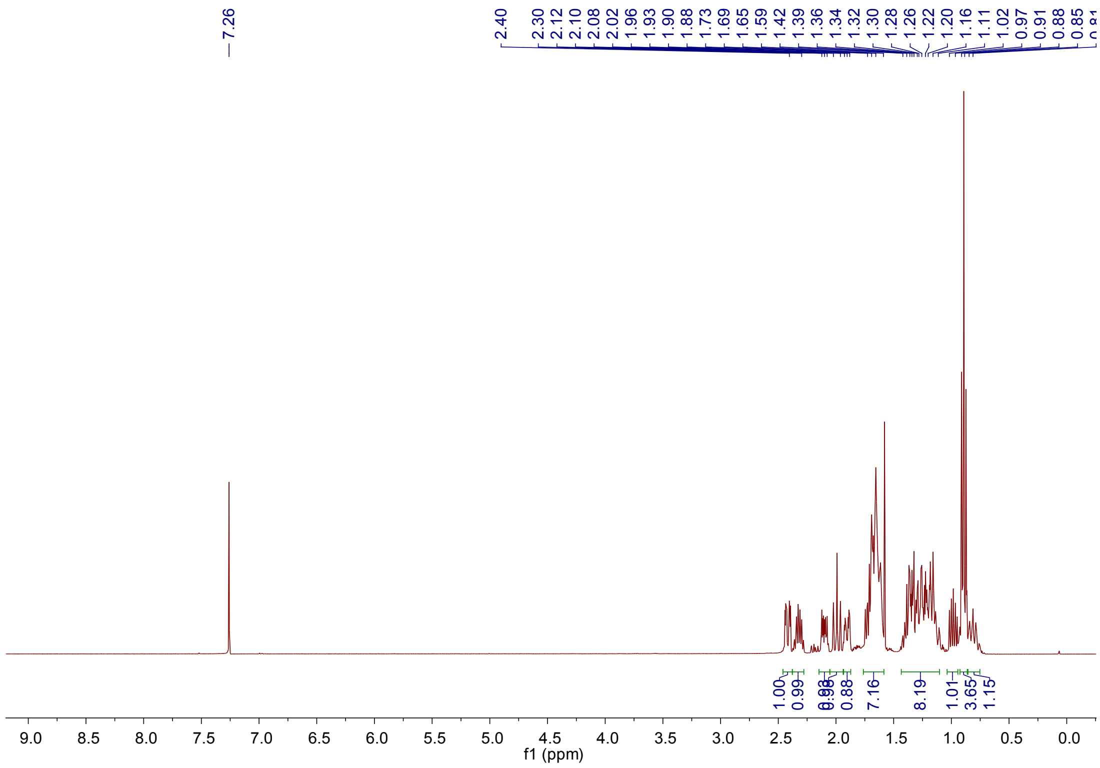

^{13}C NMR (100 MHz, $CDCl_3$) of compound **3r**

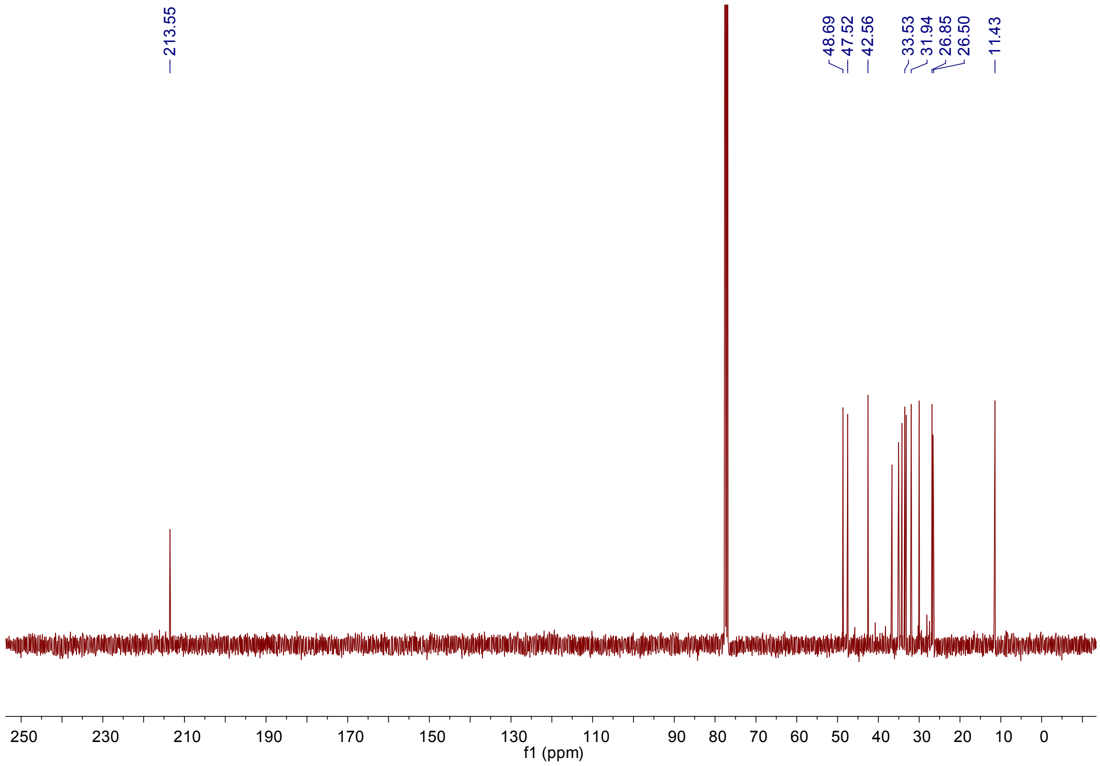

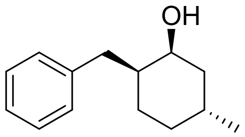

^{1}H NMR (400 MHz, $CDCl_3$) of compound **4a**

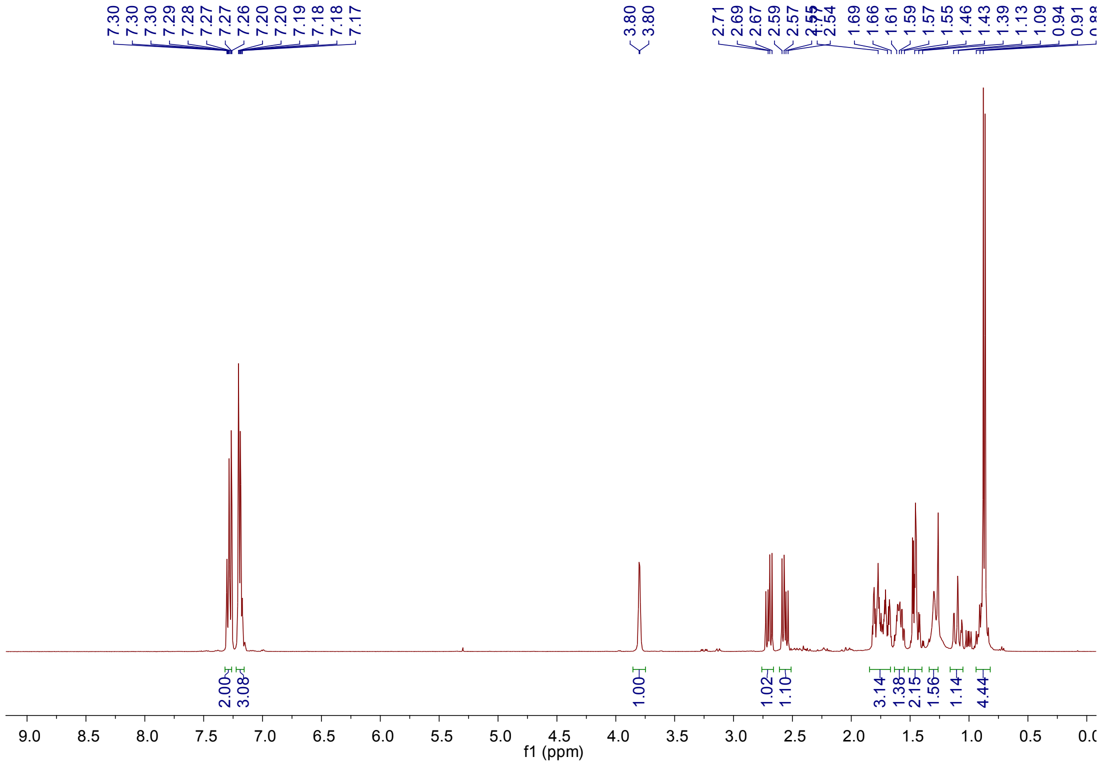

^{13}C NMR (100 MHz, $CDCl_3$) of compound **4a**

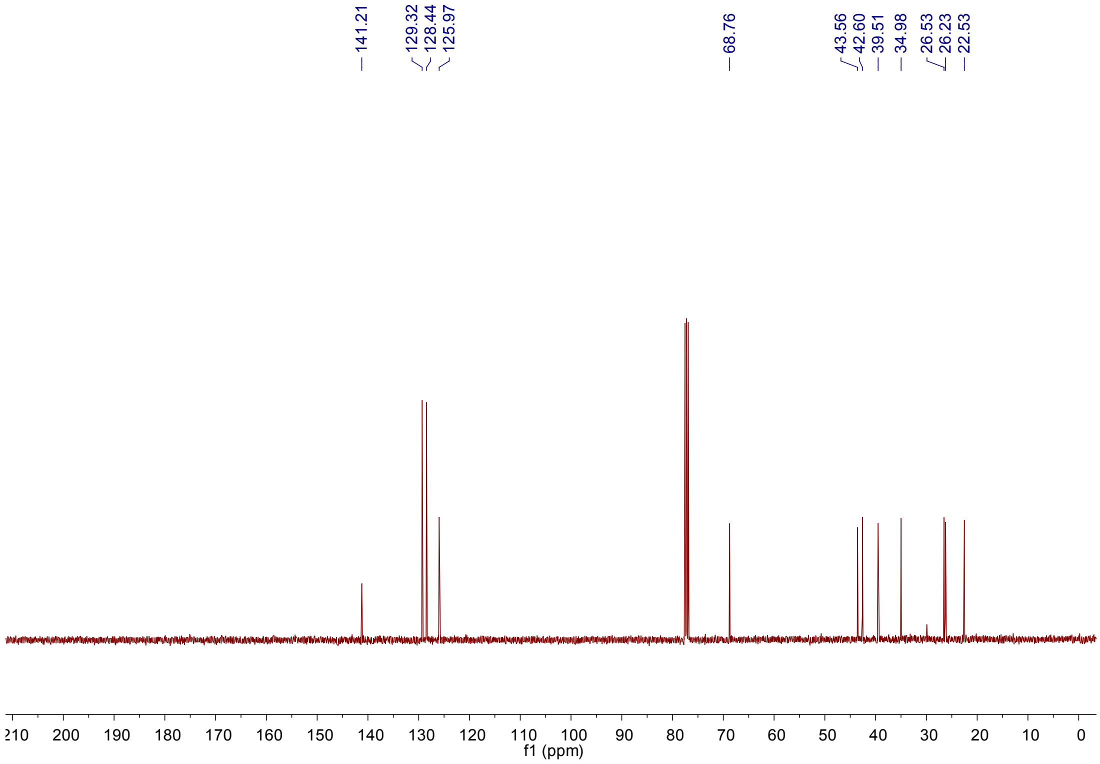

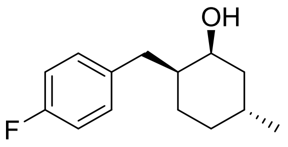

^{1}H NMR (400 MHz, $CDCl_3$) of compound **4b**

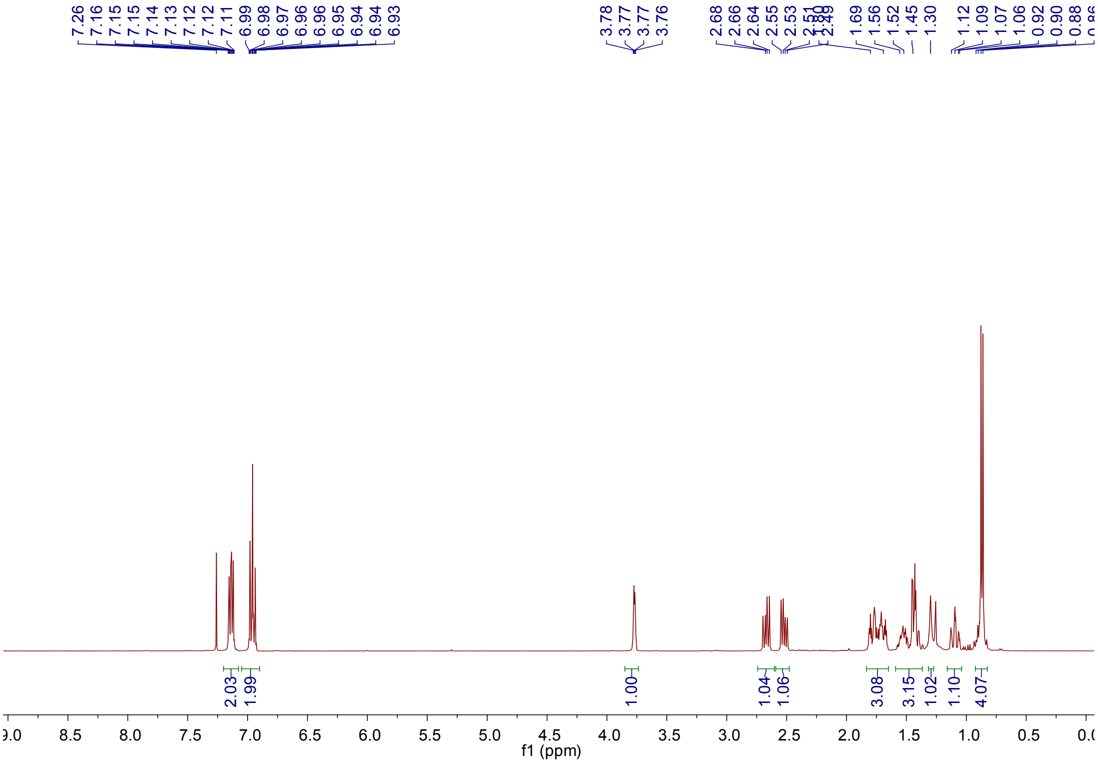

^{13}C NMR (100 MHz, $CDCl_3$) of compound **4b**

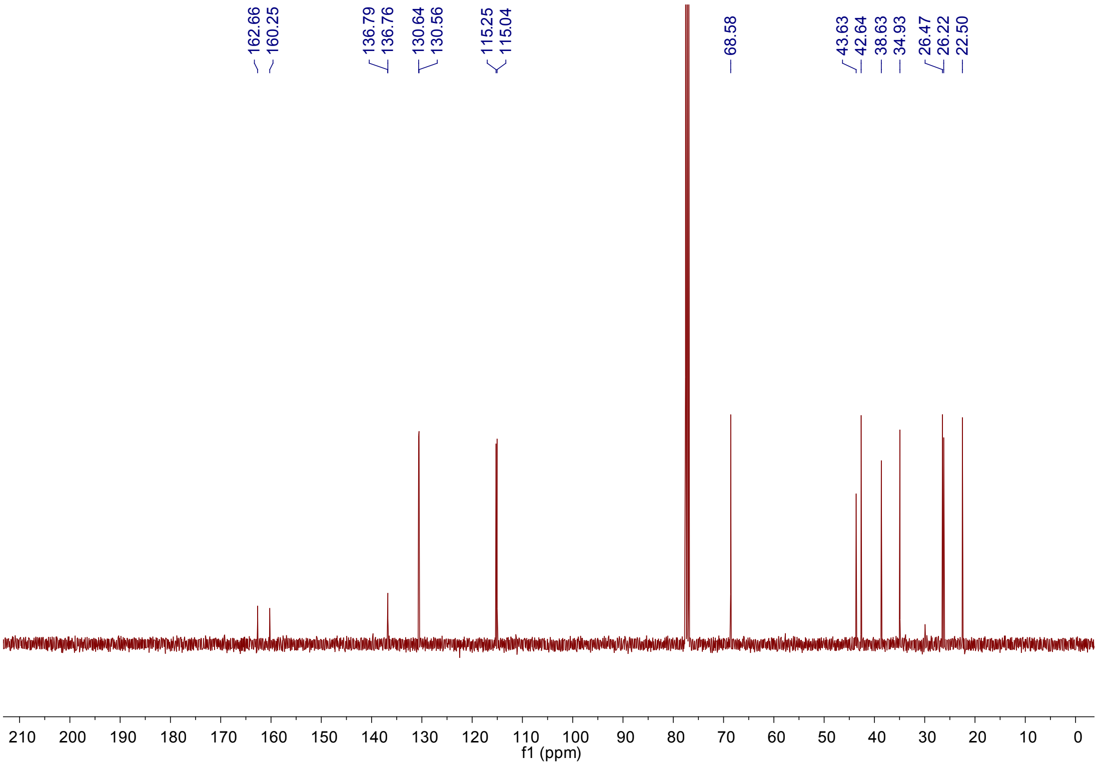

^{19}F NMR (377 MHz, $CDCl_3$) of compound **4b**

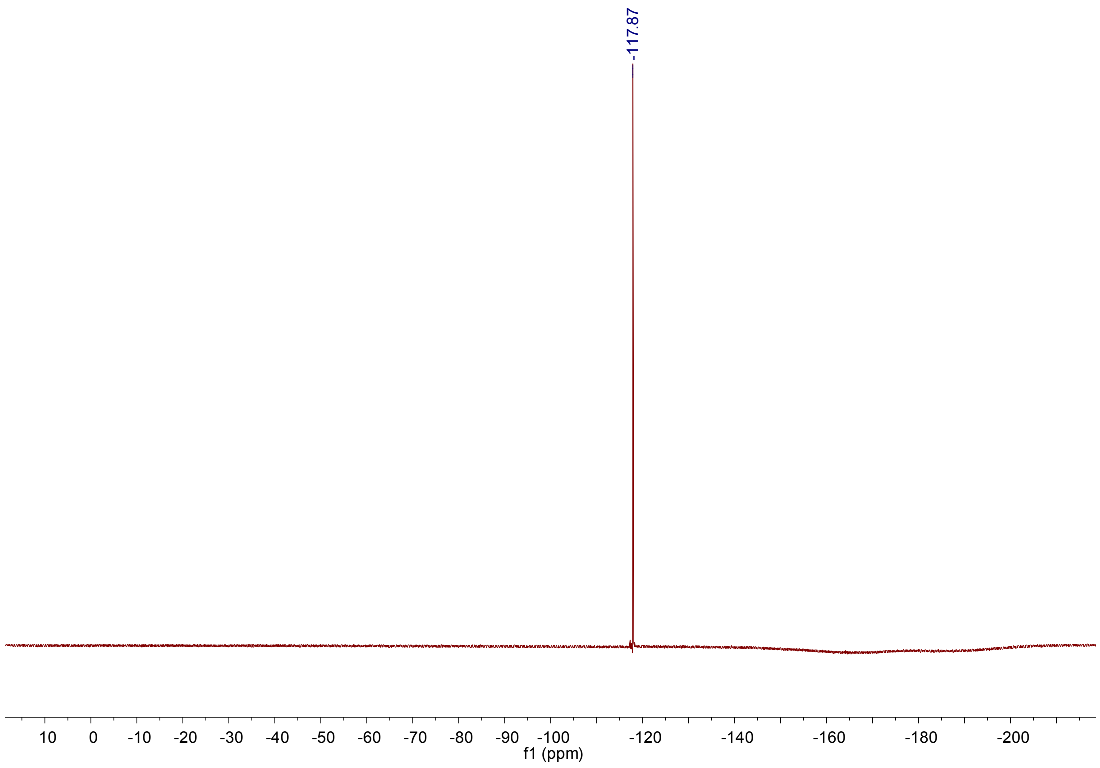

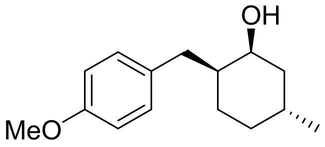

^{1}H NMR (400 MHz, $CDCl_3$) of compound **4n**

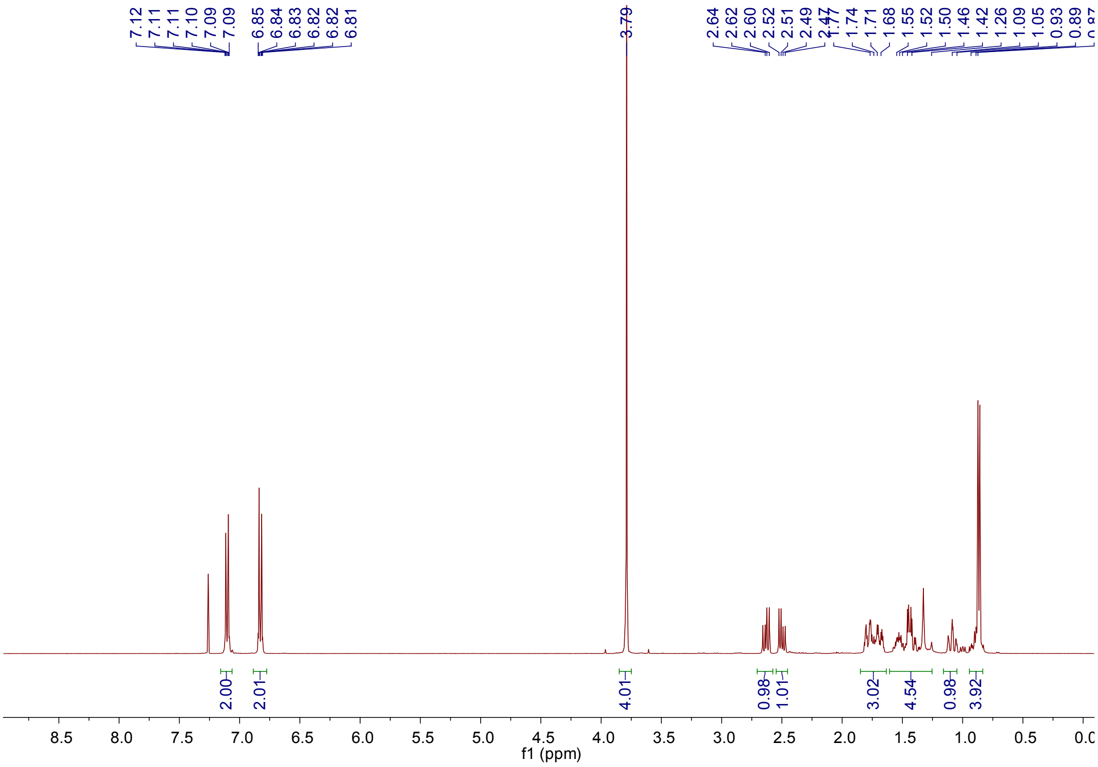

^{13}C NMR (100 MHz, $CDCl_3$) of compound **4n**

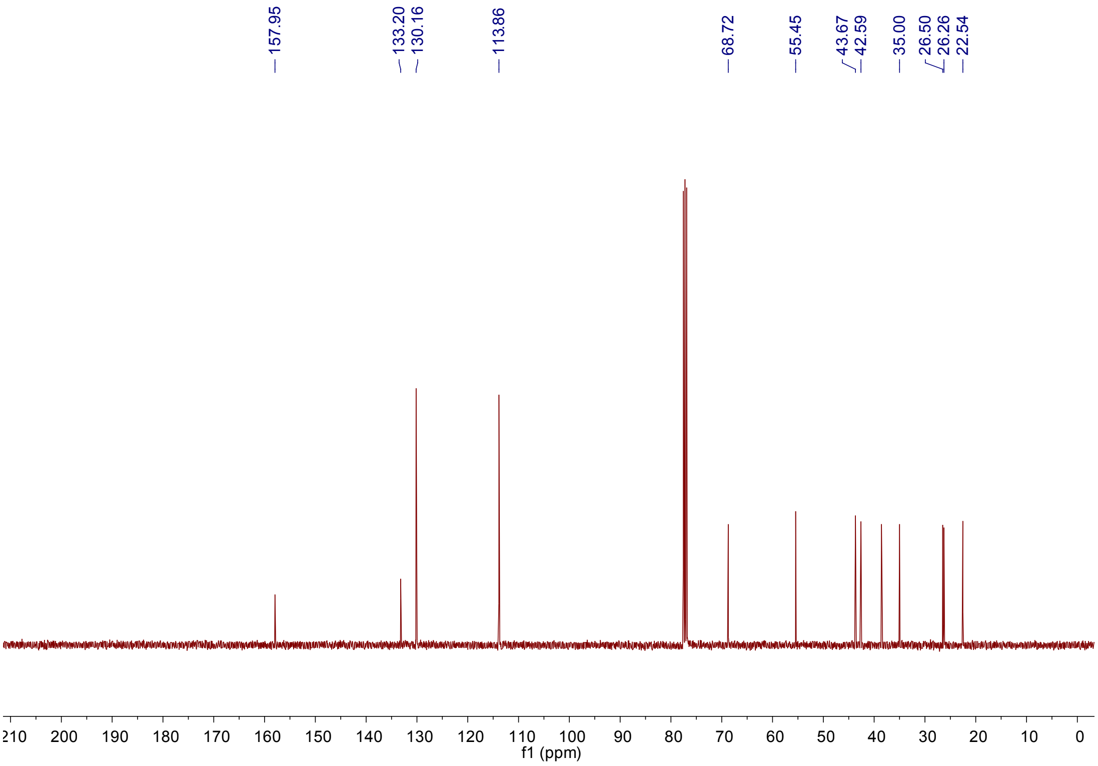

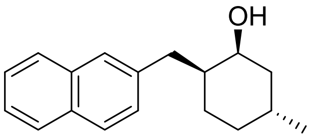

^{1}H NMR (400 MHz, $CDCl_3$) of compound **4e**

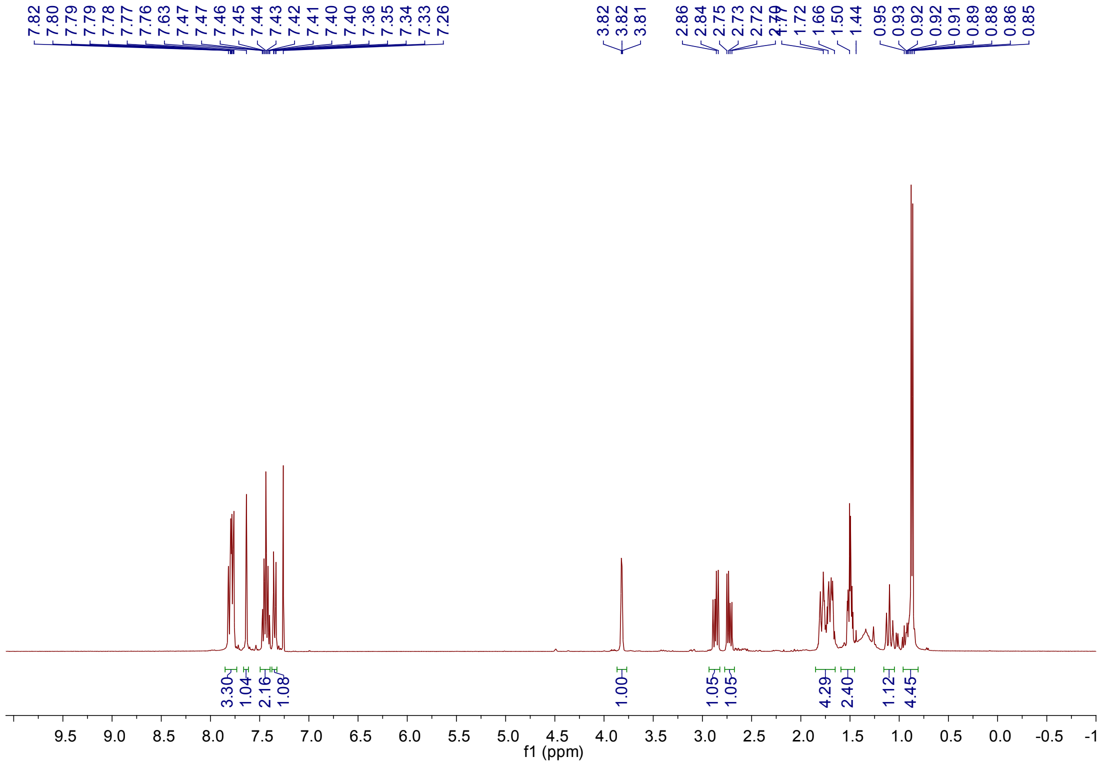

^{13}C NMR (100 MHz, $CDCl_3$) of compound **4e**

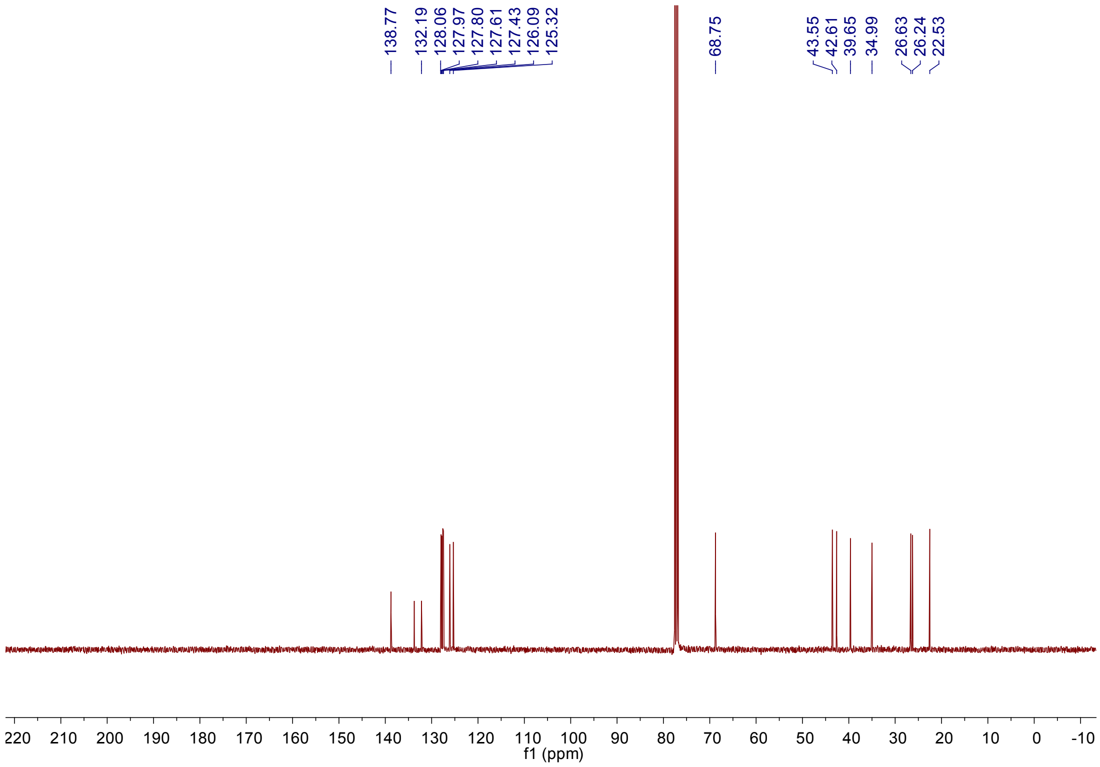

7. Separation of chiral products

Entry	Product	Separation method	Optical rotation	*ee* (%), d.r.
1	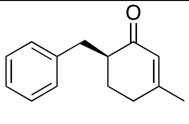	SFC: Chiralcel AS-H column (95% CO_2 5% MeOH), 2.0 mL/min, t_R = 9.0 min (minor) / 10.8 min (major)	$[\alpha]_D^{26}$ = +37.0 (c = 0.1, $CHCl_3$)	99
2	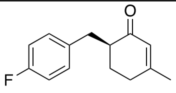	SFC: Chiralcel AS-H column (95% CO_2 5% MeOH), 2.0 mL/min, t_R = 8.0 min (minor) / 10.0 min (major)	$[\alpha]_D^{26}$ = +41.0 (c = 0.1, $CHCl_3$)	99
3	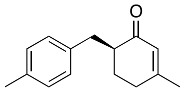	SFC: Chiralcel AS-H column (95% CO_2 5% MeOH), 2.0 mL/min, t_R = 9.4 min (minor) / 11.6 min (major)	$[\alpha]_D^{26}$ = +26.0 (c = 0.1, $CHCl_3$)	99
4	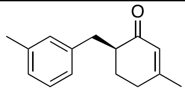	SFC: Chiralcel AS-H column (95% CO_2 5% MeOH), 2.0 mL/min, t_R = 7.9 min (minor) / 8.5 min (major)	$[\alpha]_D^{26}$ = +35.0 (c = 0.1, $CHCl_3$)	99
5	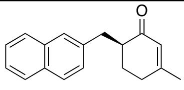	SFC: Chiralpak IB column (90% CO_2 10% MeOH), 2.0 mL/min, t_R = 26.1 min (minor) / 26.6 min (major)	$[\alpha]_D^{26}$ = +42.0 (c = 0.1, $CHCl_3$)	99
6	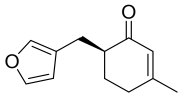	SFC: Chiralpak IA column (95% CO_2 5% MeOH), 2.0 mL/min, t_R = 8.3 min (major) / 8.8 min (minor)	$[\alpha]_D^{26}$ = +50.0 (c = 0.1, $CHCl_3$)	99
7	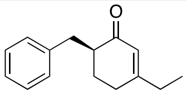	SFC: Chiralcel AS-H column (95% CO_2 5% MeOH), 2.0 mL/min, t_R = 9.5 min (minor) / 12.3 min (major)	$[\alpha]_D^{26}$ = +32.0 (c = 0.1, $CHCl_3$)	99
8	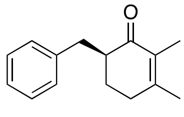	SFC: Chiralcel OJ-H column (90% CO_2 10% MeOH), 2.0 mL/min, t_R = 5.0 min (minor) / 5.5 min (major)	$[\alpha]_D^{26}$ = +49.0 (c = 0.1, $CHCl_3$)	99
9	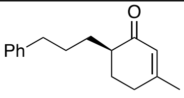	SFC: Chiralcel OJ-H column (95% CO_2 5% MeOH), 2.0 mL/min, t_R = 9.5 min (major) / 10.9 min (minor)	$[\alpha]_D^{26}$ = +34.0 (c = 0.1, $CHCl_3$)	99
10	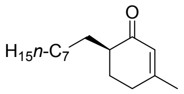	SFC: Chiralcel AS-H column (95% CO_2 5% MeOH), 2.0 mL/min, t_R = 5.4 min (minor) / 5.9 min (major)	$[\alpha]_D^{26}$ = +32.0 (c = 0.1, $CHCl_3$)	99
11	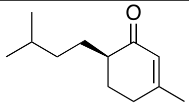	SFC: Chiralcel AD-H column (95% CO_2 5% MeOH), 2.0 mL/min, t_R = 6.7 min (minor) / 7.2 min (major)	$[\alpha]_D^{26}$ = +33.0 (c = 0.1, $CHCl_3$)	94
12	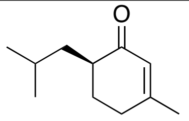	SFC: Chiralcel AD-H column (95% CO_2 5% MeOH), 2.0 mL/min, t_R = 5.5 min (minor) / 6.0 min (major)	$[\alpha]_D^{26}$ = +15.0 (c = 0.1, $CHCl_3$)	94

13	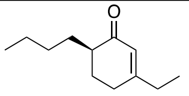	SFC: Chiralcel AD-H column (95% CO_2 5% MeOH), 2.0 mL/min, t_R = 6.5 min (minor) / 7.1 min (major)	$[\alpha]_D^{26}$ = +35.0 (c = 0.1, $CHCl_3$)	97
14	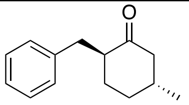	GC: Chiraldex β-DM column (50 to 170 °C, 1 °C/min, 1.0 mL/min), t_R = 85.0 min (major) / 85.5 min (minor) / 86.1 min (minor) / 87.2 min (minor)	$[\alpha]_D^{26}$ = -27.0 (c = 0.1, $CHCl_3$)	99, 99/1
15	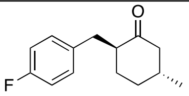	GC: Chiraldex β-DM column (50 to 170 °C, 1 °C/min, 1.0 mL/min), t_R = 86.5 min (minor) / 87.0 min (major) / 87.9 min (minor) / 89.1 min (minor)	$[\alpha]_D^{26}$ = -23.0 (c = 0.1, $CHCl_3$)	99, 99/1
16	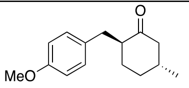	SFC: Chiralcel AS-H column (95% CO_2 5% MeOH), 2.0 mL/min, t_R = 4.7 min (major) / 5.0 min (minor) / 5.2 min (minor) / 6.0 min (minor)	$[\alpha]_D^{26}$ = -26.0 (c = 0.1, $CHCl_3$)	99, 99/1
17	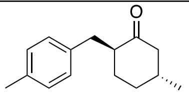	GC: Chiraldex β-DM column (50 to 170 °C, 1 °C/min, 1.0 mL/min), t_R = 95.0 min (minor) / 95.5 min (major) / 96.4 min (minor) / 96.8 min (minor)	$[\alpha]_D^{26}$ = -23.0 (c = 0.1, $CHCl_3$)	99, 99/1
18	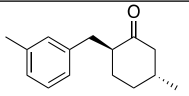	SFC: Chiralcel AY-H column (90% CO_2 10% MeOH), 2.0 mL/min, t_R = 3.6 min (major) / 3.7 min (minor) / 3.9 min (minor)	$[\alpha]_D^{26}$ = -21.0 (c = 0.1, $CHCl_3$)	99, 99/1
19	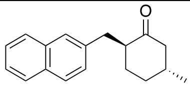	SFC: Chiralcel AS-H column (95% CO_2 5% MeOH), 2.0 mL/min, t_R = 9.4 min (major) / 10.5 min (minor) / 11.3 min (minor) / 13.6 min (minor)	$[\alpha]_D^{26}$ = -14.0 (c = 0.1, $CHCl_3$)	99, 99/1
20	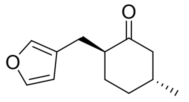	SFC: Chiralcel AY-H column (95% CO_2 5% MeOH), 2.0 mL/min, t_R = 4.0 min (major) / 4.4 min (minor) / 4.7 min (minor) / 5.2 min (minor)	$[\alpha]_D^{26}$ = −10.0 (c = 0.1, $CHCl_3$)	99, 97/3
21	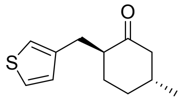	SFC: Chiralcel AY-H column (95% CO_2 5% MeOH), 2.0 mL/min, t_R = 7.7 min (major) / 8.9 min (minor) / 9.5 min (minor) / 10.6 min (minor)	$[\alpha]_D^{26}$ = -21.0 (c = 0.1, $CHCl_3$)	99, 99/1
22	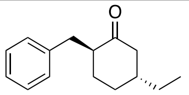	GC: Chiraldex β-DM column (50 to 170 °C, 1 °C/min, 1.0 mL/min), t_R = 95.1 min (minor) / 95.9 min (minor) / 97.0 min (minor) / 97.6 min (major)	$[\alpha]_D^{26}$ = -21.0 (c = 0.1, $CHCl_3$)[2]	99, 99/1
23	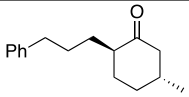	SFC: Chiralcel OJ-H column (98% CO_2 2% MeOH), 2.0 mL/min, t_R = 19.2 min (major) /	$[\alpha]_D^{26}$ = +3.0 (c = 0.1, $CHCl_3$)	99, 90/10

		19.6 min (minor) / 21.1 min (minor) / 22.0 min (minor)		
24	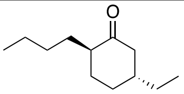	GC: Chiraldex β-DM column (50 to 170 °C, 1 °C/min, 1.0 mL/min), t_R = 51.1 min (minor) / 52.2 min (minor) / 53.1 min (minor) / 53.6 min (major)	$[\alpha]_D^{26}$ = +4.0 (c = 0.1, $CHCl_3$)	99, 97/3
25	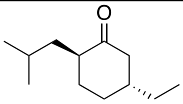	GC: Chiraldex β-DM column (50 to 170 °C, 1 °C/min, 1.0 mL/min), t_R = 43.9 min (minor) / 44.4 min (minor) / 45.4 min (minor) / 45.9 min (major)	$[\alpha]_D^{26}$ = -5.0 (c = 0.1, $CHCl_3$)	98, 93/7
26	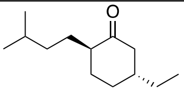	GC: Chiraldex β-DM column (50 to 170 °C, 1 °C/min, 1.0 mL/min), t_R = 58.1 min (minor) / 58.5 min (minor) / 60.2 min (minor) / 60.6 min (major)	$[\alpha]_D^{26}$ = +3.0 (c = 0.1, $CHCl_3$)	99, 95/5
27	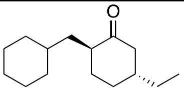	GC: Chiraldex β-DM column (50 to 170 °C, 1 °C/min, 1.0 mL/min), t_R = 89.8 min (minor) / 90.3 min (minor) / 91.3 min (minor) / 92.0 min (major)	$[\alpha]_D^{26}$ = -7.0 (c = 0.1, $CHCl_3$)	99, 97/3
28	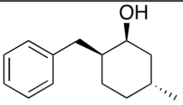	*ee*<br>SFC: Chiralcel OJ-H column (80% CO_2 20% MeOH), 2.0 mL/min, t_R = 3.4 min (major) / 3.6 min (minor)<br>d.r.<br>Determined by ^{1}H NMR spectroscopy	$[\alpha]_D^{26}$ = +26.0 (c = 0.1, $CHCl_3$)	99, >95/5
29	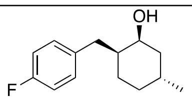	*ee*<br>SFC: Chiralcel AD-H column (80% CO_2 20% MeOH), 2.0 mL/min, t_R = 4.1 min (major) / 4.5 min (minor)<br>d.r.<br>Determined by ^{1}H NMR spectroscopy	$[\alpha]_D^{26}$ = +23.0 (c = 0.1, $CHCl_3$)	99, >95/5
30	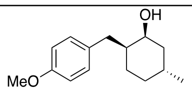	*ee*<br>SFC: Chiralcel OJ-H column (80% CO_2 20% MeOH), 2.0 mL/min, t_R = 3.8 min (major) / 4.0 min (minor)<br>d.r.<br>Determined by ^{1}H NMR spectroscopy	$[\alpha]_D^{26}$ = +20.0 (c = 0.1, $CHCl_3$)	99, >95/5
31	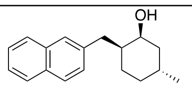	*ee*<br>SFC: Chiralpak IA column (90% CO_2 10% MeOH), 2.0 mL/min, t_R = 13.9 min (minor)/ 16.0 min (major).	$[\alpha]_D^{26}$ = +25.0 (c = 0.1, $CHCl_3$)	99, >95/5

		d.r.<br>Determined by ^{1}H NMR spectroscopy		

8. Chromatograms

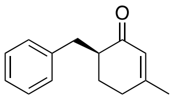

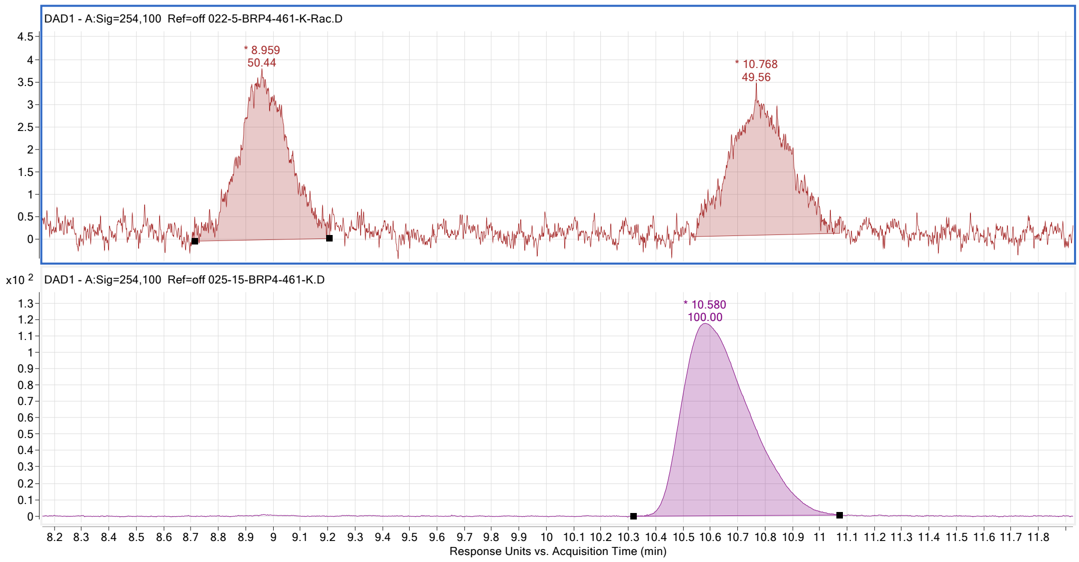

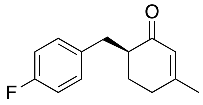

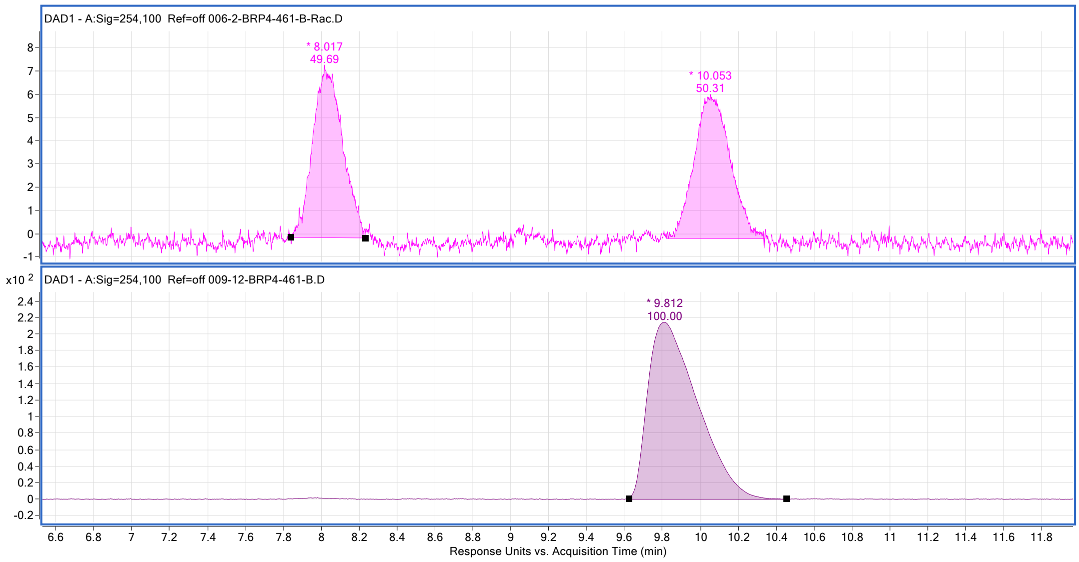

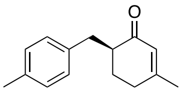

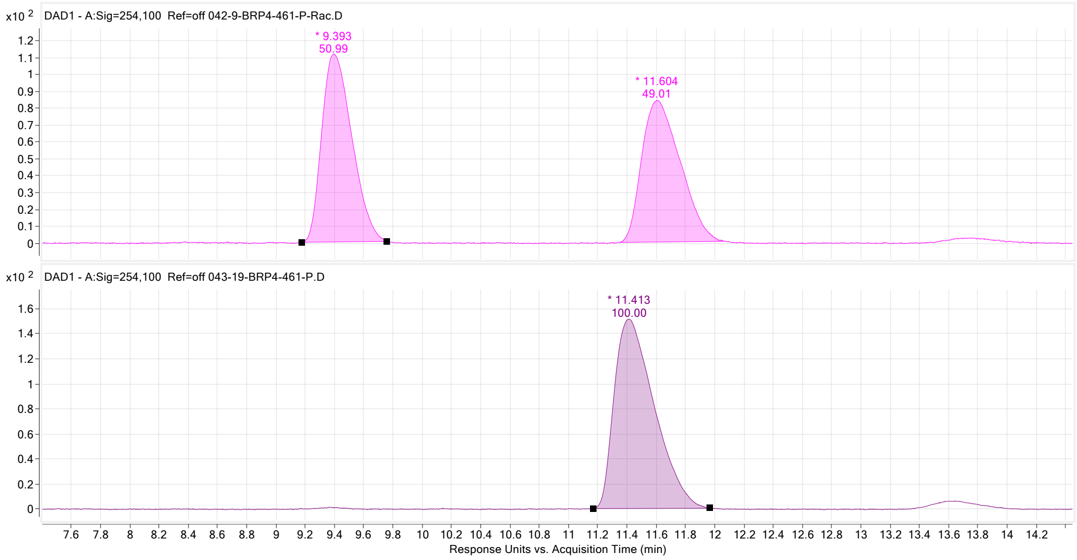
x10 2 DAD1 - A:Sig=254,100 Ref=off 042-9-BRP4-461-P-Rac.D
* 9.393
50.99
* 11.604
49.01
x10 2 DAD1 - A:Sig=254,100 Ref=off 043-19-BRP4-461-P.D
* 11.413
100.00
Response Units vs. Acquisition Time (min)

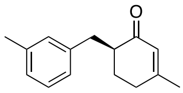

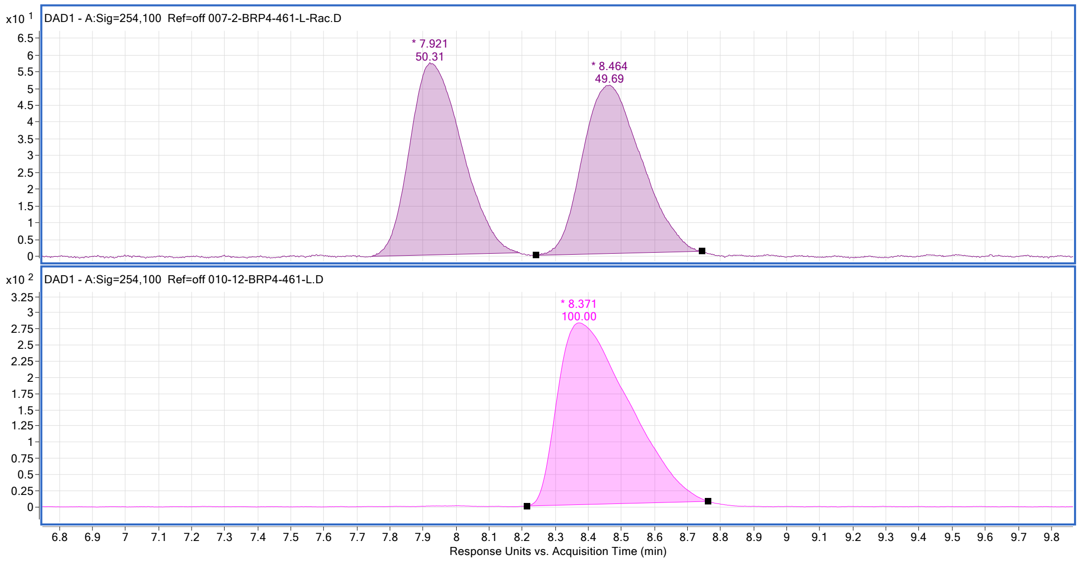
x10 1 DAD1 - A:Sig=254,100 Ref=off 007-2-BRP4-461-L-Rac.D
* 7.921
50.31
* 8.464
49.69
x10 2 DAD1 - A:Sig=254,100 Ref=off 010-12-BRP4-461-L.D
* 8.371
100.00
Response Units vs. Acquisition Time (min)

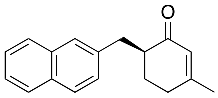
O

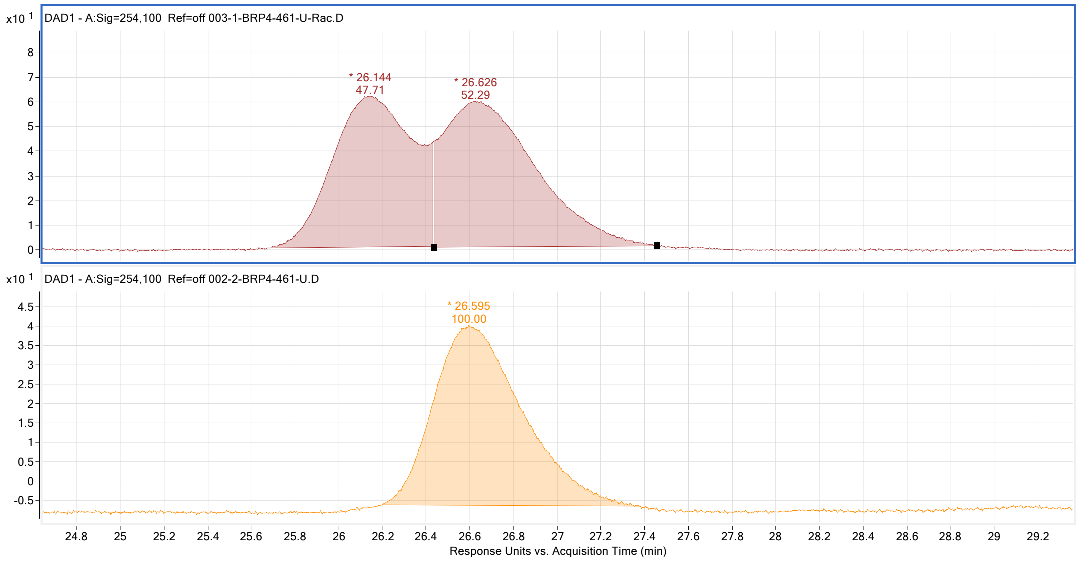
x10 1
DAD1 - A:Sig=254,100 Ref=off 003-1-BRP4-461-U-Rac.D
* 26.144
47.71
* 26.626
52.29
x10 1
DAD1 - A:Sig=254,100 Ref=off 002-2-BRP4-461-U.D
* 26.595
100.00
Response Units vs. Acquisition Time (min)

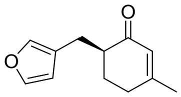
O
O

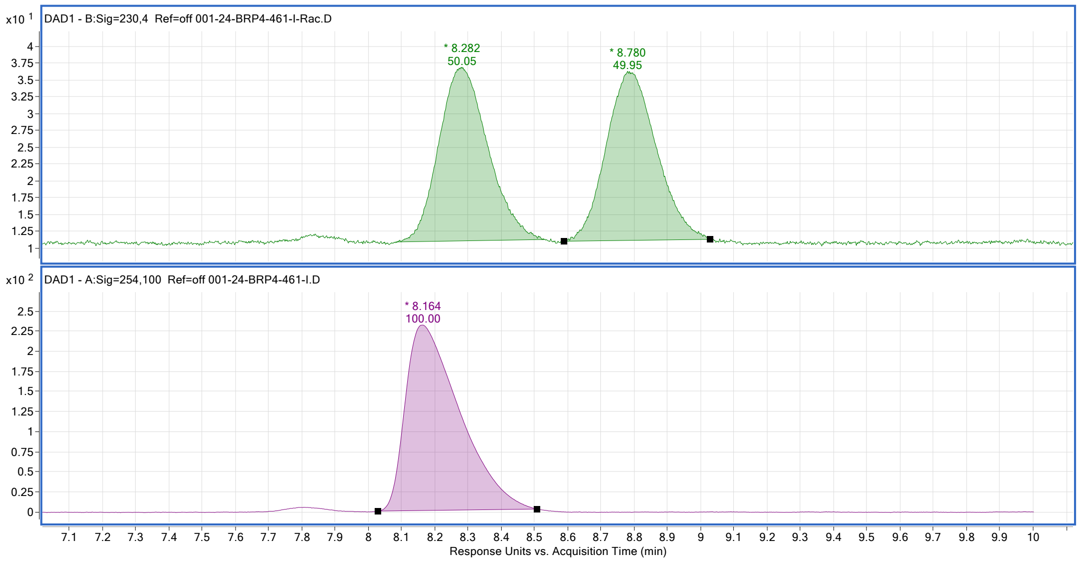
x10 1
DAD1 - B:Sig=230,4 Ref=off 001-24-BRP4-461-I-Rac.D
* 8.282
50.05
* 8.780
49.95
x10 2
DAD1 - A:Sig=254,100 Ref=off 001-24-BRP4-461-I.D
* 8.164
100.00
Response Units vs. Acquisition Time (min)

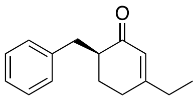

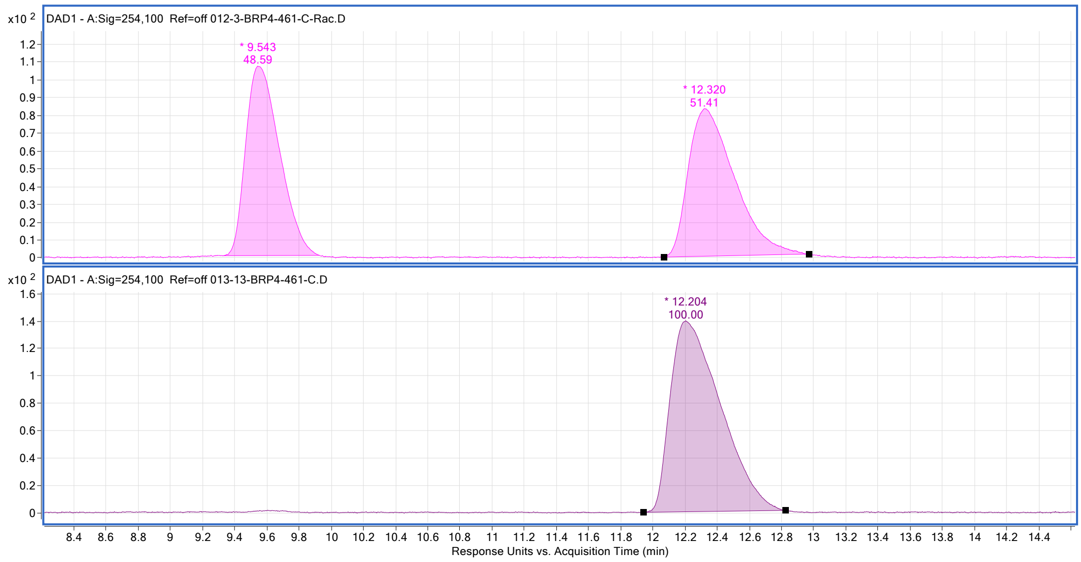
x10 2
DAD1 - A:Sig=254,100 Ref=off 012-3-BRP4-461-C-Rac.D
* 9.543
48.59
* 12.320
51.41
x10 2
DAD1 - A:Sig=254,100 Ref=off 013-13-BRP4-461-C.D
* 12.204
100.00
Response Units vs. Acquisition Time (min)

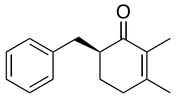

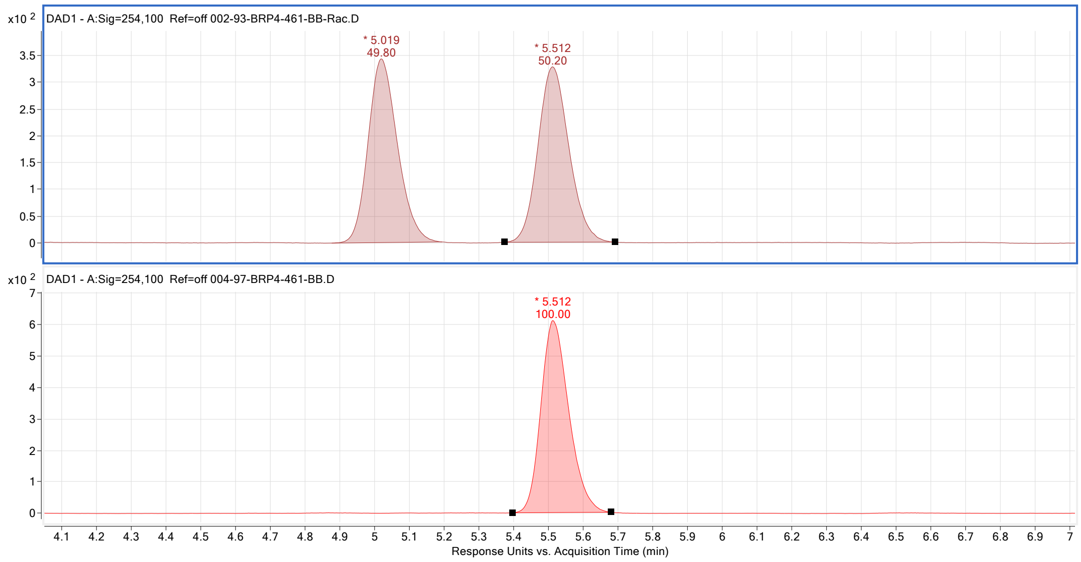
x10 2
DAD1 - A:Sig=254,100 Ref=off 002-93-BRP4-461-BB-Rac.D
* 5.019
49.80
* 5.512
50.20
x10 2
DAD1 - A:Sig=254,100 Ref=off 004-97-BRP4-461-BB.D
* 5.512
100.00
Response Units vs. Acquisition Time (min)

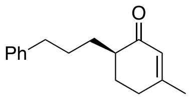
O
Ph

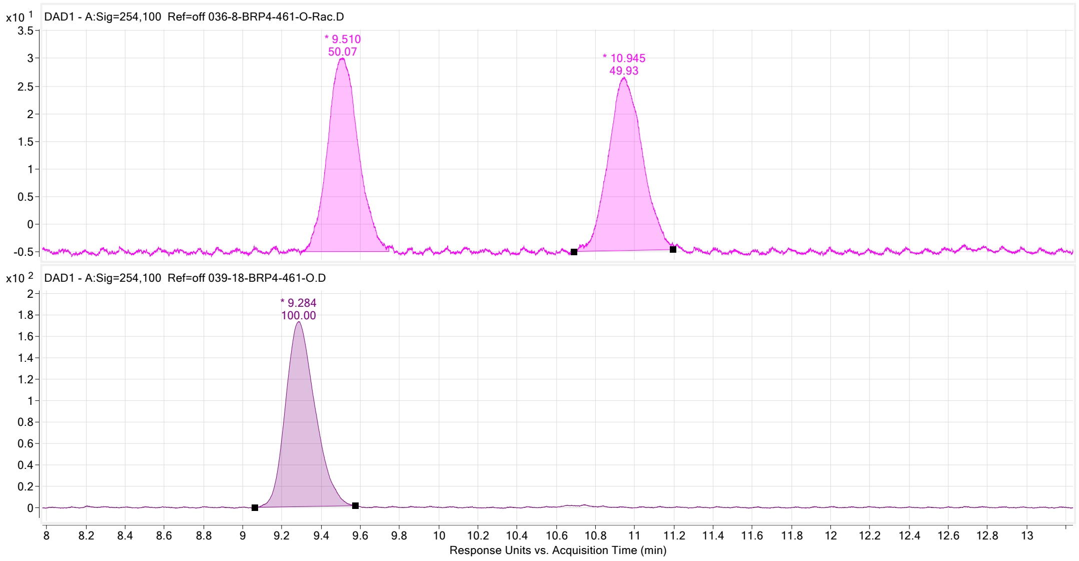
x10 1
DAD1 - A:Sig=254,100 Ref=off 036-8-BRP4-461-O-Rac.D
* 9.510
50.07
* 10.945
49.93
x10 2
DAD1 - A:Sig=254,100 Ref=off 039-18-BRP4-461-O.D
* 9.284
100.00
Response Units vs. Acquisition Time (min)

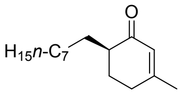
O
H15n-C7

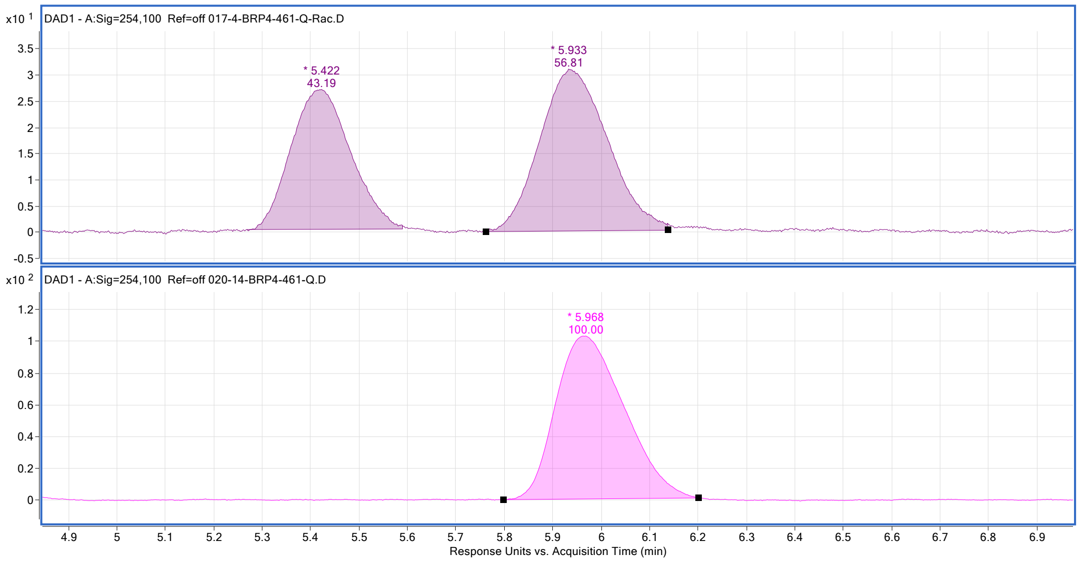
x10 1
DAD1 - A:Sig=254,100 Ref=off 017-4-BRP4-461-Q-Rac.D
* 5.422
43.19
* 5.933
56.81
x10 2
DAD1 - A:Sig=254,100 Ref=off 020-14-BRP4-461-Q.D
* 5.968
100.00
Response Units vs. Acquisition Time (min)

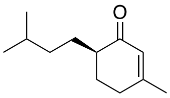
O

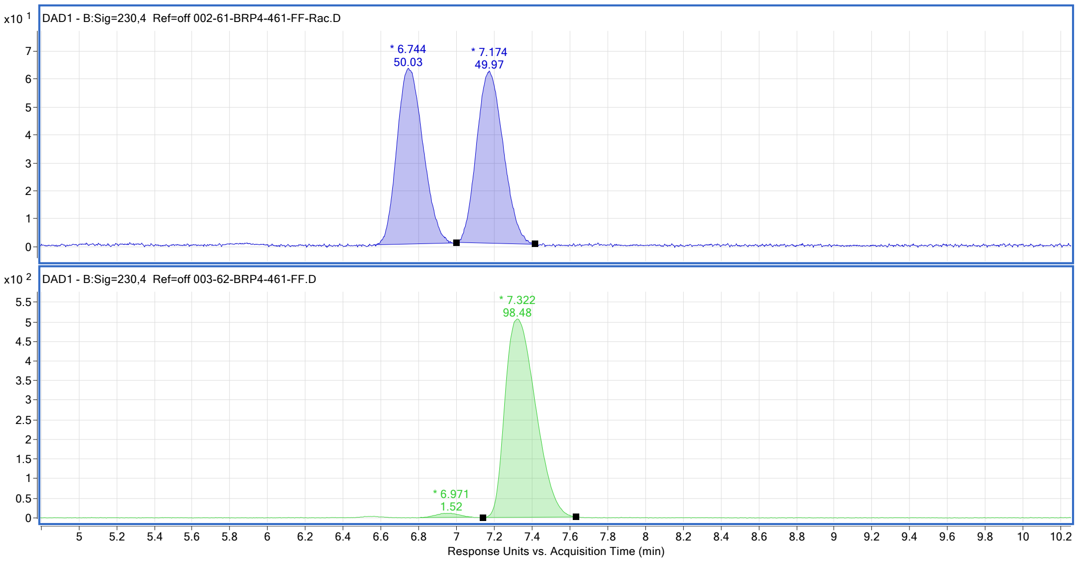
x10 1
DAD1 - B:Sig=230,4 Ref=off 002-61-BRP4-461-FF-Rac.D
* 6.744
50.03
* 7.174
49.97
x10 2
DAD1 - B:Sig=230,4 Ref=off 003-62-BRP4-461-FF.D
* 7.322
98.48
* 6.971
1.52
Response Units vs. Acquisition Time (min)

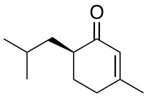
O

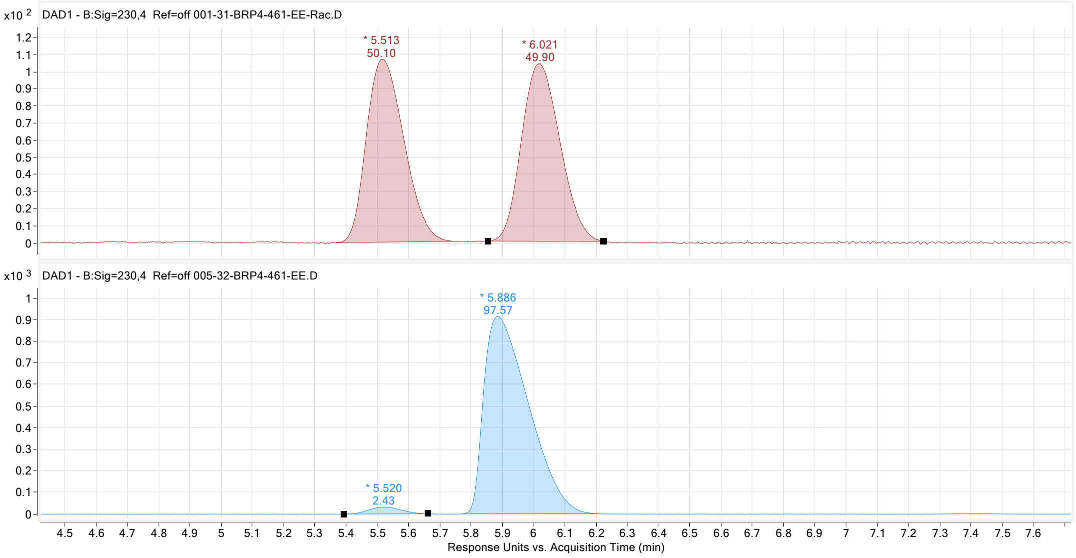
x10 2
DAD1 - B:Sig=230,4 Ref=off 001-31-BRP4-461-EE-Rac.D
* 5.513
50.10
* 6.021
49.90
x10 3
DAD1 - B:Sig=230,4 Ref=off 005-32-BRP4-461-EE.D
* 5.886
97.57
* 5.520
2.43
Response Units vs. Acquisition Time (min)

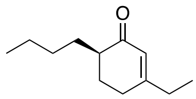

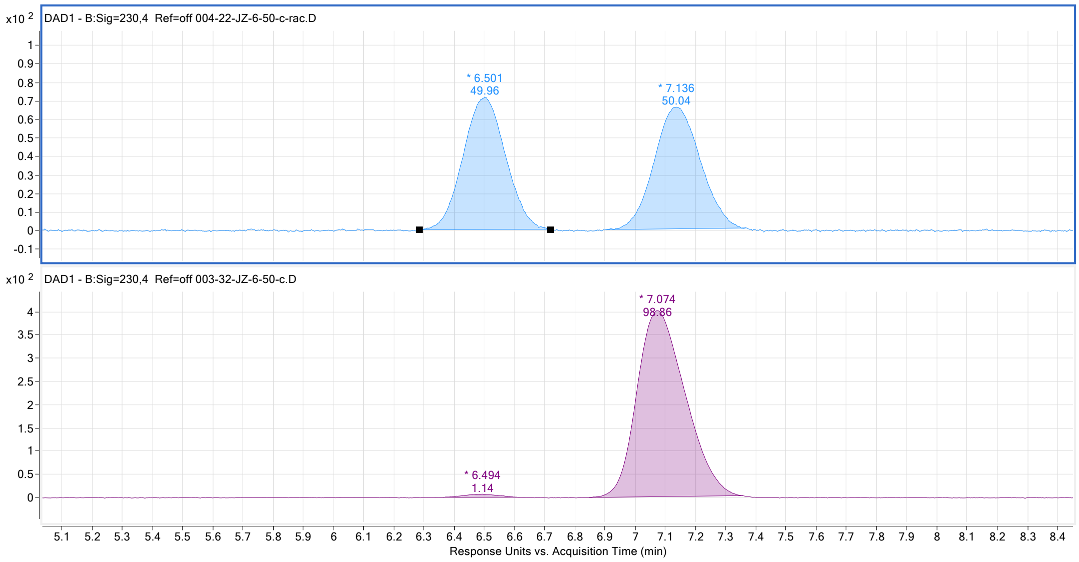

x10 2 DAD1 - B:Sig=230,4 Ref=off 004-22-JZ-6-50-c-rac.D
* 6.501
49.96
* 7.136
50.04
x10 2 DAD1 - B:Sig=230,4 Ref=off 003-32-JZ-6-50-c.D
* 7.074
98.86
* 6.494
1.14
Response Units vs. Acquisition Time (min)

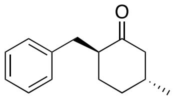

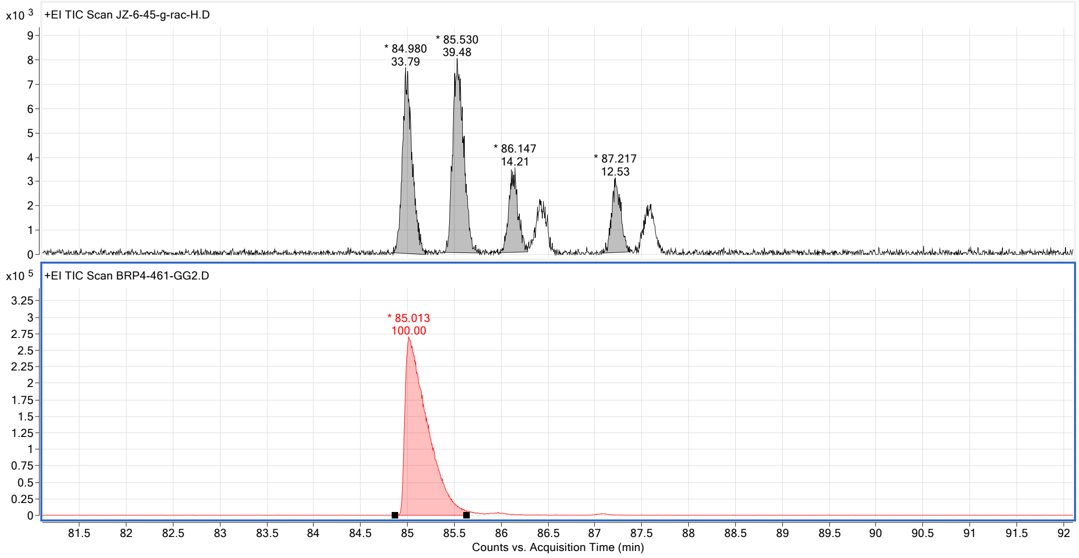

x10 3 +EI TIC Scan JZ-6-45-g-rac-H.D
* 84.980
33.79
* 85.530
39.48
* 86.147
14.21
* 87.217
12.53
x10 5 +EI TIC Scan BRP4-461-GG2.D
* 85.013
100.00
Counts vs. Acquisition Time (min)

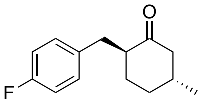
O
F

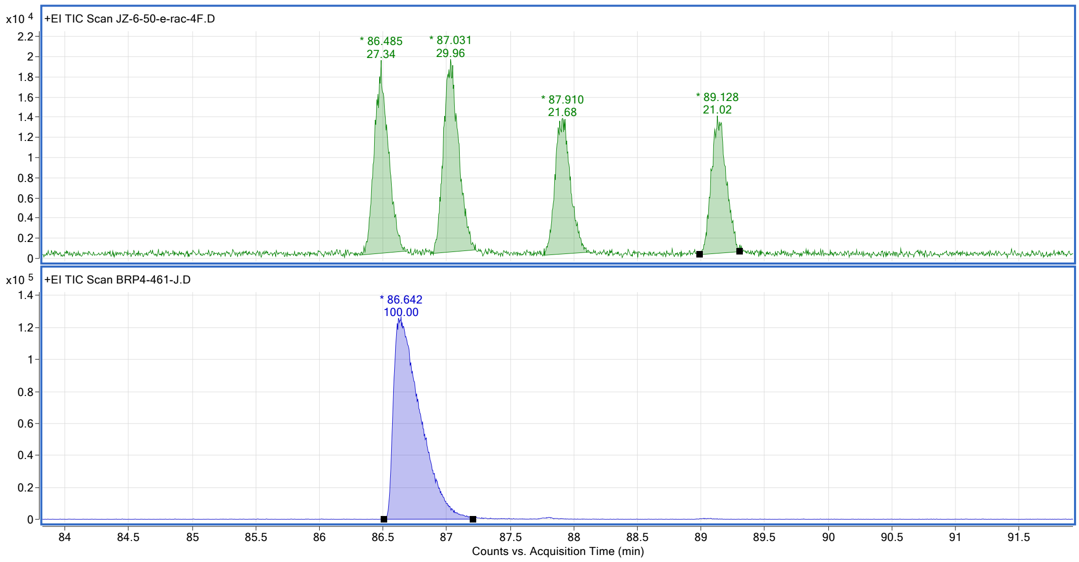
x10 4 +EI TIC Scan JZ-6-50-e-rac-4F.D
* 86.485
27.34
* 87.031
29.96
* 87.910
21.68
* 89.128
21.02
x10 5 +EI TIC Scan BRP4-461-J.D
* 86.642
100.00
Counts vs. Acquisition Time (min)

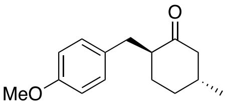
O
MeO

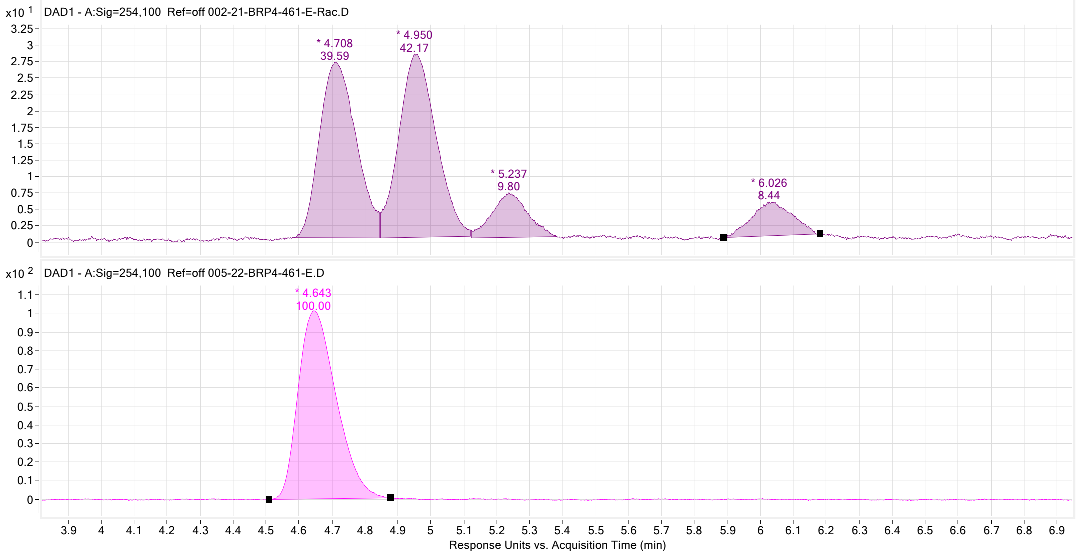
x10 1 DAD1 - A:Sig=254,100 Ref=off 002-21-BRP4-461-E-Rac.D
* 4.708
39.59
* 4.950
42.17
* 5.237
9.80
* 6.026
8.44
x10 2 DAD1 - A:Sig=254,100 Ref=off 005-22-BRP4-461-E.D
* 4.643
100.00
Response Units vs. Acquisition Time (min)

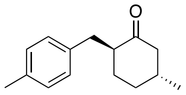

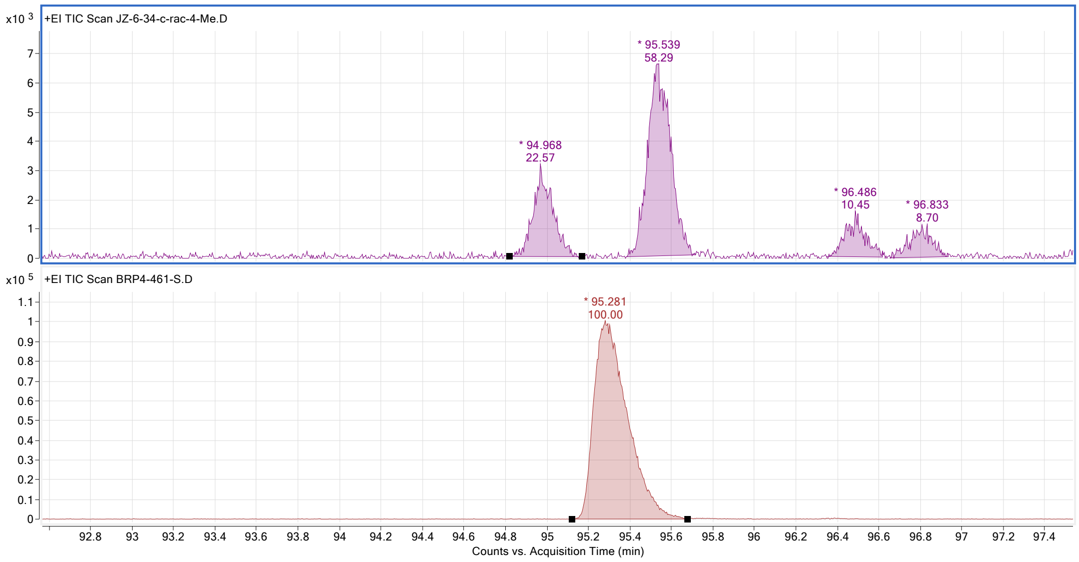

x10 3
+EI TIC Scan JZ-6-34-c-rac-4-Me.D
* 94.968
22.57
* 95.539
58.29
* 96.486
10.45
* 96.833
8.70
x10 5
+EI TIC Scan BRP4-461-S.D
* 95.281
100.00
Counts vs. Acquisition Time (min)

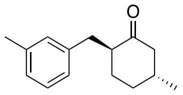

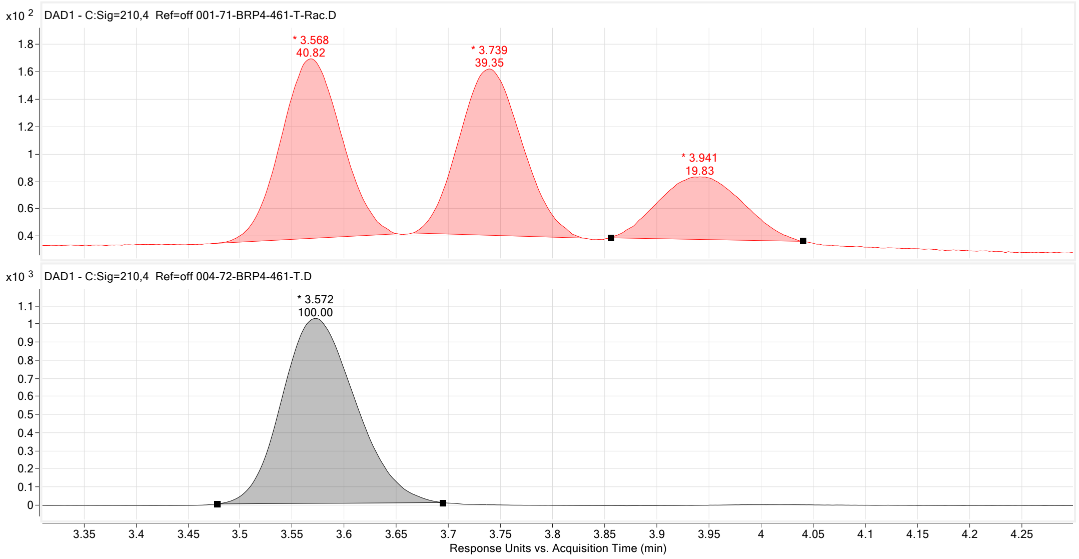

x10 2
DAD1 - C:Sig=210,4 Ref=off 001-71-BRP4-461-T-Rac.D
* 3.568
40.82
* 3.739
39.35
* 3.941
19.83
x10 3
DAD1 - C:Sig=210,4 Ref=off 004-72-BRP4-461-T.D
* 3.572
100.00
Response Units vs. Acquisition Time (min)

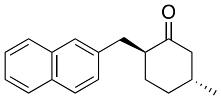

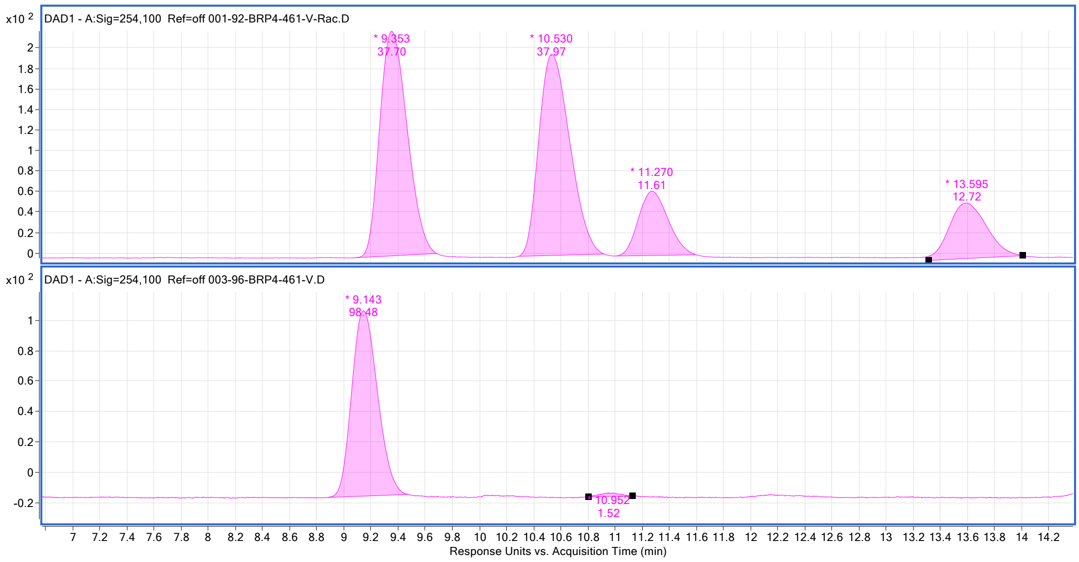
x10 2
DAD1 - A:Sig=254,100 Ref=off 001-92-BRP4-461-V-Rac.D
* 9.353
37.70
* 10.530
37.97
* 11.270
11.61
* 13.595
12.72
x10 2
DAD1 - A:Sig=254,100 Ref=off 003-96-BRP4-461-V.D
* 9.143
98.48
10.952
1.52
Response Units vs. Acquisition Time (min)

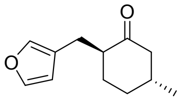

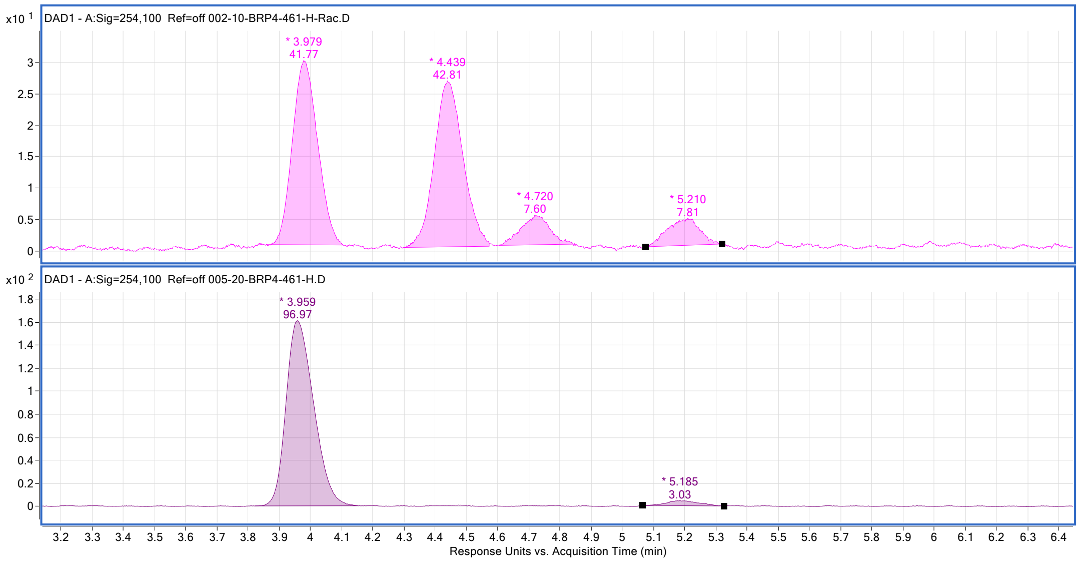
x10 1
DAD1 - A:Sig=254,100 Ref=off 002-10-BRP4-461-H-Rac.D
* 3.979
41.77
* 4.439
42.81
* 4.720
7.60
* 5.210
7.81
x10 2
DAD1 - A:Sig=254,100 Ref=off 005-20-BRP4-461-H.D
* 3.959
96.97
* 5.185
3.03
Response Units vs. Acquisition Time (min)

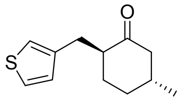

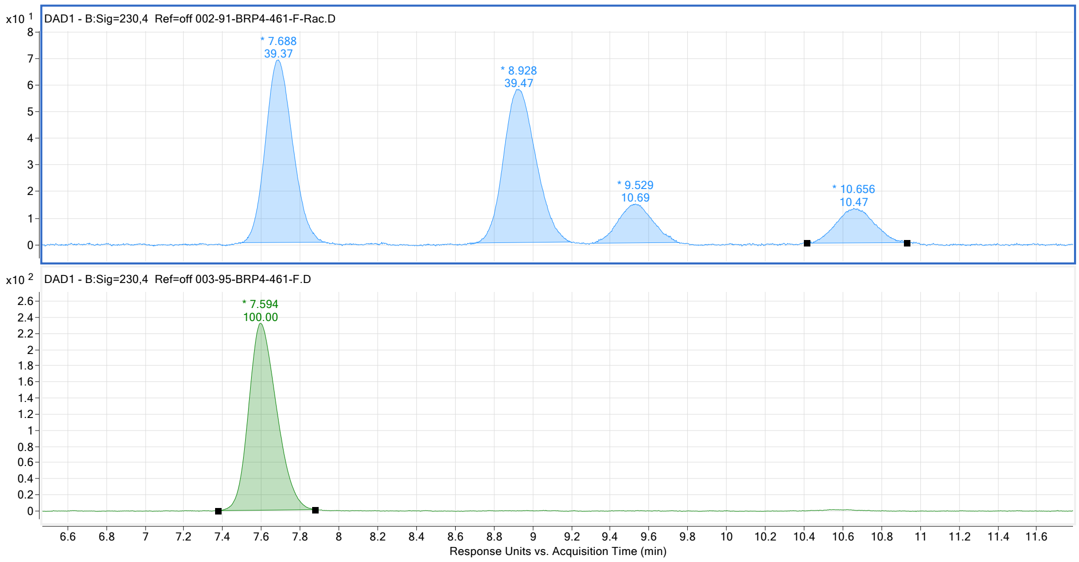

x10 1
DAD1 - B:Sig=230,4 Ref=off 002-91-BRP4-461-F-Rac.D
* 7.688
39.37
* 8.928
39.47
* 9.529
10.69
* 10.656
10.47
x10 2
DAD1 - B:Sig=230,4 Ref=off 003-95-BRP4-461-F.D
* 7.594
100.00
Response Units vs. Acquisition Time (min)

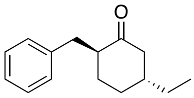

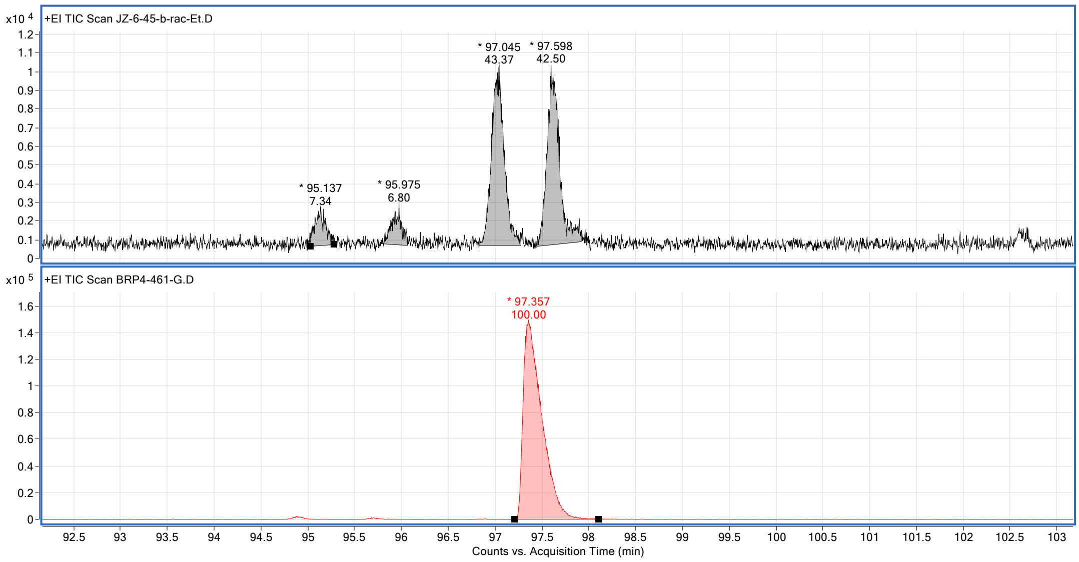

x10 4
+EI TIC Scan JZ-6-45-b-rac-Et.D
* 95.137
7.34
* 95.975
6.80
* 97.045
43.37
* 97.598
42.50
x10 5
+EI TIC Scan BRP4-461-G.D
* 97.357
100.00
Counts vs. Acquisition Time (min)

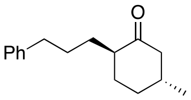
Ph
O

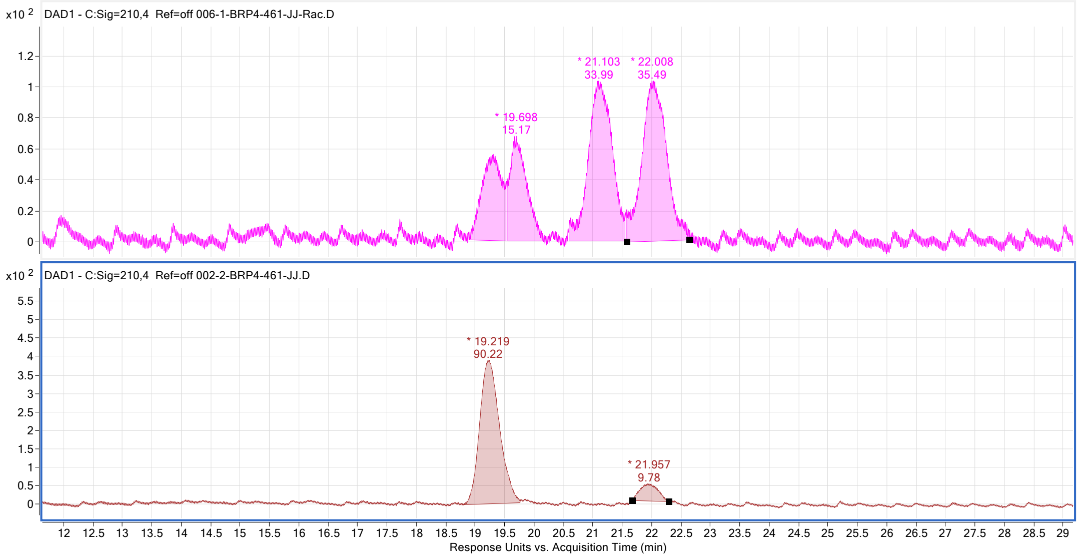
x10 2 DAD1 - C:Sig=210,4 Ref=off 006-1-BRP4-461-JJ-Rac.D
* 19.698
15.17
* 21.103
33.99
* 22.008
35.49
x10 2 DAD1 - C:Sig=210,4 Ref=off 002-2-BRP4-461-JJ.D
* 19.219
90.22
* 21.957
9.78
Response Units vs. Acquisition Time (min)

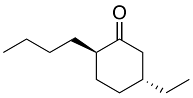
O

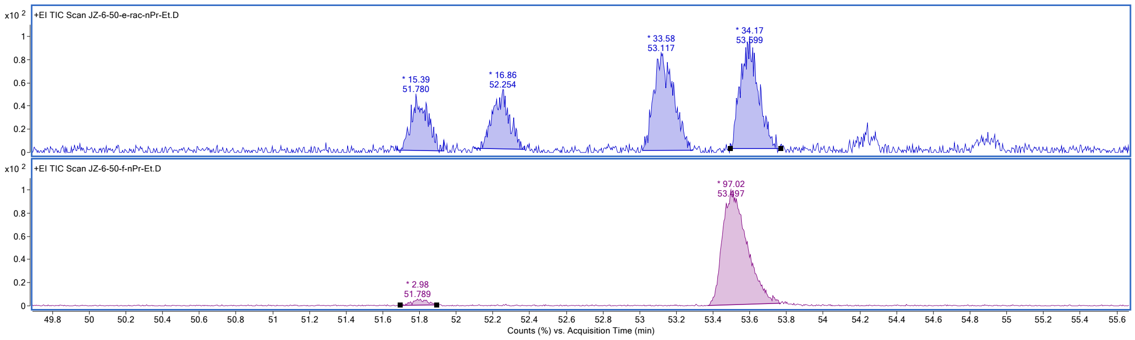
x10 2 +EI TIC Scan JZ-6-50-e-rac-nPr-Et.D
* 15.39
51.780
* 16.86
52.254
* 33.58
53.117
* 34.17
53.699
x10 2 +EI TIC Scan JZ-6-50-f-nPr-Et.D
* 2.98
51.789
* 97.02
53.697
Counts (%) vs. Acquisition Time (min)

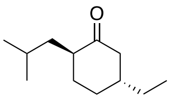
O

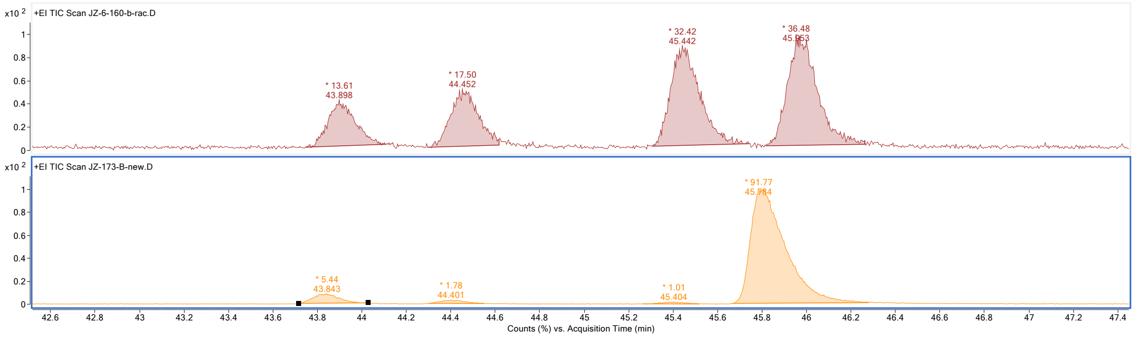
x10 2 +EI TIC Scan JZ-6-160-b-rac.D
* 13.61
43.898
* 17.50
44.452
* 32.42
45.442
* 36.48
45.963
x10 2 +EI TIC Scan JZ-173-B-new.D
* 5.44
43.843
* 1.78
44.401
* 1.01
45.404
* 91.77
45.784
Counts (%) vs. Acquisition Time (min)

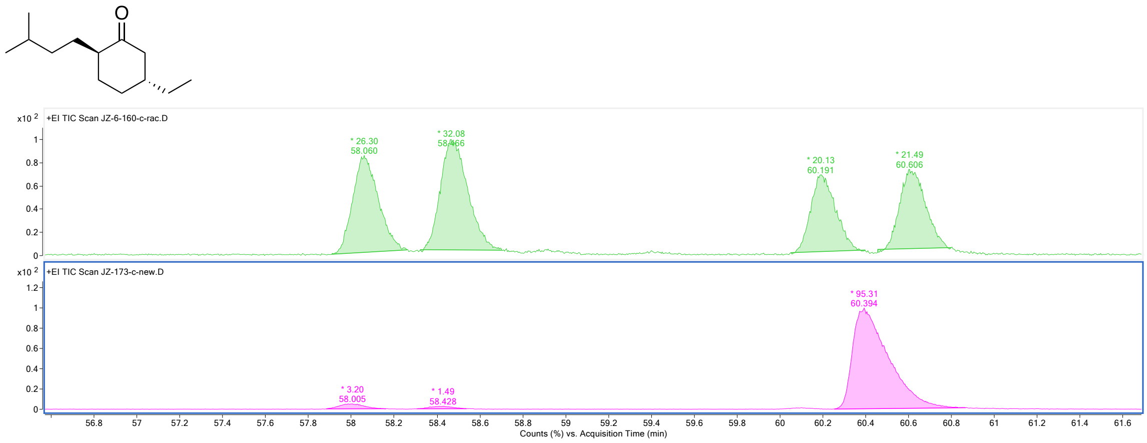
x10 2 +EI TIC Scan JZ-6-160-c-rac.D
* 26.30 58.060
* 32.08 58.466
* 20.13 60.191
* 21.49 60.606
x10 2 +EI TIC Scan JZ-173-c-new.D
* 95.31 60.394
* 3.20 58.005
* 1.49 58.428
Counts (%) vs. Acquisition Time (min)

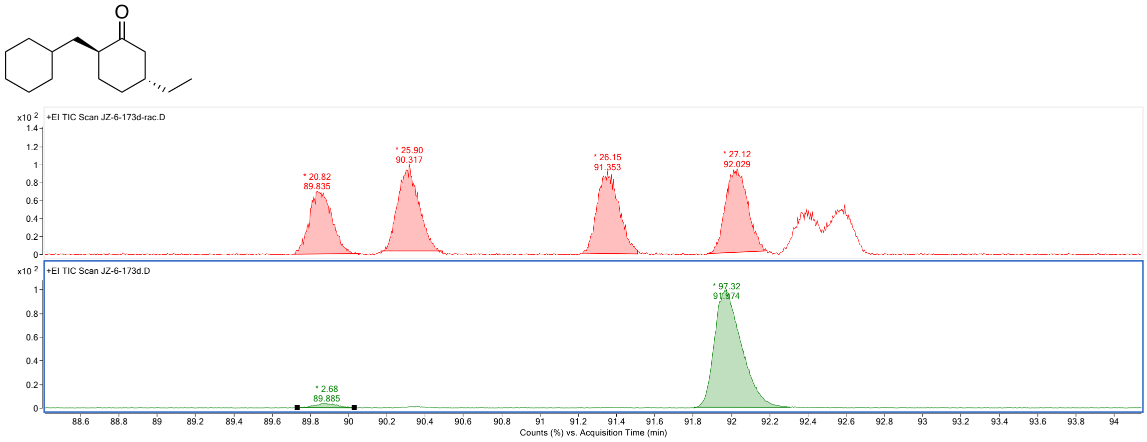
x10 2 +EI TIC Scan JZ-6-173d-rac.D
* 20.82 89.835
* 25.90 90.317
* 26.15 91.353
* 27.12 92.029
x10 2 +EI TIC Scan JZ-6-173d.D
* 97.32
* 2.68 89.885
Counts (%) vs. Acquisition Time (min)

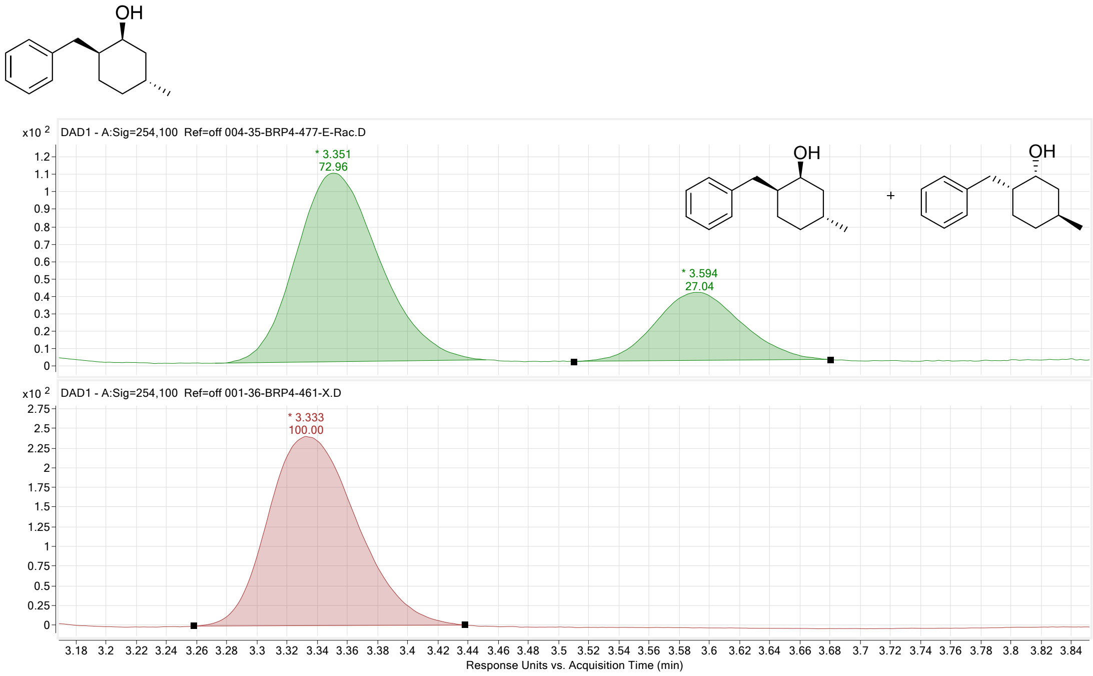
x10 2 DAD1 - A:Sig=254,100 Ref=off 004-35-BRP4-477-E-Rac.D
* 3.351 72.96
* 3.594 27.04
OH
+
OH
x10 2 DAD1 - A:Sig=254,100 Ref=off 001-36-BRP4-461-X.D
* 3.333 100.00
Response Units vs. Acquisition Time (min)

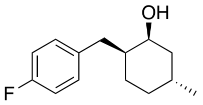

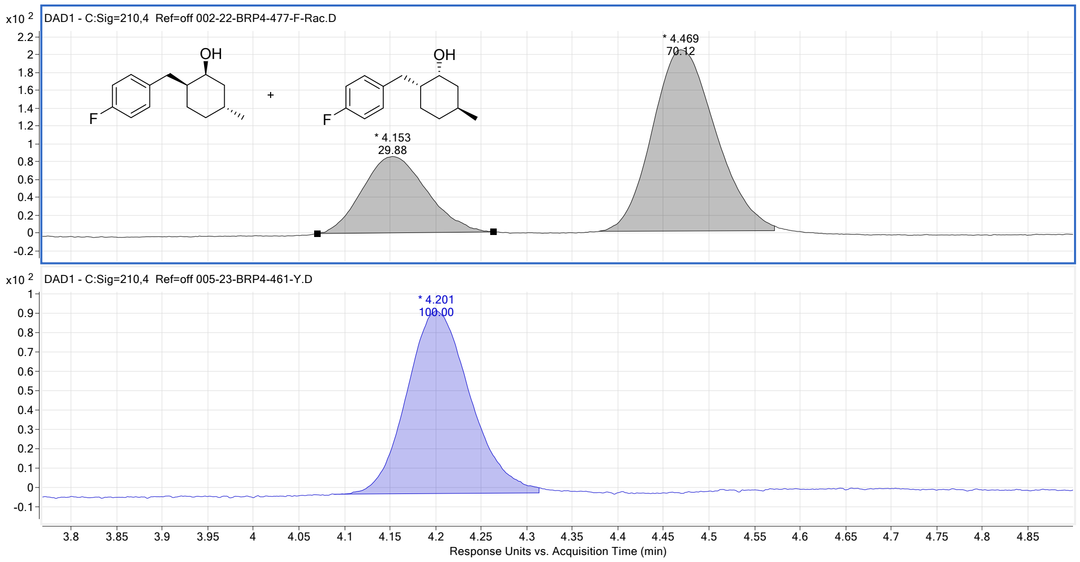

DAD1 - C:Sig=210,4 Ref=off 002-22-BRP4-477-F-Rac.D
* 4.153
29.88
* 4.469
70.12
DAD1 - C:Sig=210,4 Ref=off 005-23-BRP4-461-Y.D
* 4.201
100.00
Response Units vs. Acquisition Time (min)

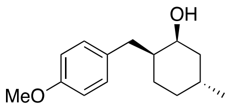

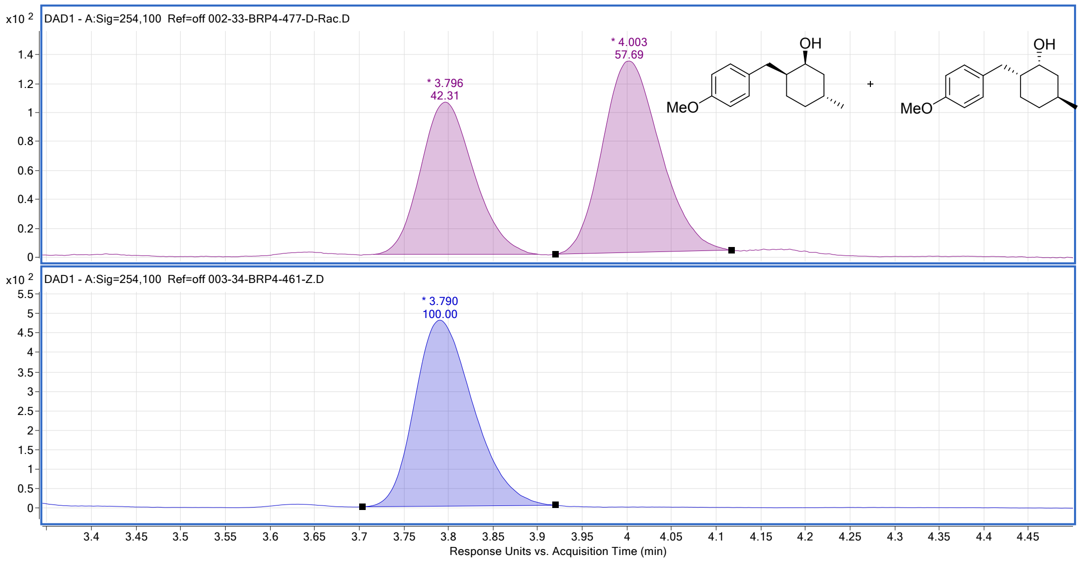

DAD1 - A:Sig=254,100 Ref=off 002-33-BRP4-477-D-Rac.D
* 3.796
42.31
* 4.003
57.69
DAD1 - A:Sig=254,100 Ref=off 003-34-BRP4-461-Z.D
* 3.790
100.00
Response Units vs. Acquisition Time (min)

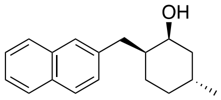

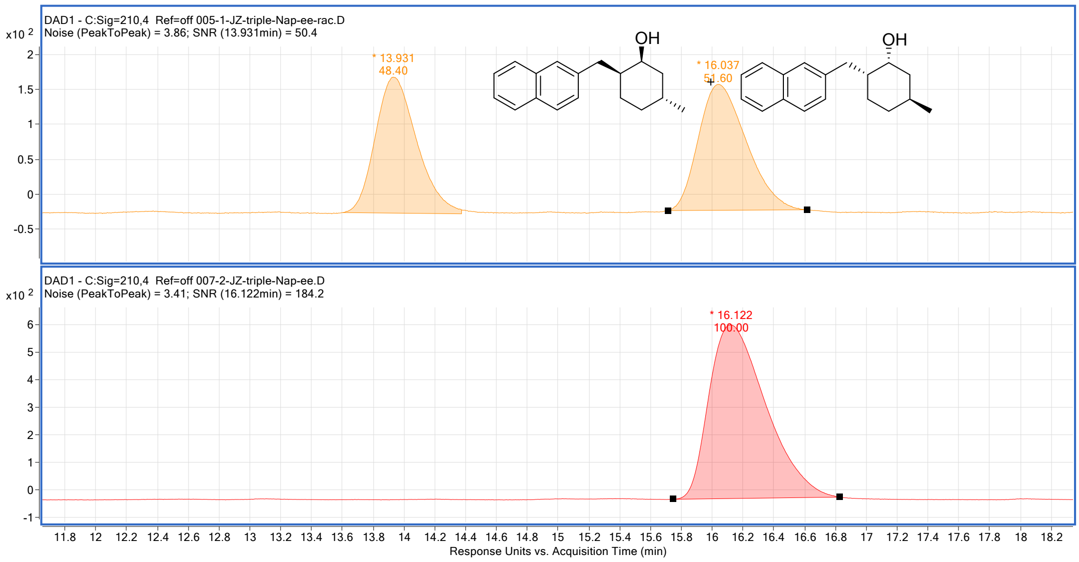

DAD1 - C:Sig=210,4 Ref=off 005-1-JZ-triple-Nap-ee-rac.D
Noise (PeakToPeak) = 3.86; SNR (13.931min) = 50.4
x10 2
* 13.931
48.40
* 16.037
51.60
OH
OH
DAD1 - C:Sig=210,4 Ref=off 007-2-JZ-triple-Nap-ee.D
Noise (PeakToPeak) = 3.41; SNR (16.122min) = 184.2
x10 2
* 16.122
100.00
Response Units vs. Acquisition Time (min)
